# Gendered pathways to adolescent mental health: An empirical assessment of a new conceptual framework

**DOI:** 10.1371/journal.pgph.0006728

**Published:** 2026-11-10

**Authors:** Anita Alaze, Daniel Hagen, Tamara Schamberger, Oliver Razum, Céline Miani

**Affiliations:** 1 Department of Epidemiology and International Public Health, School of Public Health, Bielefeld University, Bielefeld, Germany; 2 Northwell, New York City, New York, United States of America; 3 Department of Occupational Medicine, Epidemiology and Prevention, Donald and Barbara Zucker School of Medicine at Hofstra/Northwell, Hempstead, New York, United States of America; 4 Department of Psychiatry, Donald and Barbara Zucker School of Medicine at Hofstra/Northwell, Hempstead, New York, United States of America; 5 Institute of Behavioral Science, Feinstein Institutes for Medical Research, Manhasset, New York, United States of America; 6 Department Empirical Methods, Faculty of Business Administration and Economics, Bielefeld University, Bielefeld, Germany; Islamic Azad University South Tehran Branch, IRAN, ISLAMIC REPUBLIC OF

## Abstract

Gender norms and roles are important determinants of physical and mental health in the key period of adolescence. Yet, the gendered pathways to mental health in adolescents are not fully understood. Using a conceptual framework for global adolescent mental health that we developed based on a Delphi process, we empirically investigated the associations between six gender-related constructs and adolescent mental health. We used an existing cross-sectional dataset from Ethiopia (2020) from the Gender and Adolescence: Global Evidence (GAGE) project to explore the associations between sex, gender norms, psychological competencies, gender attitudes, gender roles, with the latter two also serving as mediators, and psychological distress (GHQ-12), using Structural Equation Modelling (SEM). The SEM model contained measurements from 1,651 adolescents, including 901 girls and 750 boys, with a median age of 13 years. Out of 14 pathways tested, we found statistically significant associations between psychological competencies and psychological distress; sex and psychological distress; sex and gender attitudes; and between gender norms and psychological competencies, gender attitudes, and gender roles. Hence, the gender-related constructs were mostly associated with each other, rather than with psychological distress. The gender-related constructs were associated with each other rather than with psychological distress. The mechanisms underlying these relationships and a potential interplay of gender-related pathways require longitudinal investigation.

## 1. Introduction

Adolescence is the first stage of life in which sex differences in mental health emerge in terms of morbidity and mortality [1]. Mental health problems, such as depression, anxiety and suicidality, increase sharply during this period [2]. This phenomenon and the magnitude of these problems have been reported globally [3]. These sex differences are assumed to, at least in part, stem from gender norms, roles, and relations [4]. However, it is not yet known through which specific pathways or mechanisms gender affects mental health outcomes in adolescents [1,5].

Gender norms, roles, and relations are acquired through gender socialisation, defined as the “process by which individuals learn the cultural behaviours associated with the concepts of femininity or masculinity” [6]. This process begins primarily in the *family* as parents directly or indirectly influence children to conform to expected gender norms, roles and relations [7]. The effects of gender socialisation become particularly evident during adolescence [8].

The *school environment* can reinforce the gender socialisation process through teachers (e.g., gendered role assignments) and peers (e.g., bullying due to gender non-conformity) [9]. Gender norms either incentivise or disincentivise specific health behaviours, potentially leading to adverse (mental) health outcomes [10]. “Beliefs about appropriate roles for males and females regarding the division of paid labor, homework, and childcare” [11] are often referred to as gender role attitudes, although great variability in the conceptualisation of gender roles exists. Masculine gender role characteristics are a protective factor against depression and depressive symptoms [4]. The use of *social media* can be associated with increased mental distress [12]. Media and social media use play an important role in reproducing social pressures and increase the exposure to stereotyping, objectifying and sexualising representations, which in turn strengthen beliefs in, e.g., gender stereotypes and body ideals [13]. Psychological *competencies*, such as coping skills, have been identified as strong determinants of well-being not only in individuals with mental health disorders but also in the absence of mental disorders [14].

To explore more complex gendered pathways to adolescent mental health, we previously developed a conceptual framework (hereafter referred to as the ‘GAM framework’) in an international Delphi study [15]. It encompasses six gender-related constructs, namely sex assigned at birth, gender identity, gender norms (of the social environment), gender attitudes, gender roles, psychological competencies, and mental health. As gender norms, roles, and attitudes are often conflated conceptually, we define gender norms as the “spoken and unspoken rules of societies about the acceptable behaviours of girls and boys […] - how they should act, look, and even think or feel” [10], while we define gender attitudes as the individual perceptions toward these gender norms [9]. Gender roles concern the “sexual division of labor, where specific tasks are strictly assigned based on gender” [16]. The framework also incorporates a range of socio-demographic variables that together form an intersectional lens. The gender-related constructs are hypothesised to be associated with adolescent mental health but have not yet been empirically tested. Therefore, we investigate multiple (mediated) regression models via Structural Equation Modelling (SEM) for analysis of the interlinked effects. SEM is considered an innovative approach for analysing sex/gender [17], is compatible with an intersectional approach [18], and is used in cross-sectional studies [19]. We also conduct Exploratory (EFA) and Confirmatory Factor Analysis (CFA) to validate a newly developed gender norms scale for the dataset employed in this study. The EFA serves to explore and refine the scale’s underlying factor structure, while the CFA aims to confirm the hypothesised factor model of the EFA in a confirmatory setting [20].

## 2. Materials and methods

### 2.1. Ethical approval

Our study did not require ethical approval, as it is based on the secondary analysis of previously collected, publicly available, fully anonymous, and non-identifiable third-party data.

### 2.2. Data

Our secondary data analysis draws on quantitative data from the Gender & Adolescence: Global Evidence (GAGE) project, a ten-year (2015–2024) mixed-methods longitudinal research and evaluation programme. GAGE surveys 18,000 adolescents aged 10–19 years and other community members across six countries in the Global South, with a particular focus on vulnerable adolescents, such as those with disabilities, who married before the age of 18, refugees, adolescent mothers and out-of-school youth [21]. We use cross-sectional data from the second round of data collection in Ethiopia in 2020, as it includes validated tools for mental health and gender-related variables. Data collection took place in six locations in Ethiopia. A household census was used to identify potential participants [22]. In addition to the random sample, out-of-school adolescents and adolescents with disabilities were purposefully collected by the GAGE project [21]. Sampling weights reflect the probability of selection into the study sample and ensure the representativeness of the target population in the study area. Further details on data collection procedures are described in Baird et al. [22]. The data on which the results of our study are based are third-party data that are publicly archived by the data provider ‘UK data service’ (https://ukdataservice.ac.uk/). They are available free of charge for any researcher following registration with the data provider. We have complied with the data provider’s terms of use.

### 2.3. Measures

We operationalised measures guided by the proposed operationalisations of the GAM framework [15]. Fig 1 is an adaptation of this framework based on the data available in the Ethiopian dataset.

**Fig 1:**
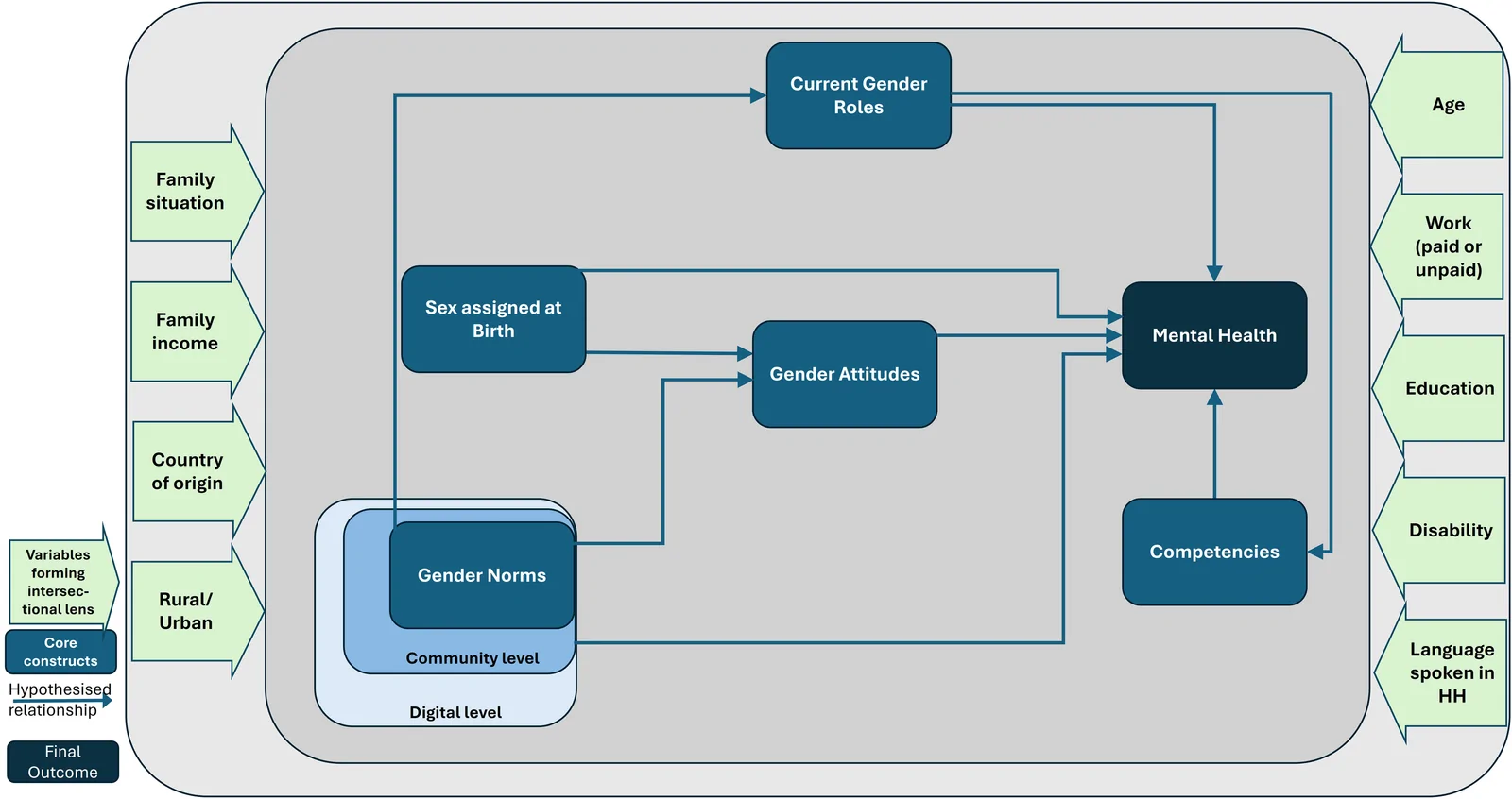
Adapted conceptual framework from a previous Delphi study [15] illustrating the operationalised constructs with the Ethiopian GAGE dataset.

We used the binary variable *sex* for the construct **sex assigned at birth**. We could not operationalise the construct of **gender identity** as no such measure exists in the dataset. While we could not operationalise the household and political levels of **gender norms** due to a lack of appropriate items, we operationalised the community level with a *peer violence index* and *being a club member*, and the digital level with *having a role model, media,* and *social media use*. We chose these operationalisations for the gender norms construct because the GAM framework operationalises this construct based on actors who carry gender norms that influence adolescents’ gender attitudes in different social environment levels, with the community level consisting of the indicators peers and school, and the digital level of media, social media, and influencers [15]. We recoded these variables so that higher values indicate higher exposure to gender norms. A detailed description of the recoded variables is shown in S1 Appendix.

We operationalised **gender attitudes** using the GAGE gender norms scale (although it is labelled as a gender norms scale, it measures adolescents’ perceptions of gender norms, that is, gender attitudes). In previous work [23], we explored the factor structure of this newly developed scale using data from an earlier GAGE round. The scale combines items from existing adolescent gender norms scales with items developed specifically for the GAGE project, but neither the original scales nor the GAGE scale have undergone prior empirical validation for the specific study population under investigation. Therefore, we explored its factor structure in the present sample. Following methodological recommendations and our previous approach, we used EFA, as the underlying factor structure could not be assumed a priori, and subsequently tested the hypothesised factor model of the EFA in a confirmatory setting using CFA (see S2 Appendix) [20]. Ultimately, the CFA suggested retaining 20 of the scale’s original 32 items as a latent variable with seven sum scores (hereafter referred to as gender attitudes subscale F1-F7). We recoded the scale so that a higher score indicates more gender egalitarian attitudes.

**Gender roles** can be defined by the sexual division of labour, where specific tasks are assigned based on gender and are operationalised in the GAM framework by time spent on gendered activities. Accordingly, we operationalised the gender roles construct with a *gendered activities score*. A higher score translates to more hours spent on activities per day, indicating adherence to more traditional gender roles.

We operationalised the construct **psychological competencies** with six instruments: a *voice* index; a r*esilience* index; an *index for talking to caregivers about work, religion, education, and bullying*; *speaking up in class*; *expression of opinion to friends;* and *to elders*. These variables were recoded so that higher values indicate a higher level of psychological competencies.

We operationalised the **mental health** construct using the validated instrument General Health Questionnaire (GHQ)-12. The GHQ-12 is a short and self-administered screening tool to detect symptoms of psychological distress and short-term changes in mental health [24]. A higher GHQ-12 score translates to increased psychological distress.

### 2.4. Statistical analysis

#### 2.4.1. Structural equation modelling.

We employed SEM to simultaneously explore the hypothesised associations between the gender-related construct and adolescent mental health using a mix of measured, observed variables and unmeasured, latent variables. SEM subsumes various other statistical techniques, including CFA, path analysis, and multiple regression analysis.

We tested for multivariate normality (Shapiro-Wilk normality test), multicollinearity (Variance Inflation Factor), and common method bias (Harman’s Single Factor Test). We then developed and tested the *measurement model* for the latent variables using CFA. We used the Chi-squared test statistics, the Bentler Comparative Fit Index (CFI), the Tucker-Lewis Index (TLI), the Goodness-of-Fit Index (GFI), the Adjusted Goodness-of-Fit Index (AGFI), the Root Mean Square Error of Approximation (RMSEA), and the Standard Root Mean Square Residual (SRMR) to assess model fit [20,25]. Thresholds for good model fits are: CFI, TLI, GFI, AGFI ≥ 0.90, RMSEA < 0.06, and SRMR < 0.08 [20,25]. We assessed localised areas of model misfit using standardised factor loadings with the commonly recommended threshold of 0.4 [26]. Due to factor loadings below 0.4, we removed the indicators social media, voice, expression of opinion to friends, expression of opinion to elders, the peer violence index, and the gender attitudes subscales F3, F5, F6, and F7. Removing these gender attitudes subscales results in a gender attitudes construct that is narrower than the validated 20-item scale. The remaining gender attitudes subscales F1, F2, and F4 in the SEM model cover 10 items from the content domains of education, violence, and time use.

We estimated parameters using maximum likelihood with robust standard errors to account for non-normality as indicated by the Shapiro-Wilk normality test. We applied sample weights to ensure representativeness of the Ethiopian adolescent population, and we treated ordinal variables with ≥5 response categories as continuous. Subsequently, we specified a *structural model* to assess the extent to which the exogenous and mediator variables explain the variance in the dependent variables psychological competencies and the outcome mental health. This resulted in five regression and four mediation models. We obtained the share of mediation as the quotient of the absolute indirect effect by the absolute total effect. Following the intersectional lens in the GAM framework, we included a range of covariates as predictors for the two exogenous variables gender norms and sex.

In addition, we conducted sensitivity analyses to test the robustness of the gender norms construct by i) removing both the club member and role model variables (Table A in S3 Appendix), ii) by removing only the club member variable (Table B in S3 Appendix), and iii) by removing only the role model variable (Table C in S3 Appendix). Statistical significance was assessed at the 5% level. We conducted all analyses in R version 4.5.2 (R Core Team, 2025). We estimated the CFA and SEM using the “lavaan” package version 0.6-21 [27], and followed reporting guidelines for SEM [28].

## 3. Results

### 3.1. Sample characteristics

Our sample included measures of 1,651 adolescents with complete data. The descriptive statistics are presented in Table 1. The sample comprised a higher number of girls (55%) than boys (45%), with an average age of 13 years (standard deviation (SD): 0.9). The average GHQ-12 score was 7.0 (SD: 3.8), which is below the validated GHQ-12 cut-off of 9 for boys and 10 for girls for psychological distress and a potential mental disorder [29]. The study participants reached a relatively high resilience score of 20 (SD: 4.0). The sample had slightly more equal than unequal gender attitudes with a value of 41 (SD: 6.7) and spent on average two hours per day on gendered activities (SD: 2.2). More adolescents expressed their opinions to their friends (78%) than to older people (45%). They talked on average about two of the four sensitive topics with their parents (SD: 2.3) and scored 19 on perceived influence in family matters. About half of the participants reported having a role model (54%) and being a member of a club (46%). 87% and 40% of adolescents reported not using social media and media, respectively, in the last 30 days. The peer violence index indicated low peer violence with 0.9 (SD: 1.8).

**Table 1:** Descriptive statistics of the variables operationalising the GAM framework (n = 1,651).

Variable	n (%) mean ± SD (range)
**Outcome**	
**Mental Health**	
**GHQ-12 score**	7.0 ± 3.8 (0-36)
**Exposures of interest**	
**Sex assigned at birth**	
**Sex**	
Boys	750 (45.4)
Girls	901 (54.6)
**Competencies**	
**Resilience score**	20.1 ± 4.0 (0-24)
**Index for talking to parents** about work, religion, education, and bullying	2.6 ± 1.2 (0-4)
**Voice score**	18.7 ± 5.1 (8-32)
**Expression of opinion to friends**	
No	371 (22.5)
Yes	1279 (77.5)
**Expression of opinion to elderly**	
No	917 (55.5)
Yes	734 (44.5)
**Gender Attitudes**	
Gender attitudes subscale F1*	10.2 ± 2.2 (4–12)
Gender attitudes subscale F2*	6.3 ± 2.1 (3–9)
Gender attitudes subscale F3*	4.9 ± 1.5 (2–6)
Gender attitudes subscale F4*	5.3 ± 2.0 (3–9)
Gender attitudes subscale F5*	5.9 ± 1.9 (4–12)
Gender attitudes subscale F6*	3.2 ± 1.3 (2–6)
Gender attitudes subscale F7*	5.4 ± 1.0 (2–6)
**Gender attitudes scale overall sum score (20 items of F1-F7)**	41.3 ± 6.7 (20-58)
**Gender Roles**	
**Gendered Activities score**	2.1 ± 2.3 (0-13)
**Gender Norms**	
**Having a role model**	
No	755 (45.7)
Yes	896 (54.3)
**Being a club member**	
No	856 (54.0)
Yes	728 (46.0)
**Social media use**	
Never	1424 (86.5)
Hardly ever	89 (5.4)
At least every week	94 (5.4)
Daily or almost daily	21 (1.3)
Several times a week	12 (0.7)
Almost all the time	7 (0.4)
**Media use**	
Never	653 (39.6)
Hardly ever	212 (12.8)
At least every week	227 (13.8)
Daily or almost daily	279 (16.9)
Several times a week	190 (11.5)
Almost all the time	90 (5.5)
**Peer violence index**	0.9 ± 1.8 (0-12)
**Covariates**	
**Age**	13.0 ± 0.9 (9–14)
**Being married**	
No	1511 (95.4)
Yes	73 (4.6)
**Urbanisation**	
Rural	1014 (61.4)
Urban	637 (38.6)
**Purposefully sampled**	
No	1504 (91.1)
Yes	147 (8.9)
**Disability**	
No	1532 (92.8)
Yes	119 (7.2)
**Language in HH**	
Afan Oromo	429 (26.0)
Afar	263 (15.9)
Amharic	933 (56.5)
Somali	26 (1.6)
**Asset index**	6.1 ± 5.1 (0-48)**
**Aspirations higher education**	
No aspirations	120 (7.3)
Secondary education	388 (23.5)
Teacher training	75 (4.5)
University education	1068 (64.7)
**Working in family business**	
No	1582 (95.8)
Yes	69 (4.2)
**Father in household**	
No	1200 (72.7)
Yes	451 (27.3)

*Reduced, validated set of gender attitudes items (2^nd^ version based on 20 gender attitudes items with seven subscales due to seven identified factors, see S2 Appendix).

**Interpretation: Participants possess 6 items across 16 asset categories in their households: mattress and/or bed; working refrigerator or freezer; working computer, laptop, or tablet; working mobile phone; working non-mobile phone; working radio; working television; working bicycle; working motorcycle, motor scooter or bajaj; working car or truck; working wrist watch / clock; working solar panel; sofa set; table; chair; plow.

### 3.2. Structural equation modelling

The fully specified SEM model included data for 1,651 adolescents from 1,651 households. We found no multicollinearity, as all variance inflation factors (VIF) were below 1.7 (threshold <5) [30]. Additionally, no common method bias was detected, with a proportion of variance of 0.22 (threshold <0.5). The SEM model simultaneously fitted latent variables, multiple regression models, and multiple mediation paths (Fig 2). In sensitivity analyses, we also modelled residual covariances and additional regression paths between latent variables and covariates using modification indices.

**Fig 2:**
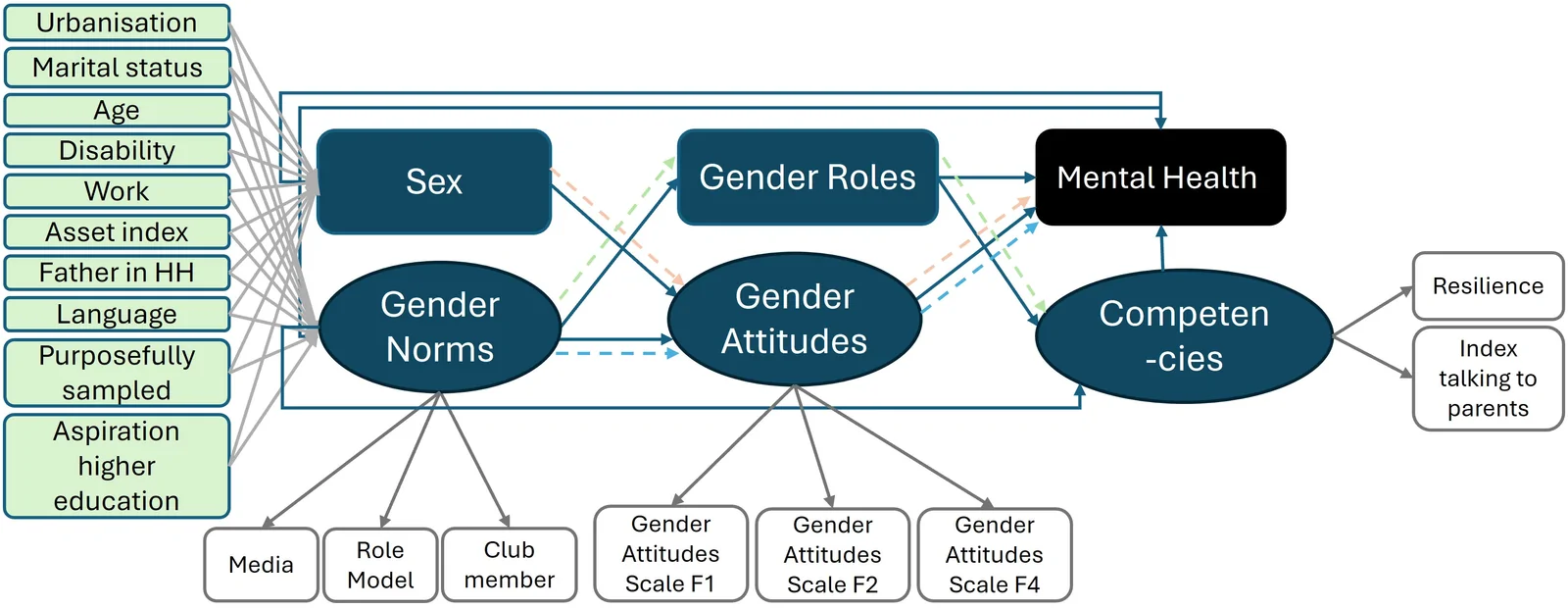
Illustration of a structural equation model examining how the gendered pathways (sex, gender norms, gender attitudes, gender roles, and competencies) influence adolescent mental health, with a set of covariates regressed on the two exogenous variables sex and gender norms. For simplicity reasons, we have omitted the error terms in the figure. *Light green box = covariates and variables forming an intersectional lens. Blue box = construct; Black box = final outcome; Grey box = indicator for the latent variable. Oval box = latent variable (measured through observed indicator variables); Rectangular box = indicator for observed variable. Full line arrow (blue) = regression path. Dashed arrow (multiple colours)  = mediation paths (one colour per mediation).*

The fit statistics of the SEM model presented a mixed picture: The RMSEA and the SRMR (≤0.06) indicated a good absolute model fit. The CFI, TLI, GFI and AGFI, with values of 0.82, 0.75, 0.91, and 0.80, respectively, fell mostly below the recommended ≥0.90 threshold. This indicates that the model does not adequately capture the data structure of the specified model. The Chi-Squared test, with χ²(155)=1041.886, p < 0.001, rejected the null hypothesis, indicating that the model does not fit the data. However, it is well known that the Chi-Squared test is sensitive to sample size and tends to reject the null hypothesis for sample sizes over 400 observations [20].

Six of ten analysed regression paths were statistically significant (Table 2). Psychological competencies were negatively associated with psychological distress, which means that a higher level of competencies was, on average, associated with lower values of psychological distress and thus better mental health. Being a girl (vs. being a boy) was also, on average, associated with lower psychological distress.

**Table 2:** Results of the model fit, the latent variables, and the regression analysis in the SEM model (N = 1,651).

Model Fit Statistics			
Fit Statistics	Value		
Chi-Squared	1041.886, p<0.001		
Df	155		
CFI	0.82		
TLI	0.75		
GFI	0.91		
AGFI	0.80		
RMSEA	0.059		
SRMR	0.038		
**Confirmatory Factor Analysis**			
**Latent Construct**	**SFL**	**FL (SE)**	
**Competencies**			
Index talking to parents	0.59	1.0 (-)	
Resilience Score	0.64	3.60 (0.28)	
**Gender Norms**			
Media use	0.59	1.0 (-)	
Having a role model	0.41	0.22 (0.02)	
Being a club member	0.44	0.24 (0.02)	
**Gender Attitudes**			
Gender Attitudes Subscale F1	0.60	1.0 (-)	
Gender Attitudes Subscale F2	0.57	0.88 (0.06)	
Gender Attitudes Subscale F4	0.61	0.87 (0.06)	
**Regression Analysis**			
**Variable**	**ß (SE)**	**95 % CI**	**p-value**
**Outcome: Mental Health**			
**Gender Attitudes**	-0.11 (0.18)	[-0.46; 0.24]	p=0.533
**Sex**			
*Ref.: Male*			
Female	**-0.49** (0.24)	[-0.96; -0.02]	p=0.040
**Gender Roles**	-0.08 (0.07)	[-0.21; 0.05]	p=0.235
**Gender Norms**	0.30 (0.34)	[-0.38; 0.98]	p=0.377
**Competencies**	**-1.71** (0.42)	[-2.53; -0.89]	p<0.001
**Outcome: Competencies**			
**Gender Roles**	0.02 (0.02)	[-0.02; 0.05]	p=0.387
**Gender Norms**	**0.68** (0.08)	[0.54; 0.83]	p<0.001
**Outcome: Gender Attitudes**			
**Gender Norms**	**0.90** (0.09)	[0.72; 1.07]	p<0.001
**Sex**			
*Ref.: Male*			
Female	**0.54** (0.09)	[0.36; 0.72]	p<0.001
**Outcome: Gender Roles**			
**Gender Norms**	**-1.37** (0.08)	[-1.53; -1.20]	p<0.001

Ref. = Reference category; ß = beta coefficient, CI = Confidence interval, SFL = Standardised factor loading, FL = Factor loading, SE = Standard error, CFI = Bentler Comparative Fit Index, TLI = Tucker-Lewis Index, GFI = Goodness-of-Fit Index, AGFI = Adjusted Goodness-of-Fit Index, RMSEA = Root Mean Square Error of Approximation, SRMR = Standard Root Mean Square Residual, Df = Degrees of freedom

Bold text = *p* < 0.05

Competencies R^2^ = 65.2%; Gender norms R^2^ = 71.0%; Gender attitudes R^2^ = 62.5%; Psychological distress R^2^ = 7.7%; Gender roles R^2^ = 34.8%; Sex R^2^ = 8.7%

Note:

1) The regression analysis was performed simultaneously in one SEM model and was adjusted for: age, work (working in family business or not working in family business), aspiration for higher education (aspiration for secondary school, teacher training, university, or no such aspiration), disability (living with disability or without disability), language spoken in the household (vernacular Afan Oromo, Afar, Amharic or Somali), urbanisation (living in urban or rural area), father living in household (living or not living in household), asset index (sum score capturing material factors in the household), sampling mode (purposeful or non-purposeful), and marital status (married before age 18 or not), regressed on the two exogeneous variables

2) Residual covariances between: gender roles and sex, gender attitudes subscale F4 and psychological distress, gender attitudes subscale F4 and female sex, media and gender roles, resilience and gender attitudes subscale F2 based on the modification indices; regressions: language Afan Oromo predicting gender attitudes (ß:-0.78, SE: 0.11, CI[-1.00; -0.56], p < 0.001), urbanisation predicting competencies (ß: 0.31, SE: 0.06, CI[0.20; 0.43], p < 0.001), language Afar predicting gender attitudes (ß: -0.64, SE:0.15, CI[-0.93; -0.34], p < 0.001) based on the modification indices.

Higher exposure to gender norms and more hours spent on gendered activities were, on average, associated with higher levels of competence, albeit only significantly for gender norms. Being a girl compared to being a boy and being more exposed to gender norms were positively and significantly associated with more egalitarian gender attitudes. Higher exposure to gender norms was negatively associated with more egalitarian gender roles, which means that higher exposure to gender norms was, on average, associated with fewer hours spent on gendered activities.

Egalitarian gender attitudes and traditional gender roles were also negatively but not significantly associated with psychological distress. In contrast, higher exposure to gender norms was positively and not significantly associated with psychological distress.

The sensitivity analyses testing the robustness of the gender norms construct showed that the differences in model fit statistics between the initial model and the three sensitivity analyses were fairly minor, and the model with both variables (club member and role model) removed showed reduced model fit statistics (Tables A, B, and C in S3 Appendix). The estimates remain stable when the variable club membership or role model is removed individually. Tables B and C in S3 Appendix. When both variables are removed. (Table A in S3 Appendix), the media-only model no longer has a positive-definite covariance matrix for the residuals of the observed variables. This means that the matrix is singular and has at least one eigenvalue that is zero or negative, which could be attributed to the now single-indicator latent construct gender norms, with media as the only remaining variable in the latent construct. This model yields three different findings: sex is no longer significantly associated with psychological distress (p = 0.058), and gender roles becomes significantly associated both with psychological distress (p = 0.044) and competencies (p < 0.001). However, due to the model estimation error, the estimates in the media-only model cannot be considered reliable.

We found no statistically significant mediation effects (Table 3). All estimated indirect effects were negative, except for the pathway from gender norms to psychological distress through egalitarian gender roles. Notably, the indirect effects of higher exposure to gender norms on psychological distress differ, as the former is modelled through two pathways, egalitarian gender attitudes and traditional gender roles, neither of which reached significance. The ratios of the total indirect effect to the total effect were 10.8%, 31.5%, 34.5% and 3.3%, respectively. The second and fourth ratios in Table 3 may represent competitive mediations, indicating suppression effects (although non-significant).

**Table 3:** Results of the mediation analysis in the SEM model (N = 1,651).

Variable	Direct effect		Indirect effect		Total indirect effect		Total effect		Ratio mediation
	Outcome: Psychological distress								
	ß-coefficient (mediator: gender attitudes):								
Sex	**-0.49**	[-0.96; 0.02], p = 0.040	-0.06	[-0.25; 0.13] p = 0.536	-0.06	[-0.25; 0.13] p = 0.536	**-0.55**	[-0.98; -0.13] p = 0.011	10.8%
Gender Norms	0.30	[-0.37; 0.98], p = 0.377	-0.10	[-0.41; 0.21] p = 0.536	0.01	[-0.35; 0.37] p = 0.959	0.31	[-0.20; 0.83] p = 0.234	31.5%^N^
	ß-coefficient (mediator: gender roles):								
Gender Norms	0.30	[-0.37; 0.98], p = 0.377	0.11	[-0.07; 0.29] p = 0.237	0.01	[-0.35; 0.37] p = 0.959	0.31	[-0.20; 0.83] p = 0.234	34.5%
	Outcome: Competencies								
	ß-coefficient (mediator: gender roles)								
Gender Norms	**0.68**	[0.54; 0.83], p < 0.001	-0.02	[-0.07; 0.03] p = 0.390	-0.02	[-0.07; 0.03] p = 0.390	**0.66**	[0.54; 0.78] p < 0.001	03.3%^N^

Square brackets = 95% Confidence Intervals

Bold text = *p* < 0.05

[] = 95% Confidence interval; p = p-value

All values are rounded to the second decimal number

^N^ = negative ratio

## 4. Discussion

### 4.1. Principal findings

In this study, we empirically tested the hypothesised pathways from the GAM framework [15]. We employed SEM to simultaneously model the set of gender-related constructs and their relationships with mental health, including a wide range of covariates to operationalise the intersectional lens applied in the framework. Based on these data collected from Ethiopian adolescents, six of ten regression paths and none of four mediation paths were statistically significant. Since the SEM model does not provide an optimal representation of the data, the significant paths should be interpreted as an initial assessment of the plausibility of the proposed pathways.

In the regression models, higher exposure to gender norms was significantly associated with both fewer hours spent on gendered activities (gender roles construct) and more egalitarian gender attitudes. The former association between gender norms (operationalised by being a member of a club, having a role model, and media use) and gender roles might be explained by an increased use of media, which was found to be associated with physical inactivity [31]. Physical inactivity may also extend to household and caregiving activities. Being enrolled in a club may also contribute to less time spent in the household and, as a result, fewer household and caregiving activities. The association between higher exposure to gender norms and more egalitarian gender attitudes supports a contact hypothesis, arguing that stereotypes are lower when individuals regularly meet, such as in sports clubs [32].

Furthermore, higher exposure to gender norms was associated with higher levels of psychological competencies. Extracurricular activities have been linked to promoting social and emotional competencies in young people [33]. Interactions with other adolescents, such as in a club, could thus contribute to developing adolescents’ competencies and ultimately promote mental health.

Higher levels of psychological competencies were negatively associated with psychological distress, indicating better mental health. This finding is consistent with the literature that psychological competencies are an important determinant of well-being, even in individuals without mental disorders [14]. The negative directionality of the significant association between female sex (vs. male sex) and psychological distress is noteworthy, indicating that being an adolescent girl is associated with lower psychological distress and thus better mental health. While girls tend to have lower mental health than boys [22] in most countries, a possible explanation for this opposite directionality in this study could be that we controlled for multiple gender-related constructs that intersect at the individual and meta-level and without which the negative effects on girls could be reversed. Moreover, countries with higher gender inequities exhibit some of the smallest gender gaps in mental health. In some cases, this gap is reversed, with boys reporting worse mental health than girls [34]. Also, lower expectations of gender equity could result in fewer feelings of both unmet expectations and relative deprivation [35]. Nevertheless, the directionality identified in this study must be confirmed in future studies, which should also investigate hypotheses regarding potential underlying mechanisms using longitudinal study designs.

The observed association of egalitarian gender attitudes with psychological distress was not significant, but existing literature would similarly indicate an association between egalitarian gender attitudes and (better) mental health [12,14,36]. The association of traditional gender roles with psychological distress was also non-significant, but is equally noteworthy as the association between sex and psychological distress, as it would suggest that traditional gender roles (i.e., more hours spent on gendered activities) could reduce psychological distress (although non-significant). Findings from countries similar to Ethiopia indicate that adherence to gender roles within a predominantly gendered normative context may contribute to better mental health, as gender roles might be more clearly defined, potentially resulting in less role conflict and a reduced double burden [34]. However, the non-significant finding and cross-sectional study design do not support this hypothesis.

We could not find evidence for mediation effects in our SEM model. The direct effect between higher exposure to gender norms and psychological distress had a positive directionality, while more egalitarian gender attitudes showed a negative, non-significant indirect effect. The same directionality is evident in the relationship between gender norms and competencies through gender roles. These positive direct and negative indirect effects may partly cancel each other out and produce a much smaller coefficient total effect. Although exploratory and non-significant, this may reflect the complex interplay between the gender-related constructs.

Consequently, the gender-related pathways analysed in the SEM model appear to be more strongly associated with each other than directly associated with mental health. This is also reflected in the low R^2^ of the primary outcome of psychological distress, which indicates that our model only explains 7.7% of the variation in mental health. Besides the fact that we controlled for gender-related constructs, another possible explanation may be the overall relatively high self-perceived mental health status of the Ethiopian adolescents. This could have limited the variability in the outcome measure and reduced the ability to detect subtle differences. Moreover, in countries with higher gender inequity, sex differences in mental health tend to be smaller. Traditional gendered expectations may be perceived as normative rather than oppressive [34], thereby mitigating their direct effect on psychological distress. Psychological competencies might be a more proximal factor for psychological distress than the other gender-related constructs and might therefore impact mental health more immediately. In this SEM model, we focused on gender-related pathways to adolescent mental health and thus may have left out other influential predictors for psychological distress.

### 4.2. Strengths and limitations

SEM is an innovative yet still underutilised statistical method for analysing sex/gender in social epidemiology [17,37]. It can be employed to simultaneously investigate potential relationships across multiple variables [17], which is compatible with an intersectional approach [18]. We followed reporting guidelines [28] and statistical recommendations for SEM and mediation analysis (e.g., large sample size, the ratio of mediations) [38]. We studied indirect and complex relationships through latent variables, which allowed us to model measurement error, as well as multiple regression and mediations [39]. Moreover, we used EFA and CFA to ensure the appropriateness of the GAGE gender attitudes scale for the study population. The findings are representative of GAGE’s target population in the study area, but may also be relevant for a similar adolescent population in countries with comparable cultural contexts.

Limitations arise from the GAGE dataset and from our analytical approach. First, we could not examine gender identity due to its absence in the dataset. This is important, as transgender and gender non-conforming adolescents often experience greater discrimination and more pronounced adverse mental health outcomes than their cisgender peers [40]. Second, despite being collected by trained interviewers, the data may be subject to social desirability bias. Third, many measures reflect perceived rather than objective measures. To mitigate this limitation, we prioritised measures that were as objective as possible. For example, gender roles were operationalised using time spent on gendered activities rather than attitudes towards gender roles, which are conceptually difficult to distinguish from other gender attitudes. Fourth, we operationalised the gender norms construct in accordance with propositions from the GAM framework. However, the indicators measure a potential exposure to gender norms rather than gender norms specifically. This may introduce measurement bias and limit construct validity.

Limitations also relate to our analytical approach. While absolute fit indices such as RMSEA and SRMR suggest a small misfit per degree of freedom and small standardised discrepancies between the observed and model-implied correlations, relative fit indices such as CFI and TLI fell below recommended thresholds, indicating that the specified model might not fully capture the underlying data structure. Several factors may contribute to this, including model complexity, large sample size, numerous covariates necessary to operationalise the intersectional lens, local model misspecifications, and the use of sample weights [39]. We investigated modification indices to identify potential sources of misfit. We incorporated residual covariances and regressions between latent variables and covariates (so as not to change the structural model) into our SEM model. We did not include any further indicators in the latent variables, as this would alter the interpretation of the constructs and thus result in an SEM model that no longer corresponds to the GAM framework. Since model evaluation should be guided by both theory and statistical fit [39], we assessed model fit by combining theoretical considerations with fit statistics. Although the remaining modification indices do not show a clear pattern suitable for any interpretations regarding other sources of misfit, a substantial unexplained misfit remains. The SEM model does not provide an optimal representation of the data but can be interpreted as an initial assessment of the plausibility of the proposed pathways. Future research should examine the proposed relationships using alternative model specifications that are not constrained by the GAM framework and adapt the framework accordingly.

Given the complexity of our SEM model, we used cross-sectional data as a first step of theory and plausibility testing. As is common in cross-sectional data, they limit the causal ordering assumption. Future research should seek to use longitudinal data to assess the causality and directionality of the effects.

### 4.3. Conclusions

We found significant associations between psychological competencies and psychological distress; sex and psychological distress; sex and gender attitudes; and between gender norms and psychological competencies, gender attitudes, and gender roles among Ethiopian adolescents. Therefore, the gender-related constructs were associated with each other rather than with psychological distress. The mechanisms underlying these relationships and a potential interplay of gender-related pathways should be further investigated using longitudinal study designs that can establish causality and better investigate dose-response relationships.

## Supporting information

S1 Appendix Recoding of the measures used in the analysis. (DOCX)

S2 Appendix Validation of the GAGE gender norms scale for the gender attitudes construct using Exploratory (EFA) and Confirmatory Factor Analysis (CFA). Methods and results of the EFA and CFA; Table A: Factor loadings and communality from the EFA; Table B: Factor loadings and model fit statistics from three CFA models.
(DOCX)

S3 Appendix Sensitivity analyses of the gender norms construct. Table A: Structural equation model without the variables club member and role model; Table B: Structural equation model without the variable club member; Table C: Structural equation model without the variable role model.
(DOCX)

S1 File Statistical analysis. R code for data cleaning and statistical analyses conducted in the manuscript.
(PDF)
