# Supplementary material for: Gendered pathways to adolescent mental health: An empirical assessment of a new conceptual framework
Source: PLOS Glob Public Health. 2026 Nov 10;6(11):e0006728. doi: 10.1371/journal.pgph.0006728 (PMC13750542; doi:10.1371/journal.pgph.0006728)
Supplement: Supplementary file 1 [file pgph.0006728.s004.pdf]

1 Preparation

2 Selecting variables

3 Recoding variables

4 Calculate reliability of scales

5 Statistical Analyses

6 Structural Equation Modelling

7 Structural Equation Models

GAGEValidationFramework

Anita Alaze

2024-09-04

1 Preparation

2 Selecting variables

2.1 Variables CR ET 2020

```
CRET20_violence_norms_ind = c("cr_vi_tolerate",
                              "cr_vi_hit",
                              "cr_vi_privematt")

CRET20_violence_norms_ind_OLD = c("cr_vi_blamed",
                              "cr_vi_stigma",
                              "cr_vi_accepted",
                              "cr_vi_ignore",
                              "cr_vi_abandon",
                              "cr_vi_demand")

CRET20_education_norms_ind = c("cr_edu_boysch",
                          "cr_edu_science",
                          "cr_edu_girlnohelp",
                          "cr_edu_raisingvoice",
                          "cr_edu_boysfeelings",
                          "cr_edu_boystough",
                          "cr_edu_boysdefend",
                          "cr_edu_girlsprotect",
                           "cr_edu_boysout",
                          "cr_edu_equaltreat",
                          "cr_edu_teachpunish",
                          "cr_edu_teachnotpunish")


CRET20_education_norms_com = c("cr_edu_culture",
                          "cr_edu_boysweak",
                          "cr_edu_girlshumble")


CRET20_time_norms_ind = c("cr_ta_eesharetasks",
                      "cr_ta_womanrole",
                      "cr_ta_eemandecid")

CRET20_financial_norms_ind = c("cr_fi_girlchance",
                           "cr_fi_goodwife",
                           "cr_fi_girlsavings")

CRET20_SRH_norms_ind_OLD = c("cr_srh_obeyhus")

CRET20_SRH_norms_ind = c("cr_srh_waitedu",
                         "cr_srh_controldaugt",
                     "cr_srh_boyfinal",
                     "cr_srh_teasegirl",
                     "cr_srh_teaseboy",
                     "cr_srh_aware",
                     "cr_srh_genderroles",
                     "cr_srh_changereaction")
```

```
CRET20_mobility = c("cr_mva_howoften_1", #also who is gender for activity & company
                    "cr_mva_howoften_2",
                    "cr_mva_howoften_3",
                    "cr_mva_howoften_4")


CRET20_voice = c("cr_mva_opinfriend",
                 "cr_mva_opinelder",
                 "cr_mva_timehwork",
                 "cr_mva_contschool",
                 "cr_mva_marrywhen",
                 "cr_mva_marrywho",
                 "cr_mva_friends",
                 "cr_mva_freetime",
                 "cr_mva_leavehouse",
                 "cr_mva_earnact",
                 "cr_mva_listen",
                 "cr_mva_opinion",
                 "cr_mva_advice",
                 "cr_mva_tell",
                 "cr_mva_speakup",
                 "cr_mva_adulthelp")

CRET20_socioemotionalskills = c("cr_edu_attainasp",
                                "cr_edu_participate",
                                "cr_hn_scale")
```

```
#note: if round 3 approves of operationalisation of gender roles as gendered tasks

CRET20_genderroles = c("cr_ta_careother",
                       "cr_ta_chores",
                       "cr_ta_workagri",
                       "cr_ta_occhildcare")
```

```
CRET20_groupmember = c("cr_si_partsport",
                        "cr_si_member_1",
                        "cr_si_member_2",
                        "cr_si_member_3",
                        "cr_si_member_4",
                        "cr_si_member_5")


CRET20_rolemodel = c("cr_si_rmyn",
                     "cr_si_rmhhwho")


CRET20_media = c("cr_ict_paper",
                 "cr_ict_radio",
                 "cr_ict_tv",
                 "cr_ict_comp",
                 "cr_ict_phone",
                 "cr_ict_airtime",
                 "cr_ict_badtext",
                 "cr_ict_socialnet",
                 "cr_ict_onlinenews",
                 "cr_ict_im",
                 "cr_ict_watchclip",
                 "cr_ict_playgame")

CRET20_peerviolencescale = c("cr_vi_numk_1",
                             "cr_vi_gender_1",
                             "cr_vi_where_1",
                             "cr_vi_peers_1",
                             "cr_vi_numk_2",
                             "cr_vi_gender_2",
                             "cr_vi_where_2",
                             "cr_vi_peers_2",
                             "cr_vi_numk_3",
                             "cr_vi_gender_3",
                             "cr_vi_where_3",
                             "cr_vi_peers_3",
                             "cr_vi_numk_4",
                             "cr_vi_gender_4",
                             "cr_vi_where_4",
                             "cr_vi_peers_4",
                             "cr_vi_numk_5",
                             "cr_vi_gender_5",
                             "cr_vi_where_5",
                             "cr_vi_peers_5",
                             "cr_vi_numk_6",
                             "cr_vi_gender_6",
                             "cr_vi_where_6",
                             "cr_vi_peers_6")

CRET20_peerpressure = c("cr_hn_pressure_1",
                        "cr_hn_pressure_2",
                        "cr_hn_pressure_3",
                        "cr_hn_pressure_4",
                        "cr_hn_pressure_5")
```

```r
CRET20_GHQ12 = c("cr_ps_ghqdistracted",
                 "cr_ps_ghqlostsleep",
                 "cr_ps_ghquseful",
                 "cr_ps_ghqcapable",
                 "cr_ps_ghqstrain",
                 "cr_ps_ghqovercome",
                 "cr_ps_ghqenjoy",
                 "cr_ps_ghqfaceprob",
                 "cr_ps_ghqunhappy",
                 "cr_ps_ghqconfident",
                 "cr_ps_ghqnoworth",
                 "cr_ps_ghqhappy")

CRET20_PHQ9 = c("cr_si_phq_interest",
                "cr_si_phq_sad",
                "cr_si_phq_sleep",
                "cr_si_phq_tired",
                "cr_si_phq_appetite",
                "cr_si_phq_failure",
                "cr_si_phq_concentrate",
                "cr_si_phq_slow",
                "cr_si_phq_selfharm")

CRET20_resilience_coping = c("cr_ps_resrolemodels",
           "cr_ps_resedu",
           "cr_ps_resparentsknow",
           "cr_ps_resfinish",
           "cr_ps_resnothurt",
           "cr_ps_reshelp",
           "cr_ps_resbelongschool",
           "cr_ps_resfamilycare",
           "cr_ps_resfriendscare",
           "cr_ps_resfair",
           "cr_ps_reslearn",
           "cr_ps_rescommunity")

CRET20_selfefficacyscale =c("cr_mva_effevent",
                         "cr_mva_effsolvewtry",
                         "cr_mva_effcalm",
                         "cr_mva_effhandle",
                         "cr_mva_trouble",
                         "cr_mva_effmeans",
                         "cr_mva_effgoal",
                         "cr_mva_effsolution",
                         "cr_mva_effsituation",
                         "cr_mva_effsolveeffort")

#done
CRET20_overallhealth = c("cr_hn_gnhlth")
```

```
CRET20_controls = c("hhid",
                  "orig_regionid",
                 "orig_woredaidp",
                 "orig_kebeleidp",
                 "orig_subkebeleidp",
                 "cr_cs_residence",
                 "sample_r2_disability",
                 "sample_r2_disability2",
                 "sample_r2_new",
                 "sample_r2_all",
                 "sample_r2_purposeful",
                 "sample_r2_random",
                 "sample_r2_xnodal_qual",
                 "sample_r2_childmarr",
                 "rand_weight",
                 "cr_si_edumother",
                 "cr_si_workmother",
                 "cr_si_romanmother",
                 "cr_si_pubmother",
                 "cr_si_marmother",
                 "cr_si_harrasmother",
                 "cr_si_relmother",
                 "cr_si_edufather",
                 "cr_si_workfather",
                 "cr_si_romanfather",
                 "cr_si_pubfather",
                 "cr_si_marfather",
                 "cr_si_harrasfather",
                 "cr_si_relfather",
                 "rand_multi_eligible_hh",
                 "cr_si_friendouthh",
                 "cr_si_friendouthhgend")

CRET20_intersectionality = c("cr_edu_attndever",
                              "cr_edu_highatt_adj",
                              "cr_edu_highedq",
                              "cr_edu_currlvl",
                              "cr_edu_currhighedq",
                              "cr_cs_crage",
                              "cr_cs_crgender",
                              "rand_young",
                              "cr_fi_control",
                              "cr_mr_married",
                              "cr_mr_marrever",
                              "cr_mr_marronce",
                              "cr_mr_agemar",
                              "cr_ta_workinhh",
                              "cr_ta_workouthh")
```

2.2 Variables AF ET 2020

```
AFET20_assets = c("af_dg_bed",
                  "af_dg_fridge",
                  "af_dg_computer",
                  "af_dg_cellphone",
                  "af_dg_landphone",
                  "af_dg_radio",
                  "af_dg_tv",
                  "af_dg_bicycle",
                  "af_dg_motor",
                  "af_dg_car",
                  "af_dg_watch",
                  "af_dg_solar",
                  "af_dg_sofa",
                  "af_dg_table",
                  "af_dg_chair",
                  "af_dg_plow")

AFET20_familysituation = c("hhid",
                           "af_hhr_childnum",
                           "af_hhr_num",
                           "af_fb_biofhh",
                           "af_fb_biofalive",
                           "af_fb_biomhh",
                           "af_fb_biomalive",
                           "af_dw_lang_recode",
                           "af_apc_incsource",
                           "af_sn_psnp_yn")
```

2.3 Creating dataframes

```
data_CRET20_prefinal %>% summarise_all(~ sum(is.na(.)))
```

```
## # A tibble: 1 × 206
##   cr_edu_attndever cr_edu_highatt_adj cr_edu_highedq cr_edu_currlvl
##              <int>              <int>          <int>          <int>
## 1                0               3685           4502           1767
## # ℹ 202 more variables: cr_edu_currhighedq <int>, cr_cs_crage <int>,
## #   cr_cs_crgender <int>, rand_young <int>, cr_fi_control <int>,
## #   cr_mr_married <int>, cr_mr_marrever <int>, cr_mr_marronce <int>,
## #   cr_mr_agemar <int>, cr_ta_workinhh <int>, cr_ta_workouthh <int>,
## #   hhid <int>, orig_regionid <int>, orig_woredaidp <int>,
## #   orig_kebeleidp <int>, orig_subkebeleidp <int>, cr_cs_residence <int>,
## #   sample_r2_disability <int>, sample_r2_disability2 <int>, …
```

```
data_CRET20_prefinal %>% summarise_all(~ max(.))
```

```
## # A tibble: 1 × 206
##   cr_edu_attndever cr_edu_highatt_adj cr_edu_highedq cr_edu_currlvl
##   <dbl+lbl>        <dbl+lbl>          <dbl+lbl>      <dbl+lbl>
## 1 2 [No]           NA                 NA             NA
## # ℹ 202 more variables: cr_edu_currhighedq <dbl+lbl>, cr_cs_crage <dbl>,
## #   cr_cs_crgender <dbl>, rand_young <dbl>, cr_fi_control <dbl+lbl>,
## #   cr_mr_married <dbl+lbl>, cr_mr_marrever <dbl+lbl>,
## #   cr_mr_marronce <dbl+lbl>, cr_mr_agemar <dbl>, cr_ta_workinhh <dbl>,
## #   cr_ta_workouthh <dbl>, hhid <dbl>, orig_regionid <dbl+lbl>,
## #   orig_woredaidp <dbl>, orig_kebeleidp <dbl>, orig_subkebeleidp <dbl>,
## #   cr_cs_residence <dbl+lbl>, sample_r2_disability <dbl+lbl>, …
```

```
data_AFET20_prefinal %>% summarise_all(~ sum(is.na(.)))
```

```
## # A tibble: 1 × 26
##   af_dg_bed af_dg_fridge af_dg_computer af_dg_cellphone af_dg_landphone
##       <int>        <int>          <int>           <int>           <int>
## 1         0            0              0               0               0
## # ℹ 21 more variables: af_dg_radio <int>, af_dg_tv <int>, af_dg_bicycle <int>,
## #   af_dg_motor <int>, af_dg_car <int>, af_dg_watch <int>, af_dg_solar <int>,
## #   af_dg_sofa <int>, af_dg_table <int>, af_dg_chair <int>, af_dg_plow <int>,
## #   hhid <int>, af_hhr_childnum <int>, af_hhr_num <int>, af_fb_biofhh <int>,
## #   af_fb_biofalive <int>, af_fb_biomhh <int>, af_fb_biomalive <int>,
## #   af_dw_lang_recode <int>, af_apc_incsource <int>, af_sn_psnp_yn <int>
```

```
data_AFET20_prefinal %>% summarise_all(~ max(.))
```

```
## # A tibble: 1 × 26
##   af_dg_bed af_dg_fridge af_dg_computer af_dg_cellphone af_dg_landphone
##       <dbl>        <dbl>          <dbl>           <dbl>           <dbl>
## 1         9            4              5              19               3
## # ℹ 21 more variables: af_dg_radio <dbl>, af_dg_tv <dbl>, af_dg_bicycle <dbl>,
## #   af_dg_motor <dbl>, af_dg_car <dbl>, af_dg_watch <dbl>, af_dg_solar <dbl>,
## #   af_dg_sofa <dbl>, af_dg_table <dbl>, af_dg_chair <dbl>, af_dg_plow <dbl>,
## #   hhid <dbl>, af_hhr_childnum <dbl>, af_hhr_num <dbl>,
## #   af_fb_biofhh <dbl+lbl>, af_fb_biofalive <dbl+lbl>, af_fb_biomhh <dbl+lbl>,
## #   af_fb_biomalive <dbl+lbl>, af_dw_lang_recode <dbl>,
## #   af_apc_incsource <dbl+lbl>, af_sn_psnp_yn <dbl+lbl>
```

3 Recoding variables

3.1 Variables CR ET 2020

```
data_CRET20_rec = data_CRET20_prefinal %>%
  dplyr::select(-all_of(CRET20_SRH_norms_ind_OLD)) %>%
  dplyr::select(-all_of(CRET20_violence_norms_ind_OLD)) %>%
  mutate(across(everything(), ~ na_if(., -96))) %>%
  mutate(across(everything(), ~ na_if(., -97))) %>%
  mutate(across(everything(), ~ na_if(., -99))) %>%
  mutate(across(everything(), ~ na_if(., -98))) %>%
  mutate(
    cr_ta_eesharetasks = 4 - cr_ta_eesharetasks,
    cr_fi_girlchance = 4 - cr_fi_girlchance,
    cr_fi_girlsavings = 4 - cr_fi_girlsavings,
    cr_srh_waitedu = 4 - cr_srh_waitedu,
    cr_srh_changereaction = 4 - cr_srh_changereaction,
    cr_gendernormscale_overall = (cr_ta_womanrole+ cr_edu_raisingvoice+ cr_ta_eemandeci
d+ cr_vi_tolerate+ cr_fi_girlchance+ cr_edu_equaltreat+ cr_edu_teachpunish+ cr_edu_girl
nohelp+ cr_edu_boysch+ cr_edu_boystough+ cr_vi_hit+ cr_srh_teaseboy+ cr_edu_boysdefend+
cr_srh_teasegirl+ cr_edu_boysout+ cr_edu_science+ cr_edu_teachnotpunish+ cr_vi_privemat
t+ cr_fi_girlsavings+ cr_edu_boysfeelings),

     cr_gendernormscale12 = (cr_edu_boysch+ cr_edu_science+ cr_edu_girlnohelp+ cr_edu_r
aisingvoice+  cr_edu_boysout+ cr_ta_womanrole+ cr_ta_eemandecid+ cr_srh_teasegirl+ cr_s
rh_teaseboy+cr_vi_hit+ cr_vi_privematt+ cr_vi_tolerate),
    cr_gendernormscale12rec = cr_gendernormscale12 - min(cr_gendernormscale12, na.rm =
TRUE),

cr_gendernormscale_f1 = (cr_edu_science+cr_edu_boysch+cr_edu_girlnohelp+cr_edu_boysou
t),
cr_gendernormscale_f2 = (cr_vi_tolerate+cr_vi_hit+cr_vi_privematt),
cr_gendernormscale_f3 = (cr_srh_teasegirl+cr_srh_teaseboy),
cr_gendernormscale_f4 = (cr_ta_womanrole+ cr_edu_raisingvoice+ cr_ta_eemandecid),
cr_gendernormscale_f5 = (cr_edu_boystough+cr_edu_boysdefend+cr_edu_boysfeelings+cr_edu_
equaltreat),
cr_gendernormscale_f6 = (cr_edu_teachpunish+cr_edu_teachnotpunish),
cr_gendernormscale_f7 = (cr_fi_girlsavings+cr_fi_girlchance),

    ################ MENTAL HEALTH ##############################

    #PHQ-9 Score (0-27)
    cr_phq9 = (cr_si_phq_interest + cr_si_phq_sad + cr_si_phq_sleep + cr_si_phq_tired +
cr_si_phq_appetite + cr_si_phq_failure + cr_si_phq_concentrate + cr_si_phq_slow + cr_si
_phq_selfharm),
    #GHQ-12 score 4-point Likert scale (I chose to not use the binary scale model)
  #create sum score (the negative items are already reversed)
    cr_ghq12 = (cr_ps_ghqdistracted + cr_ps_ghqlostsleep + cr_ps_ghquseful + cr_ps_ghqc
apable + cr_ps_ghqstrain + cr_ps_ghqovercome + cr_ps_ghqenjoy + cr_ps_ghqfaceprob + cr_
ps_ghqunhappy + cr_ps_ghqconfident + cr_ps_ghqnoworth + cr_ps_ghqhappy),
    #GHQ-12 cut-off for psychological distress, cut-off 14 (between 13 and 14: https://
pmc.ncbi.nlm.nih.gov/articles/PMC9987474/)
    #cr_ghq12_distress = case_when(cr_ghq12>=14 ~1, TRUE~0, TRUE ~ NA_real_),
#=1 if comfortable expressing an opinion to a friend
cr_opfriend = case_when(cr_mva_opinfriend==1~1, cr_mva_opinfriend==2~0, TRUE ~ NA_real
_),
#=1 if comfortable expressing an opinion to an elder
```

```
cr_opelder = case_when(cr_mva_opinelder==1 ~1, cr_mva_opinelder==2 ~0, TRUE ~ NA_real
_),
#cr_selfhealth=1 if self-reported health is good or very good
                    cr_selfhealth = case_when(cr_hn_gnhlth%in%c(1,2)~ 1, cr_hn_gnhlth%i
n%c(3,4,5) ~ 0, TRUE ~ NA_real_),
    #self-efficacy scale score/GSE scale, 10-40, only collected for older cohort (proba
bly not needed for our analysis)
    cr_selfeff = (cr_mva_effevent + cr_mva_effsolvewtry + cr_mva_effcalm + cr_mva_effha
ndle + cr_mva_trouble + cr_mva_effmeans + cr_mva_effgoal + cr_mva_effsolution + cr_mva_
effsituation + cr_mva_effsolveweffort),

##################### VOICE AND AGENCY ###################################

#cr_trustfr==1 when cr has friends they trust
cr_trustfr = case_when(cr_si_friendouthh==2 ~ 0, cr_si_friendouthh==1 ~ 1, TRUE ~ NA_re
al_),
#cr_talk_religion==1 if cr talked to f/m about religion
cr_talk_religion = case_when(cr_si_relfather==1 | cr_si_relmother==1~1, cr_si_relfather
==2 | cr_si_relmother==2~0,  TRUE ~ NA_real_),
#cr_talk_bullying==1 if cr talked to f/m about bullying/harrasment
cr_talk_bullying = case_when(cr_si_harrasfather==1 | cr_si_harrasmother==1~1, cr_si_har
rasfather==2 | cr_si_harrasmother==2~0,  TRUE ~ NA_real_),
#cr_talk_ed==1 if cr talked to f/m about education
cr_talk_ed = case_when(cr_si_edufather==1 | cr_si_edumother==1~1,
                       cr_si_edufather==2 | cr_si_edumother==2~0,  TRUE ~ NA_real_),
#cr_talk_ed==1 if cr talked to f/m about work
cr_talk_work = case_when(cr_si_workfather==1 | cr_si_workmother==1~1, cr_si_workfather=
=2 | cr_si_workmother==2~0,  TRUE ~ NA_real_),
#generate index cr_talk = cr_talk_ed + cr_talk_bullying + cr_talk_religion
cr_talk = (cr_talk_ed + cr_talk_bullying + cr_talk_religion + cr_talk_work),
#voice
cr_voice= (cr_mva_timehwork + cr_mva_contschool + cr_mva_marrywhen + cr_mva_marrywho +
cr_mva_friends + cr_mva_freetime + cr_mva_leavehouse + cr_mva_earnact),
#cr_speakup=1 if cr often speaks up in class
cr_speakup = case_when(cr_edu_participate==3 ~ 1, cr_edu_participate==2 | cr_edu_partic
ipate==1 ~ 0,   TRUE ~ NA_real_),
#cr_speakup ordinal
cr_speakup_ord = case_when(cr_edu_participate==3 ~ 3, cr_edu_participate==2 ~ 2, cr_edu
_participate==1~1, TRUE ~ NA_real_),

#resilience/coping variables into score (0=no, 1=sometimes, 2=yes)
cr_ps_resrolemodels_rec = case_when(cr_ps_resrolemodels==1~0, cr_ps_resrolemodels==2~1,
cr_ps_resrolemodels==3~2, TRUE ~ NA_real_),
cr_ps_resedu_rec = case_when(cr_ps_resedu==1~0, cr_ps_resedu==2~1, cr_ps_resedu==3~2, T
RUE ~ NA_real_),
cr_ps_resparentsknow_rec = case_when(cr_ps_resparentsknow==1~0, cr_ps_resparentsknow==2
~1, cr_ps_resparentsknow==3~2, TRUE ~ NA_real_),
cr_ps_resfinish_rec = case_when(cr_ps_resfinish==1~0, cr_ps_resfinish==2~1, cr_ps_resfi
nish==3~2, TRUE ~ NA_real_),
cr_ps_resnothurt_rec = case_when(cr_ps_resnothurt==1~0, cr_ps_resnothurt==2~1, cr_ps_re
snothurt==3~2, TRUE ~ NA_real_),
cr_ps_reshelp_rec = case_when(cr_ps_reshelp==1~0, cr_ps_reshelp==2~1, cr_ps_reshelp==3~
2, TRUE ~ NA_real_),
cr_ps_resbelongschool_rec = case_when(cr_ps_resbelongschool==1~0, cr_ps_resbelongschool
==2~1, cr_ps_resbelongschool==3~2, TRUE ~ NA_real_),
cr_ps_resfamilycare_rec = case_when(cr_ps_resfamilycare==1~0, cr_ps_resfamilycare==2~1,
```

```
cr_ps_resfamilycare==3~2, TRUE ~ NA_real_),
cr_ps_resfriendscare_rec = case_when(cr_ps_resfriendscare==1~0, cr_ps_resfriendscare==2
~1, cr_ps_resfriendscare==3~2, TRUE ~ NA_real_),
cr_ps_resfair_rec = case_when(cr_ps_resfair==1~0, cr_ps_resfair==2~1, cr_ps_resfair==3~
2, TRUE ~ NA_real_),
cr_ps_reslearn_rec = case_when(cr_ps_reslearn==1~0, cr_ps_reslearn==2~1, cr_ps_reslearn
==3~2, TRUE ~ NA_real_),
cr_ps_rescommunity_rec = case_when(cr_ps_rescommunity==1~0, cr_ps_rescommunity==2~1, cr
_ps_rescommunity==3~2, TRUE ~ NA_real_),
cr_copingresilience = (cr_ps_resrolemodels_rec + cr_ps_resedu_rec + cr_ps_resparentskno
w_rec + cr_ps_resfinish_rec + cr_ps_resnothurt_rec + cr_ps_reshelp_rec + cr_ps_resbelon
gschool_rec + cr_ps_resfamilycare_rec + cr_ps_resfriendscare_rec + cr_ps_resfair_rec +
cr_ps_reslearn_rec + cr_ps_rescommunity_rec),

######gender roles ##############

#gender roles operationalised by time spend on gendered activities, values=hours spent
on activity
cr_genderedactivities = (cr_ta_careother + cr_ta_chores),
#I had to take cr_ta_occhildcare out due to NAs


########################## SOCIAL ENVIRONMENT ######################

#recode media use, keep ordinal structure with 1=Never; 2=Hardly ever; 3=At least every
week; 4=Daily or almost daily; 5=Several times a week; 6=Almost all the time
cr_media = case_when(
  cr_ict_paper == 7 | cr_ict_radio == 7 | cr_ict_tv == 7 | cr_ict_onlinenews == 6 ~ 6,
cr_ict_paper == 6 | cr_ict_radio == 6 | cr_ict_tv == 6 | cr_ict_onlinenews == 5 ~ 5,
cr_ict_paper == 5 | cr_ict_radio == 5 | cr_ict_tv == 5 | cr_ict_onlinenews == 4 ~ 4,
cr_ict_paper == 4 | cr_ict_radio == 4 | cr_ict_tv == 4 | cr_ict_onlinenews == 3 ~ 3, cr
_ict_paper %in% c(2, 3) | cr_ict_radio %in% c(2, 3) | cr_ict_tv %in% c(2, 3) | cr_ict_o
nlinenews == 2 ~ 2,
cr_ict_paper == 1 | cr_ict_radio == 1 | cr_ict_tv == 1 | cr_ict_onlinenews == 1 ~ 1,
      TRUE ~ NA_real_),

#recode social media use, keep ordinal structure with 1=Never; 2=Hardly ever; 3=At leas
t every week; 4=Daily or almost daily; 5=Several times a week; 6=Almost all the time
cr_socialmedia = case_when(cr_ict_comp==7 | cr_ict_socialnet==6 | cr_ict_im==6 | cr_ict
_watchclip==6 | cr_ict_playgame==6 ~6,
cr_ict_comp==6 | cr_ict_socialnet==5 | cr_ict_im==5 | cr_ict_watchclip==5 | cr_ict_play
game==5 ~5,
cr_ict_comp==5 | cr_ict_socialnet==4 | cr_ict_im==4 | cr_ict_watchclip==4 | cr_ict_play
game==4 ~4,
cr_ict_comp==4 | cr_ict_socialnet==3 | cr_ict_im==3 | cr_ict_watchclip==3 | cr_ict_play
game==3 ~3,
cr_ict_comp==2 | cr_ict_comp==3 | cr_ict_socialnet==2 | cr_ict_im==2 | cr_ict_watchclip
==2 | cr_ict_playgame==2 ~2,
cr_ict_comp==1 | cr_ict_socialnet==1 | cr_ict_im==1 | cr_ict_watchclip==1 | cr_ict_play
game==1 ~1,TRUE ~ NA_real_),

#recode all member variables into var indicating whether individual is a member of any
club 1=Yes, 0=No
cr_clubmember = case_when(cr_si_member_1==1 | cr_si_member_2==1 | cr_si_member_3==1 | c
r_si_member_4==1 | cr_si_member_5==1 ~1,
cr_si_member_1==2 | cr_si_member_2==2 | cr_si_member_3==2 | cr_si_member_4==2 | cr_si_m
```

```
ember_5==2 ~0, TRUE ~ NA_real_),

#cr_rolemodel=1 if cr has role model #2834 NAs
cr_rolemodel = case_when(cr_si_rmyn==2 ~ 0, cr_si_rmyn==1 ~ 1, TRUE ~ NA_real_),
#cr_rolemodel_outside, =1 if cr has rolemodels outside the household many NAs!
cr_rolemodel_outside = case_when(cr_si_rmhhwho==1 | cr_si_rmhhwho==2 | cr_si_rmhhwho==3
| cr_si_rmhhwho==4 | cr_si_rmhhwho==5 | cr_si_rmhhwho==6 | cr_si_rmhhwho==7 | cr_si_rmh
hwho==8 | cr_si_rmhhwho==9 ~ 0,
cr_si_rmhhwho==10 | cr_si_rmhhwho==11 | cr_si_rmhhwho==12 | cr_si_rmhhwho==13 | cr_si_r
mhhwho==14 | cr_si_rmhhwho==15 | cr_si_rmhhwho==16 | cr_si_rmhhwho==17 ~1, TRUE ~ NA_re
al_),
#role model famous
cr_rolemodel_famous = case_when(cr_si_rmhhwho==16 ~ 1, cr_si_rmhhwho %in% 1:15 | cr_si_
rmhhwho==17~0, TRUE ~ NA_real_),

#peer pressure
cr_peerpressure = (cr_hn_pressure_1 + cr_hn_pressure_2 + cr_hn_pressure_3 + cr_hn_press
ure_4),

#Peer violence scale
#*Peer Violence Scale - ranges from 0-6; constructed as the sum of indicators for
#*has experienced (at least once in the 12 months preceeding survey), either in person
#*or digitally, violence from peers, including: hurtful words, exclusion,
#*theft/damage of personal property, physical violence, being forced to do something
#*or threats to you or someone close to you. Not asked if aged >=13 and married
#*#recoding binary as in GAGE 0-6
cr_vi_numk_1_rec = case_when(cr_vi_numk_1==1~0, cr_vi_numk_1 %in% c(2,3)~1, TRUE ~ NA_r
eal_),
cr_vi_numk_2_rec = case_when(cr_vi_numk_2==1~0, cr_vi_numk_2 %in% c(2,3)~1, TRUE ~ NA_r
eal_),
cr_vi_numk_3_rec = case_when(cr_vi_numk_3==1~0, cr_vi_numk_3 %in% c(2,3)~1, TRUE ~ NA_r
eal_),
cr_vi_numk_4_rec = case_when(cr_vi_numk_4==1~0, cr_vi_numk_4 %in% c(2,3)~1, TRUE ~ NA_r
eal_),
cr_vi_numk_5_rec = case_when(cr_vi_numk_5==1~0, cr_vi_numk_5 %in% c(2,3)~1, TRUE ~ NA_r
eal_),
cr_vi_numk_6_rec = case_when(cr_vi_numk_6==1~0, cr_vi_numk_6 %in% c(2,3)~1, TRUE ~ NA_r
eal_),
#recoding ordinal
cr_vi_numk_1_rec1 = case_when(cr_vi_numk_1==1~0, cr_vi_numk_1==2~1, cr_vi_numk_1==3~2,
TRUE ~ NA_real_),
cr_vi_numk_2_rec1 = case_when(cr_vi_numk_2==1~0,cr_vi_numk_2==2~1, cr_vi_numk_2==3~2, T
RUE ~ NA_real_),
cr_vi_numk_3_rec1 = case_when(cr_vi_numk_3==1~0, cr_vi_numk_3==2~1, cr_vi_numk_3==3~2,
TRUE ~ NA_real_),
cr_vi_numk_4_rec1 = case_when(cr_vi_numk_4==1~0, cr_vi_numk_4==2~1, cr_vi_numk_4==3~2,
TRUE ~ NA_real_),
cr_vi_numk_5_rec1 = case_when(cr_vi_numk_5==1~0, cr_vi_numk_5==2~1, cr_vi_numk_5==3~2,
TRUE ~ NA_real_),
cr_vi_numk_6_rec1 = case_when(cr_vi_numk_6==1~0, cr_vi_numk_6==2~1, cr_vi_numk_6==3~2,
TRUE ~ NA_real_),
peervio_binary = (cr_vi_numk_1_rec + cr_vi_numk_2_rec + cr_vi_numk_3_rec + cr_vi_numk_4
_rec +cr_vi_numk_5_rec + cr_vi_numk_6_rec),
peervio_ordinal = (cr_vi_numk_1_rec1 + cr_vi_numk_2_rec1 + cr_vi_numk_3_rec1 + cr_vi_nu
mk_4_rec1 +cr_vi_numk_5_rec1 + cr_vi_numk_6_rec1),
```

```
################### CONTROLS ########################
#cr_female=1 when cr is female
cr_female = case_when(cr_cs_crgender==1 ~ 0, cr_cs_crgender==2 ~ 1, TRUE ~ NA_real_),
#cr_urban==1 if cr lives in urban area, if correct, Debre Tabor, Dire Dawa, Batu are th
e urban sites
cr_urban = case_when(orig_woredaidp==4 | orig_woredaidp==3 | orig_woredaidp==5~1, orig_
woredaidp%in%1:2 |orig_woredaidp==6 ~0, TRUE ~ NA_real_),
#CR aspirations for highest level of education (18=Teacher Training, vocational and rel
igious have 0 obs ->excluded)
#working in family business
cr_workfambus = case_when(cr_ta_workinhh==0  ~0, cr_ta_workinhh>=1 ~1, TRUE ~ NA_real
_),
#aspirations for educational attainment
cr_aspiratt = case_when(cr_edu_attainasp==0 ~0, cr_edu_attainasp==201 ~1, cr_edu_attain
asp==202 ~2,cr_edu_attainasp==203 ~3, cr_edu_attainasp==204 ~4, cr_edu_attainasp==205 ~
5, cr_edu_attainasp==206 ~6, cr_edu_attainasp==207 ~7, cr_edu_attainasp==208 ~8, cr_edu
_attainasp==209 ~9, cr_edu_attainasp==210 ~10, cr_edu_attainasp==211 ~11, cr_edu_attain
asp==212 ~12, cr_edu_attainasp==218 ~13, cr_edu_attainasp==219 ~ 14, TRUE ~ NA_real_),
#cr aspires secondary school, teacher or uni
#=1 if cr aspires to attain secondary school (209,210,211,212)
#=2 if cr aspires at least some vocational/teacher/religious training 13
#=3 if cr aspires at least some uiversity 14
cr_aspiratt_higheredu = case_when(cr_aspiratt==9 | cr_aspiratt==10 | cr_aspiratt==11 |
cr_aspiratt==12 ~1,
cr_aspiratt==13 ~2, cr_aspiratt==14 ~3, cr_aspiratt %in% 0:8 ~ 0, TRUE ~ NA_real_),

#=1 if cr is or was married
cr_married = case_when(sample_r2_childmarr==1 ~1, sample_r2_childmarr==0 ~0,TRUE ~ NA_r
eal_),
#purpsamp==1 when cr is purposely sampled ->make sure this is comparable with ET r1!!!!
purpsamp = case_when(sample_r2_purposeful==1 |  sample_r2_random==0 ~ 1, sample_r2_purp
oseful==0 |  sample_r2_random==1 ~ 0, TRUE ~ NA_real_),


#determining the grade of students: 1-12 are grade 1 to grade 12, (13 is university)
cr_edu_currlvl_rec = case_when(cr_edu_currlvl==201 ~1, cr_edu_currlvl==202 ~2, cr_edu_c
urrlvl==203 ~3, cr_edu_currlvl==204 ~4, cr_edu_currlvl==205 ~5, cr_edu_currlvl==206 ~6,
cr_edu_currlvl==207 ~7, cr_edu_currlvl==208 ~8, cr_edu_currlvl==209 ~9, cr_edu_currlvl=
=210 ~10, cr_edu_currlvl==211 ~11, cr_edu_currlvl==212 ~12, cr_edu_currlvl==200 | cr_ed
u_currlvl %in% 213:223 ~0, TRUE ~ NA_real_),
#age-for-grade
cr_age_for_grade = (cr_edu_currlvl_rec + 5 - cr_cs_crage)
)
```

3.2 Remove unnecessary variables

```
#remove non-relevant variables
data_CRET20_final = data_CRET20_rec %>% dplyr::select(-c( cr_edu_currlvl, cr_mr_marrie
d, cr_mr_marrever, cr_mr_marronce, cr_si_edumother, cr_si_workmother, cr_si_romanmothe
r, cr_si_friendouthh, cr_si_pubmother, cr_si_friendouthhgend, cr_si_marmother, cr_si_ha
rrasmother, cr_si_relmother, cr_si_relmother, cr_si_edufather, cr_si_workfather, cr_si_
romanfather, cr_si_marfather, cr_si_pubfather, cr_si_harrasfather, cr_si_relfather, cr_
si_member_1, cr_si_member_2, cr_si_member_3, cr_si_member_4, cr_si_member_5, cr_si_rmhh
who, cr_si_rmyn, cr_edu_attainasp, cr_edu_participate, cr_ict_paper, cr_ict_playgame, c
r_ict_comp, cr_ict_radio, cr_ict_im, cr_ict_tv, cr_ict_socialnet, cr_ict_onlinenews, cr
_ict_watchclip,                                           cr_cs_crgender, cr_ta_workinh
h, cr_vi_numk_1,cr_vi_gender_1, cr_vi_where_1, cr_vi_peers_1, cr_vi_numk_2, cr_vi_gende
r_2, cr_vi_where_2, cr_vi_peers_2, cr_vi_numk_3, cr_vi_gender_3, cr_vi_where_3, cr_vi_p
eers_3, cr_vi_numk_4, cr_vi_gender_4, cr_vi_where_4, cr_vi_peers_4, cr_vi_numk_5, cr_vi
_gender_5, cr_vi_where_5, cr_vi_peers_5, cr_vi_numk_6, cr_vi_gender_6, cr_vi_where_6, c
r_vi_peers_6, orig_regionid, orig_woredaidp, orig_kebeleidp, orig_subkebeleidp, cr_cs_r
esidence, sample_r2_disability, sample_r2_new, sample_r2_all, sample_r2_purposeful,samp
le_r2_random, sample_r2_xnodal_qual, cr_ict_phone, cr_ict_airtime, cr_ict_badtext, cr_e
du_highatt_adj, cr_edu_currhighedq, cr_edu_highedq, cr_mva_howoften_1, cr_mva_howoften_
2, cr_mva_howoften_3,  cr_mva_howoften_4))
```

3.3 Variables AF ET 2020

```
#recode data from caregiver dataset
data_AFET20_prefinal <- data_AFET20_prefinal %>%
  mutate(across(everything(), ~ na_if(., -96))) %>%
  mutate(across(everything(), ~ na_if(., -97))) %>%
  mutate(across(everything(), ~ na_if(., -99))) %>%
  mutate(across(everything(), ~ na_if(., -98))) %>%
  mutate(
    af_asset_index=(af_dg_bed+ af_dg_fridge+ af_dg_computer+ af_dg_cellphone+ af_dg_lan
dphone+ af_dg_radio+ af_dg_tv+ af_dg_bicycle+ af_dg_motor+ af_dg_car+ af_dg_watch+ af_d
g_solar+ af_dg_sofa+ af_dg_table+ af_dg_chair+ af_dg_plow),
    af_asset_10 = ntile(af_asset_index, 10),
    #6+7 zusammen in (non)gov support, 9 (property rent) und 8 in 7 "other"
    familyincome=case_when(af_apc_incsource==1~1, af_apc_incsource==2 ~2, af_apc_incsou
rce==3~3, af_apc_incsource==4~4, af_apc_incsource==5~5, af_apc_incsource==6~6, af_apc_i
ncsource==7~6, af_apc_incsource==8~7, af_apc_incsource==9~7, TRUE ~ NA_real_)
     )
label(data_AFET20_prefinal$af_asset_10) <- "Decile of assets"
frq(data_AFET20_prefinal$af_asset_index)
```

```
## How many mattresses and/or bed(s) does the household own? (x) <numeric>
## # total N=4857 valid N=4855 mean=6.59 sd=5.11
##
## Value |   N | Raw % | Valid % | Cum. %
## --------------------------------------
##     0 | 105 |  2.16 |    2.16 |   2.16
##     1 | 338 |  6.96 |    6.96 |   9.12
##     2 | 533 | 10.97 |   10.98 |  20.10
##     3 | 573 | 11.80 |   11.80 |  31.91
##     4 | 519 | 10.69 |   10.69 |  42.60
##     5 | 460 |  9.47 |    9.47 |  52.07
##     6 | 371 |  7.64 |    7.64 |  59.71
##     7 | 311 |  6.40 |    6.41 |  66.12
##     8 | 298 |  6.14 |    6.14 |  72.26
##     9 | 249 |  5.13 |    5.13 |  77.38
##    10 | 212 |  4.36 |    4.37 |  81.75
##    11 | 173 |  3.56 |    3.56 |  85.31
##    12 | 143 |  2.94 |    2.95 |  88.26
##    13 | 122 |  2.51 |    2.51 |  90.77
##    14 |  93 |  1.91 |    1.92 |  92.69
##    15 |  74 |  1.52 |    1.52 |  94.21
##    16 |  55 |  1.13 |    1.13 |  95.35
##    17 |  41 |  0.84 |    0.84 |  96.19
##    18 |  35 |  0.72 |    0.72 |  96.91
##    19 |  25 |  0.51 |    0.51 |  97.43
##    20 |  31 |  0.64 |    0.64 |  98.06
##    21 |  14 |  0.29 |    0.29 |  98.35
##    22 |  21 |  0.43 |    0.43 |  98.78
##    23 |  11 |  0.23 |    0.23 |  99.01
##    24 |   7 |  0.14 |    0.14 |  99.16
##    25 |   7 |  0.14 |    0.14 |  99.30
##    26 |   6 |  0.12 |    0.12 |  99.42
##    27 |   3 |  0.06 |    0.06 |  99.49
##    28 |   3 |  0.06 |    0.06 |  99.55
##    29 |   7 |  0.14 |    0.14 |  99.69
##    30 |   3 |  0.06 |    0.06 |  99.75
##    31 |   3 |  0.06 |    0.06 |  99.81
##    34 |   1 |  0.02 |    0.02 |  99.84
##    35 |   2 |  0.04 |    0.04 |  99.88
##    39 |   1 |  0.02 |    0.02 |  99.90
##    40 |   1 |  0.02 |    0.02 |  99.92
##    42 |   1 |  0.02 |    0.02 |  99.94
##    43 |   1 |  0.02 |    0.02 |  99.96
##    47 |   1 |  0.02 |    0.02 |  99.98
##    48 |   1 |  0.02 |    0.02 | 100.00
##  <NA> |   2 |  0.04 |    <NA> |   <NA>
```

```
frq(data_AFET20_prefinal$af_asset_10)
```

```
## Decile of assets (x) <integer>
## # total N=4857 valid N=4855 mean=5.50 sd=2.87
##
## Value |   N | Raw % | Valid % | Cum. %
## ------------------------------------
##     1 | 486 | 10.01 |   10.01 |  10.01
##     2 | 486 | 10.01 |   10.01 |  20.02
##     3 | 486 | 10.01 |   10.01 |  30.03
##     4 | 486 | 10.01 |   10.01 |  40.04
##     5 | 486 | 10.01 |   10.01 |  50.05
##     6 | 485 |  9.99 |    9.99 |  60.04
##     7 | 485 |  9.99 |    9.99 |  70.03
##     8 | 485 |  9.99 |    9.99 |  80.02
##     9 | 485 |  9.99 |    9.99 |  90.01
##    10 | 485 |  9.99 |    9.99 | 100.00
##  <NA> |   2 |  0.04 |    <NA> |   <NA>
```

```
frq(data_AFET20_prefinal$familyincome)
```

```
## x <numeric>
## # total N=4857 valid N=4570 mean=3.14 sd=1.91
##
## Value |    N | Raw % | Valid % | Cum. %
## -------------------------------------
##     1 | 1572 | 32.37 |   34.40 |  34.40
##     2 |  335 |  6.90 |    7.33 |  41.73
##     3 |  653 | 13.44 |   14.29 |  56.02
##     4 |  575 | 11.84 |   12.58 |  68.60
##     5 |  978 | 20.14 |   21.40 |  90.00
##     6 |  237 |  4.88 |    5.19 |  95.19
##     7 |  220 |  4.53 |    4.81 | 100.00
##  <NA> |  287 |  5.91 |    <NA> |   <NA>
```

```
frq(data_AFET20_prefinal$af_apc_incsource)
```

```
## Warning: The table contains very wide columns that don't fit into the available
##   display-width of the console. Splitting tables into multiple parts did
##   not have the desired effect.
```

```
## What is your household's main source of income? (x) <numeric>
## # total N=4857 valid N=4570 mean=3.22 sd=2.10
##
## Value
## -----
##   -97
##   -96
##     1
##     2
##     3
##     4
##     5
##     6
##     7
##     8
##     9
##  <NA>
##
##
Label
## ----------------------------------------------------------------------------------------
----------------------------------
##
Refused
##
Other (specify)
##                                                    Food crop farming (crops grown pri
marily for household consumption).
##                                                          Cash crop farming (crops gro
wn primary for sale in the market)
##                                                                        Livestock rais
ing, fishing, or fishpond culture.
## Wage and salary employment (including work that is paid in cash or in kind, includin
g both agriculture and other work)
##     Other non-farm economic activities (including  things like running a small busin
ess, self-employment, buy-and-sell
##                                                                                   Tra
nsfers/support from the government
##                                                                     Transfers/support
from non-government organizations
##                                                        Transfers/support from individ
uals who are not household members
##
House/property rent
##
<NA>
##
##    N | Raw % | Valid % | Cum. %
## ------------------------------
##    0 |  0.00 |    0.00 |   0.00
##    0 |  0.00 |    0.00 |   0.00
## 1572 | 32.37 |   34.40 |  34.40
##  335 |  6.90 |    7.33 |  41.73
##  653 | 13.44 |   14.29 |  56.02
##  575 | 11.84 |   12.58 |  68.60
```

```
##  978 | 20.14 |   21.40 |  90.00
##  205 |  4.22 |    4.49 |  94.49
##   32 |  0.66 |    0.70 |  95.19
##   89 |  1.83 |    1.95 |  97.13
##  131 |  2.70 |    2.87 | 100.00
##  287 |  5.91 |    <NA> |   <NA>
```

```
data_AFET20_prefinal$familyincome = labelled(data_AFET20_prefinal$familyincome, labels=
c(
  "Food crop farming" = 1,
  "Cash crop farming" = 2,
  "Lifestock raising" = 3,
  "Employed" = 4,
  "Selfemployed" = 5,
  "(Non)government support" = 6,
  "Other"=7))
```

3.4 Combining datasets

```
#combine the recoded variables from both datasets
data_ET20_final <- left_join(data_CRET20_final, data_AFET20_prefinal, by = "hhid")
```

4 Calculate reliability of scales

```
#create subset with 20-item GAGE gender norms scale
data_ET20_subset_GNS_CFA = data_ET20_final %>%  dplyr::select(c(cr_edu_boysch, cr_edu_s
cience, cr_edu_girlnohelp, cr_edu_raisingvoice, cr_edu_boysfeelings, cr_edu_boystough,
cr_edu_boysdefend, cr_edu_equaltreat, cr_edu_teachpunish, cr_edu_teachnotpunish, cr_edu
_boysout, cr_ta_womanrole, cr_ta_eemandecid, cr_fi_girlchance, cr_fi_girlsavings, cr_sr
h_teasegirl, cr_srh_teaseboy,cr_vi_hit, cr_vi_privematt, cr_vi_tolerate))
```

```
#test reliability using Chronbachs Alpha for 20-item GAGE gender norms scale

alpha(data_ET20_subset_GNS_CFA[, c("cr_edu_boysch", "cr_edu_science", "cr_edu_girlnohel
p", "cr_edu_raisingvoice", "cr_edu_boysfeelings", "cr_edu_boystough", "cr_edu_boysdefen
d", "cr_edu_equaltreat", "cr_edu_teachpunish", "cr_edu_teachnotpunish", "cr_edu_boysou
t", "cr_ta_womanrole", "cr_ta_eemandecid", "cr_fi_girlchance", "cr_fi_girlsavings", "cr
_srh_teasegirl", "cr_srh_teaseboy","cr_vi_hit", "cr_vi_privematt", "cr_vi_tolerate")])
```

```
##
## Reliability analysis
## Call: alpha(x = data_ET20_subset_GNS_CFA[, c("cr_edu_boysch", "cr_edu_science",
##     "cr_edu_girlnohelp", "cr_edu_raisingvoice", "cr_edu_boysfeelings",
##     "cr_edu_boystough", "cr_edu_boysdefend", "cr_edu_equaltreat",
##     "cr_edu_teachpunish", "cr_edu_teachnotpunish", "cr_edu_boysout",
##     "cr_ta_womanrole", "cr_ta_eemandecid", "cr_fi_girlchance",
##     "cr_fi_girlsavings", "cr_srh_teasegirl", "cr_srh_teaseboy",
##     "cr_vi_hit", "cr_vi_privematt", "cr_vi_tolerate")])
##
##   raw_alpha std.alpha G6(smc) average_r S/N    ase mean   sd median_r
##       0.78      0.77    0.79      0.14 3.3 0.0045  2.1 0.36     0.14
##
##     95% confidence boundaries
##          lower alpha upper
## Feldt     0.77  0.78  0.79
## Duhachek  0.77  0.78  0.79
##
##  Reliability if an item is dropped:
##                       raw_alpha std.alpha G6(smc) average_r S/N alpha se var.r
## cr_edu_boysch              0.76      0.74    0.77      0.13 2.9   0.0049 0.015
## cr_edu_science             0.77      0.75    0.78      0.14 3.0   0.0048 0.015
## cr_edu_girlnohelp          0.76      0.75    0.77      0.13 2.9   0.0048 0.015
## cr_edu_raisingvoice        0.76      0.74    0.77      0.13 2.9   0.0049 0.015
## cr_edu_boysfeelings        0.79      0.78    0.80      0.15 3.5   0.0044 0.014
## cr_edu_boystough           0.78      0.77    0.79      0.15 3.3   0.0045 0.016
## cr_edu_boysdefend          0.77      0.76    0.79      0.14 3.2   0.0046 0.016
## cr_edu_equaltreat          0.78      0.76    0.79      0.14 3.2   0.0045 0.016
## cr_edu_teachpunish         0.77      0.76    0.78      0.14 3.2   0.0046 0.016
## cr_edu_teachnotpunish      0.78      0.77    0.79      0.15 3.3   0.0044 0.016
## cr_edu_boysout             0.77      0.75    0.78      0.14 3.1   0.0047 0.015
## cr_ta_womanrole            0.76      0.75    0.77      0.13 3.0   0.0049 0.015
## cr_ta_eemandecid           0.76      0.75    0.77      0.13 2.9   0.0049 0.015
## cr_fi_girlchance           0.78      0.77    0.79      0.15 3.3   0.0045 0.015
## cr_fi_girlsavings          0.78      0.78    0.80      0.15 3.4   0.0044 0.014
## cr_srh_teasegirl           0.77      0.76    0.78      0.14 3.1   0.0047 0.015
## cr_srh_teaseboy            0.77      0.75    0.78      0.14 3.1   0.0047 0.015
## cr_vi_hit                  0.76      0.75    0.77      0.13 3.0   0.0049 0.015
## cr_vi_privematt            0.77      0.75    0.78      0.14 3.0   0.0048 0.016
## cr_vi_tolerate             0.76      0.75    0.78      0.14 3.0   0.0049 0.015
##                       med.r
## cr_edu_boysch          0.14
## cr_edu_science         0.14
## cr_edu_girlnohelp      0.13
## cr_edu_raisingvoice    0.13
## cr_edu_boysfeelings    0.15
## cr_edu_boystough       0.15
## cr_edu_boysdefend      0.14
## cr_edu_equaltreat      0.14
## cr_edu_teachpunish     0.14
## cr_edu_teachnotpunish  0.15
## cr_edu_boysout         0.14
## cr_ta_womanrole        0.13
## cr_ta_eemandecid       0.13
## cr_fi_girlchance       0.15
```

```
## cr_fi_girlsavings      0.15
## cr_srh_teasegirl       0.14
## cr_srh_teaseboy        0.14
## cr_vi_hit              0.13
## cr_vi_privematt        0.14
## cr_vi_tolerate         0.13
##
##  Item statistics
##                         n raw.r std.r r.cor r.drop mean   sd
## cr_edu_boysch        4741  0.57  0.58 0.561  0.483  2.5 0.83
## cr_edu_science       4728  0.50  0.52 0.495  0.424  2.7 0.69
## cr_edu_girlnohelp    4743  0.55  0.56 0.543  0.464  2.5 0.81
## cr_edu_raisingvoice  4750  0.60  0.58 0.556  0.499  2.0 0.94
## cr_edu_boysfeelings  4739  0.14  0.15 0.057  0.046  1.4 0.67
## cr_edu_boystough     4743  0.30  0.31 0.231  0.202  1.5 0.73
## cr_edu_boysdefend    4740  0.38  0.38 0.312  0.275  1.6 0.84
## cr_edu_equaltreat    4744  0.36  0.35 0.282  0.251  1.6 0.80
## cr_edu_teachpunish   4715  0.40  0.39 0.330  0.295  1.5 0.80
## cr_edu_teachnotpunish 4717  0.29  0.28 0.198  0.172  1.7 0.86
## cr_edu_boysout       4748  0.46  0.47 0.428  0.366  2.6 0.76
## cr_ta_womanrole      4755  0.56  0.54 0.518  0.465  1.8 0.90
## cr_ta_eemandecid     4754  0.57  0.55 0.526  0.477  1.8 0.90
## cr_fi_girlchance     4728  0.23  0.26 0.181  0.138  2.7 0.67
## cr_fi_girlsavings    4708  0.11  0.17 0.079  0.047  2.8 0.48
## cr_srh_teasegirl     4704  0.44  0.45 0.420  0.343  2.5 0.80
## cr_srh_teaseboy      4708  0.45  0.46 0.435  0.359  2.5 0.79
## cr_vi_hit            4729  0.57  0.54 0.517  0.467  2.1 0.93
## cr_vi_privematt      4728  0.51  0.50 0.456  0.410  2.2 0.93
## cr_vi_tolerate       4729  0.55  0.53 0.495  0.449  1.9 0.92
##
## Non missing response frequency for each item
##                         1    2    3 miss
## cr_edu_boysch        0.22 0.10 0.68 0.01
## cr_edu_science       0.13 0.07 0.80 0.01
## cr_edu_girlnohelp    0.20 0.09 0.71 0.01
## cr_edu_raisingvoice  0.44 0.12 0.45 0.01
## cr_edu_boysfeelings  0.76 0.13 0.11 0.01
## cr_edu_boystough     0.69 0.17 0.15 0.01
## cr_edu_boysdefend    0.60 0.16 0.24 0.01
## cr_edu_equaltreat    0.65 0.16 0.20 0.01
## cr_edu_teachpunish   0.66 0.15 0.19 0.01
## cr_edu_teachnotpunish 0.60 0.15 0.26 0.01
## cr_edu_boysout       0.16 0.10 0.74 0.01
## cr_ta_womanrole      0.56 0.13 0.31 0.00
## cr_ta_eemandecid     0.54 0.14 0.32 0.00
## cr_fi_girlchance     0.11 0.10 0.79 0.01
## cr_fi_girlsavings    0.05 0.08 0.87 0.01
## cr_srh_teasegirl     0.20 0.12 0.68 0.02
## cr_srh_teaseboy      0.19 0.12 0.69 0.01
## cr_vi_hit            0.38 0.11 0.51 0.01
## cr_vi_privematt      0.36 0.11 0.52 0.01
## cr_vi_tolerate       0.46 0.14 0.40 0.01
```

```
#alpha = 0.78
```

```
#test reliability using Chronbachs Alpha for 12-item GAGE gender norms scale (result fr
om EFA & CFA statistical analysis chapter 5)

alpha(data_ET20_subset_GNS_CFA[, c("cr_edu_boysch", "cr_edu_science", "cr_edu_girlnohel
p", "cr_edu_raisingvoice", "cr_edu_boysout", "cr_ta_womanrole", "cr_ta_eemandecid", "cr
_srh_teasegirl", "cr_srh_teaseboy", "cr_vi_hit", "cr_vi_privematt", "cr_vi_tolerate")])
```

```
##
## Reliability analysis
## Call: alpha(x = data_ET20_subset_GNS_CFA[, c("cr_edu_boysch", "cr_edu_science",
##     "cr_edu_girlnohelp", "cr_edu_raisingvoice", "cr_edu_boysout",
##     "cr_ta_womanrole", "cr_ta_eemandecid", "cr_srh_teasegirl",
##     "cr_srh_teaseboy", "cr_vi_hit", "cr_vi_privematt", "cr_vi_tolerate")])
##
##   raw_alpha std.alpha G6(smc) average_r S/N    ase mean   sd median_r
##       0.81      0.81    0.82      0.26 4.2 0.0041  2.2 0.48     0.24
##
##     95% confidence boundaries
##          lower alpha upper
## Feldt      0.8  0.81  0.81
## Duhachek   0.8  0.81  0.82
##
##  Reliability if an item is dropped:
##                     raw_alpha std.alpha G6(smc) average_r S/N alpha se  var.r
## cr_edu_boysch            0.79      0.79    0.79      0.25 3.7   0.0045 0.0078
## cr_edu_science           0.79      0.79    0.80      0.26 3.8   0.0044 0.0079
## cr_edu_girlnohelp        0.79      0.79    0.80      0.25 3.7   0.0045 0.0079
## cr_edu_raisingvoice      0.79      0.79    0.80      0.26 3.8   0.0045 0.0084
## cr_edu_boysout           0.80      0.80    0.81      0.26 4.0   0.0043 0.0081
## cr_ta_womanrole          0.79      0.79    0.80      0.26 3.9   0.0045 0.0078
## cr_ta_eemandecid         0.79      0.79    0.80      0.26 3.9   0.0045 0.0079
## cr_srh_teasegirl         0.80      0.80    0.80      0.27 4.0   0.0043 0.0061
## cr_srh_teaseboy          0.80      0.80    0.80      0.27 4.0   0.0043 0.0065
## cr_vi_hit                0.79      0.79    0.80      0.26 3.8   0.0045 0.0084
## cr_vi_privematt          0.80      0.80    0.80      0.26 3.9   0.0044 0.0085
## cr_vi_tolerate           0.79      0.80    0.80      0.26 3.9   0.0044 0.0080
##                     med.r
## cr_edu_boysch        0.24
## cr_edu_science       0.24
## cr_edu_girlnohelp    0.23
## cr_edu_raisingvoice  0.24
## cr_edu_boysout       0.24
## cr_ta_womanrole      0.24
## cr_ta_eemandecid     0.24
## cr_srh_teasegirl     0.24
## cr_srh_teaseboy      0.25
## cr_vi_hit            0.24
## cr_vi_privematt      0.24
## cr_vi_tolerate       0.24
##
##  Item statistics
##                        n raw.r std.r r.cor r.drop mean   sd
## cr_edu_boysch       4741  0.62  0.63  0.59   0.52  2.5 0.83
## cr_edu_science      4728  0.56  0.58  0.53   0.47  2.7 0.69
## cr_edu_girlnohelp   4743  0.61  0.63  0.58   0.51  2.5 0.81
## cr_edu_raisingvoice 4750  0.61  0.59  0.53   0.49  2.0 0.94
## cr_edu_boysout      4748  0.51  0.53  0.47   0.41  2.6 0.76
## cr_ta_womanrole     4755  0.58  0.57  0.51   0.46  1.8 0.90
## cr_ta_eemandecid    4754  0.58  0.57  0.51   0.46  1.8 0.90
## cr_srh_teasegirl    4704  0.49  0.51  0.46   0.38  2.5 0.80
## cr_srh_teaseboy     4708  0.51  0.52  0.47   0.39  2.5 0.79
## cr_vi_hit           4729  0.60  0.58  0.53   0.48  2.1 0.93
```

```
## cr_vi_privematt     4728  0.55  0.54  0.47   0.43  2.2 0.93
## cr_vi_tolerate      4729  0.58  0.56  0.50   0.45  1.9 0.92
##
## Non missing response frequency for each item
##                        1    2    3 miss
## cr_edu_boysch       0.22 0.10 0.68 0.01
## cr_edu_science      0.13 0.07 0.80 0.01
## cr_edu_girlnohelp   0.20 0.09 0.71 0.01
## cr_edu_raisingvoice 0.44 0.12 0.45 0.01
## cr_edu_boysout      0.16 0.10 0.74 0.01
## cr_ta_womanrole     0.56 0.13 0.31 0.00
## cr_ta_eemandecid    0.54 0.14 0.32 0.00
## cr_srh_teasegirl    0.20 0.12 0.68 0.02
## cr_srh_teaseboy     0.19 0.12 0.69 0.01
## cr_vi_hit           0.38 0.11 0.51 0.01
## cr_vi_privematt     0.36 0.11 0.52 0.01
## cr_vi_tolerate      0.46 0.14 0.40 0.01
```

```
#alpha = 0.81
```

```
#test reliability using Chronbachs alpha for 7-factor GAGE gender norms scale (per fact
or) (result from EFA & CFA statistical analysis chapter 5)

f1 <- alpha(data_ET20_subset_GNS_CFA[, c("cr_edu_boysch", "cr_edu_science", "cr_edu_gir
lnohelp", "cr_edu_boysout")])
f1  #alpha = 0.72
```

```
##
## Reliability analysis
## Call: alpha(x = data_ET20_subset_GNS_CFA[, c("cr_edu_boysch", "cr_edu_science",
##     "cr_edu_girlnohelp", "cr_edu_boysout")])
##
##   raw_alpha std.alpha G6(smc) average_r S/N    ase mean   sd median_r
##       0.72      0.72    0.66      0.39 2.6 0.0066  2.6 0.57     0.39
##
##     95% confidence boundaries
##          lower alpha upper
## Feldt     0.71  0.72  0.73
## Duhachek  0.71  0.72  0.73
##
##  Reliability if an item is dropped:
##                   raw_alpha std.alpha G6(smc) average_r S/N alpha se   var.r
## cr_edu_boysch          0.64      0.65    0.55      0.38 1.8   0.0089 0.00137
## cr_edu_science         0.65      0.65    0.56      0.38 1.9   0.0087 0.00233
## cr_edu_girlnohelp      0.64      0.65    0.55      0.38 1.8   0.0089 0.00287
## cr_edu_boysout         0.69      0.70    0.60      0.43 2.3   0.0076 0.00012
##                   med.r
## cr_edu_boysch      0.37
## cr_edu_science     0.37
## cr_edu_girlnohelp  0.35
## cr_edu_boysout     0.44
##
##  Item statistics
##                      n raw.r std.r r.cor r.drop mean   sd
## cr_edu_boysch     4741  0.77  0.75  0.63   0.53  2.5 0.83
## cr_edu_science    4728  0.73  0.75  0.62   0.52  2.7 0.69
## cr_edu_girlnohelp 4743  0.76  0.75  0.63   0.53  2.5 0.81
## cr_edu_boysout    4748  0.70  0.70  0.53   0.45  2.6 0.76
##
## Non missing response frequency for each item
##                      1    2    3 miss
## cr_edu_boysch     0.22 0.10 0.68 0.01
## cr_edu_science    0.13 0.07 0.80 0.01
## cr_edu_girlnohelp 0.20 0.09 0.71 0.01
## cr_edu_boysout    0.16 0.10 0.74 0.01
```

```
f2 <- alpha(data_ET20_subset_GNS_CFA[, c("cr_vi_hit", "cr_vi_privematt", "cr_vi_tolerat
e")])
f2   #alpha=0.64
```

```
##
## Reliability analysis
## Call: alpha(x = data_ET20_subset_GNS_CFA[, c("cr_vi_hit", "cr_vi_privematt",
##     "cr_vi_tolerate")])
##
##   raw_alpha std.alpha G6(smc) average_r S/N    ase mean   sd median_r
##       0.64      0.64    0.54      0.37 1.8 0.0091  2.1 0.71     0.38
##
##     95% confidence boundaries
##          lower alpha upper
## Feldt     0.62  0.64  0.66
## Duhachek  0.62  0.64  0.66
##
##  Reliability if an item is dropped:
##                 raw_alpha std.alpha G6(smc) average_r S/N alpha se var.r med.r
## cr_vi_hit            0.52      0.52    0.35      0.35 1.1    0.014    NA  0.35
## cr_vi_privematt      0.56      0.56    0.39      0.39 1.3    0.013    NA  0.39
## cr_vi_tolerate       0.55      0.55    0.38      0.38 1.2    0.013    NA  0.38
##
##  Item statistics
##                    n raw.r std.r r.cor r.drop mean   sd
## cr_vi_hit       4729  0.77  0.77  0.58   0.46  2.1 0.93
## cr_vi_privematt 4728  0.75  0.75  0.54   0.44  2.2 0.93
## cr_vi_tolerate  4729  0.76  0.76  0.55   0.44  1.9 0.92
##
## Non missing response frequency for each item
##                    1    2    3 miss
## cr_vi_hit       0.38 0.11 0.51 0.01
## cr_vi_privematt 0.36 0.11 0.52 0.01
## cr_vi_tolerate  0.46 0.14 0.40 0.01
```

```
f3 <- alpha(data_ET20_subset_GNS_CFA[, c("cr_srh_teasegirl", "cr_srh_teaseboy")])
f3  #alpha=0.73
```

```
##
## Reliability analysis
## Call: alpha(x = data_ET20_subset_GNS_CFA[, c("cr_srh_teasegirl", "cr_srh_teasebo
y")])
##
##   raw_alpha std.alpha G6(smc) average_r S/N    ase mean   sd median_r
##       0.73      0.73    0.58      0.58 2.7 0.0077  2.5 0.71     0.58
##
##     95% confidence boundaries
##          lower alpha upper
## Feldt     0.72  0.73  0.75
## Duhachek  0.72  0.73  0.75
##
##  Reliability if an item is dropped:
##                  raw_alpha std.alpha G6(smc) average_r S/N alpha se var.r med.r
## cr_srh_teasegirl      0.59      0.58    0.33      0.58 1.4       NA     0  0.58
## cr_srh_teaseboy       0.57      0.58    0.33      0.58 1.4       NA     0  0.58
##
##  Item statistics
##                     n raw.r std.r r.cor r.drop mean   sd
## cr_srh_teasegirl 4704  0.89  0.89  0.68   0.58  2.5 0.80
## cr_srh_teaseboy  4708  0.89  0.89  0.68   0.58  2.5 0.79
##
## Non missing response frequency for each item
##                     1    2    3 miss
## cr_srh_teasegirl 0.20 0.12 0.68 0.02
## cr_srh_teaseboy  0.19 0.12 0.69 0.01
```

```
f4 <- alpha(data_ET20_subset_GNS_CFA[, c("cr_ta_womanrole", "cr_ta_eemandecid","cr_edu_
raisingvoice")])
f4  #alpha=0.65
```

```
##
## Reliability analysis
## Call: alpha(x = data_ET20_subset_GNS_CFA[, c("cr_ta_womanrole", "cr_ta_eemandecid",
##     "cr_edu_raisingvoice")])
##
##   raw_alpha std.alpha G6(smc) average_r S/N    ase mean  sd median_r
##       0.65      0.65    0.56      0.39 1.9 0.0087  1.8 0.7     0.39
##
##     95% confidence boundaries
##          lower alpha upper
## Feldt     0.64  0.65  0.67
## Duhachek  0.64  0.65  0.67
##
##  Reliability if an item is dropped:
##                     raw_alpha std.alpha G6(smc) average_r S/N alpha se var.r
## cr_ta_womanrole          0.54      0.54    0.37      0.37 1.2    0.013    NA
## cr_ta_eemandecid         0.57      0.57    0.40      0.40 1.3    0.012    NA
## cr_edu_raisingvoice      0.56      0.56    0.39      0.39 1.3    0.013    NA
##                     med.r
## cr_ta_womanrole      0.37
## cr_ta_eemandecid     0.40
## cr_edu_raisingvoice  0.39
##
##  Item statistics
##                        n raw.r std.r r.cor r.drop mean   sd
## cr_ta_womanrole     4755  0.77  0.78  0.59   0.48  1.8 0.90
## cr_ta_eemandecid    4754  0.76  0.76  0.56   0.45  1.8 0.90
## cr_edu_raisingvoice 4750  0.77  0.77  0.57   0.46  2.0 0.94
##
## Non missing response frequency for each item
##                        1    2    3 miss
## cr_ta_womanrole     0.56 0.13 0.31 0.00
## cr_ta_eemandecid    0.54 0.14 0.32 0.00
## cr_edu_raisingvoice 0.44 0.12 0.45 0.01
```

```
f5 <- alpha(data_ET20_subset_GNS_CFA[, c("cr_edu_boysfeelings", "cr_edu_boystough", "cr
_edu_boysdefend", "cr_edu_equaltreat")])
f5  #alpha=0.53
```

```
##
## Reliability analysis
## Call: alpha(x = data_ET20_subset_GNS_CFA[, c("cr_edu_boysfeelings",
##     "cr_edu_boystough", "cr_edu_boysdefend", "cr_edu_equaltreat")])
##
##   raw_alpha std.alpha G6(smc) average_r S/N   ase mean   sd median_r
##       0.53      0.53    0.46      0.22 1.1 0.011  1.5 0.49     0.22
##
##     95% confidence boundaries
##          lower alpha upper
## Feldt     0.51  0.53  0.55
## Duhachek  0.51  0.53  0.55
##
##  Reliability if an item is dropped:
##                     raw_alpha std.alpha G6(smc) average_r  S/N alpha se   var.r
## cr_edu_boysfeelings      0.46      0.47    0.37      0.23 0.87    0.013 3.3e-04
## cr_edu_boystough         0.44      0.44    0.35      0.21 0.80    0.014 2.1e-04
## cr_edu_boysdefend        0.46      0.46    0.36      0.22 0.85    0.013 5.3e-04
## cr_edu_equaltreat        0.46      0.47    0.37      0.23 0.87    0.013 4.8e-06
##                     med.r
## cr_edu_boysfeelings  0.23
## cr_edu_boystough     0.21
## cr_edu_boysdefend    0.22
## cr_edu_equaltreat    0.23
##
##  Item statistics
##                        n raw.r std.r r.cor r.drop mean   sd
## cr_edu_boysfeelings 4739  0.60  0.64  0.42   0.31  1.4 0.67
## cr_edu_boystough    4743  0.65  0.66  0.46   0.34  1.5 0.73
## cr_edu_boysdefend   4740  0.67  0.64  0.44   0.32  1.6 0.84
## cr_edu_equaltreat   4744  0.65  0.64  0.43   0.31  1.6 0.80
##
## Non missing response frequency for each item
##                        1    2    3 miss
## cr_edu_boysfeelings 0.76 0.13 0.11 0.01
## cr_edu_boystough    0.69 0.17 0.15 0.01
## cr_edu_boysdefend   0.60 0.16 0.24 0.01
## cr_edu_equaltreat   0.65 0.16 0.20 0.01
```

```
f6 <- alpha(data_ET20_subset_GNS_CFA[, c("cr_edu_teachpunish", "cr_edu_teachnotpunis
h")])
f6  #alpha=0.45
```

```
##
## Reliability analysis
## Call: alpha(x = data_ET20_subset_GNS_CFA[, c("cr_edu_teachpunish",
##     "cr_edu_teachnotpunish")])
##
##   raw_alpha std.alpha G6(smc) average_r  S/N   ase mean   sd median_r
##       0.45      0.45    0.29      0.29 0.81 0.016  1.6 0.66     0.29
##
##     95% confidence boundaries
##          lower alpha upper
## Feldt     0.41  0.45  0.48
## Duhachek  0.41  0.45  0.48
##
##  Reliability if an item is dropped:
##                       raw_alpha std.alpha G6(smc) average_r S/N alpha se var.r
## cr_edu_teachpunish         0.27      0.29   0.083      0.29 0.4       NA     0
## cr_edu_teachnotpunish      0.31      0.29   0.083      0.29 0.4       NA     0
##                       med.r
## cr_edu_teachpunish     0.29
## cr_edu_teachnotpunish  0.29
##
##  Item statistics
##                          n raw.r std.r r.cor r.drop mean   sd
## cr_edu_teachpunish    4715  0.79   0.8  0.43   0.29  1.5 0.80
## cr_edu_teachnotpunish 4717  0.82   0.8  0.43   0.29  1.7 0.86
##
## Non missing response frequency for each item
##                          1    2    3 miss
## cr_edu_teachpunish    0.66 0.15 0.19 0.01
## cr_edu_teachnotpunish 0.60 0.15 0.26 0.01
```

```
f7 <- alpha(data_ET20_subset_GNS_CFA[, c("cr_fi_girlchance", "cr_fi_girlsavings")])
f7 #alpha=0.44
```

```
##
## Reliability analysis
## Call: alpha(x = data_ET20_subset_GNS_CFA[, c("cr_fi_girlchance", "cr_fi_girlsaving
s")])
##
##   raw_alpha std.alpha G6(smc) average_r  S/N   ase mean   sd median_r
##       0.44      0.46     0.3       0.3 0.86 0.015  2.7 0.47      0.3
##
##     95% confidence boundaries
##          lower alpha upper
## Feldt     0.41  0.44  0.47
## Duhachek  0.41  0.44  0.47
##
##  Reliability if an item is dropped:
##                     raw_alpha std.alpha G6(smc) average_r  S/N alpha se var.r
## cr_fi_girlchance       0.41       0.3    0.09       0.3 0.43       NA     0
## cr_fi_girlsavings      0.22       0.3    0.09       0.3 0.43       NA     0
##                     med.r
## cr_fi_girlchance    0.3
## cr_fi_girlsavings   0.3
##
##  Item statistics
##                        n raw.r std.r r.cor r.drop mean   sd
## cr_fi_girlchance  4728  0.87  0.81  0.44    0.3  2.7 0.67
## cr_fi_girlsavings 4708  0.73  0.81  0.44    0.3  2.8 0.48
##
## Non missing response frequency for each item
##                        1    2    3 miss
## cr_fi_girlchance  0.11 0.10 0.79 0.01
## cr_fi_girlsavings 0.05 0.08 0.87 0.01
```

```
#test reliability for gender roles/cr_genderedactivities
splitHalf(data_CRET20_rec[, c("cr_ta_careother", "cr_ta_chores")])
```

```
## Split half reliabilities
## Call: splitHalf(r = data_CRET20_rec[, c("cr_ta_careother", "cr_ta_chores")])
##
## Maximum split half reliability (lambda 4) =  0.51
## Guttman lambda 6                          =  0.35
## Average split half reliability            =  0.51
## Guttman lambda 3 (alpha)                  =  0.51
## Guttman lambda 2                          =  0.51
## Minimum split half reliability  (beta)    =  0.51
## Average interitem r =  0.35  with median =  0.35
```

```
#reliability=0.51
```

```
#test reliability using Cronbach's Alpha for cr_talk
alpha(data_CRET20_rec[, c("cr_talk_ed", "cr_talk_bullying", "cr_talk_religion", "cr_tal
k_work")])
```

```
##
## Reliability analysis
## Call: alpha(x = data_CRET20_rec[, c("cr_talk_ed", "cr_talk_bullying",
##     "cr_talk_religion", "cr_talk_work")])
##
##   raw_alpha std.alpha G6(smc) average_r S/N    ase mean   sd median_r
##       0.64      0.64    0.59      0.31 1.8 0.0086 0.63 0.31     0.29
##
##     95% confidence boundaries
##          lower alpha upper
## Feldt     0.62  0.64  0.65
## Duhachek  0.62  0.64  0.65
##
##  Reliability if an item is dropped:
##                  raw_alpha std.alpha G6(smc) average_r S/N alpha se  var.r
## cr_talk_ed            0.53      0.53    0.43      0.27 1.1   0.0119 0.0029
## cr_talk_bullying      0.65      0.65    0.56      0.38 1.9   0.0089 0.0059
## cr_talk_religion      0.58      0.59    0.51      0.32 1.4   0.0106 0.0167
## cr_talk_work          0.52      0.53    0.43      0.27 1.1   0.0121 0.0052
##                  med.r
## cr_talk_ed        0.26
## cr_talk_bullying  0.35
## cr_talk_religion  0.26
## cr_talk_work      0.24
##
##  Item statistics
##                     n raw.r std.r r.cor r.drop mean   sd
## cr_talk_ed       1934  0.71  0.74  0.63   0.50 0.81 0.39
## cr_talk_bullying 1928  0.63  0.62  0.38   0.31 0.35 0.48
## cr_talk_religion 1935  0.69  0.68  0.50   0.40 0.70 0.46
## cr_talk_work     1936  0.75  0.74  0.62   0.48 0.65 0.48
##
## Non missing response frequency for each item
##                     0    1 miss
## cr_talk_ed       0.19 0.81 0.60
## cr_talk_bullying 0.65 0.35 0.60
## cr_talk_religion 0.30 0.70 0.59
## cr_talk_work     0.35 0.65 0.59
```

```
#alpha=0.64
```

```
#test reliability using Cronbach's Alpha for cr_voice
alpha(data_CRET20_rec[, c("cr_mva_timehwork", "cr_mva_contschool", "cr_mva_marrywhen",
"cr_mva_marrywho", "cr_mva_friends", "cr_mva_freetime", "cr_mva_leavehouse", "cr_mva_ea
rnact")])
```

```
##
## Reliability analysis
## Call: alpha(x = data_CRET20_rec[, c("cr_mva_timehwork", "cr_mva_contschool",
##     "cr_mva_marrywhen", "cr_mva_marrywho", "cr_mva_friends",
##     "cr_mva_freetime", "cr_mva_leavehouse", "cr_mva_earnact")])
##
##   raw_alpha std.alpha G6(smc) average_r S/N   ase mean   sd median_r
##       0.77      0.77    0.78      0.29 3.3 0.005  2.4 0.71     0.26
##
##     95% confidence boundaries
##          lower alpha upper
## Feldt     0.76  0.77  0.78
## Duhachek  0.76  0.77  0.78
##
##  Reliability if an item is dropped:
##                   raw_alpha std.alpha G6(smc) average_r S/N alpha se var.r
## cr_mva_timehwork       0.78      0.78    0.79      0.34 3.5   0.0050 0.019
## cr_mva_contschool      0.77      0.76    0.78      0.32 3.3   0.0050 0.023
## cr_mva_marrywhen       0.73      0.73    0.72      0.28 2.7   0.0057 0.013
## cr_mva_marrywho        0.74      0.73    0.72      0.28 2.7   0.0057 0.012
## cr_mva_friends         0.73      0.72    0.74      0.27 2.6   0.0060 0.022
## cr_mva_freetime        0.73      0.73    0.74      0.28 2.7   0.0059 0.026
## cr_mva_leavehouse      0.74      0.74    0.76      0.29 2.8   0.0057 0.026
## cr_mva_earnact         0.74      0.73    0.75      0.28 2.8   0.0058 0.026
##                   med.r
## cr_mva_timehwork   0.31
## cr_mva_contschool  0.29
## cr_mva_marrywhen   0.26
## cr_mva_marrywho    0.26
## cr_mva_friends     0.26
## cr_mva_freetime    0.26
## cr_mva_leavehouse  0.26
## cr_mva_earnact     0.26
##
##  Item statistics
##                      n raw.r std.r r.cor r.drop mean   sd
## cr_mva_timehwork  4509  0.39  0.42  0.26   0.24  3.2 0.88
## cr_mva_contschool 4451  0.52  0.50  0.38   0.33  2.9 1.21
## cr_mva_marrywhen  4291  0.66  0.68  0.67   0.53  1.7 1.06
## cr_mva_marrywho   4275  0.65  0.67  0.66   0.52  1.5 0.98
## cr_mva_friends    4408  0.71  0.70  0.64   0.57  2.1 1.20
## cr_mva_freetime   4483  0.69  0.68  0.62   0.56  2.7 1.15
## cr_mva_leavehouse 4399  0.64  0.63  0.54   0.49  2.4 1.18
## cr_mva_earnact    4425  0.67  0.65  0.57   0.51  2.3 1.24
##
## Non missing response frequency for each item
##                      1    2    3    4 miss
## cr_mva_timehwork  0.06 0.12 0.34 0.48 0.06
## cr_mva_contschool 0.23 0.09 0.20 0.48 0.07
## cr_mva_marrywhen  0.67 0.09 0.13 0.11 0.10
## cr_mva_marrywho   0.73 0.08 0.11 0.08 0.11
## cr_mva_friends    0.50 0.10 0.23 0.18 0.08
## cr_mva_freetime   0.24 0.12 0.32 0.33 0.06
## cr_mva_leavehouse 0.37 0.11 0.31 0.21 0.08
## cr_mva_earnact    0.40 0.11 0.23 0.25 0.07
```

```
#test reliability using Cronbach's Alpha for resilience scale
alpha(data_CRET20_rec[, c("cr_ps_resrolemodels_rec", "cr_ps_resedu_rec", "cr_ps_respare
ntsknow_rec", "cr_ps_resfinish_rec", "cr_ps_resnothurt_rec", "cr_ps_reshelp_rec", "cr_p
s_resbelongschool_rec", "cr_ps_resfamilycare_rec", "cr_ps_resfriendscare_rec", "cr_ps_r
esfair_rec", "cr_ps_reslearn_rec", "cr_ps_rescommunity_rec")])
```

```
##
## Reliability analysis
## Call: alpha(x = data_CRET20_rec[, c("cr_ps_resrolemodels_rec", "cr_ps_resedu_rec",
##     "cr_ps_resparentsknow_rec", "cr_ps_resfinish_rec", "cr_ps_resnothurt_rec",
##     "cr_ps_reshelp_rec", "cr_ps_resbelongschool_rec", "cr_ps_resfamilycare_rec",
##     "cr_ps_resfriendscare_rec", "cr_ps_resfair_rec", "cr_ps_reslearn_rec",
##     "cr_ps_rescommunity_rec")])
##
##   raw_alpha std.alpha G6(smc) average_r S/N   ase mean   sd median_r
##       0.76      0.78    0.79      0.23 3.6 0.005  1.6 0.36     0.24
##
##     95% confidence boundaries
##          lower alpha upper
## Feldt     0.75  0.76  0.77
## Duhachek  0.76  0.76  0.77
##
##  Reliability if an item is dropped:
##                           raw_alpha std.alpha G6(smc) average_r S/N alpha se
## cr_ps_resrolemodels_rec        0.78      0.79    0.79      0.25 3.7   0.0046
## cr_ps_resedu_rec               0.75      0.77    0.77      0.24 3.4   0.0052
## cr_ps_resparentsknow_rec       0.75      0.77    0.77      0.23 3.3   0.0053
## cr_ps_resfinish_rec            0.74      0.76    0.76      0.22 3.1   0.0055
## cr_ps_resnothurt_rec           0.74      0.77    0.77      0.23 3.3   0.0054
## cr_ps_reshelp_rec              0.75      0.77    0.77      0.23 3.3   0.0054
## cr_ps_resbelongschool_rec      0.74      0.76    0.76      0.23 3.2   0.0055
## cr_ps_resfamilycare_rec        0.75      0.77    0.77      0.23 3.3   0.0053
## cr_ps_resfriendscare_rec       0.75      0.77    0.77      0.23 3.3   0.0054
## cr_ps_resfair_rec              0.74      0.77    0.77      0.23 3.3   0.0054
## cr_ps_reslearn_rec             0.74      0.76    0.76      0.22 3.2   0.0056
## cr_ps_rescommunity_rec         0.75      0.77    0.77      0.23 3.4   0.0053
##                            var.r med.r
## cr_ps_resrolemodels_rec   0.0058  0.25
## cr_ps_resedu_rec          0.0064  0.25
## cr_ps_resparentsknow_rec  0.0079  0.24
## cr_ps_resfinish_rec       0.0076  0.22
## cr_ps_resnothurt_rec      0.0068  0.24
## cr_ps_reshelp_rec         0.0072  0.24
## cr_ps_resbelongschool_rec 0.0074  0.22
## cr_ps_resfamilycare_rec   0.0081  0.22
## cr_ps_resfriendscare_rec  0.0081  0.22
## cr_ps_resfair_rec         0.0078  0.24
## cr_ps_reslearn_rec        0.0074  0.22
## cr_ps_rescommunity_rec    0.0078  0.24
##
##  Item statistics
##                              n raw.r std.r r.cor r.drop mean   sd
## cr_ps_resrolemodels_rec   1938  0.42  0.36  0.25   0.22  1.3 0.92
## cr_ps_resedu_rec          1924  0.48  0.51  0.45   0.37  1.8 0.53
## cr_ps_resparentsknow_rec  1938  0.49  0.54  0.47   0.39  1.8 0.51
## cr_ps_resfinish_rec       1927  0.59  0.63  0.59   0.51  1.8 0.50
## cr_ps_resnothurt_rec      1927  0.57  0.55  0.50   0.43  1.5 0.76
## cr_ps_reshelp_rec         1911  0.60  0.54  0.49   0.44  1.4 0.87
## cr_ps_resbelongschool_rec 1920  0.60  0.59  0.54   0.46  1.6 0.75
## cr_ps_resfamilycare_rec   1941  0.51  0.56  0.50   0.43  1.9 0.46
## cr_ps_resfriendscare_rec  1926  0.56  0.55  0.48   0.43  1.5 0.73
```

```
## cr_ps_resfair_rec         1917  0.56  0.56  0.50   0.44  1.7 0.66
## cr_ps_reslearn_rec        1917  0.63  0.62  0.57   0.51  1.6 0.70
## cr_ps_rescommunity_rec    1941  0.48  0.52  0.45   0.38  1.8 0.49
##
## Non missing response frequency for each item
##                              0    1    2 miss
## cr_ps_resrolemodels_rec   0.32 0.06 0.62 0.59
## cr_ps_resedu_rec          0.07 0.03 0.90 0.60
## cr_ps_resparentsknow_rec  0.06 0.06 0.88 0.59
## cr_ps_resfinish_rec       0.04 0.11 0.85 0.60
## cr_ps_resnothurt_rec      0.17 0.16 0.67 0.60
## cr_ps_reshelp_rec         0.27 0.11 0.62 0.60
## cr_ps_resbelongschool_rec 0.16 0.06 0.78 0.60
## cr_ps_resfamilycare_rec   0.05 0.05 0.90 0.59
## cr_ps_resfriendscare_rec  0.14 0.17 0.68 0.60
## cr_ps_resfair_rec         0.11 0.13 0.77 0.60
## cr_ps_reslearn_rec        0.13 0.14 0.73 0.60
## cr_ps_rescommunity_rec    0.05 0.08 0.87 0.59
```

```
#alpha=0.76
```

```
#test reliability using Cronbach's Alpha for peervio ordinal
alpha(data_CRET20_rec[, c("cr_vi_numk_1_rec1", "cr_vi_numk_2_rec1", "cr_vi_numk_3_rec
1", "cr_vi_numk_4_rec1", "cr_vi_numk_5_rec1", "cr_vi_numk_6_rec1")])
```

```
##
## Reliability analysis
## Call: alpha(x = data_CRET20_rec[, c("cr_vi_numk_1_rec1", "cr_vi_numk_2_rec1",
##     "cr_vi_numk_3_rec1", "cr_vi_numk_4_rec1", "cr_vi_numk_5_rec1",
##     "cr_vi_numk_6_rec1")])
##
##   raw_alpha std.alpha G6(smc) average_r S/N    ase mean   sd median_r
##       0.69       0.7    0.67      0.28 2.4 0.0066 0.12 0.26     0.26
##
##     95% confidence boundaries
##          lower alpha upper
## Feldt     0.68  0.69   0.7
## Duhachek  0.68  0.69   0.7
##
##  Reliability if an item is dropped:
##                   raw_alpha std.alpha G6(smc) average_r S/N alpha se  var.r
## cr_vi_numk_1_rec1      0.62      0.64    0.59      0.27 1.8   0.0083 0.0010
## cr_vi_numk_2_rec1      0.64      0.66    0.61      0.28 1.9   0.0077 0.0017
## cr_vi_numk_3_rec1      0.65      0.67    0.62      0.28 2.0   0.0075 0.0019
## cr_vi_numk_4_rec1      0.65      0.67    0.62      0.29 2.0   0.0074 0.0025
## cr_vi_numk_5_rec1      0.66      0.67    0.62      0.29 2.0   0.0074 0.0025
## cr_vi_numk_6_rec1      0.67      0.68    0.63      0.30 2.1   0.0072 0.0020
##                   med.r
## cr_vi_numk_1_rec1  0.25
## cr_vi_numk_2_rec1  0.26
## cr_vi_numk_3_rec1  0.28
## cr_vi_numk_4_rec1  0.28
## cr_vi_numk_5_rec1  0.27
## cr_vi_numk_6_rec1  0.30
##
##  Item statistics
##                      n raw.r std.r r.cor r.drop  mean   sd
## cr_vi_numk_1_rec1 4036  0.75  0.68  0.60   0.50 0.212 0.56
## cr_vi_numk_2_rec1 4036  0.64  0.64  0.53   0.46 0.102 0.40
## cr_vi_numk_3_rec1 4036  0.67  0.63  0.51   0.44 0.169 0.49
## cr_vi_numk_4_rec1 4036  0.63  0.62  0.50   0.41 0.119 0.43
## cr_vi_numk_5_rec1 4035  0.55  0.63  0.51   0.41 0.048 0.27
## cr_vi_numk_6_rec1 4036  0.53  0.60  0.47   0.38 0.059 0.29
##
## Non missing response frequency for each item
##                      0    1    2 miss
## cr_vi_numk_1_rec1 0.86 0.06 0.07 0.16
## cr_vi_numk_2_rec1 0.93 0.04 0.03 0.16
## cr_vi_numk_3_rec1 0.88 0.07 0.05 0.16
## cr_vi_numk_4_rec1 0.92 0.04 0.04 0.16
## cr_vi_numk_5_rec1 0.96 0.02 0.01 0.16
## cr_vi_numk_6_rec1 0.96 0.03 0.01 0.16
```

```
#alpha=0.69
```

5 Statistical Analyses

5.1 Exploratory Factor Analysis (EFA) for GAGE gender norm scale

```
#EFA
remove_low_communality = function(data, fa_result, threshold) {

  communalities = fa_result$communality
  low_comm_vars <- names(communalities[communalities < threshold])
  print(low_comm_vars)

  data_new = data %>%
    dplyr::select(-all_of(low_comm_vars))

  return(data_new)
}

remove_low_loadings = function(data, fa_result, threshold) {

  loadings = fa_result$loadings
  high_loading_vars <- apply(loadings, 1, function(x) any(abs(x) >= threshold))

  data_new = data[, high_loading_vars]

  return(data_new)
}

factors_table = function(fa_result) {
  fa_result$loadings[] %>%
  datatable(options = list(pageLength = 50)) %>%
  formatRound(colnames(fa_result$loadings[]), digits=2) %>%
  formatStyle(colnames(fa_result$loadings[]), backgroundColor = styleInterval(c(0.3, 0.
5), c("white", "yellow", "green")))
}

extract_loadings <- function(fa_object, threshold = 0.3) {
  loadings_df <- as.data.frame(unclass(FA_ET_pa_v1$loadings))

  loading_variables = list()

  for (i in 1:ncol(loadings_df)) {
    factor = colnames(loadings_df)[i]
    variables = loadings_df %>%
      filter(abs(.[[i]]) > threshold) %>%
      rownames()
    print(factor)
    print(variables)
  }}
```

```
#subset for GAGE gender norms scale
data_ET20_subset_gendernormsscale = data_ET20_final %>%
  dplyr::select(c(cr_edu_culture, cr_edu_boysch, cr_edu_science, cr_edu_girlnohelp, cr_
edu_raisingvoice, cr_edu_boysfeelings, cr_edu_boystough, cr_edu_boysdefend, cr_edu_girl
sprotect, cr_edu_girlshumble, cr_edu_boysweak, cr_edu_equaltreat, cr_edu_teachpunish, c
r_edu_teachnotpunish, cr_edu_boysout, cr_ta_eesharetasks, cr_ta_womanrole, cr_ta_eemand
ecid, cr_fi_girlchance, cr_fi_goodwife, cr_fi_girlsavings, cr_srh_waitedu, cr_srh_contr
oldaugt, cr_srh_boyfinal, cr_srh_teasegirl, cr_srh_teaseboy, cr_srh_genderroles, cr_srh
_aware, cr_srh_changereaction, cr_vi_hit, cr_vi_privematt, cr_vi_tolerate))

data_ET20_GNS <- na.omit(data_ET20_subset_gendernormsscale)
```

5.1.1 Factor solution parallel analysis

```
#Bartletts test of sphericity
cor_data_ET20_GNS <- cor(data_ET20_GNS)
bartlett_data_ET20_GNS <- cortest.bartlett(cor_data_ET20_GNS, n = nrow(data_ET20_GNS))
print(bartlett_data_ET20_GNS)
```

```
## $chisq
## [1] 22272.41853
##
## $p.value
## [1] 0
##
## $df
## [1] 496
```

```
fa.parallel(data_ET20_subset_gendernormsscale, fm = "pa", fa = "fa", quant = 0.95, sho
w.legend = F, main = "Parallel Analysis Plots (pa)", sqrt = T)
```

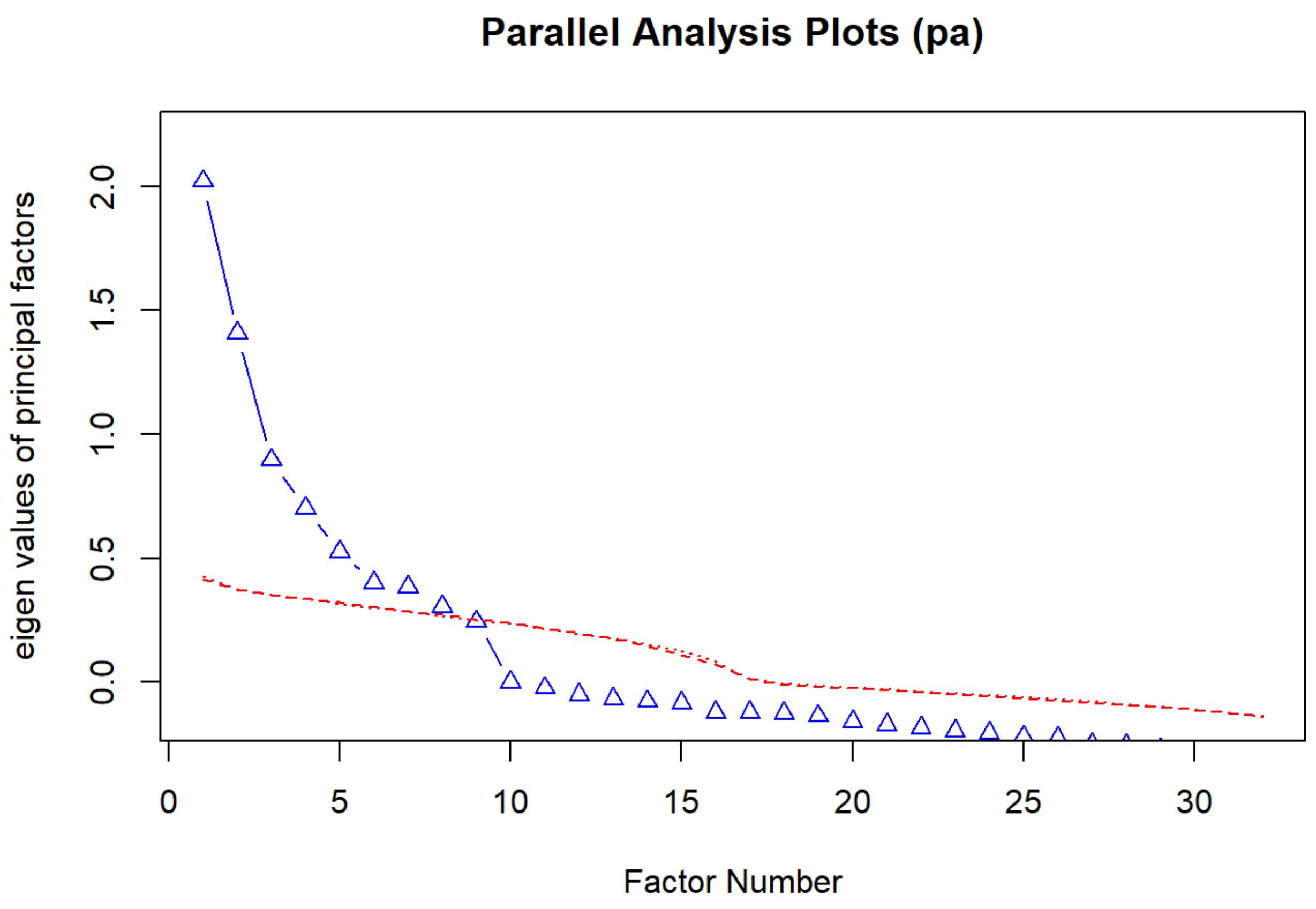


```
## Parallel analysis suggests that the number of factors =  8  and the number of compon
ents =  NA
```

```
fa.parallel(data_ET20_subset_gendernormsscale, fm = "minres", fa = "fa", quant = 0.95,
show.legend = F, main = "Parallel Analysis Plots (minres)", sqrt = T)
```

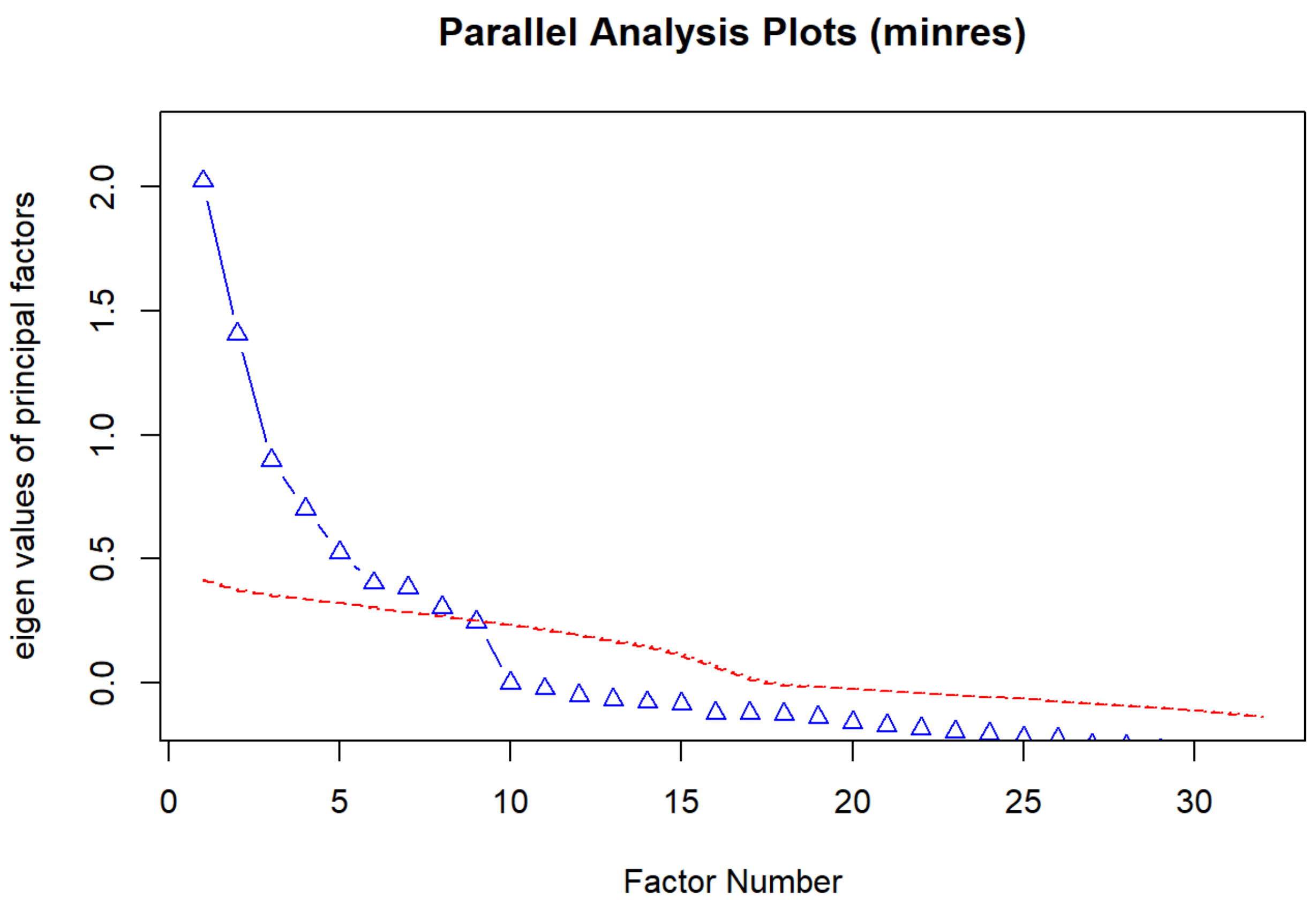


```
## Parallel analysis suggests that the number of factors =  8  and the number of compon
ents =  NA
```

```
#8 factors
```

```
FA_ET20_pa = fa(data_ET20_subset_gendernormsscale, nfactors = 8, fm = "pa", rotate = "o
blimin")

print(FA_ET20_pa)
```

```
## Factor Analysis using method =  pa
## Call: fa(r = data_ET20_subset_gendernormsscale, nfactors = 8, rotate = "oblimin",
##     fm = "pa")
## Standardized loadings (pattern matrix) based upon correlation matrix
##                          PA4   PA3   PA1   PA8   PA2   PA5   PA6   PA7   h2   u2
## cr_edu_culture          0.31 -0.02  0.01 -0.16  0.16  0.08 -0.01  0.12 0.15 0.85
## cr_edu_boysch           0.60 -0.05  0.08  0.05  0.02 -0.02  0.05  0.01 0.44 0.56
## cr_edu_science          0.64  0.01 -0.06  0.06  0.00 -0.01 -0.02 -0.01 0.41 0.59
## cr_edu_girlnohelp       0.59  0.04  0.08  0.00 -0.02  0.02  0.06 -0.04 0.45 0.55
## cr_edu_raisingvoice     0.12  0.07  0.46  0.04  0.04 -0.01  0.07  0.07 0.40 0.60
## cr_edu_boysfeelings    -0.01 -0.02 -0.15  0.02  0.50  0.04 -0.07  0.02 0.29 0.71
## cr_edu_boystough        0.03 -0.01  0.08 -0.02  0.49  0.02  0.02 -0.03 0.25 0.75
## cr_edu_boysdefend      -0.01  0.06  0.00  0.13  0.40  0.01  0.03  0.08 0.23 0.77
## cr_edu_girlsprotect     0.09 -0.06  0.11 -0.08  0.26  0.05 -0.04  0.10 0.15 0.85
## cr_edu_girlshumble     -0.05 -0.01  0.08 -0.01  0.31  0.00 -0.10  0.09 0.18 0.82
## cr_edu_boysweak         0.29  0.15  0.12 -0.09  0.12  0.00 -0.07  0.01 0.18 0.82
## cr_edu_equaltreat      -0.03  0.03  0.11  0.07  0.37  0.01  0.02  0.03 0.21 0.79
## cr_edu_teachpunish      0.00  0.00  0.15  0.01  0.04  0.01  0.02  0.43 0.26 0.74
## cr_edu_teachnotpunish -0.02  0.00 -0.05  0.00 -0.01 -0.01 -0.01  0.61 0.36 0.64
## cr_edu_boysout          0.53  0.07 -0.04  0.00 -0.03  0.03  0.00  0.03 0.31 0.69
## cr_ta_eesharetasks      0.02 -0.04  0.08  0.11 -0.01 -0.04  0.36 -0.04 0.20 0.80
## cr_ta_womanrole         0.06  0.02  0.55  0.09 -0.01 -0.04  0.02  0.00 0.41 0.59
## cr_ta_eemandecid        0.05  0.01  0.41  0.18  0.04  0.04  0.05  0.07 0.36 0.64
## cr_fi_girlchance       -0.01  0.00  0.04 -0.02  0.02  0.01  0.63 -0.02 0.39 0.61
## cr_fi_goodwife          0.26  0.15  0.00  0.19 -0.12  0.05  0.02  0.00 0.25 0.75
## cr_fi_girlsavings       0.10  0.04 -0.13 -0.02 -0.11 -0.05  0.42  0.04 0.27 0.73
## cr_srh_waitedu          0.06  0.05 -0.15  0.00  0.04 -0.22  0.19  0.00 0.12 0.88
## cr_srh_controldaugt    -0.04  0.04  0.31  0.04  0.07  0.19 -0.02  0.06 0.22 0.78
## cr_srh_boyfinal        -0.03  0.09  0.10 -0.15  0.15  0.20 -0.14 -0.01 0.15 0.85
## cr_srh_teasegirl        0.00  0.76 -0.02  0.02  0.01 -0.02  0.00  0.00 0.57 0.43
## cr_srh_teaseboy         0.00  0.76  0.03 -0.02 -0.01  0.02  0.00  0.00 0.59 0.41
## cr_srh_genderroles     -0.01 -0.01 -0.02  0.01  0.09  0.43  0.00  0.00 0.22 0.78
## cr_srh_aware           -0.06  0.05 -0.01  0.08  0.04  0.49  0.05 -0.02 0.27 0.73
## cr_srh_changereaction -0.10  0.02  0.04  0.01  0.07 -0.49  0.05 -0.04 0.25 0.75
## cr_vi_hit               0.01  0.04  0.05  0.57  0.03  0.04  0.04  0.03 0.41 0.59
## cr_vi_privematt         0.08  0.03 -0.05  0.55  0.04  0.01 -0.01  0.00 0.35 0.65
## cr_vi_tolerate          0.04 -0.02  0.19  0.48 -0.01  0.03 -0.06  0.02 0.38 0.62
##                        com
## cr_edu_culture         2.6
## cr_edu_boysch          1.1
## cr_edu_science         1.0
## cr_edu_girlnohelp      1.1
## cr_edu_raisingvoice    1.3
## cr_edu_boysfeelings    1.2
## cr_edu_boystough       1.1
## cr_edu_boysdefend      1.3
## cr_edu_girlsprotect    2.7
## cr_edu_girlshumble     1.6
## cr_edu_boysweak        2.8
## cr_edu_equaltreat      1.3
## cr_edu_teachpunish     1.2
## cr_edu_teachnotpunish 1.0
## cr_edu_boysout         1.1
## cr_ta_eesharetasks     1.4
## cr_ta_womanrole        1.1
```

```
## cr_ta_eemandecid      1.6
## cr_fi_girlchance      1.0
## cr_fi_goodwife        3.1
## cr_fi_girlsavings     1.6
## cr_srh_waitedu        3.2
## cr_srh_controldaugt   2.0
## cr_srh_boyfinal       4.8
## cr_srh_teasegirl      1.0
## cr_srh_teaseboy       1.0
## cr_srh_genderroles    1.1
## cr_srh_aware          1.1
## cr_srh_changereaction 1.2
## cr_vi_hit             1.1
## cr_vi_privematt       1.1
## cr_vi_tolerate        1.4
##
##                        PA4  PA3  PA1  PA8  PA2  PA5  PA6  PA7
## SS loadings           1.94 1.34 1.29 1.26 1.24 0.94 0.92 0.74
## Proportion Var        0.06 0.04 0.04 0.04 0.04 0.03 0.03 0.02
## Cumulative Var        0.06 0.10 0.14 0.18 0.22 0.25 0.28 0.30
## Proportion Explained  0.20 0.14 0.13 0.13 0.13 0.10 0.09 0.08
## Cumulative Proportion 0.20 0.34 0.47 0.60 0.73 0.83 0.92 1.00
##
##  With factor correlations of
##      PA4  PA3  PA1  PA8   PA2   PA5   PA6   PA7
## PA4 1.00 0.47 0.40 0.47  0.01  0.11  0.36  0.17
## PA3 0.47 1.00 0.19 0.31  0.02  0.24  0.12  0.13
## PA1 0.40 0.19 1.00 0.52  0.28  0.12  0.19  0.26
## PA8 0.47 0.31 0.52 1.00  0.12  0.21  0.17  0.20
## PA2 0.01 0.02 0.28 0.12  1.00  0.39 -0.27  0.41
## PA5 0.11 0.24 0.12 0.21  0.39  1.00 -0.31  0.23
## PA6 0.36 0.12 0.19 0.17 -0.27 -0.31  1.00 -0.14
## PA7 0.17 0.13 0.26 0.20  0.41  0.23 -0.14  1.00
##
## Mean item complexity =  1.6
## Test of the hypothesis that 8 factors are sufficient.
##
## df null model =  496  with the objective function =  5.07 with Chi Square =  24141.5
7
## df of  the model are 268  and the objective function was  0.14
##
## The root mean square of the residuals (RMSR) is  0.01
## The df corrected root mean square of the residuals is  0.02
##
## The harmonic n.obs is  4707 with the empirical chi square  733.03  with prob <  7.4e
-45
## The total n.obs was  4777  with Likelihood Chi Square =  681  with prob <  8.6e-38
##
## Tucker Lewis Index of factoring reliability =  0.968
## RMSEA index =  0.018  and the 90 % confidence intervals are  0.016 0.02
## BIC =  -1589.38
## Fit based upon off diagonal values = 0.99
## Measures of factor score adequacy
##                                                    PA4  PA3  PA1  PA8  PA2  PA5
## Correlation of (regression) scores with factors   0.88 0.87 0.82 0.83 0.80 0.76
## Multiple R square of scores with factors          0.78 0.76 0.68 0.69 0.64 0.58
```

```
## Minimum correlation of possible factor scores     0.55 0.52 0.36 0.38 0.29 0.17
##                                                    PA6  PA7
## Correlation of (regression) scores with factors   0.78 0.74
## Multiple R square of scores with factors          0.61 0.54
## Minimum correlation of possible factor scores     0.21 0.08
```

```
factors_table(FA_ET20_pa)
```

Show 50 entries Search:

	PA4	PA3	PA1	PA8	PA2	PA5	PA6	PA7
cr_edu_culture	0.31	-0.02	0.01	-0.16	0.16	0.08	-0.01	0.12
cr_edu_boysch	0.60	-0.05	0.08	0.05	0.02	-0.02	0.05	0.01
cr_edu_science	0.64	0.01	-0.06	0.06	-0.00	-0.01	-0.02	-0.01
cr_edu_girlnohelp	0.59	0.04	0.08	-0.00	-0.02	0.02	0.06	-0.04
cr_edu_raisingvoice	0.12	0.07	0.46	0.04	0.04	-0.01	0.07	0.07
cr_edu_boysfeelings	-0.01	-0.02	-0.15	0.02	0.50	0.04	-0.07	0.02
cr_edu_boystough	0.03	-0.01	0.08	-0.02	0.49	0.02	0.02	-0.03
cr_edu_boysdefend	-0.01	0.06	-0.00	0.13	0.40	0.01	0.03	0.08
cr_edu_girlsprotect	0.09	-0.06	0.11	-0.08	0.26	0.05	-0.04	0.10
cr_edu_girlshumble	-0.05	-0.01	0.08	-0.01	0.31	0.00	-0.10	0.09
cr_edu_boysweak	0.29	0.15	0.12	-0.09	0.12	0.00	-0.07	0.01
cr_edu_equaltreat	-0.03	0.03	0.11	0.07	0.37	0.01	0.02	0.03
cr_edu_teachpunish	-0.00	-0.00	0.15	0.01	0.04	0.01	0.02	0.43
cr_edu_teachnotpunish	-0.02	0.00	-0.05	0.00	-0.01	-0.01	-0.01	0.61
cr_edu_boysout	0.53	0.07	-0.04	-0.00	-0.03	0.03	-0.00	0.03
cr_ta_eesharetasks	0.02	-0.04	0.08	0.11	-0.01	-0.04	0.36	-0.04
cr_ta_womanrole	0.06	0.02	0.55	0.09	-0.01	-0.04	0.02	0.00
cr_ta_eemandecid	0.05	0.01	0.41	0.18	0.04	0.04	0.05	0.07
cr_fi_girlchance	-0.01	-0.00	0.04	-0.02	0.02	0.01	0.63	-0.02
cr_fi_goodwife	0.26	0.15	-0.00	0.19	-0.12	0.05	0.02	-0.00
cr_fi_girlsavings	0.10	0.04	-0.13	-0.02	-0.11	-0.05	0.42	0.04

	PA4	PA3	PA1	PA8	PA2	PA5	PA6	PA7
cr_srh_waitedu	0.06	0.05	-0.15	-0.00	0.04	-0.22	0.19	-0.00
cr_srh_controldaugt	-0.04	0.04	0.31	0.04	0.07	0.19	-0.02	0.06
cr_srh_boyfinal	-0.03	0.09	0.10	-0.15	0.15	0.20	-0.14	-0.01
cr_srh_teasegirl	0.00	0.76	-0.02	0.02	0.01	-0.02	-0.00	-0.00
cr_srh_teaseboy	-0.00	0.76	0.03	-0.02	-0.01	0.02	0.00	0.00
cr_srh_genderroles	-0.01	-0.01	-0.02	0.01	0.09	0.43	-0.00	-0.00
cr_srh_aware	-0.06	0.05	-0.01	0.08	0.04	0.49	0.05	-0.02
cr_srh_changereaction	-0.10	0.02	0.04	0.01	0.07	-0.49	0.05	-0.04
cr_vi_hit	0.01	0.04	0.05	0.57	0.03	0.04	0.04	0.03
cr_vi_privematt	0.08	0.03	-0.05	0.55	0.04	0.01	-0.01	0.00
cr_vi_tolerate	0.04	-0.02	0.19	0.48	-0.01	0.03	-0.06	0.02

Showing 1 to 32 of 32 entries

Previous 1 Next

```
data_ET20_pa = data_ET20_subset_gendernormsscale %>% dplyr::select(-c(cr_srh_waitedu, cr_srh_changereaction, cr_fi_goodwife, cr_edu_girlsprotect, cr_edu_boysweak, cr_srh_boyfinal))
```

```
KMO(data_ET20_pa)
```

```
## Kaiser-Meyer-Olkin factor adequacy
## Call: KMO(r = data_ET20_pa)
## Overall MSA =  0.86
## MSA for each item =
##        cr_edu_culture          cr_edu_boysch         cr_edu_science
##                  0.87                   0.89                   0.89
##     cr_edu_girlnohelp   cr_edu_raisingvoice    cr_edu_boysfeelings
##                  0.90                   0.92                   0.79
##      cr_edu_boystough      cr_edu_boysdefend     cr_edu_girlshumble
##                  0.84                   0.88                   0.85
##     cr_edu_equaltreat     cr_edu_teachpunish cr_edu_teachnotpunish
##                  0.88                   0.85                   0.78
##        cr_edu_boysout     cr_ta_eesharetasks        cr_ta_womanrole
##                  0.90                   0.83                   0.89
##      cr_ta_eemandecid      cr_fi_girlchance      cr_fi_girlsavings
##                  0.91                   0.76                   0.78
##   cr_srh_controldaugt      cr_srh_teasegirl        cr_srh_teaseboy
##                  0.92                   0.73                   0.74
##    cr_srh_genderroles          cr_srh_aware              cr_vi_hit
##                  0.79                   0.81                   0.90
##       cr_vi_privematt        cr_vi_tolerate
##                  0.89                   0.90
```

```
fa.parallel(data_ET20_pa, fm = "pa", fa = "fa", quant = 0.95, show.legend = F, main =
"Parallel Analysis Plots (pa)", sqrt = T)
```

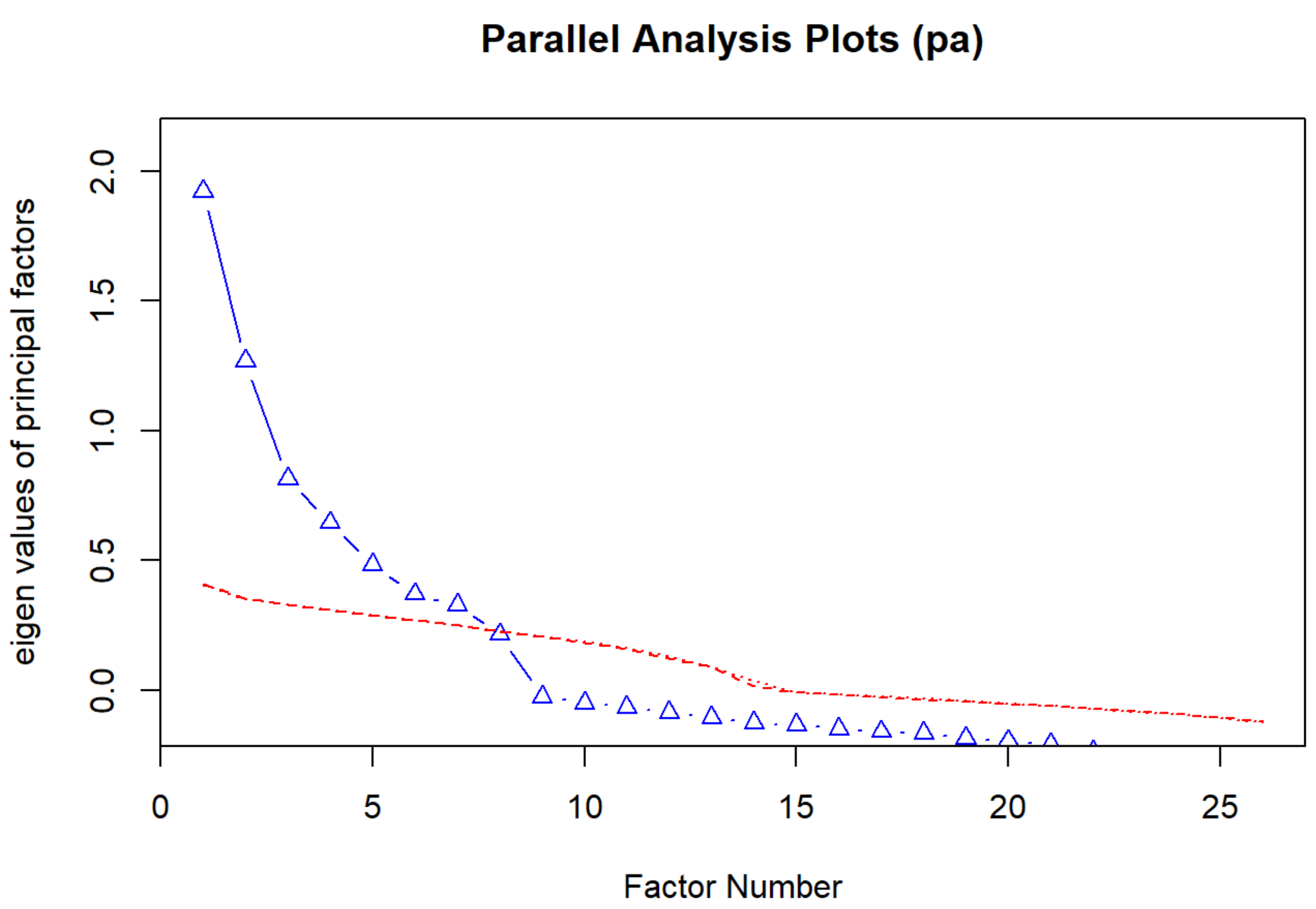


```
## Parallel analysis suggests that the number of factors =  7  and the number of compon
ents =  NA
```

```
fa.parallel(data_ET20_pa, fm = "minres", fa = "fa", quant = 0.95, show.legend = F, main
= "Parallel Analysis Plots (minres)", sqrt = T)
```

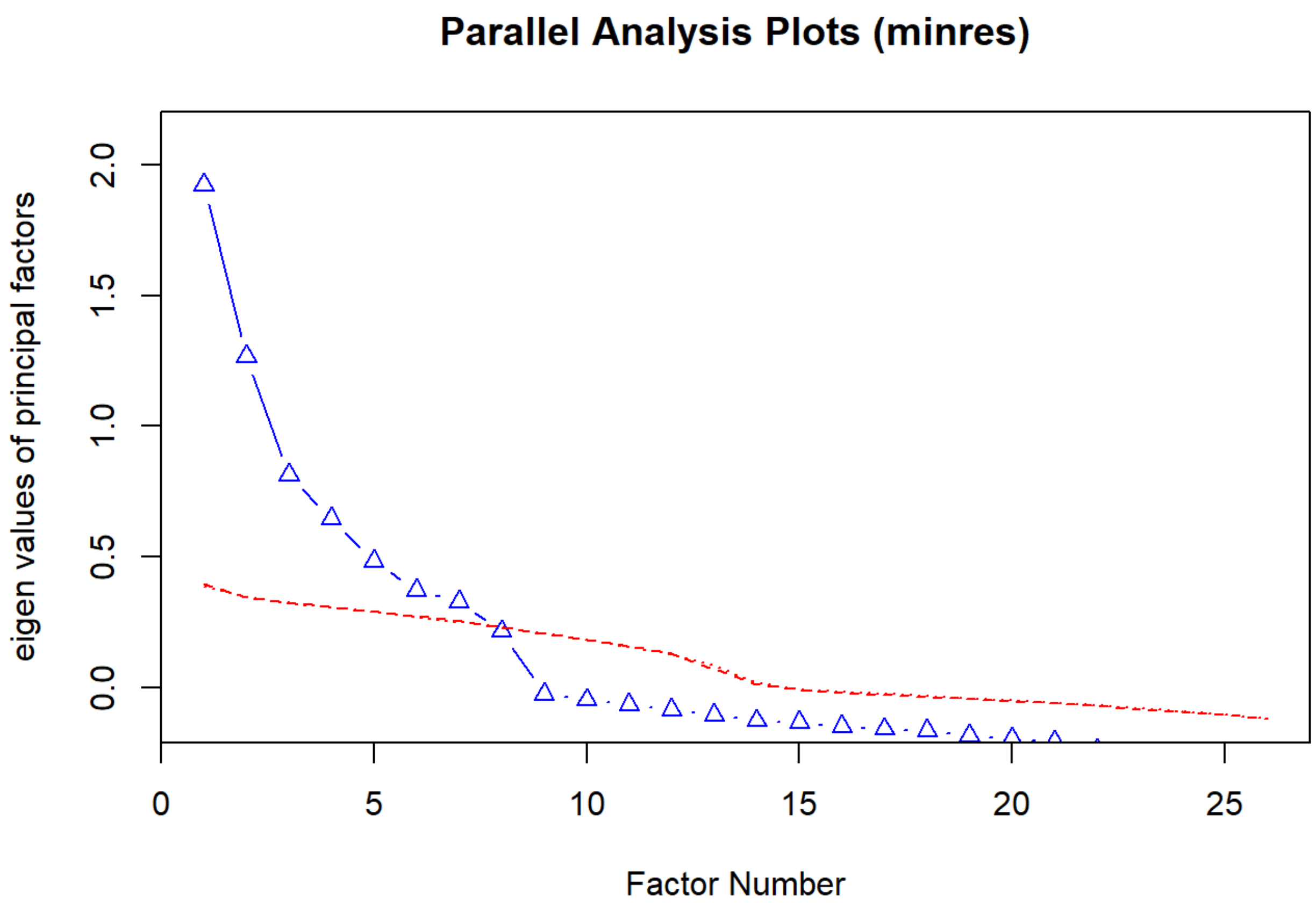


```
## Parallel analysis suggests that the number of factors =  7  and the number of compon
ents =  NA
```

```
#7 factors
```

```
FA_ET20_pa1 = fa(data_ET20_pa, nfactors = 7, fm = "pa", rotate = "oblimin")

print(FA_ET20_pa1)
```

```
## Factor Analysis using method =  pa
## Call: fa(r = data_ET20_pa, nfactors = 7, rotate = "oblimin", fm = "pa")
## Standardized loadings (pattern matrix) based upon correlation matrix
##                        PA4   PA7   PA2   PA3   PA1   PA5   PA6   h2   u2 com
## cr_edu_culture         0.29 -0.09  0.18  0.00 -0.02 -0.03  0.12 0.12 0.88 2.4
## cr_edu_boysch          0.63  0.04  0.01 -0.06  0.08  0.03  0.02 0.46 0.54 1.1
## cr_edu_science         0.66  0.02 -0.01  0.01 -0.05 -0.02  0.00 0.42 0.58 1.0
## cr_edu_girlnohelp      0.60  0.00  0.00  0.04  0.07  0.04 -0.04 0.44 0.56 1.1
## cr_edu_raisingvoice    0.11  0.05  0.05  0.08  0.46  0.06  0.07 0.39 0.61 1.3
## cr_edu_boysfeelings    0.00 -0.03  0.49 -0.04 -0.10 -0.06  0.05 0.27 0.73 1.2
## cr_edu_boystough       0.03 -0.05  0.50 -0.03  0.09  0.06  0.00 0.25 0.75 1.1
## cr_edu_boysdefend     -0.01  0.08  0.37  0.04  0.03  0.04  0.10 0.21 0.79 1.3
## cr_edu_girlshumble    -0.04 -0.06  0.32 -0.01  0.12 -0.10  0.10 0.18 0.82 1.8
## cr_edu_equaltreat     -0.03  0.04  0.37  0.01  0.13  0.03  0.05 0.21 0.79 1.3
## cr_edu_teachpunish     0.01  0.01  0.05  0.00  0.14  0.01  0.45 0.28 0.72 1.2
## cr_edu_teachnotpunish -0.01  0.02 -0.01  0.01 -0.06 -0.02  0.59 0.34 0.66 1.0
## cr_edu_boysout         0.54  0.00 -0.01  0.08 -0.04 -0.04  0.01 0.31 0.69 1.1
## cr_ta_eesharetasks     0.02  0.12 -0.05 -0.05  0.10  0.34 -0.04 0.19 0.81 1.6
## cr_ta_womanrole        0.06  0.05  0.00  0.03  0.60  0.00 -0.01 0.44 0.56 1.0
## cr_ta_eemandecid       0.05  0.20  0.06  0.01  0.40  0.00  0.04 0.37 0.63 1.6
## cr_fi_girlchance      -0.02  0.01  0.04  0.01  0.01  0.67 -0.02 0.43 0.57 1.0
## cr_fi_girlsavings      0.12 -0.03 -0.14  0.05 -0.13  0.40  0.03 0.26 0.74 1.7
## cr_srh_controldaugt   -0.04  0.16  0.17  0.08  0.20 -0.06  0.04 0.19 0.81 3.6
## cr_srh_teasegirl       0.01  0.00 -0.01  0.74 -0.01  0.00  0.00 0.56 0.44 1.0
## cr_srh_teaseboy       -0.01 -0.01  0.00  0.77  0.02  0.00  0.00 0.59 0.41 1.0
## cr_srh_genderroles     0.04  0.15  0.31  0.06 -0.13 -0.12 -0.05 0.16 0.84 2.4
## cr_srh_aware          -0.02  0.22  0.27  0.13 -0.11 -0.09 -0.08 0.16 0.84 3.3
## cr_vi_hit              0.00  0.60  0.00  0.03  0.04  0.03  0.03 0.42 0.58 1.0
## cr_vi_privematt        0.07  0.57  0.00  0.01 -0.06  0.02  0.01 0.34 0.66 1.1
## cr_vi_tolerate         0.03  0.49 -0.01 -0.03  0.17 -0.03  0.02 0.37 0.63 1.3
##
##                        PA4  PA7  PA2  PA3  PA1  PA5  PA6
## SS loadings           1.74 1.32 1.28 1.26 1.19 0.85 0.70
## Proportion Var        0.07 0.05 0.05 0.05 0.05 0.03 0.03
## Cumulative Var        0.07 0.12 0.17 0.22 0.26 0.29 0.32
## Proportion Explained  0.21 0.16 0.15 0.15 0.14 0.10 0.08
## Cumulative Proportion 0.21 0.37 0.52 0.67 0.81 0.92 1.00
##
##  With factor correlations of
##      PA4  PA7   PA2  PA3  PA1   PA5   PA6
## PA4 1.00 0.49  0.01 0.46 0.43  0.36  0.16
## PA7 0.49 1.00  0.23 0.34 0.55  0.10  0.23
## PA2 0.01 0.23  1.00 0.06 0.23 -0.29  0.40
## PA3 0.46 0.34  0.06 1.00 0.18  0.09  0.12
## PA1 0.43 0.55  0.23 0.18 1.00  0.23  0.27
## PA5 0.36 0.10 -0.29 0.09 0.23  1.00 -0.13
## PA6 0.16 0.23  0.40 0.12 0.27 -0.13  1.00
##
## Mean item complexity =  1.5
## Test of the hypothesis that 7 factors are sufficient.
##
## df null model =  325  with the objective function =  4.22 with Chi Square =  20112.6
7
## df of  the model are 164  and the objective function was  0.09
##
```

```
## The root mean square of the residuals (RMSR) is  0.01
## The df corrected root mean square of the residuals is  0.02
##
## The harmonic n.obs is  4712 with the empirical chi square  484.51  with prob <  2.1e
-33
## The total n.obs was  4777  with Likelihood Chi Square =  448.08  with prob <  3.2e-2
8
##
## Tucker Lewis Index of factoring reliability =  0.972
## RMSEA index =  0.019  and the 90 % confidence intervals are  0.017 0.021
## BIC =  -941.26
## Fit based upon off diagonal values = 0.99
## Measures of factor score adequacy
##                                                    PA4  PA7  PA2  PA3  PA1  PA5
## Correlation of (regression) scores with factors   0.88 0.84 0.80 0.87 0.83 0.77
## Multiple R square of scores with factors          0.77 0.70 0.64 0.75 0.68 0.60
## Minimum correlation of possible factor scores     0.54 0.41 0.28 0.50 0.36 0.20
##                                                    PA6
## Correlation of (regression) scores with factors   0.73
## Multiple R square of scores with factors          0.53
## Minimum correlation of possible factor scores     0.05
```

```
factors_table(FA_ET20_pa1)
```

Show 50 entries Search:

	PA4	PA7	PA2	PA3	PA1	PA5	PA6
cr_edu_culture	0.29	-0.09	0.18	-0.00	-0.02	-0.03	0.12
cr_edu_boysch	0.63	0.04	0.01	-0.06	0.08	0.03	0.02
cr_edu_science	0.66	0.02	-0.01	0.01	-0.05	-0.02	-0.00
cr_edu_girlnohelp	0.60	-0.00	0.00	0.04	0.07	0.04	-0.04
cr_edu_raisingvoice	0.11	0.05	0.05	0.08	0.46	0.06	0.07
cr_edu_boysfeelings	-0.00	-0.03	0.49	-0.04	-0.10	-0.06	0.05
cr_edu_boystough	0.03	-0.05	0.50	-0.03	0.09	0.06	0.00
cr_edu_boysdefend	-0.01	0.08	0.37	0.04	0.03	0.04	0.10
cr_edu_girlshumble	-0.04	-0.06	0.32	-0.01	0.12	-0.10	0.10
cr_edu_equaltreat	-0.03	0.04	0.37	0.01	0.13	0.03	0.05
cr_edu_teachpunish	0.01	0.01	0.05	0.00	0.14	0.01	0.45
cr_edu_teachnotpunish	-0.01	0.02	-0.01	0.01	-0.06	-0.02	0.59
cr_edu_boysout	0.54	-0.00	-0.01	0.08	-0.04	-0.04	0.01

	PA4	PA7	PA2	PA3	PA1	PA5	PA6
cr_ta_eesharetasks	0.02	0.12	-0.05	-0.05	0.10	0.34	-0.04
cr_ta_womanrole	0.06	0.05	-0.00	0.03	0.60	0.00	-0.01
cr_ta_eemandecid	0.05	0.20	0.06	0.01	0.40	-0.00	0.04
cr_fi_girlchance	-0.02	0.01	0.04	0.01	0.01	0.67	-0.02
cr_fi_girlsavings	0.12	-0.03	-0.14	0.05	-0.13	0.40	0.03
cr_srh_controldaugt	-0.04	0.16	0.17	0.08	0.20	-0.06	0.04
cr_srh_teasegirl	0.01	0.00	-0.01	0.74	-0.01	0.00	-0.00
cr_srh_teaseboy	-0.01	-0.01	-0.00	0.77	0.02	0.00	0.00
cr_srh_genderroles	0.04	0.15	0.31	0.06	-0.13	-0.12	-0.05
cr_srh_aware	-0.02	0.22	0.27	0.13	-0.11	-0.09	-0.08
cr_vi_hit	0.00	0.60	-0.00	0.03	0.04	0.03	0.03
cr_vi_privematt	0.07	0.57	-0.00	0.01	-0.06	0.02	0.01
cr_vi_tolerate	0.03	0.49	-0.01	-0.03	0.17	-0.03	0.02

Showing 1 to 26 of 26 entries

Previous 1 Next

#exclude variables again

```
data_ET20_pa1 = data_ET20_pa %>% dplyr::select(-c(cr_srh_aware, cr_srh_controldaugt, cr
_edu_culture))
```

```
KMO(data_ET20_pa1)
```

```
## Kaiser-Meyer-Olkin factor adequacy
## Call: KMO(r = data_ET20_pa1)
## Overall MSA =  0.85
## MSA for each item =
##          cr_edu_boysch         cr_edu_science     cr_edu_girlnohelp
##                   0.89                   0.88                  0.90
##    cr_edu_raisingvoice   cr_edu_boysfeelings      cr_edu_boystough
##                   0.91                   0.79                  0.82
##      cr_edu_boysdefend    cr_edu_girlshumble     cr_edu_equaltreat
##                   0.87                   0.83                  0.87
##     cr_edu_teachpunish cr_edu_teachnotpunish        cr_edu_boysout
##                   0.83                   0.76                  0.90
##     cr_ta_eesharetasks       cr_ta_womanrole      cr_ta_eemandecid
##                   0.83                   0.89                  0.90
##       cr_fi_girlchance      cr_fi_girlsavings     cr_srh_teasegirl
##                   0.76                   0.77                  0.72
##        cr_srh_teaseboy    cr_srh_genderroles             cr_vi_hit
##                   0.73                   0.83                  0.89
##        cr_vi_privematt        cr_vi_tolerate
##                   0.89                   0.89
```

```
fa.parallel(data_ET20_pa1, fm = "pa", fa = "fa", quant = 0.95, show.legend = F, main =
"Parallel Analysis Plots (pa)", sqrt = T)
```

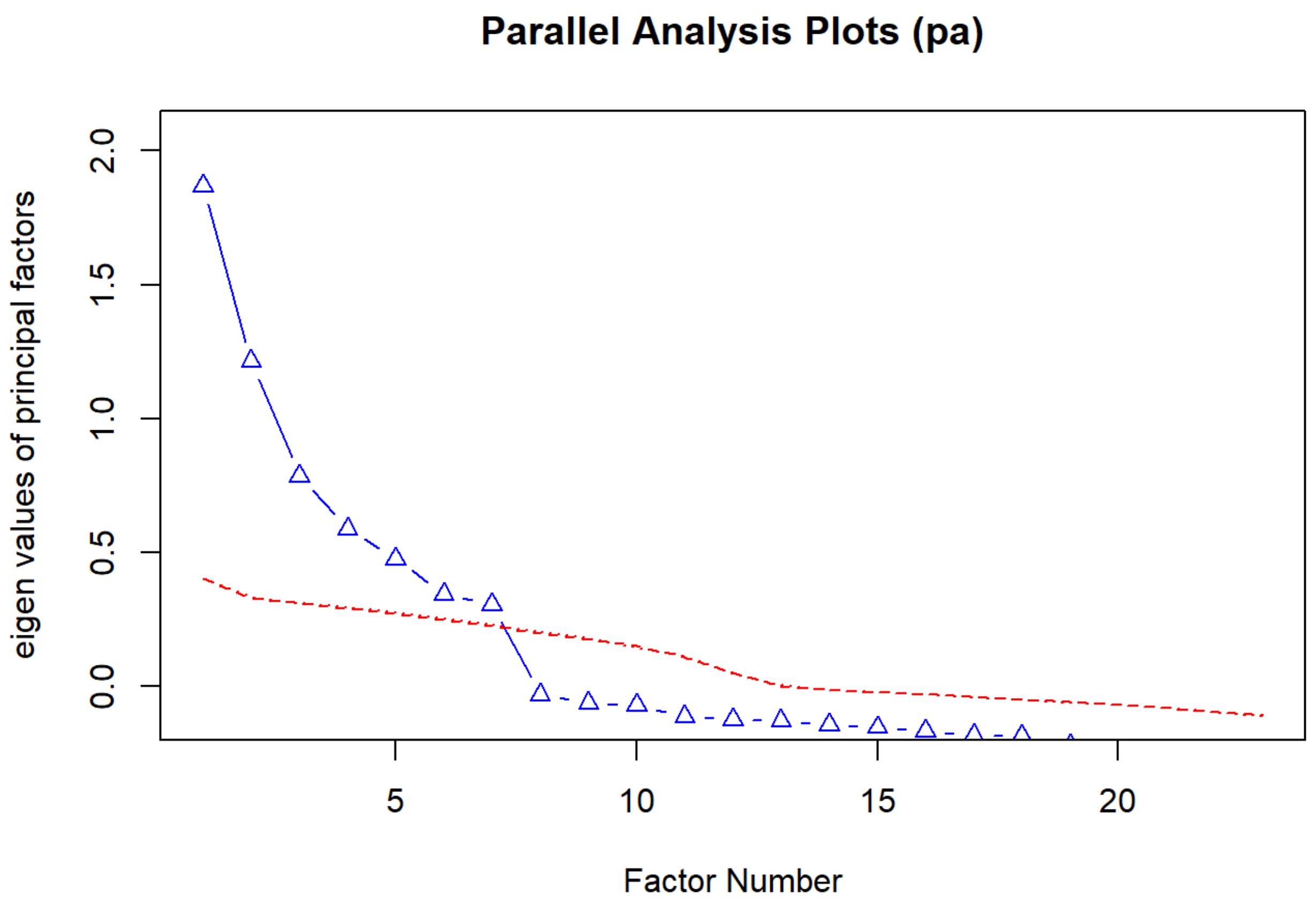


```
## Parallel analysis suggests that the number of factors =  7  and the number of compon
ents =  NA
```

```
fa.parallel(data_ET20_pa1, fm = "minres", fa = "fa", quant = 0.95, show.legend = F, main = "Parallel Analysis Plots (minres)", sqrt = T)
```

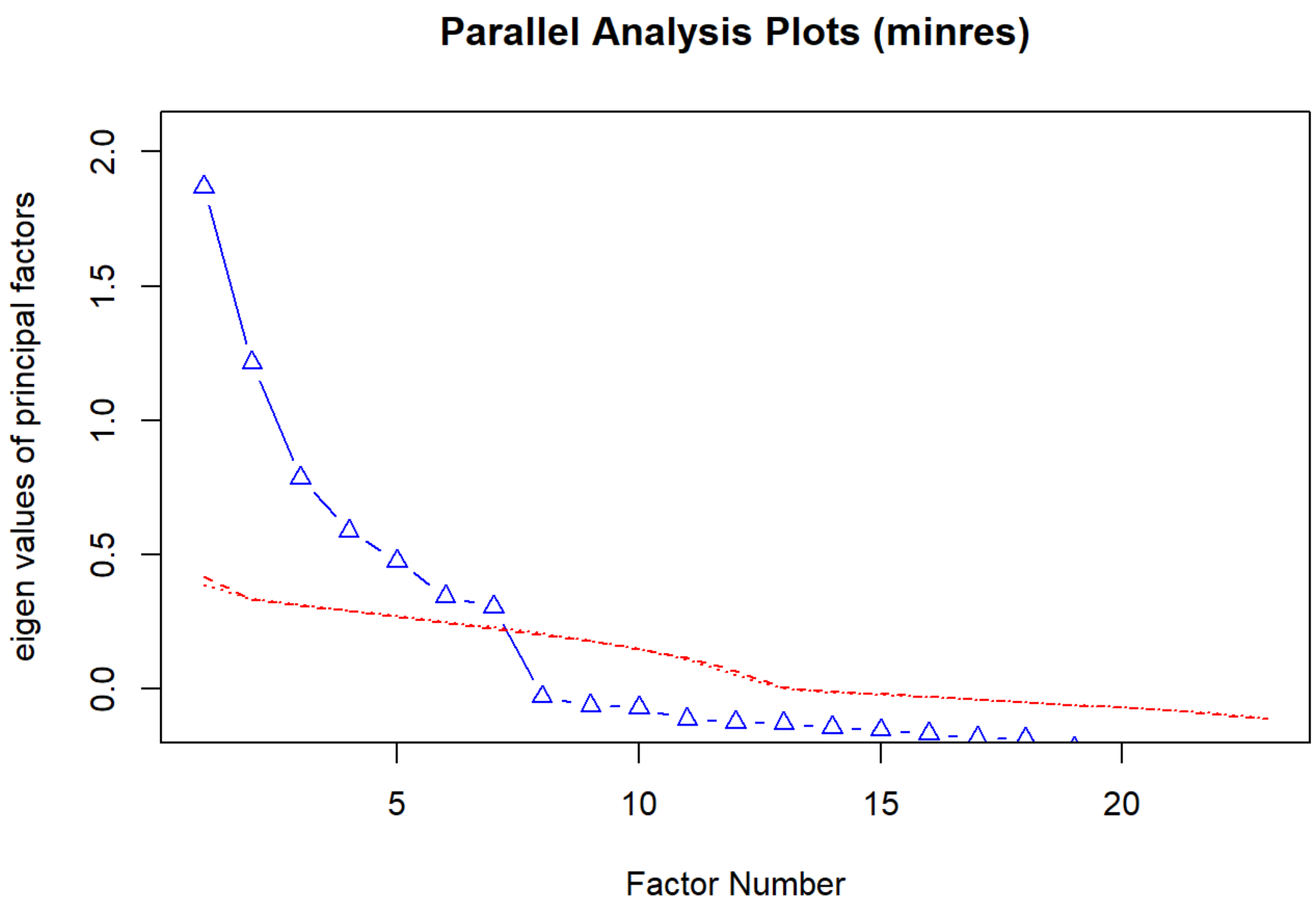


```
## Parallel analysis suggests that the number of factors =  7  and the number of components =  NA
```

```
#7 factors
```

```
FA_ET20_pa2 = fa(data_ET20_pa1, nfactors = 7, fm = "pa", rotate = "oblimin")

print(FA_ET20_pa2)
```

```
## Factor Analysis using method =  pa
## Call: fa(r = data_ET20_pa1, nfactors = 7, rotate = "oblimin", fm = "pa")
## Standardized loadings (pattern matrix) based upon correlation matrix
##                          PA1   PA7   PA3   PA2   PA4   PA5   PA6   h2   u2 com
## cr_edu_boysch           0.63  0.03 -0.06  0.01  0.08  0.03  0.02 0.46 0.54 1.1
## cr_edu_science          0.68  0.02  0.00  0.01 -0.07 -0.02  0.00 0.43 0.57 1.0
## cr_edu_girlnohelp       0.60 -0.02  0.04  0.00  0.08  0.04 -0.03 0.44 0.56 1.1
## cr_edu_raisingvoice     0.09  0.05  0.08  0.04  0.47  0.06  0.07 0.39 0.61 1.3
## cr_edu_boysfeelings     0.00 -0.02 -0.02  0.51 -0.12 -0.07  0.03 0.28 0.72 1.2
## cr_edu_boystough        0.04 -0.03 -0.01  0.52  0.05  0.05 -0.03 0.26 0.74 1.1
## cr_edu_boysdefend      -0.01  0.11  0.05  0.40  0.00  0.04  0.08 0.23 0.77 1.3
## cr_edu_girlshumble     -0.04 -0.04  0.00  0.33  0.10 -0.10  0.08 0.19 0.81 1.6
## cr_edu_equaltreat      -0.02  0.05  0.02  0.39  0.10  0.03  0.03 0.21 0.79 1.2
## cr_edu_teachpunish      0.00 -0.01  0.00  0.02  0.12  0.02  0.50 0.31 0.69 1.1
## cr_edu_teachnotpunish  0.00  0.01  0.00  0.00 -0.07 -0.02  0.56 0.30 0.70 1.0
## cr_edu_boysout          0.53 -0.01  0.09 -0.02 -0.03 -0.04  0.02 0.30 0.70 1.1
## cr_ta_eesharetasks      0.00  0.14 -0.05 -0.06  0.10  0.34 -0.05 0.19 0.81 1.7
## cr_ta_womanrole         0.06  0.02  0.02  0.00  0.61  0.01  0.01 0.44 0.56 1.0
## cr_ta_eemandecid        0.04  0.18  0.01  0.04  0.42 -0.01  0.06 0.37 0.63 1.5
## cr_fi_girlchance       -0.01  0.00  0.00  0.03  0.02  0.68 -0.01 0.45 0.55 1.0
## cr_fi_girlsavings       0.12 -0.03  0.04 -0.15 -0.13  0.40  0.04 0.26 0.74 1.8
## cr_srh_teasegirl        0.01  0.00  0.75  0.00 -0.01  0.00  0.00 0.57 0.43 1.0
## cr_srh_teaseboy        -0.01  0.00  0.76  0.00  0.02  0.00  0.00 0.58 0.42 1.0
## cr_srh_genderroles      0.04  0.09  0.06  0.24 -0.07 -0.13 -0.01 0.10 0.90 2.3
## cr_vi_hit              -0.02  0.63  0.04  0.00  0.02  0.02  0.01 0.43 0.57 1.0
## cr_vi_privematt         0.05  0.60  0.01  0.01 -0.08  0.01 -0.01 0.35 0.65 1.1
## cr_vi_tolerate          0.02  0.50 -0.03  0.00  0.15 -0.04  0.02 0.37 0.63 1.2
##
##                         PA1  PA7  PA3  PA2  PA4  PA5  PA6
## SS loadings            1.66 1.25 1.22 1.13 1.12 0.84 0.67
## Proportion Var         0.07 0.05 0.05 0.05 0.05 0.04 0.03
## Cumulative Var         0.07 0.13 0.18 0.23 0.28 0.31 0.34
## Proportion Explained  0.21 0.16 0.16 0.14 0.14 0.11 0.08
## Cumulative Proportion 0.21 0.37 0.52 0.67 0.81 0.92 1.00
##
##  With factor correlations of
##      PA1  PA7  PA3   PA2  PA4   PA5   PA6
## PA1 1.00 0.52 0.47  0.00 0.45  0.36  0.17
## PA7 0.52 1.00 0.35  0.22 0.58  0.13  0.28
## PA3 0.47 0.35 1.00  0.03 0.20  0.10  0.14
## PA2 0.00 0.22 0.03  1.00 0.25 -0.27  0.44
## PA4 0.45 0.58 0.20  0.25 1.00  0.24  0.30
## PA5 0.36 0.13 0.10 -0.27 0.24  1.00 -0.12
## PA6 0.17 0.28 0.14  0.44 0.30 -0.12  1.00
##
## Mean item complexity =  1.2
## Test of the hypothesis that 7 factors are sufficient.
##
## df null model =  253  with the objective function =  3.84 with Chi Square =  18310.6
4
## df of  the model are 113  and the objective function was  0.04
##
## The root mean square of the residuals (RMSR) is  0.01
## The df corrected root mean square of the residuals is  0.01
##
```

```
## The harmonic n.obs is  4714 with the empirical chi square  172.56  with prob <  0.00
026
## The total n.obs was  4777  with Likelihood Chi Square =  199.74  with prob <  9.1e-0
7
##
## Tucker Lewis Index of factoring reliability =  0.989
## RMSEA index =  0.013  and the 90 % confidence intervals are  0.01 0.016
## BIC =  -757.54
## Fit based upon off diagonal values = 1
## Measures of factor score adequacy
##                                                    PA1  PA7  PA3  PA2  PA4  PA5
## Correlation of (regression) scores with factors   0.88 0.84 0.87 0.79 0.83 0.77
## Multiple R square of scores with factors          0.77 0.71 0.75 0.62 0.68 0.60
## Minimum correlation of possible factor scores     0.54 0.42 0.50 0.25 0.37 0.20
##                                                    PA6
## Correlation of (regression) scores with factors   0.73
## Multiple R square of scores with factors          0.53
## Minimum correlation of possible factor scores     0.06
```

```
factors_table(FA_ET20_pa2)
```

Show 50 entries   Search:

	PA1	PA7	PA3	PA2	PA4	PA5	PA6
cr_edu_boysch	0.63	0.03	-0.06	0.01	0.08	0.03	0.02
cr_edu_science	0.68	0.02	0.00	0.01	-0.07	-0.02	-0.00
cr_edu_girlnohelp	0.60	-0.02	0.04	0.00	0.08	0.04	-0.03
cr_edu_raisingvoice	0.09	0.05	0.08	0.04	0.47	0.06	0.07
cr_edu_boysfeelings	-0.00	-0.02	-0.02	0.51	-0.12	-0.07	0.03
cr_edu_boystough	0.04	-0.03	-0.01	0.52	0.05	0.05	-0.03
cr_edu_boysdefend	-0.01	0.11	0.05	0.40	0.00	0.04	0.08
cr_edu_girlshumble	-0.04	-0.04	0.00	0.33	0.10	-0.10	0.08
cr_edu_equaltreat	-0.02	0.05	0.02	0.39	0.10	0.03	0.03
cr_edu_teachpunish	0.00	-0.01	-0.00	0.02	0.12	0.02	0.50
cr_edu_teachnotpunish	-0.00	0.01	0.00	-0.00	-0.07	-0.02	0.56
cr_edu_boysout	0.53	-0.01	0.09	-0.02	-0.03	-0.04	0.02
cr_ta_eesharetasks	0.00	0.14	-0.05	-0.06	0.10	0.34	-0.05
cr_ta_womanrole	0.06	0.02	0.02	-0.00	0.61	0.01	0.01
cr_ta_eemandecid	0.04	0.18	0.01	0.04	0.42	-0.01	0.06

	PA1	PA7	PA3	PA2	PA4	PA5	PA6
cr_fi_girlchance	-0.01	-0.00	0.00	0.03	0.02	0.68	-0.01
cr_fi_girlsavings	0.12	-0.03	0.04	-0.15	-0.13	0.40	0.04
cr_srh_teasegirl	0.01	0.00	0.75	0.00	-0.01	-0.00	-0.00
cr_srh_teaseboy	-0.01	0.00	0.76	-0.00	0.02	0.00	0.00
cr_srh_genderroles	0.04	0.09	0.06	0.24	-0.07	-0.13	-0.01
cr_vi_hit	-0.02	0.63	0.04	0.00	0.02	0.02	0.01
cr_vi_privematt	0.05	0.60	0.01	0.01	-0.08	0.01	-0.01
cr_vi_tolerate	0.02	0.50	-0.03	-0.00	0.15	-0.04	0.02

Showing 1 to 23 of 23 entries

Previous 1 Next

#another exclusion step

```
data_ET20_pa2 = data_ET20_pa1 %>% dplyr::select(-cr_srh_genderroles)
```

```
KMO(data_ET20_pa2)
```

```
## Kaiser-Meyer-Olkin factor adequacy
## Call: KMO(r = data_ET20_pa2)
## Overall MSA =  0.85
## MSA for each item = 
##          cr_edu_boysch        cr_edu_science     cr_edu_girlnohelp 
##                   0.89                  0.88                  0.90 
##   cr_edu_raisingvoice   cr_edu_boysfeelings      cr_edu_boystough 
##                   0.91                  0.77                  0.82 
##     cr_edu_boysdefend     cr_edu_girlshumble     cr_edu_equaltreat 
##                   0.86                  0.82                  0.86 
##    cr_edu_teachpunish cr_edu_teachnotpunish        cr_edu_boysout 
##                   0.83                  0.76                  0.90 
##    cr_ta_eesharetasks       cr_ta_womanrole      cr_ta_eemandecid 
##                   0.83                  0.89                  0.90 
##      cr_fi_girlchance     cr_fi_girlsavings      cr_srh_teasegirl 
##                   0.76                  0.76                  0.72 
##       cr_srh_teaseboy             cr_vi_hit        cr_vi_privematt 
##                   0.73                  0.89                  0.89 
##        cr_vi_tolerate 
##                   0.89
```

```
fa.parallel(data_ET20_pa2, fm = "pa", fa = "fa", quant = 0.95, show.legend = F, main =
"Parallel Analysis Plots (pa)", sqrt = T)
```

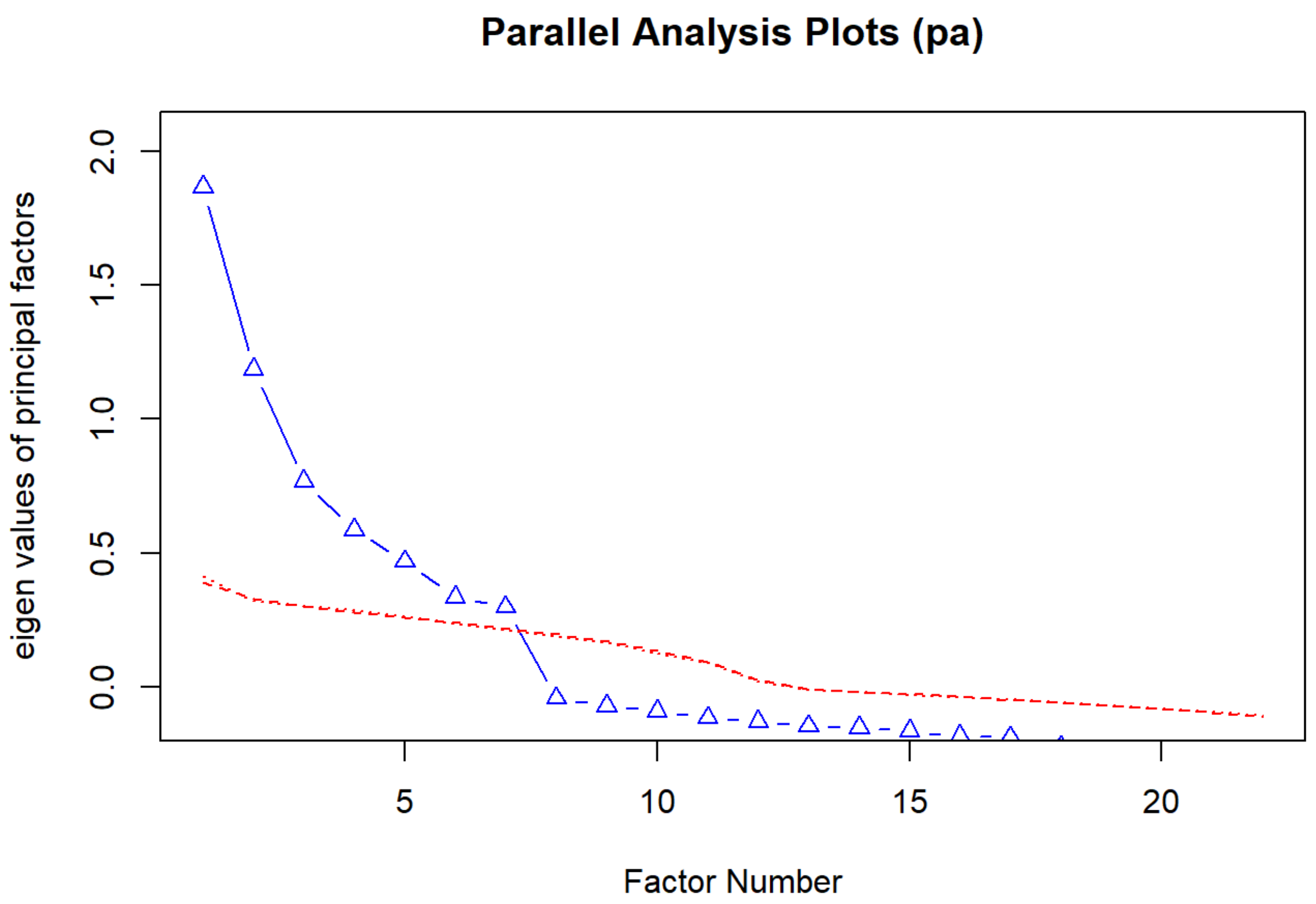


```
## Parallel analysis suggests that the number of factors =  7  and the number of compon
ents =  NA
```

```
fa.parallel(data_ET20_pa2, fm = "minres", fa = "fa", quant = 0.95, show.legend = F, mai
n = "Parallel Analysis Plots (minres)", sqrt = T)
```

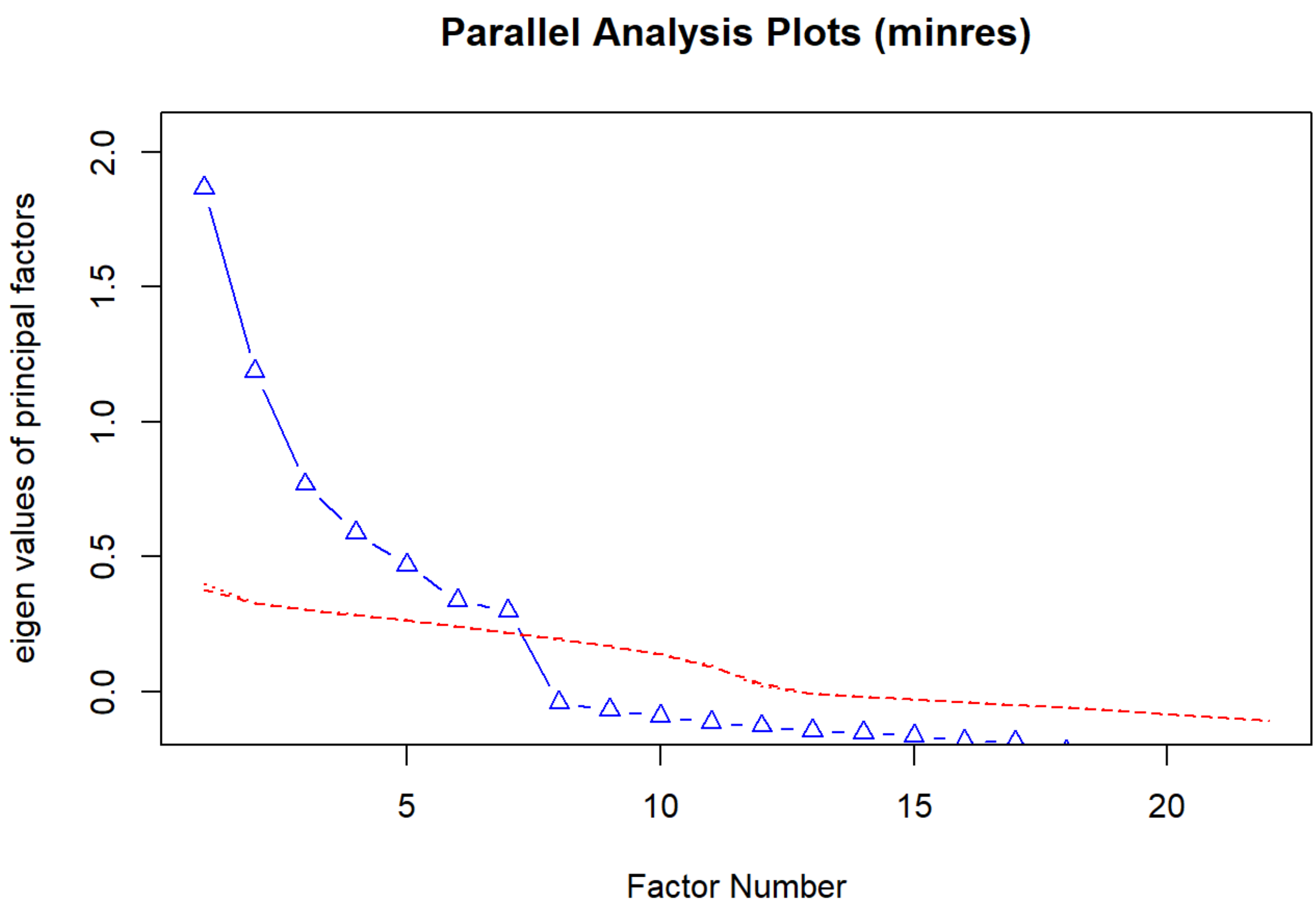


```
## Parallel analysis suggests that the number of factors =  7  and the number of compon
ents =  NA
```

```
FA_ET20_pa3 = fa(data_ET20_pa2, nfactors = 7, fm = "pa", rotate = "oblimin")

print(FA_ET20_pa3)
```

```
## Factor Analysis using method =  pa
## Call: fa(r = data_ET20_pa2, nfactors = 7, rotate = "oblimin", fm = "pa")
## Standardized loadings (pattern matrix) based upon correlation matrix
##                        PA1   PA4   PA3   PA7   PA2   PA5   PA6   h2   u2 com
## cr_edu_boysch         0.63  0.03 -0.06  0.08  0.01  0.03  0.02 0.46 0.54 1.1
## cr_edu_science        0.68  0.03  0.00 -0.07  0.01 -0.02  0.00 0.43 0.57 1.0
## cr_edu_girlnohelp     0.60 -0.02  0.04  0.09  0.00  0.04 -0.03 0.44 0.56 1.1
## cr_edu_raisingvoice   0.09  0.04  0.08  0.47  0.04  0.06  0.07 0.39 0.61 1.2
## cr_edu_boysfeelings   0.00 -0.01 -0.02 -0.13  0.52 -0.07  0.02 0.29 0.71 1.2
## cr_edu_boystough      0.03 -0.03 -0.01  0.06  0.51  0.05 -0.03 0.26 0.74 1.1
## cr_edu_boysdefend     0.00  0.11  0.05  0.00  0.40  0.04  0.07 0.23 0.77 1.3
## cr_edu_girlshumble   -0.04 -0.04  0.01  0.10  0.33 -0.10  0.08 0.19 0.81 1.6
## cr_edu_equaltreat    -0.02  0.05  0.02  0.11  0.39  0.02  0.03 0.21 0.79 1.2
## cr_edu_teachpunish    0.00 -0.01 -0.01  0.13  0.03  0.02  0.50 0.31 0.69 1.1
## cr_edu_teachnotpunish  0.00  0.01  0.00 -0.07  0.00 -0.02  0.57 0.31 0.69 1.0
## cr_edu_boysout        0.52 -0.01  0.09 -0.02 -0.03 -0.04  0.02 0.30 0.70 1.1
## cr_ta_eesharetasks    0.00  0.13 -0.05  0.10 -0.06  0.34 -0.05 0.19 0.81 1.7
## cr_ta_womanrole       0.06  0.03  0.02  0.60  0.01  0.01  0.00 0.43 0.57 1.0
## cr_ta_eemandecid      0.04  0.17  0.01  0.44  0.03  0.00  0.06 0.37 0.63 1.4
## cr_fi_girlchance     -0.02 -0.01  0.00  0.02  0.03  0.68 -0.01 0.46 0.54 1.0
## cr_fi_girlsavings     0.12 -0.03  0.04 -0.13 -0.15  0.40  0.03 0.25 0.75 1.8
## cr_srh_teasegirl      0.01  0.00  0.76 -0.01  0.00  0.00  0.00 0.58 0.42 1.0
## cr_srh_teaseboy      -0.01  0.00  0.76  0.02  0.00  0.00  0.00 0.58 0.42 1.0
## cr_vi_hit            -0.02  0.62  0.04  0.04  0.00  0.02  0.01 0.42 0.58 1.0
## cr_vi_privematt       0.05  0.61  0.01 -0.08  0.01  0.00 -0.01 0.36 0.64 1.1
## cr_vi_tolerate        0.02  0.50 -0.03  0.16  0.00 -0.04  0.02 0.37 0.63 1.2
##
##                        PA1  PA4  PA3  PA7  PA2  PA5  PA6
## SS loadings           1.65 1.23 1.22 1.14 1.07 0.83 0.67
## Proportion Var        0.08 0.06 0.06 0.05 0.05 0.04 0.03
## Cumulative Var        0.08 0.13 0.19 0.24 0.29 0.32 0.35
## Proportion Explained  0.21 0.16 0.16 0.15 0.14 0.11 0.09
## Cumulative Proportion 0.21 0.37 0.53 0.67 0.81 0.91 1.00
##
##  With factor correlations of
##       PA1  PA4  PA3  PA7   PA2   PA5   PA6
## PA1  1.00 0.52 0.47 0.45 -0.01  0.37  0.17
## PA4  0.52 1.00 0.34 0.58  0.21  0.14  0.27
## PA3  0.47 0.34 1.00 0.20  0.02  0.11  0.14
## PA7  0.45 0.58 0.20 1.00  0.25  0.24  0.30
## PA2 -0.01 0.21 0.02 0.25  1.00 -0.26  0.44
## PA5  0.37 0.14 0.11 0.24 -0.26  1.00 -0.12
## PA6  0.17 0.27 0.14 0.30  0.44 -0.12  1.00
##
## Mean item complexity =  1.2
## Test of the hypothesis that 7 factors are sufficient.
##
## df null model =  231  with the objective function =  3.77 with Chi Square =  17966.8
4
## df of  the model are 98  and the objective function was  0.04
##
## The root mean square of the residuals (RMSR) is  0.01
## The df corrected root mean square of the residuals is  0.01
##
## The harmonic n.obs is  4716 with the empirical chi square  149.19  with prob <  0.00
```

```
067
## The total n.obs was  4777  with Likelihood Chi Square =  179.09  with prob <  1.1e-0
6
##
## Tucker Lewis Index of factoring reliability =  0.989
## RMSEA index =  0.013  and the 90 % confidence intervals are  0.01 0.016
## BIC =  -651.12
## Fit based upon off diagonal values = 1
## Measures of factor score adequacy
##                                                    PA1  PA4  PA3  PA7  PA2  PA5
## Correlation of (regression) scores with factors   0.88 0.84 0.87 0.83 0.78 0.77
## Multiple R square of scores with factors          0.77 0.71 0.75 0.68 0.61 0.60
## Minimum correlation of possible factor scores     0.54 0.41 0.50 0.37 0.23 0.20
##                                                    PA6
## Correlation of (regression) scores with factors   0.73
## Multiple R square of scores with factors          0.53
## Minimum correlation of possible factor scores     0.06
```

```
factors_table(FA_ET20_pa3)
```

Show 50 entries Search:

	PA1	PA4	PA3	PA7	PA2	PA5	PA6
cr_edu_boysch	0.63	0.03	-0.06	0.08	0.01	0.03	0.02
cr_edu_science	0.68	0.03	0.00	-0.07	0.01	-0.02	-0.00
cr_edu_girlnohelp	0.60	-0.02	0.04	0.09	-0.00	0.04	-0.03
cr_edu_raisingvoice	0.09	0.04	0.08	0.47	0.04	0.06	0.07
cr_edu_boysfeelings	0.00	-0.01	-0.02	-0.13	0.52	-0.07	0.02
cr_edu_boystough	0.03	-0.03	-0.01	0.06	0.51	0.05	-0.03
cr_edu_boysdefend	-0.00	0.11	0.05	0.00	0.40	0.04	0.07
cr_edu_girlshumble	-0.04	-0.04	0.01	0.10	0.33	-0.10	0.08
cr_edu_equaltreat	-0.02	0.05	0.02	0.11	0.39	0.02	0.03
cr_edu_teachpunish	0.00	-0.01	-0.01	0.13	0.03	0.02	0.50
cr_edu_teachnotpunish	-0.00	0.01	-0.00	-0.07	-0.00	-0.02	0.57
cr_edu_boysout	0.52	-0.01	0.09	-0.02	-0.03	-0.04	0.02
cr_ta_eesharetasks	0.00	0.13	-0.05	0.10	-0.06	0.34	-0.05
cr_ta_womanrole	0.06	0.03	0.02	0.60	0.01	0.01	0.00
cr_ta_eemandecid	0.04	0.17	0.01	0.44	0.03	-0.00	0.06

	PA1	PA4	PA3	PA7	PA2	PA5	PA6
cr_fi_girlchance	-0.02	-0.01	-0.00	0.02	0.03	0.68	-0.01
cr_fi_girlsavings	0.12	-0.03	0.04	-0.13	-0.15	0.40	0.03
cr_srh_teasegirl	0.01	0.00	0.76	-0.01	0.00	-0.00	-0.00
cr_srh_teaseboy	-0.01	0.00	0.76	0.02	-0.00	0.00	0.00
cr_vi_hit	-0.02	0.62	0.04	0.04	-0.00	0.02	0.01
cr_vi_privematt	0.05	0.61	0.01	-0.08	0.01	0.00	-0.01
cr_vi_tolerate	0.02	0.50	-0.03	0.16	0.00	-0.04	0.02

Showing 1 to 22 of 22 entries

Previous 1 Next

#removing low communalities

```
data_ET20_pa3 = remove_low_communality(data_ET20_pa2, FA_ET20_pa3, 0.2)
```

```
## [1] "cr_edu_girlshumble" "cr_ta_eesharetasks"
```

```
#removes "cr_edu_girlshumble" "cr_ta_eesharetasks"
```

```
KMO(data_ET20_pa3)
```

```
## Kaiser-Meyer-Olkin factor adequacy
## Call: KMO(r = data_ET20_pa3)
## Overall MSA =  0.85
## MSA for each item = 
##         cr_edu_boysch        cr_edu_science     cr_edu_girlnohelp 
##                  0.89                  0.88                  0.89 
##   cr_edu_raisingvoice   cr_edu_boysfeelings      cr_edu_boystough 
##                  0.91                  0.73                  0.80 
##      cr_edu_boysdefend      cr_edu_equaltreat    cr_edu_teachpunish 
##                  0.85                  0.84                  0.82 
## cr_edu_teachnotpunish         cr_edu_boysout       cr_ta_womanrole 
##                  0.74                  0.90                  0.88 
##       cr_ta_eemandecid      cr_fi_girlchance     cr_fi_girlsavings 
##                  0.90                  0.74                  0.71 
##       cr_srh_teasegirl        cr_srh_teaseboy             cr_vi_hit 
##                  0.72                  0.73                  0.89 
##        cr_vi_privematt        cr_vi_tolerate 
##                  0.89                  0.89
```

```
fa.parallel(data_ET20_pa3, fm = "pa", fa = "fa", quant = 0.95, show.legend = F, main =
"Parallel Analysis Plots (pa)", sqrt = T)
```

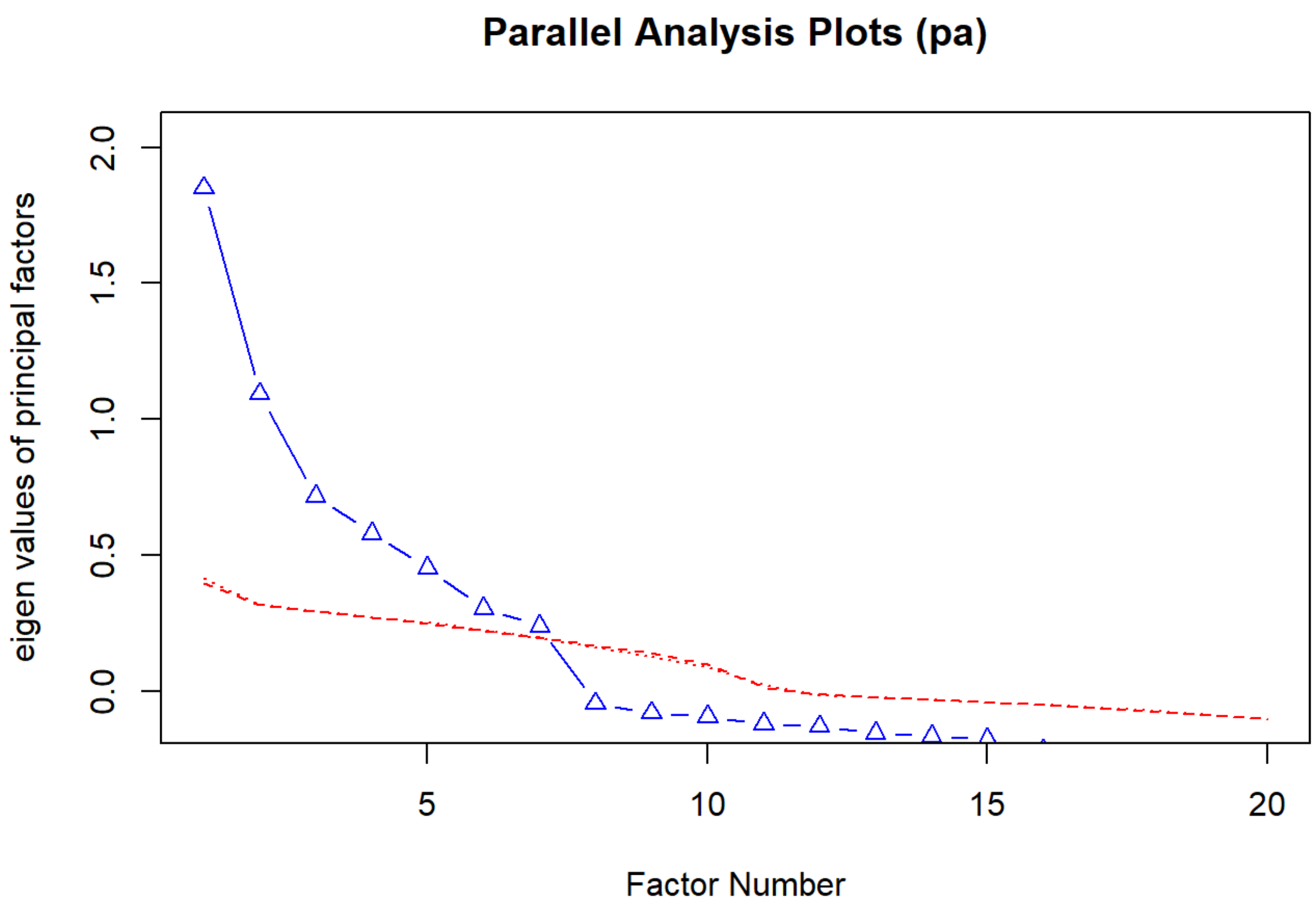


```
## Parallel analysis suggests that the number of factors =  7  and the number of compon
ents =  NA
```

```
fa.parallel(data_ET20_pa3, fm = "minres", fa = "fa", quant = 0.95, show.legend = F, mai
n = "Parallel Analysis Plots (minres)", sqrt = T)
```

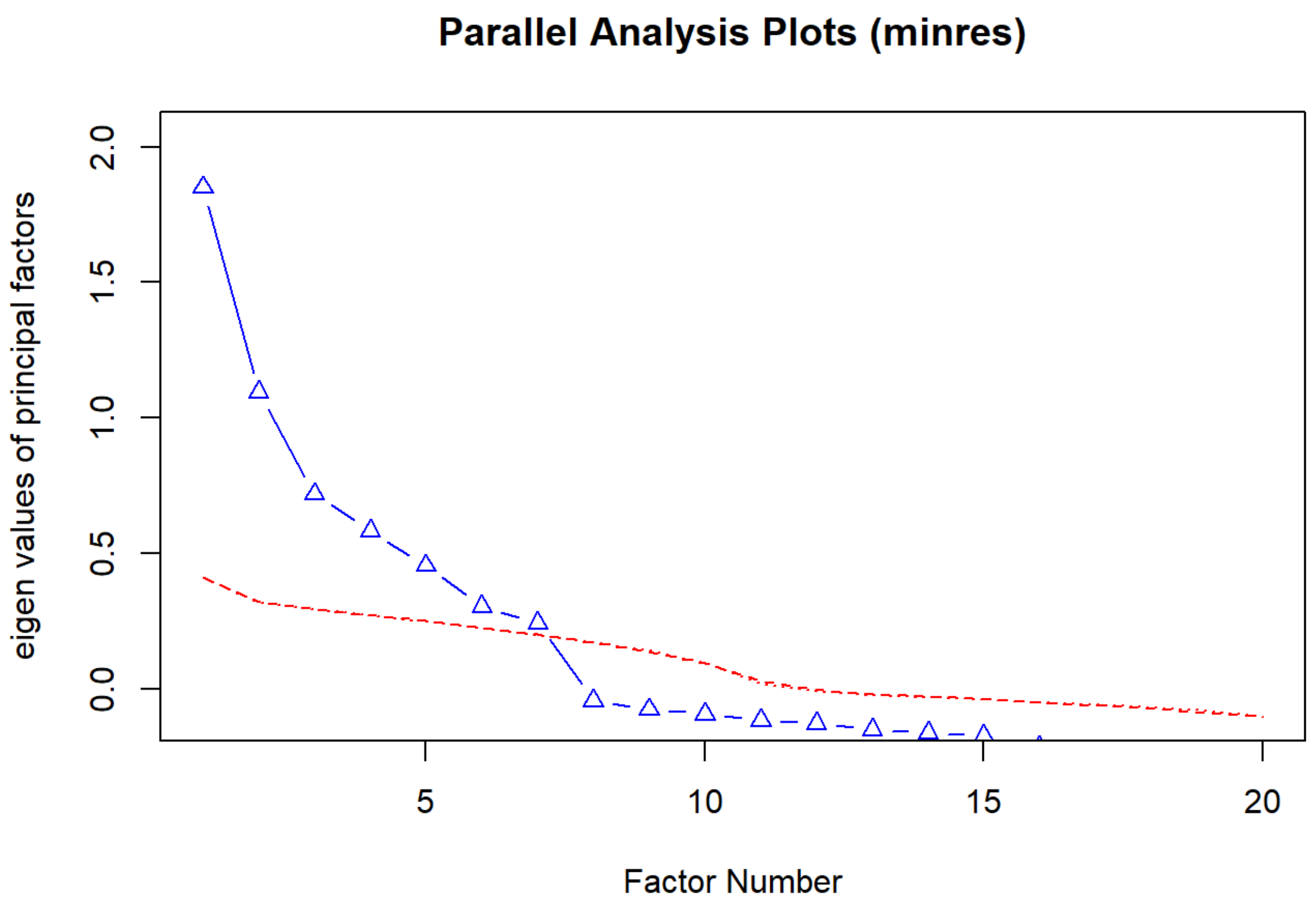


```
## Parallel analysis suggests that the number of factors =  7  and the number of compon
ents =  NA
```

```
FA_ET20_pa4 = fa(data_ET20_pa3, nfactors = 7, fm = "pa", rotate = "oblimin")

print(FA_ET20_pa4)
```

```
## Factor Analysis using method =  pa
## Call: fa(r = data_ET20_pa3, nfactors = 7, rotate = "oblimin", fm = "pa")
## Standardized loadings (pattern matrix) based upon correlation matrix
##                          PA1   PA4   PA3   PA5   PA2   PA6   PA7   h2   u2 com
## cr_edu_boysch           0.64  0.02 -0.06  0.07  0.01  0.02  0.02 0.46 0.54 1.1
## cr_edu_science          0.68  0.02  0.00 -0.08  0.01 -0.01 -0.01 0.43 0.57 1.0
## cr_edu_girlnohelp       0.60 -0.02  0.04  0.09  0.00 -0.03  0.04 0.44 0.56 1.1
## cr_edu_raisingvoice     0.09  0.07  0.08  0.44  0.04  0.09  0.05 0.38 0.62 1.3
## cr_edu_boysfeelings     0.00 -0.03 -0.02 -0.13  0.49  0.02 -0.09 0.28 0.72 1.2
## cr_edu_boystough        0.02 -0.03 -0.01  0.06  0.51 -0.02  0.03 0.25 0.75 1.1
## cr_edu_boysdefend      -0.01  0.10  0.05  0.00  0.41  0.07  0.02 0.23 0.77 1.2
## cr_edu_equaltreat      -0.03  0.05  0.02  0.10  0.40  0.03  0.01 0.22 0.78 1.2
## cr_edu_teachpunish      0.00 -0.02 -0.01  0.10  0.02  0.55  0.02 0.34 0.66 1.1
## cr_edu_teachnotpunish  0.00  0.00  0.00 -0.09  0.00  0.53 -0.03 0.27 0.73 1.1
## cr_edu_boysout          0.52 -0.01  0.09 -0.03 -0.03  0.03 -0.03 0.30 0.70 1.1
## cr_ta_womanrole         0.06  0.04  0.02  0.60  0.01  0.02  0.00 0.45 0.55 1.0
## cr_ta_eemandecid        0.04  0.19  0.01  0.41  0.03  0.07 -0.01 0.37 0.63 1.5
## cr_fi_girlchance       -0.01  0.00 -0.02  0.11  0.04 -0.04  0.54 0.32 0.68 1.1
## cr_fi_girlsavings       0.04  0.01  0.02 -0.12 -0.07  0.03  0.56 0.34 0.66 1.1
## cr_srh_teasegirl        0.01  0.00  0.76 -0.01  0.00  0.00 -0.01 0.58 0.42 1.0
## cr_srh_teaseboy        -0.01  0.01  0.75  0.02  0.00  0.00  0.01 0.57 0.43 1.0
## cr_vi_hit              -0.02  0.62  0.04  0.04  0.00  0.01  0.01 0.42 0.58 1.0
## cr_vi_privematt         0.04  0.62  0.01 -0.09  0.01 -0.02  0.01 0.36 0.64 1.1
## cr_vi_tolerate          0.02  0.51 -0.03  0.15  0.00  0.02 -0.04 0.37 0.63 1.2
##
##                         PA1  PA4  PA3  PA5  PA2  PA6  PA7
## SS loadings            1.64 1.23 1.22 1.05 0.90 0.67 0.66
## Proportion Var         0.08 0.06 0.06 0.05 0.05 0.03 0.03
## Cumulative Var         0.08 0.14 0.20 0.26 0.30 0.34 0.37
## Proportion Explained  0.22 0.17 0.17 0.14 0.12 0.09 0.09
## Cumulative Proportion 0.22 0.39 0.55 0.70 0.82 0.91 1.00
##
##  With factor correlations of
##      PA1  PA4  PA3  PA5   PA2   PA6   PA7
## PA1 1.00 0.53 0.47 0.44  0.05  0.20  0.39
## PA4 0.53 1.00 0.35 0.56  0.25  0.31  0.09
## PA3 0.47 0.35 1.00 0.18  0.04  0.15  0.14
## PA5 0.44 0.56 0.18 1.00  0.25  0.30  0.17
## PA2 0.05 0.25 0.04 0.25  1.00  0.45 -0.31
## PA6 0.20 0.31 0.15 0.30  0.45  1.00 -0.12
## PA7 0.39 0.09 0.14 0.17 -0.31 -0.12  1.00
##
## Mean item complexity =  1.1
## Test of the hypothesis that 7 factors are sufficient.
##
## df null model =  190  with the objective function =  3.5 with Chi Square =  16698.91
## df of  the model are 71  and the objective function was  0.03
##
## The root mean square of the residuals (RMSR) is  0.01
## The df corrected root mean square of the residuals is  0.01
##
## The harmonic n.obs is  4714 with the empirical chi square  107.94  with prob <  0.00
31
## The total n.obs was  4777  with Likelihood Chi Square =  136.31  with prob <  5.1e-0
6
```

```
##
## Tucker Lewis Index of factoring reliability =  0.989
## RMSEA index =  0.014  and the 90 % confidence intervals are  0.01 0.017
## BIC =  -465.17
## Fit based upon off diagonal values = 1
## Measures of factor score adequacy
##                                                    PA1  PA4  PA3  PA5  PA2  PA6
## Correlation of (regression) scores with factors   0.88 0.84 0.87 0.81 0.77 0.73
## Multiple R square of scores with factors          0.77 0.71 0.75 0.66 0.59 0.54
## Minimum correlation of possible factor scores     0.54 0.42 0.50 0.33 0.17 0.07
##                                                    PA7
## Correlation of (regression) scores with factors   0.74
## Multiple R square of scores with factors          0.54
## Minimum correlation of possible factor scores     0.09
```

```
factors_table(FA_ET20_pa4)
```

Show 50 entries Search:

	PA1	PA4	PA3	PA5	PA2	PA6	PA7
cr_edu_boysch	0.64	0.02	-0.06	0.07	0.01	0.02	0.02
cr_edu_science	0.68	0.02	0.00	-0.08	0.01	-0.01	-0.01
cr_edu_girlnohelp	0.60	-0.02	0.04	0.09	-0.00	-0.03	0.04
cr_edu_raisingvoice	0.09	0.07	0.08	0.44	0.04	0.09	0.05
cr_edu_boysfeelings	-0.00	-0.03	-0.02	-0.13	0.49	0.02	-0.09
cr_edu_boystough	0.02	-0.03	-0.01	0.06	0.51	-0.02	0.03
cr_edu_boysdefend	-0.01	0.10	0.05	-0.00	0.41	0.07	0.02
cr_edu_equaltreat	-0.03	0.05	0.02	0.10	0.40	0.03	0.01
cr_edu_teachpunish	-0.00	-0.02	-0.01	0.10	0.02	0.55	0.02
cr_edu_teachnotpunish	0.00	0.00	0.00	-0.09	0.00	0.53	-0.03
cr_edu_boysout	0.52	-0.01	0.09	-0.03	-0.03	0.03	-0.03
cr_ta_womanrole	0.06	0.04	0.02	0.60	0.01	0.02	0.00
cr_ta_eemandecid	0.04	0.19	0.01	0.41	0.03	0.07	-0.01
cr_fi_girlchance	-0.01	-0.00	-0.02	0.11	0.04	-0.04	0.54
cr_fi_girlsavings	0.04	0.01	0.02	-0.12	-0.07	0.03	0.56
cr_srh_teasegirl	0.01	-0.00	0.76	-0.01	0.00	-0.00	-0.01
cr_srh_teaseboy	-0.01	0.01	0.75	0.02	0.00	0.00	0.01

	PA1	PA4	PA3	PA5	PA2	PA6	PA7
cr_vi_hit	-0.02	0.62	0.04	0.04	-0.00	0.01	0.01
cr_vi_privematt	0.04	0.62	0.01	-0.09	0.01	-0.02	0.01
cr_vi_tolerate	0.02	0.51	-0.03	0.15	0.00	0.02	-0.04

Showing 1 to 20 of 20 entries

Previous 1 Next

#final version

5.2 Confirmatory Factor Analysis (CFA) for GAGE gender norms scale

```
#1st step: adapt measurement model to results of EFA
MMCFA1 <- '
#Factor loadings
GenderAttitudes =~ cr_edu_boysch+ cr_edu_science+ cr_edu_girlnohelp+ cr_edu_raisingvoic
e+ cr_edu_boysfeelings+ cr_edu_boystough+ cr_edu_boysdefend+ cr_edu_equaltreat+ cr_edu_
teachpunish+ cr_edu_teachnotpunish+ cr_edu_boysout+ cr_ta_womanrole+ cr_ta_eemandecid+
cr_fi_girlchance+ cr_fi_girlsavings+ cr_srh_teasegirl+ cr_srh_teaseboy+cr_vi_hit+ cr_vi
_privematt+ cr_vi_tolerate
'

#Estimator MLR because the variables are not normally distributed
fit.MMCFA1 <- cfa(MMCFA1, data=data_ET20_subset_GNS_CFA, estimator="MLR", mimic="Mplu
s")
```

```
## Warning: lavaan->lav_data_full():
##    some cases are empty and will be ignored: 83 85 87 126 141 279 327 589 592
##    909 921 2273 2771 2772 2773 3758 4324.
```

```
summary(fit.MMCFA1, fit.measures=TRUE, standardized=TRUE, rsq=TRUE)
```

```
## lavaan 0.6-21 ended normally after 50 iterations
##
##   Estimator                                         ML
##   Optimization method                           NLMINB
##   Number of model parameters                        60
##
##                                                   Used       Total
##   Number of observations                          4760        4777
##   Number of missing patterns                        79
##
## Model Test User Model:
##                                               Standard      Scaled
##   Test Statistic                              5983.709    5313.513
##   Degrees of freedom                               170         170
##   P-value (Chi-square)                           0.000       0.000
##   Scaling correction factor                                  1.126
##     Yuan-Bentler correction (Mplus variant)
##
## Model Test Baseline Model:
##
##   Test statistic                             16550.414   14719.355
##   Degrees of freedom                               190         190
##   P-value                                        0.000       0.000
##   Scaling correction factor                                  1.124
##
## User Model versus Baseline Model:
##
##   Comparative Fit Index (CFI)                    0.645       0.646
##   Tucker-Lewis Index (TLI)                       0.603       0.604
##
##   Robust Comparative Fit Index (CFI)                         0.645
##   Robust Tucker-Lewis Index (TLI)                            0.603
##
## Loglikelihood and Information Criteria:
##
##   Loglikelihood user model (H0)            -107221.579 -107221.579
##   Scaling correction factor                                  0.996
##       for the MLR correction
##   Loglikelihood unrestricted model (H1)             NA          NA
##   Scaling correction factor                                  1.092
##       for the MLR correction
##
##   Akaike (AIC)                              214563.159  214563.159
##   Bayesian (BIC)                            214951.239  214951.239
##   Sample-size adjusted Bayesian (SABIC)     214760.581  214760.581
##
## Root Mean Square Error of Approximation:
##
##   RMSEA                                          0.085       0.080
##   90 Percent confidence interval - lower         0.083       0.078
##   90 Percent confidence interval - upper         0.087       0.081
##   P-value H_0: RMSEA <= 0.050                    0.000       0.000
##   P-value H_0: RMSEA >= 0.080                    1.000       0.400
##
##   Robust RMSEA                                               0.085
```

```
##   90 Percent confidence interval - lower                     0.083
##   90 Percent confidence interval - upper                     0.087
##   P-value H_0: Robust RMSEA <= 0.050                         0.000
##   P-value H_0: Robust RMSEA >= 0.080                         1.000
##
## Standardized Root Mean Square Residual:
##
##   SRMR                                         0.073       0.073
##
## Parameter Estimates:
##
##   Standard errors                            Sandwich
##   Information bread                          Observed
##   Observed information based on               Hessian
##
## Latent Variables:
##                      Estimate  Std.Err  z-value  P(>|z|)   Std.lv  Std.all
##   GenderAttitudes =~
##     cr_edu_boysch       1.000                               0.493    0.591
##     cr_edu_science      0.731    0.022   33.120    0.000    0.361    0.525
##     cr_edu_grlnhlp      0.941    0.024   38.875    0.000    0.464    0.576
##     cr_edu_rasngvc      1.069    0.037   28.990    0.000    0.527    0.562
##     cr_ed_bysflngs     -0.010    0.022   -0.439    0.661   -0.005   -0.007
##     cr_edu_boystgh      0.247    0.026    9.480    0.000    0.122    0.166
##     cr_edu_bysdfnd      0.410    0.031   13.227    0.000    0.202    0.241
##     cr_edu_equltrt      0.360    0.030   11.959    0.000    0.178    0.222
##     cr_edu_tchpnsh      0.446    0.030   14.991    0.000    0.220    0.276
##     cr_d_tchntpnsh      0.260    0.029    8.893    0.000    0.128    0.149
##     cr_edu_boysout      0.702    0.026   27.232    0.000    0.346    0.457
##     cr_ta_womanrol      0.973    0.038   25.879    0.000    0.480    0.532
##     cr_ta_eemandcd      0.975    0.038   25.376    0.000    0.481    0.532
##     cr_fi_girlchnc      0.276    0.022   12.308    0.000    0.136    0.204
##     cr_fi_grlsvngs      0.108    0.017    6.466    0.000    0.053    0.110
##     cr_srh_teasgrl      0.653    0.031   20.837    0.000    0.322    0.401
##     cr_srh_teaseby      0.664    0.031   21.448    0.000    0.327    0.414
##     cr_vi_hit           1.001    0.037   26.919    0.000    0.493    0.528
##     cr_vi_privemtt      0.886    0.034   25.987    0.000    0.437    0.471
##     cr_vi_tolerate      0.952    0.038   25.079    0.000    0.469    0.508
##
## Intercepts:
##                    Estimate  Std.Err  z-value  P(>|z|)   Std.lv  Std.all
##    .cr_edu_boysch     2.453    0.012  202.479    0.000    2.453    2.941
##    .cr_edu_science    2.676    0.010  266.878    0.000    2.676    3.891
##    .cr_edu_grlnhlp    2.508    0.012  214.317    0.000    2.508    3.113
##    .cr_edu_rasngvc    2.008    0.014  147.437    0.000    2.008    2.139
##    .cr_ed_bysflngs    1.351    0.010  138.221    0.000    1.351    2.008
##    .cr_edu_boystgh    1.456    0.011  136.633    0.000    1.456    1.984
##    .cr_edu_bysdfnd    1.634    0.012  134.316    0.000    1.634    1.951
##    .cr_edu_equltrt    1.550    0.012  133.577    0.000    1.550    1.939
##    .cr_edu_tchpnsh    1.529    0.012  132.403    0.000    1.529    1.923
##    .cr_d_tchntpnsh    1.659    0.012  132.742    0.000    1.659    1.931
##    .cr_edu_boysout    2.571    0.011  233.804    0.000    2.571    3.394
##    .cr_ta_womanrol    1.755    0.013  134.317    0.000    1.755    1.948
##    .cr_ta_eemandcd    1.784    0.013  135.998    0.000    1.784    1.972
##    .cr_fi_girlchnc    2.673    0.010  275.048    0.000    2.673    4.002
##    .cr_fi_grlsvngs    2.827    0.007  399.623    0.000    2.827    5.832
```

```
##    .cr_srh_teasgrl    2.481    0.012  211.390    0.000    2.481    3.087
##    .cr_srh_teaseby    2.502    0.012  216.942    0.000    2.502    3.167
##    .cr_vi_hit         2.122    0.014  156.378    0.000    2.122    2.273
##    .cr_vi_privemtt    2.159    0.013  160.160    0.000    2.159    2.329
##    .cr_vi_tolerate    1.936    0.013  144.184    0.000    1.936    2.095
##
## Variances:
##                    Estimate  Std.Err  z-value  P(>|z|)   Std.lv  Std.all
##    .cr_edu_boysch     0.452    0.011   39.978    0.000    0.452    0.650
##    .cr_edu_science    0.343    0.010   34.846    0.000    0.343    0.725
##    .cr_edu_grlnhlp    0.434    0.011   38.391    0.000    0.434    0.668
##    .cr_edu_rasngvc    0.604    0.012   50.548    0.000    0.604    0.685
##    .cr_ed_bysflngs    0.453    0.012   38.314    0.000    0.453    1.000
##    .cr_edu_boystgh    0.524    0.011   48.417    0.000    0.524    0.972
##    .cr_edu_bysdfnd    0.661    0.010   67.329    0.000    0.661    0.942
##    .cr_edu_equltrt    0.608    0.010   58.308    0.000    0.608    0.951
##    .cr_edu_tchpnsh    0.584    0.011   55.227    0.000    0.584    0.924
##    .cr_d_tchntpnsh    0.722    0.009   76.508    0.000    0.722    0.978
##    .cr_edu_boysout    0.454    0.011   40.904    0.000    0.454    0.792
##    .cr_ta_womanrol    0.582    0.012   50.032    0.000    0.582    0.717
##    .cr_ta_eemandcd    0.587    0.012   48.819    0.000    0.587    0.717
##    .cr_fi_girlchnc    0.428    0.012   35.976    0.000    0.428    0.958
##    .cr_fi_grlsvngs    0.232    0.010   22.905    0.000    0.232    0.988
##    .cr_srh_teasgrl    0.542    0.012   46.603    0.000    0.542    0.839
##    .cr_srh_teaseby    0.517    0.011   45.469    0.000    0.517    0.828
##    .cr_vi_hit         0.628    0.012   51.156    0.000    0.628    0.721
##    .cr_vi_privemtt    0.669    0.012   57.456    0.000    0.669    0.778
##    .cr_vi_tolerate    0.634    0.012   53.492    0.000    0.634    0.742
##     GenderAttituds    0.243    0.012   20.275    0.000    1.000    1.000
##
## R-Square:
##                    Estimate
##     cr_edu_boysch     0.350
##     cr_edu_science    0.275
##     cr_edu_grlnhlp    0.332
##     cr_edu_rasngvc    0.315
##     cr_ed_bysflngs    0.000
##     cr_edu_boystgh    0.028
##     cr_edu_bysdfnd    0.058
##     cr_edu_equltrt    0.049
##     cr_edu_tchpnsh    0.076
##     cr_d_tchntpnsh    0.022
##     cr_edu_boysout    0.208
##     cr_ta_womanrol    0.283
##     cr_ta_eemandcd    0.283
##     cr_fi_girlchnc    0.042
##     cr_fi_grlsvngs    0.012
##     cr_srh_teasgrl    0.161
##     cr_srh_teaseby    0.172
##     cr_vi_hit         0.279
##     cr_vi_privemtt    0.222
##     cr_vi_tolerate    0.258
```

```
#FL>0.7 / 0.6
#TLI, CFI >0.9
#RMSEA, SRMR <0.08
```

```
#2nd step: reduce unfit variables: -cr_edu_boysfeelings, cr_edu_boystough, cr_edu_boysd
efend, cr_edu_equaltreat, cr_edu_teachpunish, cr_edu_teachnotpunish,cr_fi_girlchance, c
r_fi_girlsavings
MMCFA2 <- '
#Factor loadings
GenderAttitudes =~ cr_edu_boysch+ cr_edu_science+ cr_edu_girlnohelp+ cr_edu_raisingvoic
e+  cr_edu_boysout+ cr_ta_womanrole+ cr_ta_eemandecid+ cr_srh_teasegirl+ cr_srh_teasebo
y+cr_vi_hit+ cr_vi_privematt+ cr_vi_tolerate
'

#Estimator MLR because the variables are not normally distributed
fit.MMCFA2 <- cfa(MMCFA2, data=data_ET20_subset_GNS_CFA, estimator="MLR", mimic="Mplu
s")
```

```
## Warning: lavaan->lav_data_full():
##    some cases are empty and will be ignored: 83 85 87 126 141 279 327 589 592
##    909 921 2273 2771 2772 2773 3758 4324.
```

```
summary(fit.MMCFA2, fit.measures=TRUE, standardized=TRUE, rsq=TRUE)
```

```
## lavaan 0.6-21 ended normally after 39 iterations
##
##   Estimator                                         ML
##   Optimization method                           NLMINB
##   Number of model parameters                        36
##
##                                                   Used       Total
##   Number of observations                          4760        4777
##   Number of missing patterns                        41
##
## Model Test User Model:
##                                               Standard      Scaled
##   Test Statistic                              3083.645    2640.719
##   Degrees of freedom                                54          54
##   P-value (Chi-square)                           0.000       0.000
##   Scaling correction factor                                  1.168
##     Yuan-Bentler correction (Mplus variant)
##
## Model Test Baseline Model:
##
##   Test statistic                             12604.643   10721.213
##   Degrees of freedom                                66          66
##   P-value                                        0.000       0.000
##   Scaling correction factor                                  1.176
##
## User Model versus Baseline Model:
##
##   Comparative Fit Index (CFI)                    0.758       0.757
##   Tucker-Lewis Index (TLI)                       0.705       0.703
##
##   Robust Comparative Fit Index (CFI)                         0.758
##   Robust Tucker-Lewis Index (TLI)                            0.705
##
## Loglikelihood and Information Criteria:
##
##   Loglikelihood user model (H0)             -66419.192  -66419.192
##   Scaling correction factor                                  0.919
##       for the MLR correction
##   Loglikelihood unrestricted model (H1)             NA          NA
##   Scaling correction factor                                  1.068
##       for the MLR correction
##
##   Akaike (AIC)                              132910.383  132910.383
##   Bayesian (BIC)                            133143.232  133143.232
##   Sample-size adjusted Bayesian (SABIC)     133028.837  133028.837
##
## Root Mean Square Error of Approximation:
##
##   RMSEA                                          0.109       0.100
##   90 Percent confidence interval - lower         0.105       0.097
##   90 Percent confidence interval - upper         0.112       0.103
##   P-value H_0: RMSEA <= 0.050                    0.000       0.000
##   P-value H_0: RMSEA >= 0.080                    1.000       1.000
##
##   Robust RMSEA                                               0.109
```

```
##   90 Percent confidence interval - lower                      0.106
##   90 Percent confidence interval - upper                      0.112
##   P-value H_0: Robust RMSEA <= 0.050                          0.000
##   P-value H_0: Robust RMSEA >= 0.080                          1.000
##
## Standardized Root Mean Square Residual:
##
##   SRMR                                         0.069       0.069
##
## Parameter Estimates:
##
##   Standard errors                            Sandwich
##   Information bread                          Observed
##   Observed information based on               Hessian
##
## Latent Variables:
##                      Estimate  Std.Err  z-value  P(>|z|)   Std.lv  Std.all
##   GenderAttitudes =~
##     cr_edu_boysch       1.000                               0.503    0.603
##     cr_edu_science      0.743    0.022   33.283    0.000    0.374    0.543
##     cr_edu_grlnhlp      0.953    0.024   39.180    0.000    0.479    0.595
##     cr_edu_rasngvc      1.011    0.035   28.759    0.000    0.509    0.542
##     cr_edu_boysout      0.714    0.026   27.703    0.000    0.359    0.474
##     cr_ta_womanrol      0.922    0.036   25.649    0.000    0.464    0.515
##     cr_ta_eemandcd      0.920    0.036   25.406    0.000    0.463    0.512
##     cr_srh_teasgrl      0.662    0.032   20.879    0.000    0.333    0.414
##     cr_srh_teaseby      0.671    0.031   21.397    0.000    0.338    0.427
##     cr_vi_hit           0.970    0.037   26.548    0.000    0.488    0.523
##     cr_vi_privemtt      0.868    0.034   25.784    0.000    0.437    0.471
##     cr_vi_tolerate      0.914    0.037   24.999    0.000    0.460    0.497
##
## Intercepts:
##                    Estimate  Std.Err  z-value  P(>|z|)   Std.lv  Std.all
##    .cr_edu_boysch     2.453    0.012  202.522    0.000    2.453    2.942
##    .cr_edu_science    2.676    0.010  267.022    0.000    2.676    3.892
##    .cr_edu_grlnhlp    2.508    0.012  214.373    0.000    2.508    3.113
##    .cr_edu_rasngvc    2.008    0.014  147.439    0.000    2.008    2.139
##    .cr_edu_boysout    2.571    0.011  233.822    0.000    2.571    3.394
##    .cr_ta_womanrol    1.755    0.013  134.309    0.000    1.755    1.948
##    .cr_ta_eemandcd    1.784    0.013  135.996    0.000    1.784    1.972
##    .cr_srh_teasgrl    2.481    0.012  211.396    0.000    2.481    3.087
##    .cr_srh_teaseby    2.502    0.012  216.940    0.000    2.502    3.167
##    .cr_vi_hit         2.122    0.014  156.375    0.000    2.122    2.273
##    .cr_vi_privemtt    2.159    0.013  160.171    0.000    2.159    2.329
##    .cr_vi_tolerate    1.936    0.013  144.192    0.000    1.936    2.095
##
## Variances:
##                    Estimate  Std.Err  z-value  P(>|z|)   Std.lv  Std.all
##    .cr_edu_boysch     0.442    0.011   39.043    0.000    0.442    0.636
##    .cr_edu_science    0.333    0.010   34.236    0.000    0.333    0.705
##    .cr_edu_grlnhlp    0.419    0.011   37.939    0.000    0.419    0.646
##    .cr_edu_rasngvc    0.623    0.012   52.024    0.000    0.623    0.707
##    .cr_edu_boysout    0.445    0.011   40.094    0.000    0.445    0.775
##    .cr_ta_womanrol    0.597    0.012   51.539    0.000    0.597    0.735
##    .cr_ta_eemandcd    0.604    0.012   51.425    0.000    0.604    0.738
##    .cr_srh_teasgrl    0.535    0.012   45.042    0.000    0.535    0.828
```

```
##    .cr_srh_teaseby    0.510    0.012   44.085    0.000    0.510    0.817
##    .cr_vi_hit         0.634    0.013   50.259    0.000    0.634    0.727
##    .cr_vi_privemtt    0.669    0.012   56.134    0.000    0.669    0.778
##    .cr_vi_tolerate    0.643    0.012   54.393    0.000    0.643    0.753
##     GenderAttituds    0.253    0.012   20.900    0.000    1.000    1.000
##
## R-Square:
##                    Estimate
##     cr_edu_boysch     0.364
##     cr_edu_science    0.295
##     cr_edu_grlnhlp    0.354
##     cr_edu_rasngvc    0.293
##     cr_edu_boysout    0.225
##     cr_ta_womanrol    0.265
##     cr_ta_eemandcd    0.262
##     cr_srh_teasgrl    0.172
##     cr_srh_teaseby    0.183
##     cr_vi_hit         0.273
##     cr_vi_privemtt    0.222
##     cr_vi_tolerate    0.247
```

```
#FL>0.7 / 0.6
#TLI, CFI >0.9
#RMSEA, SRMR <0.08
```

```
# try out by factor: definitely better results! perfect model fits, and no variable to
remove
MMCFA1a <- '
#Factor loadings
F1 =~ cr_edu_boysch+ cr_edu_science+ cr_edu_girlnohelp+cr_edu_boysout
F2 =~ cr_vi_hit+ cr_vi_privematt+ cr_vi_tolerate
F3 =~cr_srh_teasegirl+ cr_srh_teaseboy
F4 =~cr_ta_womanrole+ cr_ta_eemandecid+cr_edu_raisingvoice
F5 =~cr_edu_boysfeelings+ cr_edu_boystough+ cr_edu_boysdefend+ cr_edu_equaltreat
F6 =~cr_edu_teachpunish+ cr_edu_teachnotpunish
F7 =~cr_fi_girlchance+ cr_fi_girlsavings

#for oblique correlation between the factors
F1 ~~ F2
F2 ~~ F3
F3 ~~ F4
F4 ~~ F5
F5 ~~ F6
F6 ~~ F7
'

#Estimator MLR because the variables are not normally distributed
fit.MMCFA1a <- cfa(MMCFA1a, data=data_ET20_subset_GNS_CFA, estimator="MLR", mimic="Mplu
s")
```

```
## Warning: lavaan->lav_data_full():
##    some cases are empty and will be ignored: 83 85 87 126 141 279 327 589 592
##    909 921 2273 2771 2772 2773 3758 4324.
```

```
summary(fit.MMCFA1a, fit.measures=TRUE, standardized=TRUE, rsq=TRUE)
```

```
## lavaan 0.6-21 ended normally after 95 iterations
##
##   Estimator                                         ML
##   Optimization method                           NLMINB
##   Number of model parameters                        81
##
##                                                   Used       Total
##   Number of observations                          4760        4777
##   Number of missing patterns                        79
##
## Model Test User Model:
##                                               Standard      Scaled
##   Test Statistic                               813.975     746.123
##   Degrees of freedom                               149         149
##   P-value (Chi-square)                           0.000       0.000
##   Scaling correction factor                                  1.091
##     Yuan-Bentler correction (Mplus variant)
##
## Model Test Baseline Model:
##
##   Test statistic                             16550.414   14719.355
##   Degrees of freedom                               190         190
##   P-value                                        0.000       0.000
##   Scaling correction factor                                  1.124
##
## User Model versus Baseline Model:
##
##   Comparative Fit Index (CFI)                    0.959       0.959
##   Tucker-Lewis Index (TLI)                       0.948       0.948
##
##   Robust Comparative Fit Index (CFI)                         0.960
##   Robust Tucker-Lewis Index (TLI)                            0.949
##
## Loglikelihood and Information Criteria:
##
##   Loglikelihood user model (H0)            -104636.713 -104636.713
##   Scaling correction factor                                  1.094
##       for the MLR correction
##   Loglikelihood unrestricted model (H1)             NA          NA
##   Scaling correction factor                                  1.092
##       for the MLR correction
##
##   Akaike (AIC)                              209435.425  209435.425
##   Bayesian (BIC)                            209959.333  209959.333
##   Sample-size adjusted Bayesian (SABIC)     209701.945  209701.945
##
## Root Mean Square Error of Approximation:
##
##   RMSEA                                          0.031       0.029
##   90 Percent confidence interval - lower         0.029       0.027
##   90 Percent confidence interval - upper         0.033       0.031
##   P-value H_0: RMSEA <= 0.050                    1.000       1.000
##   P-value H_0: RMSEA >= 0.080                    0.000       0.000
##
##   Robust RMSEA                                               0.030
```

```
##   90 Percent confidence interval - lower                     0.028
##   90 Percent confidence interval - upper                     0.033
##   P-value H_0: Robust RMSEA <= 0.050                         1.000
##   P-value H_0: Robust RMSEA >= 0.080                         0.000
##
## Standardized Root Mean Square Residual:
##
##   SRMR                                         0.032       0.032
##
## Parameter Estimates:
##
##   Standard errors                            Sandwich
##   Information bread                          Observed
##   Observed information based on               Hessian
##
## Latent Variables:
##                    Estimate  Std.Err  z-value  P(>|z|)   Std.lv  Std.all
##   F1 =~
##     cr_edu_boysch     1.000                               0.559    0.670
##     cr_edu_science    0.775    0.024   32.019    0.000    0.433    0.629
##     cr_edu_grlnhlp    0.973    0.028   34.998    0.000    0.544    0.675
##     cr_edu_boysout    0.727    0.027   26.709    0.000    0.406    0.536
##   F2 =~
##     cr_vi_hit         1.000                               0.599    0.642
##     cr_vi_privemtt    0.872    0.027   32.473    0.000    0.522    0.563
##     cr_vi_tolerate    0.957    0.030   32.174    0.000    0.573    0.620
##   F3 =~
##     cr_srh_teasgrl    1.000                               0.605    0.753
##     cr_srh_teaseby    1.003    0.041   24.559    0.000    0.607    0.768
##   F4 =~
##     cr_ta_womanrol    1.000                               0.558    0.619
##     cr_ta_eemandcd    1.002    0.029   34.986    0.000    0.559    0.618
##     cr_edu_rasngvc    1.058    0.030   35.675    0.000    0.590    0.629
##   F5 =~
##     cr_ed_bysflngs    1.000                               0.279    0.415
##     cr_edu_boystgh    1.257    0.079   15.880    0.000    0.351    0.478
##     cr_edu_bysdfnd    1.492    0.102   14.674    0.000    0.417    0.498
##     cr_edu_equltrt    1.376    0.099   13.870    0.000    0.384    0.481
##   F6 =~
##     cr_edu_tchpnsh    1.000                               0.531    0.667
##     cr_d_tchntpnsh    0.698    0.053   13.251    0.000    0.371    0.431
##   F7 =~
##     cr_fi_girlchnc    1.000                               0.353    0.529
##     cr_fi_grlsvngs    0.787    0.077   10.214    0.000    0.278    0.573
##
## Covariances:
##                    Estimate  Std.Err  z-value  P(>|z|)   Std.lv  Std.all
##   F1 ~~
##     F2                0.193    0.008   23.166    0.000    0.576    0.576
##   F2 ~~
##     F3                0.134    0.009   15.046    0.000    0.370    0.370
##   F3 ~~
##     F4                0.114    0.007   15.685    0.000    0.339    0.339
##   F4 ~~
##     F5                0.064    0.004   16.234    0.000    0.411    0.411
##   F5 ~~
```

```
##     F6                0.076    0.006   12.596    0.000    0.510    0.510
##   F6 ~~
##     F7               -0.024    0.006   -4.031    0.000   -0.127   -0.127
##   F1 ~~
##     F3                0.165    0.009   19.039    0.000    0.488    0.488
##     F4                0.195    0.008   25.137    0.000    0.625    0.625
##     F5                0.016    0.004    4.657    0.000    0.105    0.105
##     F6                0.064    0.007    9.263    0.000    0.216    0.216
##     F7                0.084    0.008   10.481    0.000    0.427    0.427
##   F2 ~~
##     F4                0.252    0.009   28.404    0.000    0.755    0.755
##     F5                0.058    0.004   13.497    0.000    0.348    0.348
##     F6                0.105    0.009   12.287    0.000    0.331    0.331
##     F7                0.019    0.006    2.980    0.003    0.090    0.090
##   F3 ~~
##     F5                0.018    0.004    4.508    0.000    0.105    0.105
##     F6                0.045    0.007    6.150    0.000    0.140    0.140
##     F7                0.033    0.005    6.195    0.000    0.157    0.157
##   F4 ~~
##     F6                0.133    0.009   14.837    0.000    0.450    0.450
##     F7                0.035    0.008    4.628    0.000    0.180    0.180
##   F5 ~~
##     F7               -0.033    0.005   -7.307    0.000   -0.335   -0.335
##
## Intercepts:
##                    Estimate  Std.Err  z-value  P(>|z|)   Std.lv  Std.all
##    .cr_edu_boysch     2.452    0.012  202.426    0.000    2.452    2.940
##    .cr_edu_science    2.675    0.010  266.675    0.000    2.675    3.888
##    .cr_edu_grlnhlp    2.508    0.012  214.283    0.000    2.508    3.112
##    .cr_edu_boysout    2.571    0.011  233.834    0.000    2.571    3.394
##    .cr_vi_hit         2.122    0.014  156.381    0.000    2.122    2.273
##    .cr_vi_privemtt    2.159    0.013  160.129    0.000    2.159    2.329
##    .cr_vi_tolerate    1.936    0.013  144.150    0.000    1.936    2.095
##    .cr_srh_teasgrl    2.481    0.012  211.548    0.000    2.481    3.087
##    .cr_srh_teaseby    2.502    0.012  217.126    0.000    2.502    3.168
##    .cr_ta_womanrol    1.755    0.013  134.320    0.000    1.755    1.948
##    .cr_ta_eemandcd    1.784    0.013  135.986    0.000    1.784    1.972
##    .cr_edu_rasngvc    2.008    0.014  147.443    0.000    2.008    2.139
##    .cr_ed_bysflngs    1.351    0.010  138.169    0.000    1.351    2.008
##    .cr_edu_boystgh    1.456    0.011  136.638    0.000    1.456    1.984
##    .cr_edu_bysdfnd    1.635    0.012  134.338    0.000    1.635    1.951
##    .cr_edu_equltrt    1.550    0.012  133.573    0.000    1.550    1.939
##    .cr_edu_tchpnsh    1.530    0.012  132.436    0.000    1.530    1.925
##    .cr_d_tchntpnsh    1.659    0.012  132.761    0.000    1.659    1.931
##    .cr_fi_girlchnc    2.673    0.010  274.710    0.000    2.673    4.001
##    .cr_fi_grlsvngs    2.825    0.007  396.497    0.000    2.825    5.823
##
## Variances:
##                    Estimate  Std.Err  z-value  P(>|z|)   Std.lv  Std.all
##    .cr_edu_boysch     0.383    0.012   30.783    0.000    0.383    0.551
##    .cr_edu_science    0.286    0.010   29.715    0.000    0.286    0.604
##    .cr_edu_grlnhlp    0.354    0.012   30.081    0.000    0.354    0.545
##    .cr_edu_boysout    0.409    0.012   35.333    0.000    0.409    0.712
##    .cr_vi_hit         0.513    0.015   34.609    0.000    0.513    0.588
##    .cr_vi_privemtt    0.587    0.014   42.355    0.000    0.587    0.683
##    .cr_vi_tolerate    0.526    0.014   37.147    0.000    0.526    0.615
```

```
##    .cr_srh_teasgrl    0.280    0.017   16.465    0.000    0.280    0.434
##    .cr_srh_teaseby    0.256    0.017   15.159    0.000    0.256    0.410
##    .cr_ta_womanrol    0.501    0.013   37.431    0.000    0.501    0.616
##    .cr_ta_eemandcd    0.506    0.013   37.709    0.000    0.506    0.618
##    .cr_edu_rasngvc    0.533    0.014   38.777    0.000    0.533    0.605
##    .cr_ed_bysflngs    0.375    0.012   32.115    0.000    0.375    0.828
##    .cr_edu_boystgh    0.416    0.012   33.903    0.000    0.416    0.771
##    .cr_edu_bysdfnd    0.528    0.015   36.416    0.000    0.528    0.752
##    .cr_edu_equltrt    0.491    0.014   35.800    0.000    0.491    0.769
##    .cr_edu_tchpnsh    0.351    0.023   14.967    0.000    0.351    0.555
##    .cr_d_tchntpnsh    0.601    0.015   39.009    0.000    0.601    0.814
##    .cr_fi_girlchnc    0.322    0.016   20.260    0.000    0.322    0.720
##    .cr_fi_grlsvngs    0.158    0.010   16.330    0.000    0.158    0.671
##     F1                0.312    0.013   23.310    0.000    1.000    1.000
##     F2                0.359    0.015   23.866    0.000    1.000    1.000
##     F3                0.366    0.018   19.797    0.000    1.000    1.000
##     F4                0.311    0.014   22.557    0.000    1.000    1.000
##     F5                0.078    0.009    8.790    0.000    1.000    1.000
##     F6                0.282    0.024   11.620    0.000    1.000    1.000
##     F7                0.125    0.014    8.652    0.000    1.000    1.000
##
## R-Square:
##                    Estimate
##     cr_edu_boysch     0.449
##     cr_edu_science    0.396
##     cr_edu_grlnhlp    0.455
##     cr_edu_boysout    0.288
##     cr_vi_hit         0.412
##     cr_vi_privemtt    0.317
##     cr_vi_tolerate    0.385
##     cr_srh_teasgrl    0.566
##     cr_srh_teaseby    0.590
##     cr_ta_womanrol    0.384
##     cr_ta_eemandcd    0.382
##     cr_edu_rasngvc    0.395
##     cr_ed_bysflngs    0.172
##     cr_edu_boystgh    0.229
##     cr_edu_bysdfnd    0.248
##     cr_edu_equltrt    0.231
##     cr_edu_tchpnsh    0.445
##     cr_d_tchntpnsh    0.186
##     cr_fi_girlchnc    0.280
##     cr_fi_grlsvngs    0.329
```

```
#FL>0.7 / 0.6
#TLI, CFI >0.9
#RMSEA, SRMR <0.08
```

5.3 Create dummy variables

6 Structural Equation Modelling

6.1 Preparation

6.1.1 Multivariate normality test

```
subsetCor = data_ET20_final %>%
dplyr::select(c(cr_ghq12,cr_copingresilience, cr_genderedactivities, cr_talk , cr_media
, cr_socialmedia , cr_voice , cr_gendernormscale_f1, cr_gendernormscale_f2, cr_genderno
rmscale_f3, cr_gendernormscale_f4, cr_gendernormscale_f5, cr_gendernormscale_f6, cr_gen
dernormscale_f7, cr_cs_crage, af_asset_10, peervio_ordinal))
subsetCor <- na.omit(subsetCor)
```

```
mnormal<- mvn(subsetCor)
mnormal
```

```
## $multivariate_normality
##            Test Statistic p.value     Method          MVN
## 1 Henze-Zirkler     1.141  <0.001 asymptotic ✗ Not normal
##
## $univariate_normality
##                Test              Variable Statistic p.value    Normality
## 1  Anderson-Darling              cr_ghq12    58.258  <0.001 ✗ Not normal
## 2  Anderson-Darling   cr_copingresilience    58.984  <0.001 ✗ Not normal
## 3  Anderson-Darling cr_genderedactivities    84.104  <0.001 ✗ Not normal
## 4  Anderson-Darling               cr_talk    77.020  <0.001 ✗ Not normal
## 5  Anderson-Darling              cr_media    94.671  <0.001 ✗ Not normal
## 6  Anderson-Darling        cr_socialmedia   441.199  <0.001 ✗ Not normal
## 7  Anderson-Darling              cr_voice     4.710  <0.001 ✗ Not normal
## 8  Anderson-Darling cr_gendernormscale_f1   122.810  <0.001 ✗ Not normal
## 9  Anderson-Darling cr_gendernormscale_f2    52.462  <0.001 ✗ Not normal
## 10 Anderson-Darling cr_gendernormscale_f3   187.940  <0.001 ✗ Not normal
## 11 Anderson-Darling cr_gendernormscale_f4    56.281  <0.001 ✗ Not normal
## 12 Anderson-Darling cr_gendernormscale_f5    71.525  <0.001 ✗ Not normal
## 13 Anderson-Darling cr_gendernormscale_f6   125.689  <0.001 ✗ Not normal
## 14 Anderson-Darling cr_gendernormscale_f7   287.524  <0.001 ✗ Not normal
## 15 Anderson-Darling           cr_cs_crage   105.727  <0.001 ✗ Not normal
## 16 Anderson-Darling           af_asset_10    29.094  <0.001 ✗ Not normal
## 17 Anderson-Darling       peervio_ordinal   251.974  <0.001 ✗ Not normal
##
## $descriptives
##                 Variable    n   Mean Std.Dev Median Min Max 25th 75th   Skew
## 1               cr_ghq12 1623  7.099   3.884      6   0  36    5    8  1.476
## 2    cr_copingresilience 1623 19.980   4.099     21   0  24   18   23 -1.604
## 3  cr_genderedactivities 1623  1.988   2.204      2   0  13    0    3  1.148
## 4                cr_talk 1623  2.624   1.192      3   0   4    2    4 -0.715
## 5               cr_media 1623  2.644   1.652      2   1   6    1    4  0.503
## 6         cr_socialmedia 1623  1.262   0.756      1   1   6    1    1  3.379
## 7               cr_voice 1623 18.688   5.052     18   8  32   15   22  0.188
## 8  cr_gendernormscale_f1 1623 10.140   2.252     11   4  12    8   12 -1.052
## 9  cr_gendernormscale_f2 1623  6.250   2.109      7   3   9    5    8 -0.127
## 10 cr_gendernormscale_f3 1623  4.912   1.446      6   2   6    4    6 -0.974
## 11 cr_gendernormscale_f4 1623  5.356   1.983      5   3   9    3    7  0.388
## 12 cr_gendernormscale_f5 1623  5.907   1.896      6   4  12    4    7  0.846
## 13 cr_gendernormscale_f6 1623  3.190   1.320      3   2   6    2    4  0.739
## 14 cr_gendernormscale_f7 1623  5.445   0.986      6   2   6    5    6 -1.726
## 15           cr_cs_crage 1623 13.010   0.932     13   9  14   12   14 -0.743
## 16           af_asset_10 1623  5.133   2.859      5   1  10    3    8  0.164
## 17       peervio_ordinal 1623  0.930   1.780      0   0  12    0    1  2.665
##    Kurtosis
## 1     7.903
## 2     6.505
## 3     4.190
## 4     2.688
## 5     1.909
## 6    15.407
## 7     2.557
## 8     3.097
## 9     1.754
## 10    2.506
## 11    2.009
```

```
## 12    3.092
## 13    2.402
## 14    5.230
## 15    3.758
## 16    1.819
## 17   11.740
##
## $data
## # A tibble: 1,623 × 17
##    cr_ghq12 cr_copingresilience cr_genderedactivities cr_talk cr_media
##       <dbl>               <dbl>                 <dbl>   <dbl>    <dbl>
##  1        1                  16                     3       3        1
##  2       18                  16                     2       4        3
##  3        2                  24                     1       3        2
##  4        2                  20                     1       4        1
##  5        6                  13                     2       1        2
##  6        5                  20                     3       4        1
##  7        2                  18                     3       4        4
##  8        2                  22                     2       2        1
##  9        7                  22                     5       3        1
## 10        1                  24                     3       3        1
## # ℹ 1,613 more rows
## # ℹ 12 more variables: cr_socialmedia <dbl>, cr_voice <dbl>,
## #   cr_gendernormscale_f1 <dbl>, cr_gendernormscale_f2 <dbl>,
## #   cr_gendernormscale_f3 <dbl>, cr_gendernormscale_f4 <dbl>,
## #   cr_gendernormscale_f5 <dbl>, cr_gendernormscale_f6 <dbl>,
## #   cr_gendernormscale_f7 <dbl>, cr_cs_crage <dbl>, af_asset_10 <labelled>,
## #   peervio_ordinal <dbl>
##
## $subset
## NULL
##
## $outlierMethod
## [1] "none"
##
## attr(,"class")
## [1] "mvn"
```

```
#all items are not normally distributed ->MLR

#for item level correlation matrix
cmat=round(cor(subsetCor),2)
cmat[upper.tri(cmat)] <- ""
cmat<-as.data.frame(cmat)
cmat
```

```
##                         cr_ghq12 cr_copingresilience cr_genderedactivities
## cr_ghq12                       1
## cr_copingresilience        -0.25                   1
## cr_genderedactivities       0.04               -0.31                     1
## cr_talk                    -0.08                0.41                 -0.28
## cr_media                   -0.08                0.28                 -0.47
## cr_socialmedia             -0.06                0.14                 -0.22
## cr_voice                   -0.04                 0.2                 -0.09
## cr_gendernormscale_f1      -0.15                0.28                  -0.3
## cr_gendernormscale_f2      -0.09                0.15                 -0.25
## cr_gendernormscale_f3      -0.12                0.16                 -0.21
## cr_gendernormscale_f4      -0.02                0.23                 -0.29
## cr_gendernormscale_f5       0.01               -0.03                  0.04
## cr_gendernormscale_f6       0.01                   0                 -0.03
## cr_gendernormscale_f7      -0.12                0.15                 -0.15
## cr_cs_crage                 0.03                0.08                     0
## af_asset_10                -0.14                0.29                 -0.42
## peervio_ordinal             0.21                   0                 -0.17
##                         cr_talk cr_media cr_socialmedia cr_voice
## cr_ghq12
## cr_copingresilience
## cr_genderedactivities
## cr_talk                       1
## cr_media                   0.26        1
## cr_socialmedia             0.14     0.34              1
## cr_voice                   0.38     0.07           0.07        1
## cr_gendernormscale_f1      0.31     0.31           0.17     0.13
## cr_gendernormscale_f2      0.19     0.26           0.18     0.01
## cr_gendernormscale_f3      0.15     0.16            0.1     0.09
## cr_gendernormscale_f4      0.25     0.35           0.24     0.05
## cr_gendernormscale_f5     -0.04    -0.02           0.04    -0.02
## cr_gendernormscale_f6         0     0.04           0.03    -0.01
## cr_gendernormscale_f7      0.21     0.19            0.1     0.14
## cr_cs_crage                0.13     0.08           0.07     0.08
## af_asset_10                0.24     0.53           0.32     0.04
## peervio_ordinal            0.13     0.13           0.06     0.07
##                         cr_gendernormscale_f1 cr_gendernormscale_f2
## cr_ghq12
## cr_copingresilience
## cr_genderedactivities
## cr_talk
## cr_media
## cr_socialmedia
## cr_voice
## cr_gendernormscale_f1                       1
## cr_gendernormscale_f2                    0.37                     1
## cr_gendernormscale_f3                    0.36                  0.26
## cr_gendernormscale_f4                    0.38                  0.44
## cr_gendernormscale_f5                    0.01                  0.19
## cr_gendernormscale_f6                    0.08                   0.1
## cr_gendernormscale_f7                    0.25                  0.07
## cr_cs_crage                              0.12                  0.01
## af_asset_10                              0.31                  0.28
## peervio_ordinal                          0.06                  0.03
##                         cr_gendernormscale_f3 cr_gendernormscale_f4
```

```
## cr_ghq12
## cr_copingresilience
## cr_genderedactivities
## cr_talk
## cr_media
## cr_socialmedia
## cr_voice
## cr_gendernormscale_f1
## cr_gendernormscale_f2
## cr_gendernormscale_f3                      1
## cr_gendernormscale_f4                   0.23                     1
## cr_gendernormscale_f5                   0.07                   0.2
## cr_gendernormscale_f6                   0.09                  0.15
## cr_gendernormscale_f7                   0.11                  0.12
## cr_cs_crage                             0.07                  0.07
## af_asset_10                             0.15                  0.38
## peervio_ordinal                         0.02                  0.07
##                       cr_gendernormscale_f5 cr_gendernormscale_f6
## cr_ghq12
## cr_copingresilience
## cr_genderedactivities
## cr_talk
## cr_media
## cr_socialmedia
## cr_voice
## cr_gendernormscale_f1
## cr_gendernormscale_f2
## cr_gendernormscale_f3
## cr_gendernormscale_f4
## cr_gendernormscale_f5                      1
## cr_gendernormscale_f6                   0.23                     1
## cr_gendernormscale_f7                  -0.13                 -0.04
## cr_cs_crage                            -0.04                  0.03
## af_asset_10                             0.01                  0.07
## peervio_ordinal                        -0.03                  0.01
##                       cr_gendernormscale_f7 cr_cs_crage af_asset_10
## cr_ghq12
## cr_copingresilience
## cr_genderedactivities
## cr_talk
## cr_media
## cr_socialmedia
## cr_voice
## cr_gendernormscale_f1
## cr_gendernormscale_f2
## cr_gendernormscale_f3
## cr_gendernormscale_f4
## cr_gendernormscale_f5
## cr_gendernormscale_f6
## cr_gendernormscale_f7                      1
## cr_cs_crage                             0.11           1
## af_asset_10                              0.2         0.1           1
## peervio_ordinal                         0.02       -0.02        0.06
##                       peervio_ordinal
## cr_ghq12
## cr_copingresilience
```

```
## cr_genderedactivities
## cr_talk
## cr_media
## cr_socialmedia
## cr_voice
## cr_gendernormscale_f1
## cr_gendernormscale_f2
## cr_gendernormscale_f3
## cr_gendernormscale_f4
## cr_gendernormscale_f5
## cr_gendernormscale_f6
## cr_gendernormscale_f7
## cr_cs_crage
## af_asset_10
## peervio_ordinal                     1
```

6.1.2 Correlation / Covariance Matrix

```
#correlation matrix and covariance matrix
cormatrix <- cor(subsetCor)
cormatrix
```

```
##                               cr_ghq12 cr_copingresilience cr_genderedactivities
## cr_ghq12                1.000000000000     -0.254730071743        0.035077186701
## cr_copingresilience    -0.254730071743      1.000000000000       -0.313524215147
## cr_genderedactivities   0.035077186701     -0.313524215147        1.000000000000
## cr_talk                -0.075843846326      0.405499750433       -0.276960283162
## cr_media               -0.083283093781      0.277364662124       -0.467160736210
## cr_socialmedia         -0.056075206749      0.135529530507       -0.217754914974
## cr_voice               -0.040831738092      0.195043845867       -0.087125967912
## cr_gendernormscale_f1 -0.154200837381      0.284921525874       -0.296868371657
## cr_gendernormscale_f2 -0.090393474177      0.151398997361       -0.248709900476
## cr_gendernormscale_f3 -0.115689734275      0.155741862251       -0.206634564727
## cr_gendernormscale_f4 -0.024027893625      0.225364776516       -0.292572664349
## cr_gendernormscale_f5  0.005104663189     -0.031259799161        0.036616741629
## cr_gendernormscale_f6  0.010623100253      0.003564419895       -0.028231484017
## cr_gendernormscale_f7 -0.115843074062      0.146697471701       -0.153547762209
## cr_cs_crage             0.028180473928      0.076574040633       -0.002943937606
## af_asset_10            -0.138074727765      0.290165034641       -0.415798540965
## peervio_ordinal         0.205414487930     -0.001462892801       -0.171975352771
##                                 cr_talk       cr_media cr_socialmedia
## cr_ghq12               -0.0758438463261 -0.08328309378 -0.05607520675
## cr_copingresilience     0.4054997504326  0.27736466212  0.13552953051
## cr_genderedactivities -0.2769602831616 -0.46716073621 -0.21775491497
## cr_talk                 1.0000000000000  0.26366624626  0.13841749545
## cr_media                0.2636662462592  1.00000000000  0.33572154706
## cr_socialmedia          0.1384174954486  0.33572154706  1.00000000000
## cr_voice                0.3795529878886  0.07178831243  0.06744260710
## cr_gendernormscale_f1   0.3080980572948  0.30698306204  0.17451345238
## cr_gendernormscale_f2   0.1861426861401  0.25663082921  0.18176924007
## cr_gendernormscale_f3   0.1482188721644  0.15932137260  0.09782330476
## cr_gendernormscale_f4   0.2452890514871  0.35494982192  0.24391293399
## cr_gendernormscale_f5 -0.0438930027844 -0.02336245212  0.03852883587
## cr_gendernormscale_f6 -0.0006557027435  0.03756437516  0.02710161090
## cr_gendernormscale_f7   0.2108538404222  0.18612053125  0.09629137140
## cr_cs_crage             0.1271913905309  0.07999746693  0.06718449770
## af_asset_10             0.2405419384258  0.53266338719  0.31590010381
## peervio_ordinal         0.1278862572832  0.13048314242  0.05626624733
##                               cr_voice cr_gendernormscale_f1
## cr_ghq12               -0.040831738092        -0.15420083738
## cr_copingresilience     0.195043845867         0.28492152587
## cr_genderedactivities -0.087125967912        -0.29686837166
## cr_talk                 0.379552987889         0.30809805729
## cr_media                0.071788312427         0.30698306204
## cr_socialmedia          0.067442607101         0.17451345238
## cr_voice                1.000000000000         0.13479402517
## cr_gendernormscale_f1   0.134794025174         1.00000000000
## cr_gendernormscale_f2   0.012486569116         0.37461817658
## cr_gendernormscale_f3   0.085615011946         0.36040662449
## cr_gendernormscale_f4   0.050654760427         0.37978247706
## cr_gendernormscale_f5 -0.017581663133         0.01057243857
## cr_gendernormscale_f6 -0.008179168427         0.08309249731
## cr_gendernormscale_f7   0.141656935441         0.25008511907
## cr_cs_crage             0.080031225715         0.12159694454
## af_asset_10             0.044021315196         0.31414320140
## peervio_ordinal         0.067467906418         0.05874537501
##                        cr_gendernormscale_f2 cr_gendernormscale_f3
```

```
## cr_ghq12                   -0.090393474177     -0.11568973427
## cr_copingresilience         0.151398997361      0.15574186225
## cr_genderedactivities      -0.248709900476     -0.20663456473
## cr_talk                     0.186142686140      0.14821887216
## cr_media                    0.256630829215      0.15932137260
## cr_socialmedia              0.181769240066      0.09782330476
## cr_voice                    0.012486569116      0.08561501195
## cr_gendernormscale_f1       0.374618176581      0.36040662449
## cr_gendernormscale_f2       1.000000000000      0.25529248188
## cr_gendernormscale_f3       0.255292481878      1.00000000000
## cr_gendernormscale_f4       0.435894244643      0.22680605713
## cr_gendernormscale_f5       0.189580607748      0.07100245326
## cr_gendernormscale_f6       0.099578283729      0.08762053191
## cr_gendernormscale_f7       0.066223119399      0.10709270863
## cr_cs_crage                 0.008470044532      0.06747201255
## af_asset_10                 0.284379457424      0.15137763012
## peervio_ordinal             0.027500031337      0.01795200987
##                       cr_gendernormscale_f4 cr_gendernormscale_f5
## cr_ghq12                     -0.02402789363       0.005104663189
## cr_copingresilience           0.22536477652      -0.031259799161
## cr_genderedactivities        -0.29257266435       0.036616741629
## cr_talk                       0.24528905149      -0.043893002784
## cr_media                      0.35494982192      -0.023362452119
## cr_socialmedia                0.24391293399       0.038528835875
## cr_voice                      0.05065476043      -0.017581663133
## cr_gendernormscale_f1         0.37978247706       0.010572438566
## cr_gendernormscale_f2         0.43589424464       0.189580607748
## cr_gendernormscale_f3         0.22680605713       0.071002453258
## cr_gendernormscale_f4         1.00000000000       0.202104385085
## cr_gendernormscale_f5         0.20210438508       1.000000000000
## cr_gendernormscale_f6         0.14557476062       0.230756639514
## cr_gendernormscale_f7         0.12432287477      -0.134168204114
## cr_cs_crage                   0.07251041560      -0.044853355906
## af_asset_10                   0.38444991900       0.014908290282
## peervio_ordinal               0.06731085123      -0.027870001188
##                       cr_gendernormscale_f6 cr_gendernormscale_f7
## cr_ghq12                    0.0106231002526        -0.11584307406
## cr_copingresilience         0.0035644198949         0.14669747170
## cr_genderedactivities      -0.0282314840170        -0.15354776221
## cr_talk                    -0.0006557027435         0.21085384042
## cr_media                    0.0375643751592         0.18612053125
## cr_socialmedia              0.0271016108996         0.09629137140
## cr_voice                   -0.0081791684266         0.14165693544
## cr_gendernormscale_f1       0.0830924973141         0.25008511907
## cr_gendernormscale_f2       0.0995782837295         0.06622311940
## cr_gendernormscale_f3       0.0876205319104         0.10709270863
## cr_gendernormscale_f4       0.1455747606205         0.12432287477
## cr_gendernormscale_f5       0.2307566395142        -0.13416820411
## cr_gendernormscale_f6       1.0000000000000        -0.04332543905
## cr_gendernormscale_f7      -0.0433254390514         1.00000000000
## cr_cs_crage                 0.0275499933860         0.11401995541
## af_asset_10                 0.0731587896728         0.19745243059
## peervio_ordinal             0.0093660196034         0.02448278478
##                           cr_cs_crage    af_asset_10 peervio_ordinal
## cr_ghq12               0.028180473928 -0.13807472777  0.205414487930
## cr_copingresilience    0.076574040633  0.29016503464 -0.001462892801
```

```
## cr_genderedactivities -0.002943937606 -0.41579854097 -0.171975352771
## cr_talk                0.127191390531  0.24054193843  0.127886257283
## cr_media               0.079997466928  0.53266338719  0.130483142417
## cr_socialmedia         0.067184497703  0.31590010381  0.056266247327
## cr_voice               0.080031225715  0.04402131520  0.067467906418
## cr_gendernormscale_f1  0.121596944536  0.31414320140  0.058745375009
## cr_gendernormscale_f2  0.008470044532  0.28437945742  0.027500031337
## cr_gendernormscale_f3  0.067472012553  0.15137763012  0.017952009865
## cr_gendernormscale_f4  0.072510415600  0.38444991900  0.067310851232
## cr_gendernormscale_f5 -0.044853355906  0.01490829028 -0.027870001188
## cr_gendernormscale_f6  0.027549993386  0.07315878967  0.009366019603
## cr_gendernormscale_f7  0.114019955413  0.19745243059  0.024482784783
## cr_cs_crage            1.000000000000  0.09925605308 -0.015564102333
## af_asset_10            0.099256053079  1.00000000000  0.060691616929
## peervio_ordinal       -0.015564102333  0.06069161693  1.000000000000
```

```
cormat=round(cor(subsetCor),2)
cormat[upper.tri(cormat)] <- ""
cormat<-as.data.frame(cormat)
cormat
```

```
##                         cr_ghq12 cr_copingresilience cr_genderedactivities
## cr_ghq12                       1
## cr_copingresilience        -0.25                   1
## cr_genderedactivities       0.04               -0.31                     1
## cr_talk                    -0.08                0.41                 -0.28
## cr_media                   -0.08                0.28                 -0.47
## cr_socialmedia             -0.06                0.14                 -0.22
## cr_voice                   -0.04                 0.2                 -0.09
## cr_gendernormscale_f1      -0.15                0.28                  -0.3
## cr_gendernormscale_f2      -0.09                0.15                 -0.25
## cr_gendernormscale_f3      -0.12                0.16                 -0.21
## cr_gendernormscale_f4      -0.02                0.23                 -0.29
## cr_gendernormscale_f5       0.01               -0.03                  0.04
## cr_gendernormscale_f6       0.01                   0                 -0.03
## cr_gendernormscale_f7      -0.12                0.15                 -0.15
## cr_cs_crage                 0.03                0.08                     0
## af_asset_10                -0.14                0.29                 -0.42
## peervio_ordinal             0.21                   0                 -0.17
##                         cr_talk cr_media cr_socialmedia cr_voice
## cr_ghq12
## cr_copingresilience
## cr_genderedactivities
## cr_talk                       1
## cr_media                   0.26        1
## cr_socialmedia             0.14     0.34              1
## cr_voice                   0.38     0.07           0.07        1
## cr_gendernormscale_f1      0.31     0.31           0.17     0.13
## cr_gendernormscale_f2      0.19     0.26           0.18     0.01
## cr_gendernormscale_f3      0.15     0.16            0.1     0.09
## cr_gendernormscale_f4      0.25     0.35           0.24     0.05
## cr_gendernormscale_f5     -0.04    -0.02           0.04    -0.02
## cr_gendernormscale_f6         0     0.04           0.03    -0.01
## cr_gendernormscale_f7      0.21     0.19            0.1     0.14
## cr_cs_crage                0.13     0.08           0.07     0.08
## af_asset_10                0.24     0.53           0.32     0.04
## peervio_ordinal            0.13     0.13           0.06     0.07
##                         cr_gendernormscale_f1 cr_gendernormscale_f2
## cr_ghq12
## cr_copingresilience
## cr_genderedactivities
## cr_talk
## cr_media
## cr_socialmedia
## cr_voice
## cr_gendernormscale_f1                       1
## cr_gendernormscale_f2                    0.37                     1
## cr_gendernormscale_f3                    0.36                  0.26
## cr_gendernormscale_f4                    0.38                  0.44
## cr_gendernormscale_f5                    0.01                  0.19
## cr_gendernormscale_f6                    0.08                   0.1
## cr_gendernormscale_f7                    0.25                  0.07
## cr_cs_crage                              0.12                  0.01
## af_asset_10                              0.31                  0.28
## peervio_ordinal                          0.06                  0.03
##                         cr_gendernormscale_f3 cr_gendernormscale_f4
```

```
## cr_ghq12
## cr_copingresilience
## cr_genderedactivities
## cr_talk
## cr_media
## cr_socialmedia
## cr_voice
## cr_gendernormscale_f1
## cr_gendernormscale_f2
## cr_gendernormscale_f3                     1
## cr_gendernormscale_f4                  0.23                     1
## cr_gendernormscale_f5                  0.07                   0.2
## cr_gendernormscale_f6                  0.09                  0.15
## cr_gendernormscale_f7                  0.11                  0.12
## cr_cs_crage                           0.07                  0.07
## af_asset_10                           0.15                  0.38
## peervio_ordinal                       0.02                  0.07
##                       cr_gendernormscale_f5 cr_gendernormscale_f6
## cr_ghq12
## cr_copingresilience
## cr_genderedactivities
## cr_talk
## cr_media
## cr_socialmedia
## cr_voice
## cr_gendernormscale_f1
## cr_gendernormscale_f2
## cr_gendernormscale_f3
## cr_gendernormscale_f4
## cr_gendernormscale_f5                     1
## cr_gendernormscale_f6                  0.23                     1
## cr_gendernormscale_f7                 -0.13                 -0.04
## cr_cs_crage                          -0.04                  0.03
## af_asset_10                           0.01                  0.07
## peervio_ordinal                      -0.03                  0.01
##                       cr_gendernormscale_f7 cr_cs_crage af_asset_10
## cr_ghq12
## cr_copingresilience
## cr_genderedactivities
## cr_talk
## cr_media
## cr_socialmedia
## cr_voice
## cr_gendernormscale_f1
## cr_gendernormscale_f2
## cr_gendernormscale_f3
## cr_gendernormscale_f4
## cr_gendernormscale_f5
## cr_gendernormscale_f6
## cr_gendernormscale_f7                     1
## cr_cs_crage                           0.11           1
## af_asset_10                            0.2         0.1           1
## peervio_ordinal                       0.02       -0.02        0.06
##                       peervio_ordinal
## cr_ghq12
## cr_copingresilience
```

```
## cr_genderedactivities
## cr_talk
## cr_media
## cr_socialmedia
## cr_voice
## cr_gendernormscale_f1
## cr_gendernormscale_f2
## cr_gendernormscale_f3
## cr_gendernormscale_f4
## cr_gendernormscale_f5
## cr_gendernormscale_f6
## cr_gendernormscale_f7
## cr_cs_crage
## af_asset_10
## peervio_ordinal                     1
```

```
covmat=round(cov(subsetCor),2)
covmat[upper.tri(covmat)] <- ""
covmat<-as.data.frame(covmat)
covmat
```

```
##                         cr_ghq12 cr_copingresilience cr_genderedactivities
## cr_ghq12                   15.09
## cr_copingresilience        -4.06                16.8
## cr_genderedactivities        0.3               -2.83                  4.86
## cr_talk                    -0.35                1.98                 -0.73
## cr_media                   -0.53                1.88                  -1.7
## cr_socialmedia             -0.16                0.42                 -0.36
## cr_voice                    -0.8                4.04                 -0.97
## cr_gendernormscale_f1      -1.35                2.63                 -1.47
## cr_gendernormscale_f2      -0.74                1.31                 -1.16
## cr_gendernormscale_f3      -0.65                0.92                 -0.66
## cr_gendernormscale_f4      -0.19                1.83                 -1.28
## cr_gendernormscale_f5       0.04               -0.24                  0.15
## cr_gendernormscale_f6       0.05                0.02                 -0.08
## cr_gendernormscale_f7      -0.44                0.59                 -0.33
## cr_cs_crage                  0.1                0.29                 -0.01
## af_asset_10                -1.53                 3.4                 -2.62
## peervio_ordinal             1.42               -0.01                 -0.67
##                         cr_talk cr_media cr_socialmedia cr_voice
## cr_ghq12
## cr_copingresilience
## cr_genderedactivities
## cr_talk                    1.42
## cr_media                   0.52     2.73
## cr_socialmedia             0.12     0.42           0.57
## cr_voice                   2.28      0.6           0.26    25.52
## cr_gendernormscale_f1      0.83     1.14            0.3     1.53
## cr_gendernormscale_f2      0.47     0.89           0.29     0.13
## cr_gendernormscale_f3      0.26     0.38           0.11     0.63
## cr_gendernormscale_f4      0.58     1.16           0.37     0.51
## cr_gendernormscale_f5      -0.1    -0.07           0.06    -0.17
## cr_gendernormscale_f6         0     0.08           0.03    -0.05
## cr_gendernormscale_f7      0.25      0.3           0.07     0.71
## cr_cs_crage                0.14     0.12           0.05     0.38
## af_asset_10                0.82     2.52           0.68     0.64
## peervio_ordinal            0.27     0.38           0.08     0.61
##                         cr_gendernormscale_f1 cr_gendernormscale_f2
## cr_ghq12
## cr_copingresilience
## cr_genderedactivities
## cr_talk
## cr_media
## cr_socialmedia
## cr_voice
## cr_gendernormscale_f1                    5.07
## cr_gendernormscale_f2                    1.78                  4.45
## cr_gendernormscale_f3                    1.17                  0.78
## cr_gendernormscale_f4                     1.7                  1.82
## cr_gendernormscale_f5                    0.05                  0.76
## cr_gendernormscale_f6                    0.25                  0.28
## cr_gendernormscale_f7                    0.56                  0.14
## cr_cs_crage                              0.26                  0.02
## af_asset_10                              2.02                  1.72
## peervio_ordinal                          0.24                   0.1
##                         cr_gendernormscale_f3 cr_gendernormscale_f4
```

```
## cr_ghq12
## cr_copingresilience
## cr_genderedactivities
## cr_talk
## cr_media
## cr_socialmedia
## cr_voice
## cr_gendernormscale_f1
## cr_gendernormscale_f2
## cr_gendernormscale_f3                    2.09
## cr_gendernormscale_f4                    0.65                 3.93
## cr_gendernormscale_f5                    0.19                 0.76
## cr_gendernormscale_f6                    0.17                 0.38
## cr_gendernormscale_f7                    0.15                 0.24
## cr_cs_crage                              0.09                 0.13
## af_asset_10                              0.63                 2.18
## peervio_ordinal                          0.05                 0.24
##                       cr_gendernormscale_f5 cr_gendernormscale_f6
## cr_ghq12
## cr_copingresilience
## cr_genderedactivities
## cr_talk
## cr_media
## cr_socialmedia
## cr_voice
## cr_gendernormscale_f1
## cr_gendernormscale_f2
## cr_gendernormscale_f3
## cr_gendernormscale_f4
## cr_gendernormscale_f5                    3.59
## cr_gendernormscale_f6                    0.58                 1.74
## cr_gendernormscale_f7                   -0.25                -0.06
## cr_cs_crage                             -0.08                 0.03
## af_asset_10                              0.08                 0.28
## peervio_ordinal                         -0.09                 0.02
##                       cr_gendernormscale_f7 cr_cs_crage af_asset_10
## cr_ghq12
## cr_copingresilience
## cr_genderedactivities
## cr_talk
## cr_media
## cr_socialmedia
## cr_voice
## cr_gendernormscale_f1
## cr_gendernormscale_f2
## cr_gendernormscale_f3
## cr_gendernormscale_f4
## cr_gendernormscale_f5
## cr_gendernormscale_f6
## cr_gendernormscale_f7                    0.97
## cr_cs_crage                               0.1        0.87
## af_asset_10                              0.56        0.26        8.18
## peervio_ordinal                          0.04       -0.03        0.31
##                       peervio_ordinal
## cr_ghq12
## cr_copingresilience
```

```
## cr_genderedactivities
## cr_talk
## cr_media
## cr_socialmedia
## cr_voice
## cr_gendernormscale_f1
## cr_gendernormscale_f2
## cr_gendernormscale_f3
## cr_gendernormscale_f4
## cr_gendernormscale_f5
## cr_gendernormscale_f6
## cr_gendernormscale_f7
## cr_cs_crage
## af_asset_10
## peervio_ordinal                  3.17
```

```
#eigenvalues of correlation matrix
subsetCor <- cor(subsetCor)  # first calculate correlation matrix
eigenvalues <- eigen(subsetCor)$values

# all eigenvalues > 0?
min(eigenvalues)
```

```
## [1] 0.4516253944
```

```
#0.48 > 0 ->Korrelationsmatrix positiv definit und daher invertierbar
```

```
#spearman correlations for continuous and ordinal variables
spearman_corr <- cor(subsetCor, method = "spearman")
print(spearman_corr)
```

```
##                              cr_ghq12 cr_copingresilience cr_genderedactivities
## cr_ghq12                1.00000000000      -0.80637254902          0.7352941176
## cr_copingresilience    -0.80637254902       1.00000000000         -0.8774509804
## cr_genderedactivities   0.73529411765      -0.87745098039          1.0000000000
## cr_talk                -0.71323529412       0.94117647059         -0.7794117647
## cr_media               -0.67156862745       0.78186274510         -0.9436274510
## cr_socialmedia         -0.65196078431       0.71813725490         -0.8651960784
## cr_voice               -0.53921568627       0.67647058824         -0.4191176471
## cr_gendernormscale_f1 -0.75735294118       0.79411764706         -0.8039215686
## cr_gendernormscale_f2 -0.61029411765       0.58578431373         -0.7181372549
## cr_gendernormscale_f3 -0.80392156863       0.74264705882         -0.7720588235
## cr_gendernormscale_f4 -0.61764705882       0.67156862745         -0.7990196078
## cr_gendernormscale_f5  0.14215686275      -0.27450980392          0.1495098039
## cr_gendernormscale_f6 -0.02205882353      -0.01225490196         -0.1348039216
## cr_gendernormscale_f7 -0.74754901961       0.82598039216         -0.6985294118
## cr_cs_crage            -0.48284313725       0.65441176471         -0.4289215686
## af_asset_10            -0.69117647059       0.76225490196         -0.9191176471
## peervio_ordinal         0.08823529412       0.25245098039         -0.3995098039
##                             cr_talk      cr_media cr_socialmedia       cr_voice
## cr_ghq12               -0.7132352941 -0.6715686275  -0.6519607843 -0.53921568627
## cr_copingresilience     0.9411764706  0.7818627451   0.7181372549  0.67647058824
## cr_genderedactivities  -0.7794117647 -0.9436274510  -0.8651960784 -0.41911764706
## cr_talk                 1.0000000000  0.6862745098   0.6397058824  0.80392156863
## cr_media                0.6862745098  1.0000000000   0.9534313725  0.36029411765
## cr_socialmedia          0.6397058824  0.9534313725   1.0000000000  0.32598039216
## cr_voice                0.8039215686  0.3602941176   0.3259803922  1.00000000000
## cr_gendernormscale_f1   0.7083333333  0.7818627451   0.7843137255  0.42647058824
## cr_gendernormscale_f2   0.4632352941  0.7205882353   0.7500000000  0.05147058824
## cr_gendernormscale_f3   0.6372549020  0.7205882353   0.7279411765  0.37990196078
## cr_gendernormscale_f4   0.5318627451  0.8308823529   0.8480392157  0.10294117647
## cr_gendernormscale_f5  -0.3848039216 -0.1151960784   0.0000000000 -0.64460784314
## cr_gendernormscale_f6  -0.1862745098  0.1519607843   0.1838235294 -0.41911764706
## cr_gendernormscale_f7   0.8406862745  0.6936274510   0.6029411765  0.77205882353
## cr_cs_crage             0.6446078431  0.4558823529   0.3946078431  0.71078431373
## af_asset_10             0.6274509804  0.9828431373   0.9436274510  0.28431372549
## peervio_ordinal         0.3651960784  0.3529411765   0.3112745098  0.20833333333
##                       cr_gendernormscale_f1 cr_gendernormscale_f2
## cr_ghq12                     -0.75735294118        -0.61029411765
## cr_copingresilience           0.79411764706         0.58578431373
## cr_genderedactivities        -0.80392156863        -0.71813725490
## cr_talk                       0.70833333333         0.46323529412
## cr_media                      0.78186274510         0.72058823529
## cr_socialmedia                0.78431372549         0.75000000000
## cr_voice                      0.42647058824         0.05147058824
## cr_gendernormscale_f1         1.00000000000         0.85049019608
## cr_gendernormscale_f2         0.85049019608         1.00000000000
## cr_gendernormscale_f3         0.94607843137         0.86029411765
## cr_gendernormscale_f4         0.87745098039         0.96078431373
## cr_gendernormscale_f5         0.05882352941         0.41911764706
## cr_gendernormscale_f6         0.32107843137         0.63480392157
## cr_gendernormscale_f7         0.67401960784         0.36764705882
## cr_cs_crage                   0.47794117647         0.09313725490
## af_asset_10                   0.79656862745         0.74754901961
## peervio_ordinal               0.19852941176         0.12990196078
##                       cr_gendernormscale_f3 cr_gendernormscale_f4
```

```
## cr_ghq12                  -0.80392156863        -0.6176470588
## cr_copingresilience        0.74264705882         0.6715686275
## cr_genderedactivities     -0.77205882353        -0.7990196078
## cr_talk                    0.63725490196         0.5318627451
## cr_media                   0.72058823529         0.8308823529
## cr_socialmedia             0.72794117647         0.8480392157
## cr_voice                   0.37990196078         0.1029411765
## cr_gendernormscale_f1      0.94607843137         0.8774509804
## cr_gendernormscale_f2      0.86029411765         0.9607843137
## cr_gendernormscale_f3      1.00000000000         0.8406862745
## cr_gendernormscale_f4      0.84068627451         1.0000000000
## cr_gendernormscale_f5      0.16666666667         0.2990196078
## cr_gendernormscale_f6      0.40931372549         0.5465686275
## cr_gendernormscale_f7      0.57107843137         0.4607843137
## cr_cs_crage                0.32843137255         0.2401960784
## af_asset_10                0.74264705882         0.8651960784
## peervio_ordinal            0.07598039216         0.1617647059
##                         cr_gendernormscale_f5 cr_gendernormscale_f6
## cr_ghq12                   0.14215686275        -0.02205882353
## cr_copingresilience       -0.27450980392        -0.01225490196
## cr_genderedactivities      0.14950980392        -0.13480392157
## cr_talk                   -0.38480392157        -0.18627450980
## cr_media                  -0.11519607843         0.15196078431
## cr_socialmedia             0.00000000000         0.18382352941
## cr_voice                  -0.64460784314        -0.41911764706
## cr_gendernormscale_f1      0.05882352941         0.32107843137
## cr_gendernormscale_f2      0.41911764706         0.63480392157
## cr_gendernormscale_f3      0.16666666667         0.40931372549
## cr_gendernormscale_f4      0.29901960784         0.54656862745
## cr_gendernormscale_f5      1.00000000000         0.76225490196
## cr_gendernormscale_f6      0.76225490196         1.00000000000
## cr_gendernormscale_f7     -0.56372549020        -0.25245098039
## cr_cs_crage               -0.63480392157        -0.28186274510
## af_asset_10               -0.03676470588         0.24754901961
## peervio_ordinal           -0.26715686275        -0.15196078431
##                         cr_gendernormscale_f7   cr_cs_crage   af_asset_10
## cr_ghq12                     -0.7475490196 -0.4828431373 -0.69117647059
## cr_copingresilience           0.8259803922  0.6544117647  0.76225490196
## cr_genderedactivities        -0.6985294118 -0.4289215686 -0.91911764706
## cr_talk                       0.8406862745  0.6446078431  0.62745098039
## cr_media                      0.6936274510  0.4558823529  0.98284313725
## cr_socialmedia                0.6029411765  0.3946078431  0.94362745098
## cr_voice                      0.7720588235  0.7107843137  0.28431372549
## cr_gendernormscale_f1         0.6740196078  0.4779411765  0.79656862745
## cr_gendernormscale_f2         0.3676470588  0.0931372549  0.74754901961
## cr_gendernormscale_f3         0.5710784314  0.3284313725  0.74264705882
## cr_gendernormscale_f4         0.4607843137  0.2401960784  0.86519607843
## cr_gendernormscale_f5        -0.5637254902 -0.6348039216 -0.03676470588
## cr_gendernormscale_f6        -0.2524509804 -0.2818627451  0.24754901961
## cr_gendernormscale_f7         1.0000000000  0.8602941176  0.66421568627
## cr_cs_crage                   0.8602941176  1.0000000000  0.45833333333
## af_asset_10                   0.6642156863  0.4583333333  1.00000000000
## peervio_ordinal               0.2843137255  0.1495098039  0.24509803922
##                         peervio_ordinal
## cr_ghq12                  0.08823529412
## cr_copingresilience       0.25245098039
```

```
## cr_genderedactivities  -0.39950980392
## cr_talk                 0.36519607843
## cr_media                0.35294117647
## cr_socialmedia          0.31127450980
## cr_voice                0.20833333333
## cr_gendernormscale_f1   0.19852941176
## cr_gendernormscale_f2   0.12990196078
## cr_gendernormscale_f3   0.07598039216
## cr_gendernormscale_f4   0.16176470588
## cr_gendernormscale_f5  -0.26715686275
## cr_gendernormscale_f6  -0.15196078431
## cr_gendernormscale_f7   0.28431372549
## cr_cs_crage             0.14950980392
## af_asset_10             0.24509803922
## peervio_ordinal         1.00000000000
```

```
spearman_corr
```

```
##                          cr_ghq12 cr_copingresilience cr_genderedactivities
## cr_ghq12               1.00000000000      -0.80637254902          0.7352941176
## cr_copingresilience   -0.80637254902       1.00000000000         -0.8774509804
## cr_genderedactivities  0.73529411765      -0.87745098039          1.0000000000
## cr_talk               -0.71323529412       0.94117647059         -0.7794117647
## cr_media              -0.67156862745       0.78186274510         -0.9436274510
## cr_socialmedia        -0.65196078431       0.71813725490         -0.8651960784
## cr_voice              -0.53921568627       0.67647058824         -0.4191176471
## cr_gendernormscale_f1 -0.75735294118       0.79411764706         -0.8039215686
## cr_gendernormscale_f2 -0.61029411765       0.58578431373         -0.7181372549
## cr_gendernormscale_f3 -0.80392156863       0.74264705882         -0.7720588235
## cr_gendernormscale_f4 -0.61764705882       0.67156862745         -0.7990196078
## cr_gendernormscale_f5  0.14215686275      -0.27450980392          0.1495098039
## cr_gendernormscale_f6 -0.02205882353      -0.01225490196         -0.1348039216
## cr_gendernormscale_f7 -0.74754901961       0.82598039216         -0.6985294118
## cr_cs_crage           -0.48284313725       0.65441176471         -0.4289215686
## af_asset_10           -0.69117647059       0.76225490196         -0.9191176471
## peervio_ordinal        0.08823529412       0.25245098039         -0.3995098039
##                          cr_talk      cr_media cr_socialmedia       cr_voice
## cr_ghq12              -0.7132352941 -0.6715686275  -0.6519607843 -0.53921568627
## cr_copingresilience    0.9411764706  0.7818627451   0.7181372549  0.67647058824
## cr_genderedactivities -0.7794117647 -0.9436274510  -0.8651960784 -0.41911764706
## cr_talk                1.0000000000  0.6862745098   0.6397058824  0.80392156863
## cr_media               0.6862745098  1.0000000000   0.9534313725  0.36029411765
## cr_socialmedia         0.6397058824  0.9534313725   1.0000000000  0.32598039216
## cr_voice               0.8039215686  0.3602941176   0.3259803922  1.00000000000
## cr_gendernormscale_f1  0.7083333333  0.7818627451   0.7843137255  0.42647058824
## cr_gendernormscale_f2  0.4632352941  0.7205882353   0.7500000000  0.05147058824
## cr_gendernormscale_f3  0.6372549020  0.7205882353   0.7279411765  0.37990196078
## cr_gendernormscale_f4  0.5318627451  0.8308823529   0.8480392157  0.10294117647
## cr_gendernormscale_f5 -0.3848039216 -0.1151960784   0.0000000000 -0.64460784314
## cr_gendernormscale_f6 -0.1862745098  0.1519607843   0.1838235294 -0.41911764706
## cr_gendernormscale_f7  0.8406862745  0.6936274510   0.6029411765  0.77205882353
## cr_cs_crage            0.6446078431  0.4558823529   0.3946078431  0.71078431373
## af_asset_10            0.6274509804  0.9828431373   0.9436274510  0.28431372549
## peervio_ordinal        0.3651960784  0.3529411765   0.3112745098  0.20833333333
##                       cr_gendernormscale_f1 cr_gendernormscale_f2
## cr_ghq12                     -0.75735294118        -0.61029411765
## cr_copingresilience           0.79411764706         0.58578431373
## cr_genderedactivities        -0.80392156863        -0.71813725490
## cr_talk                       0.70833333333         0.46323529412
## cr_media                      0.78186274510         0.72058823529
## cr_socialmedia                0.78431372549         0.75000000000
## cr_voice                      0.42647058824         0.05147058824
## cr_gendernormscale_f1         1.00000000000         0.85049019608
## cr_gendernormscale_f2         0.85049019608         1.00000000000
## cr_gendernormscale_f3         0.94607843137         0.86029411765
## cr_gendernormscale_f4         0.87745098039         0.96078431373
## cr_gendernormscale_f5         0.05882352941         0.41911764706
## cr_gendernormscale_f6         0.32107843137         0.63480392157
## cr_gendernormscale_f7         0.67401960784         0.36764705882
## cr_cs_crage                   0.47794117647         0.09313725490
## af_asset_10                   0.79656862745         0.74754901961
## peervio_ordinal               0.19852941176         0.12990196078
##                       cr_gendernormscale_f3 cr_gendernormscale_f4
```

```
## cr_ghq12                    -0.80392156863        -0.6176470588
## cr_copingresilience          0.74264705882         0.6715686275
## cr_genderedactivities       -0.77205882353        -0.7990196078
## cr_talk                      0.63725490196         0.5318627451
## cr_media                     0.72058823529         0.8308823529
## cr_socialmedia               0.72794117647         0.8480392157
## cr_voice                     0.37990196078         0.1029411765
## cr_gendernormscale_f1        0.94607843137         0.8774509804
## cr_gendernormscale_f2        0.86029411765         0.9607843137
## cr_gendernormscale_f3        1.00000000000         0.8406862745
## cr_gendernormscale_f4        0.84068627451         1.0000000000
## cr_gendernormscale_f5        0.16666666667         0.2990196078
## cr_gendernormscale_f6        0.40931372549         0.5465686275
## cr_gendernormscale_f7        0.57107843137         0.4607843137
## cr_cs_crage                  0.32843137255         0.2401960784
## af_asset_10                  0.74264705882         0.8651960784
## peervio_ordinal              0.07598039216         0.1617647059
##                       cr_gendernormscale_f5 cr_gendernormscale_f6
## cr_ghq12                     0.14215686275        -0.02205882353
## cr_copingresilience         -0.27450980392        -0.01225490196
## cr_genderedactivities        0.14950980392        -0.13480392157
## cr_talk                     -0.38480392157        -0.18627450980
## cr_media                    -0.11519607843         0.15196078431
## cr_socialmedia               0.00000000000         0.18382352941
## cr_voice                    -0.64460784314        -0.41911764706
## cr_gendernormscale_f1        0.05882352941         0.32107843137
## cr_gendernormscale_f2        0.41911764706         0.63480392157
## cr_gendernormscale_f3        0.16666666667         0.40931372549
## cr_gendernormscale_f4        0.29901960784         0.54656862745
## cr_gendernormscale_f5        1.00000000000         0.76225490196
## cr_gendernormscale_f6        0.76225490196         1.00000000000
## cr_gendernormscale_f7       -0.56372549020        -0.25245098039
## cr_cs_crage                 -0.63480392157        -0.28186274510
## af_asset_10                 -0.03676470588         0.24754901961
## peervio_ordinal             -0.26715686275        -0.15196078431
##                       cr_gendernormscale_f7   cr_cs_crage    af_asset_10
## cr_ghq12                      -0.7475490196 -0.4828431373 -0.69117647059
## cr_copingresilience            0.8259803922  0.6544117647  0.76225490196
## cr_genderedactivities         -0.6985294118 -0.4289215686 -0.91911764706
## cr_talk                        0.8406862745  0.6446078431  0.62745098039
## cr_media                       0.6936274510  0.4558823529  0.98284313725
## cr_socialmedia                 0.6029411765  0.3946078431  0.94362745098
## cr_voice                       0.7720588235  0.7107843137  0.28431372549
## cr_gendernormscale_f1          0.6740196078  0.4779411765  0.79656862745
## cr_gendernormscale_f2          0.3676470588  0.0931372549  0.74754901961
## cr_gendernormscale_f3          0.5710784314  0.3284313725  0.74264705882
## cr_gendernormscale_f4          0.4607843137  0.2401960784  0.86519607843
## cr_gendernormscale_f5         -0.5637254902 -0.6348039216 -0.03676470588
## cr_gendernormscale_f6         -0.2524509804 -0.2818627451  0.24754901961
## cr_gendernormscale_f7          1.0000000000  0.8602941176  0.66421568627
## cr_cs_crage                    0.8602941176  1.0000000000  0.45833333333
## af_asset_10                    0.6642156863  0.4583333333  1.00000000000
## peervio_ordinal                0.2843137255  0.1495098039  0.24509803922
##                       peervio_ordinal
## cr_ghq12                0.08823529412
## cr_copingresilience     0.25245098039
```

```
## cr_genderedactivities  -0.39950980392
## cr_talk                 0.36519607843
## cr_media                0.35294117647
## cr_socialmedia          0.31127450980
## cr_voice                0.20833333333
## cr_gendernormscale_f1   0.19852941176
## cr_gendernormscale_f2   0.12990196078
## cr_gendernormscale_f3   0.07598039216
## cr_gendernormscale_f4   0.16176470588
## cr_gendernormscale_f5  -0.26715686275
## cr_gendernormscale_f6  -0.15196078431
## cr_gendernormscale_f7   0.28431372549
## cr_cs_crage             0.14950980392
## af_asset_10             0.24509803922
## peervio_ordinal         1.00000000000
```

```
#test normality for bivarite correlations
shapiro.test(data_ET20_final$cr_female) #significant ->not normally distributed
```

```
##
##  Shapiro-Wilk normality test
##
## data:  data_ET20_final$cr_female
## W = 0.62677704, p-value < 2.2204e-16
```

```
shapiro.test(data_ET20_final$cr_rolemodel)
```

```
##
##  Shapiro-Wilk normality test
##
## data:  data_ET20_final$cr_rolemodel
## W = 0.63636287, p-value < 2.2204e-16
```

```
shapiro.test(data_ET20_final$cr_married)
```

```
##
##  Shapiro-Wilk normality test
##
## data:  data_ET20_final$cr_married
## W = 0.50344964, p-value < 2.2204e-16
```

```
shapiro.test(data_ET20_final$cr_opfriend)
```

```
##
##  Shapiro-Wilk normality test
##
## data:  data_ET20_final$cr_opfriend
## W = 0.52655629, p-value < 2.2204e-16
```

```
shapiro.test(data_ET20_final$cr_opelder)
```

```
##
##  Shapiro-Wilk normality test
##
## data:  data_ET20_final$cr_opelder
## W = 0.63431686, p-value < 2.2204e-16
```

```
shapiro.test(data_ET20_final$cr_workfambus)
```

```
##
##  Shapiro-Wilk normality test
##
## data:  data_ET20_final$cr_workfambus
## W = 0.19482941, p-value < 2.2204e-16
```

```
shapiro.test(data_ET20_final$cr_clubmember)
```

```
##
##  Shapiro-Wilk normality test
##
## data:  data_ET20_final$cr_clubmember
## W = 0.60711272, p-value < 2.2204e-16
```

```
shapiro.test(data_ET20_final$purpsamp)
```

```
##
##  Shapiro-Wilk normality test
##
## data:  data_ET20_final$purpsamp
## W = 0.607685, p-value < 2.2204e-16
```

```
shapiro.test(data_ET20_final$sample_r2_disability2)
```

```
##
##  Shapiro-Wilk normality test
##
## data:  data_ET20_final$sample_r2_disability2
## W = 0.31488492, p-value < 2.2204e-16
```

```r
#Phi coefficient for dichotomous variables
bin_vars <- data_ET20_final[, c("cr_female", "cr_rolemodel", "cr_married", "sample_r2_d
isability2", "cr_opfriend", "cr_opelder", "cr_clubmember", "cr_urban", "purpsamp", "cr_
workfambus")]

phi_matrix <- matrix(NA, nrow = ncol(bin_vars), ncol = ncol(bin_vars))
colnames(phi_matrix) <- colnames(bin_vars)
rownames(phi_matrix) <- colnames(bin_vars)

for (i in 1:(ncol(bin_vars) - 1)) {
  for (j in (i + 1):ncol(bin_vars)) {

    contingency_table <- table(bin_vars[[i]], bin_vars[[j]])

    phi_matrix[i, j] <- phi(contingency_table)
    phi_matrix[j, i] <- phi(contingency_table)
  }
}
print(phi_matrix)
```

```
##                       cr_female cr_rolemodel cr_married sample_r2_disability2
## cr_female                    NA        -0.14       0.34                 -0.01
## cr_rolemodel              -0.14           NA      -0.09                 -0.05
## cr_married                 0.34        -0.09         NA                 -0.08
## sample_r2_disability2     -0.01        -0.05      -0.08                    NA
## cr_opfriend               -0.03         0.12      -0.03                 -0.04
## cr_opelder                -0.06         0.14       0.01                 -0.03
## cr_clubmember             -0.18         0.25      -0.24                 -0.03
## cr_urban                   0.07        -0.15       0.12                 -0.03
## purpsamp                   0.16        -0.11       0.63                  0.11
## cr_workfambus             -0.01        -0.03       0.03                  0.03
##                       cr_opfriend cr_opelder cr_clubmember cr_urban purpsamp
## cr_female                   -0.03      -0.06         -0.18     0.07     0.16
## cr_rolemodel                 0.12       0.14          0.25    -0.15    -0.11
## cr_married                  -0.03       0.01         -0.24     0.12     0.63
## sample_r2_disability2       -0.04      -0.03         -0.03    -0.03     0.11
## cr_opfriend                    NA       0.29          0.14     0.01    -0.12
## cr_opelder                   0.29         NA          0.08    -0.03    -0.02
## cr_clubmember                0.14       0.08            NA    -0.11    -0.26
## cr_urban                     0.01      -0.03         -0.11       NA     0.09
## purpsamp                    -0.12      -0.02         -0.26     0.09       NA
## cr_workfambus               -0.08      -0.04         -0.08     0.02     0.03
##                       cr_workfambus
## cr_female                     -0.01
## cr_rolemodel                  -0.03
## cr_married                     0.03
## sample_r2_disability2          0.03
## cr_opfriend                   -0.08
## cr_opelder                    -0.04
## cr_clubmember                 -0.08
## cr_urban                       0.02
## purpsamp                       0.03
## cr_workfambus                    NA
```

6.1.3 Multicollinearity test

```
#multicollinearity by VIF
library(car)  # For VIF calculation
```

```
## Warning: Paket 'car' wurde unter R Version 4.5.3 erstellt
```

```
## Lade nötiges Paket: carData
```

```
##
## Attache Paket: 'car'
```

```
## Das folgende Objekt ist maskiert 'package:psychTools':
##
##     recode
```

```
## Das folgende Objekt ist maskiert 'package:expss':
##
##     recode
```

```
## Das folgende Objekt ist maskiert 'package:psych':
##
##     logit
```

```
## Das folgende Objekt ist maskiert 'package:dplyr':
##
##     recode
```

```
## Das folgende Objekt ist maskiert 'package:purrr':
##
##     some
```

```
subsetCor1 <- as.data.frame(subsetCor)

modelVIF <- lm(cr_ghq12~ cr_copingresilience+ cr_genderedactivities+ cr_talk + cr_media
+ cr_socialmedia +  cr_voice + cr_gendernormscale_f1+ cr_gendernormscale_f2+ cr_gendern
ormscale_f3+ cr_gendernormscale_f4 + cr_cs_crage + af_asset_10, data=subsetCor1)

vifvalues <- vif(modelVIF)
vifvalues
```

```
##    cr_copingresilience cr_genderedactivities                 cr_talk
##            2.348962715           5.737402392             2.943263324
##               cr_media        cr_socialmedia                cr_voice
##            5.036755896           1.598556094             1.726167376
## cr_gendernormscale_f1 cr_gendernormscale_f2 cr_gendernormscale_f3
##            2.894101535           2.466517342             1.686682081
## cr_gendernormscale_f4           cr_cs_crage             af_asset_10
##            2.815527310           1.071105995             4.341195276
```

```
#all values below 5 ->no multicollinearity

# Visualizing the model
plot(modelVIF, which = 1, main = "Model Fit")
```

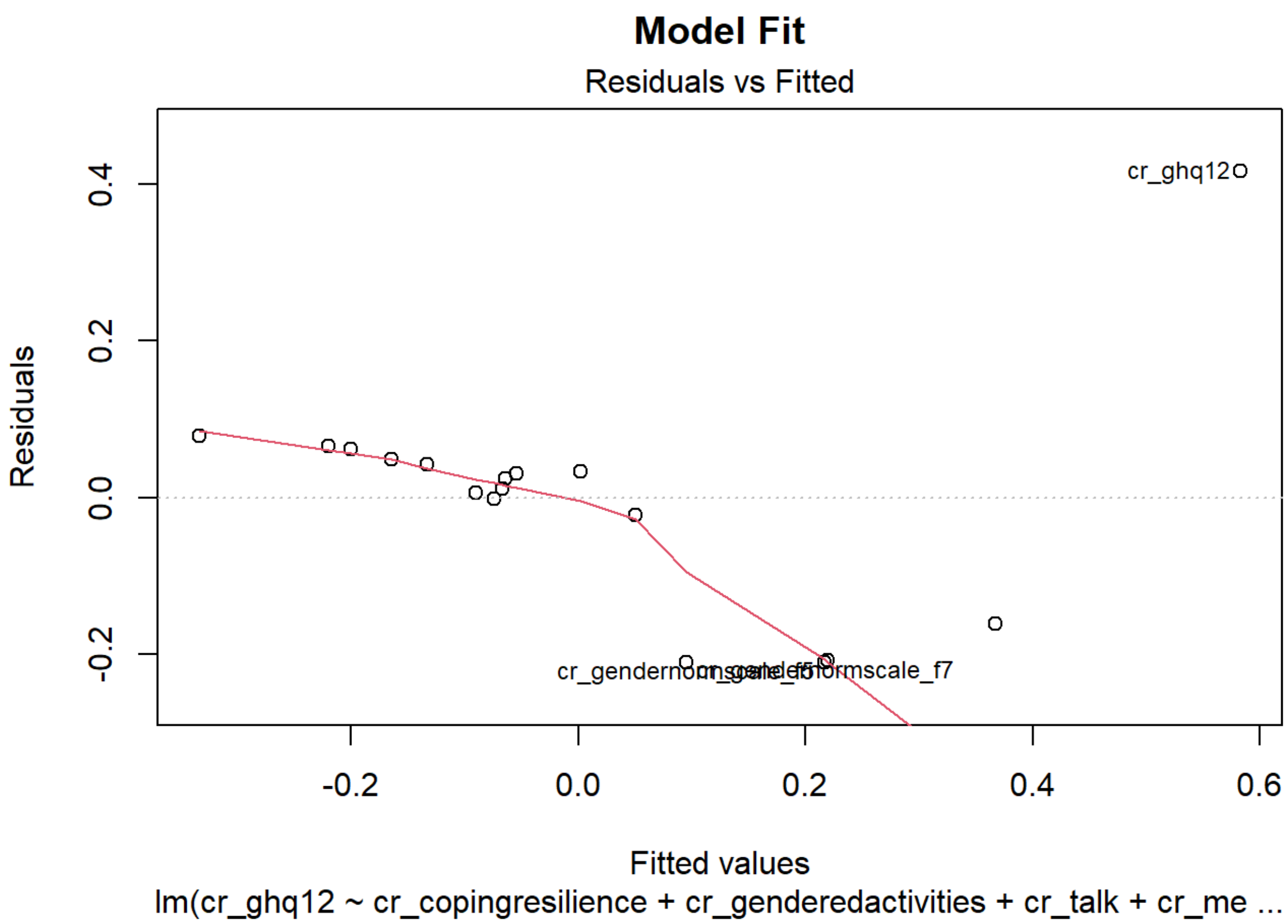


```
#3 outliers?

vifvalues <- vif(modelVIF)
vifvalues
```


```
##    cr_copingresilience cr_genderedactivities                 cr_talk
##            2.348962715           5.737402392             2.943263324
##               cr_media        cr_socialmedia                cr_voice
##            5.036755896           1.598556094             1.726167376
## cr_gendernormscale_f1 cr_gendernormscale_f2 cr_gendernormscale_f3
##            2.894101535           2.466517342             1.686682081
## cr_gendernormscale_f4           cr_cs_crage             af_asset_10
##            2.815527310           1.071105995             4.341195276
```

```
#all values below 5 = no multicollinearity

# Visualizing the model
plot(modelVIF, which = 1, main = "Model Fit")
```

6.1.4 Common method bias

```
#Hermans single factor: EFA with all measurement items without rotation using ML
#average variance explained by the factor must be less than 0.5

#creating one factor EFA -> only including indicator variables for the latent variables
(Gender Norms and Competencies) in this subset

subsetcommon = data_ET20_final %>% dplyr::select(cr_gendernormscale_f1, cr_gendernormsc
ale_f2, cr_gendernormscale_f3, cr_gendernormscale_f4, cr_socialmedia, cr_media, dCRET20
_rolem_y, dCRET20_clubm_y, cr_talk, cr_copingresilience, cr_voice ,dCRET20_opfriend_y,d
CRET20_opelder_y)


#single factor
singlefactor=fa(subsetcommon, nfactors=1, rotate="none", fm="ml")
singlefactor
```

```
## Factor Analysis using method =  ml
## Call: fa(r = subsetcommon, nfactors = 1, rotate = "none", fm = "ml")
## Standardized loadings (pattern matrix) based upon correlation matrix
##                        ML1    h2   u2 com
## cr_gendernormscale_f1 0.61 0.374 0.63   1
## cr_gendernormscale_f2 0.50 0.247 0.75   1
## cr_gendernormscale_f3 0.34 0.119 0.88   1
## cr_gendernormscale_f4 0.60 0.357 0.64   1
## cr_socialmedia        0.44 0.193 0.81   1
## cr_media              0.57 0.328 0.67   1
## dCRET20_rolem_y       0.46 0.208 0.79   1
## dCRET20_clubm_y       0.41 0.170 0.83   1
## cr_talk               0.57 0.322 0.68   1
## cr_copingresilience   0.53 0.285 0.72   1
## cr_voice              0.32 0.100 0.90   1
## dCRET20_opfriend_y    0.32 0.104 0.90   1
## dCRET20_opelder_y     0.26 0.065 0.93   1
##
##                 ML1
## SS loadings    2.87
## Proportion Var 0.22
##
## Mean item complexity =  1
## Test of the hypothesis that 1 factor is sufficient.
##
## df null model =  78  with the objective function =  2.4 with Chi Square =  11445.17
## df of  the model are 65  and the objective function was  0.65
##
## The root mean square of the residuals (RMSR) is  0.08
## The df corrected root mean square of the residuals is  0.09
##
## The harmonic n.obs is  2879 with the empirical chi square  3069.35  with prob <  0
## The total n.obs was  4777  with Likelihood Chi Square =  3088.7  with prob <  0
##
## Tucker Lewis Index of factoring reliability =  0.681
## RMSEA index =  0.099  and the 90 % confidence intervals are  0.096 0.102
## BIC =  2538.04
## Fit based upon off diagonal values = 0.89
## Measures of factor score adequacy
##                                                    ML1
## Correlation of (regression) scores with factors   0.89
## Multiple R square of scores with factors          0.80
## Minimum correlation of possible factor scores     0.60
```

```
#result: proportion var = 0.19 < 0.5 ->acceptable
```

6.2 Subsetting data

```
#cr_media is still heaven-labelled ->into numeric
class(data_ET20_final$cr_media)
```

```
## [1] "numeric"
```

```
data_ET20_final$cr_media <- as.numeric(data_ET20_final$cr_media)
class(data_ET20_final$cr_socialmedia)
```

```
## [1] "numeric"
```

```
data_ET20_final$cr_socialmedia <- as.numeric(data_ET20_final$cr_socialmedia)

#subset
subsetSEMNeu = data_ET20_final %>%
dplyr::select(c(cr_ghq12, cr_copingresilience, cr_genderedactivities, cr_media,  cr_soc
ialmedia, cr_gendernormscale12rec, cr_voice,dCRET20_rolem_y, dCRET20_trustfr_y, dCRET20
_speakup_y, dCRET20_opfriend_y, dCRET20_opelder_y,dCRET20_clubm_y, peervio_ordinal, cr_
talk, cr_gendernormscale_f1, cr_gendernormscale_f2, cr_gendernormscale_f3, cr_gendernor
mscale_f4,cr_gendernormscale_f5, cr_gendernormscale_f6, cr_gendernormscale_f7, dCRET20_
female, cr_cs_crage, dCRET20_urban, dCRET20_married_y, dCRET20_purpsamp_y, dCRET20_disa
bility_y, af_asset_10, dCRET20_fatherinhh,  dCRET20_aspiratt_sec, dCRET20_aspiratt_teac
h, dCRET20_aspiratt_uni, dCRET20_languagehh_201, dCRET20_languagehh_202, dCRET20_langua
gehh_211, dCRET20_workfambus_y,

cr_edu_culture, cr_edu_boysch, cr_edu_science, cr_edu_girlnohelp, cr_edu_raisingvoice,
cr_edu_boysfeelings, cr_edu_boystough, cr_edu_boysdefend, cr_edu_girlsprotect, cr_edu_g
irlshumble, cr_edu_boysweak, cr_edu_equaltreat, cr_edu_teachpunish, cr_edu_teachnotpuni
sh, cr_edu_boysout, cr_ta_eesharetasks, cr_ta_womanrole, cr_ta_eemandecid, cr_fi_girlch
ance, cr_fi_goodwife, cr_fi_girlsavings, cr_srh_waitedu, cr_srh_controldaugt, cr_srh_bo
yfinal, cr_srh_teasegirl, cr_srh_teaseboy, cr_srh_genderroles, cr_srh_aware, cr_srh_cha
ngereaction, cr_vi_hit, cr_vi_privematt, cr_vi_tolerate,
cr_ta_eemandecid, rand_weight))
```

7 Structural Equation Models

7.1 SEM in submission

```
testmPUBSub <- '
Competencies =~ cr_talk + cr_copingresilience +  cr_voice + dCRET20_opfriend_y + dCRET2
0_opelder_y
GenderNorms =~ cr_media  + cr_socialmedia + dCRET20_rolem_y + dCRET20_clubm_y
GenderAttitudes =~ cr_gendernormscale_f1 + cr_gendernormscale_f2 + cr_gendernormscale_f
3 + cr_gendernormscale_f4

#mediations/regressions
GenderAttitudes ~ aGN_GA * GenderNorms + aSex_GA * dCRET20_female
cr_genderedactivities ~ aGN_GR * GenderNorms
cr_ghq12~ bGA_MH * GenderAttitudes + bGR_MH * cr_genderedactivities + cSexMH * dCRET20_
female + cGNMH * GenderNorms + Competencies
Competencies ~ bGR_COM * cr_genderedactivities + cGNCOM * GenderNorms#new

#covariances to improve model fit based on modindices
#cr_copingresilience~~ cr_genderedactivities
GenderNorms ~~  GenderAttitudes
dCRET20_opfriend_y ~~ dCRET20_opelder_y
cr_genderedactivities ~~ dCRET20_female
GenderAttitudes ~~ cr_ghq12
dCRET20_rolem_y ~~ cr_genderedactivities

#partial indirect effects
partGNGAMH := aGN_GA * bGA_MH
partGNGRMH := aGN_GR * bGR_MH
partGNGRCOM  := aGN_GR * bGR_COM
partSexGAMH := aSex_GA * bGA_MH

#total indirect effects
tieGNMH := partGNGAMH + partGNGRMH
tieGNCOM := partGNGRCOM
tieSexMH:= partSexGAMH

#total effect
teSexMH := tieSexMH + cSexMH
teGNMH := tieGNMH + cGNMH
teGNCOM := tieGNCOM + cGNCOM#new

med_propSexMH := partSexGAMH / teSexMH
med_propGNGAMH := partGNGAMH / teGNMH
med_propGNGRMH := partGNGRMH / teGNMH
med_propGNGRCOM := partGNGRCOM / teGNCOM

#covariates to the two exogeneous variables (or to the ones the covariates apply)
dCRET20_female~cr_cs_crage + dCRET20_urban + dCRET20_married_y + dCRET20_purpsamp_y + d
CRET20_disability_y + af_asset_10 + dCRET20_fatherinhh +  dCRET20_aspiratt_sec +
dCRET20_aspiratt_teach + dCRET20_aspiratt_uni +  dCRET20_languagehh_201 + dCRET20_langu
agehh_202 + dCRET20_languagehh_211  + dCRET20_workfambus_y

GenderNorms~cr_cs_crage + dCRET20_urban + dCRET20_married_y +dCRET20_purpsamp_y + dCRET
20_disability_y + af_asset_10 + dCRET20_fatherinhh +  dCRET20_aspiratt_sec +
dCRET20_aspiratt_teach + dCRET20_aspiratt_uni +  dCRET20_languagehh_201 + dCRET20_langu
agehh_202 + dCRET20_languagehh_211  + dCRET20_workfambus_y
```

```
'
fit.testmPUBSub <- sem(testmPUBSub, data=subsetSEMNeu, estimator="MLR", sampling.weight
s = "rand_weight")
summary(fit.testmPUBSub, fit.measures=TRUE, standardized=TRUE, rsq=TRUE, ci=TRUE)
```

```
## lavaan 0.6-21 ended normally after 123 iterations
##
##   Estimator                                         ML
##   Optimization method                           NLMINB
##   Number of model parameters                        72
##
##                                                   Used       Total
##   Number of observations                          1584        4777
##   Sampling weights variable                 rand_weight
##
## Model Test User Model:
##                                               Standard      Scaled
##   Test Statistic                              1648.365    1426.456
##   Degrees of freedom                               288         288
##   P-value (Chi-square)                           0.000       0.000
##   Scaling correction factor                                  1.156
##     Yuan-Bentler correction (Mplus variant)
##
## Model Test Baseline Model:
##
##   Test statistic                              6091.309    5245.946
##   Degrees of freedom                               344         344
##   P-value                                        0.000       0.000
##   Scaling correction factor                                  1.161
##
## User Model versus Baseline Model:
##
##   Comparative Fit Index (CFI)                    0.763       0.768
##   Tucker-Lewis Index (TLI)                       0.717       0.723
##
##   Robust Comparative Fit Index (CFI)                         0.769
##   Robust Tucker-Lewis Index (TLI)                            0.724
##
## Loglikelihood and Information Criteria:
##
##   Loglikelihood user model (H0)             -40188.774  -40188.774
##   Scaling correction factor                                  1.245
##       for the MLR correction
##   Loglikelihood unrestricted model (H1)     -39364.592  -39364.592
##   Scaling correction factor                                  1.173
##       for the MLR correction
##
##   Akaike (AIC)                               80521.548   80521.548
##   Bayesian (BIC)                             80908.023   80908.023
##   Sample-size adjusted Bayesian (SABIC)      80679.294   80679.294
##
## Root Mean Square Error of Approximation:
##
##   RMSEA                                          0.055       0.050
##   90 Percent confidence interval - lower         0.052       0.048
##   90 Percent confidence interval - upper         0.057       0.052
##   P-value H_0: RMSEA <= 0.050                    0.002       0.507
##   P-value H_0: RMSEA >= 0.080                    0.000       0.000
##
##   Robust RMSEA                                               0.054
```

```
##   90 Percent confidence interval - lower                      0.051
##   90 Percent confidence interval - upper                      0.056
##   P-value H_0: Robust RMSEA <= 0.050                          0.014
##   P-value H_0: Robust RMSEA >= 0.080                          0.000
##
## Standardized Root Mean Square Residual:
##
##   SRMR                                           0.042       0.042
##
## Parameter Estimates:
##
##   Standard errors                             Sandwich
##   Information bread                           Observed
##   Observed information based on                Hessian
##
## Latent Variables:
##                      Estimate  Std.Err  z-value  P(>|z|) ci.lower ci.upper
##   Competencies =~
##     cr_talk             1.000                               1.000    1.000
##     cr_copingrslnc      3.210    0.274   11.722    0.000    2.673    3.747
##     cr_voice            2.549    0.178   14.340    0.000    2.201    2.898
##     dCRET20_pfrnd_      0.170    0.020    8.477    0.000    0.131    0.209
##     dCRET20_pldr_y      0.188    0.020    9.365    0.000    0.149    0.228
##   GenderNorms =~
##     cr_media            1.000                               1.000    1.000
##     cr_socialmedia      0.251    0.022   11.496    0.000    0.209    0.294
##     dCRET20_rolm_y      0.232    0.017   13.395    0.000    0.198    0.266
##     dCRET20_clbm_y      0.223    0.017   13.509    0.000    0.191    0.256
##   GenderAttitudes =~
##     cr_gndrnrmsc_1      1.000                               1.000    1.000
##     cr_gndrnrmsc_2      0.794    0.055   14.527    0.000    0.687    0.901
##     cr_gndrnrmsc_3      0.362    0.032   11.307    0.000    0.299    0.424
##     cr_gndrnrmsc_4      0.769    0.059   12.973    0.000    0.652    0.885
##    Std.lv  Std.all
##
##     0.771    0.663
##     2.476    0.636
##     1.966    0.395
##     0.131    0.320
##     0.145    0.293
##
##     0.958    0.611
##     0.241    0.367
##     0.222    0.446
##     0.214    0.430
##
##     1.446    0.651
##     1.149    0.548
##     0.523    0.360
##     1.112    0.580
##
## Regressions:
##                           Estimate  Std.Err  z-value  P(>|z|) ci.lower ci.upper
##   GenderAttitudes ~
##     GndrN (aGN_GA)           1.274    0.103   12.377    0.000    1.072    1.476
##     dCRET   (aS_G)           0.501    0.087    5.779    0.000    0.331    0.671
```

```
##   cr_genderedactivities ~
##     GndrN (aGN_GR)          -1.319    0.081  -16.287    0.000   -1.478   -1.160
##   cr_ghq12 ~
##     GndrA   (bGA_)          -0.499    0.570   -0.876    0.381   -1.615    0.617
##     cr_gn  (bGR_M)          -0.037    0.077   -0.478    0.633   -0.187    0.114
##     dCRET   (cSMH)          -0.317    0.368   -0.861    0.389   -1.037    0.404
##     GndrN   (cGNM)           0.501    0.830    0.603    0.546   -1.126    2.127
##     Cmptn                   -1.082    0.398   -2.722    0.006   -1.862   -0.303
##   Competencies ~
##     cr_gn  (bGR_C)           0.044    0.022    1.953    0.051   -0.000    0.088
##     GndrN   (cGNC)           0.629    0.069    9.121    0.000    0.494    0.764
##   dCRET20_female ~
##     cr_c_                   -0.029    0.014   -2.089    0.037   -0.056   -0.002
##     dCRET                   -0.033    0.027   -1.214    0.225   -0.087    0.020
##     dCRET                   -0.280    0.058   -4.811    0.000   -0.394   -0.166
##     dCRET                    0.077    0.068    1.123    0.261   -0.057    0.211
##     dCRET                    0.077    0.056    1.371    0.170   -0.033    0.186
##     a__10                   -0.003    0.005   -0.659    0.510   -0.013    0.007
##     dCRET                   -0.038    0.030   -1.281    0.200   -0.096    0.020
##     dCRET                   -0.022    0.059   -0.368    0.713   -0.138    0.094
##     dCRET                   -0.201    0.080   -2.496    0.013   -0.358   -0.043
##     dCRET                   -0.262    0.057   -4.600    0.000   -0.374   -0.150
##     dCRET                   -0.111    0.029   -3.762    0.000   -0.168   -0.053
##     dCRET                   -0.089    0.042   -2.131    0.033   -0.170   -0.007
##     dCRET                   -0.006    0.100   -0.060    0.953   -0.202    0.190
##     dCRET                   -0.099    0.057   -1.727    0.084   -0.211    0.013
##   GenderNorms ~
##     cr_c_                    0.072    0.024    3.047    0.002    0.026    0.119
##     dCRET                   -0.268    0.054   -4.994    0.000   -0.373   -0.163
##     dCRET                    0.716    0.138    5.201    0.000    0.446    0.986
##     dCRET                    0.036    0.107    0.337    0.736   -0.174    0.247
##     dCRET                    0.224    0.087    2.570    0.010    0.053    0.395
##     a__10                    0.125    0.013    9.902    0.000    0.100    0.150
##     dCRET                   -0.073    0.048   -1.508    0.132   -0.168    0.022
##     dCRET                    0.210    0.110    1.913    0.056   -0.005    0.425
##     dCRET                    0.431    0.128    3.370    0.001    0.180    0.682
##     dCRET                    0.914    0.106    8.602    0.000    0.706    1.122
##     dCRET                   -0.571    0.050  -11.335    0.000   -0.669   -0.472
##     dCRET                   -0.488    0.065   -7.471    0.000   -0.616   -0.360
##     dCRET                   -0.157    0.170   -0.924    0.356   -0.490    0.176
##     dCRET                   -0.201    0.083   -2.420    0.016   -0.363   -0.038
##    Std.lv  Std.all
##
##     0.844    0.844
##     0.346    0.173
##
##    -1.264   -0.610
##
##    -0.722   -0.193
##    -0.037   -0.020
##    -0.317   -0.042
##     0.480    0.128
##    -0.835   -0.223
##
##     0.057    0.118
##     0.782    0.782
```

```
##
##    -0.029   -0.054
##    -0.033   -0.033
##    -0.280   -0.096
##     0.077    0.030
##     0.077    0.037
##    -0.003   -0.018
##    -0.038   -0.032
##    -0.022   -0.018
##    -0.201   -0.088
##    -0.262   -0.250
##    -0.111   -0.103
##    -0.089   -0.064
##    -0.006   -0.001
##    -0.099   -0.044
##
##     0.075    0.070
##    -0.280   -0.139
##     0.747    0.127
##     0.038    0.007
##     0.234    0.056
##     0.131    0.358
##    -0.076   -0.032
##     0.219    0.092
##     0.450    0.099
##     0.954    0.453
##    -0.595   -0.276
##    -0.509   -0.183
##    -0.164   -0.019
##    -0.210   -0.046
##
## Covariances:
##                            Estimate  Std.Err  z-value  P(>|z|) ci.lower
##   .GenderNorms ~~
##     .GenderAttituds           -0.206    0.056   -3.705    0.000   -0.316
##   .dCRET20_opfriend_y ~~
##     .dCRET20_pldr_y            0.041    0.005    8.475    0.000    0.032
##   .cr_genderedactivities ~~
##     .dCRET20_female            0.290    0.024   12.000    0.000    0.243
##   .GenderAttitudes ~~
##     .cr_ghq12                  0.491    0.544    0.902    0.367   -0.576
##   .dCRET20_rolem_y ~~
##     .cr_gendrdctvts            0.099    0.023    4.294    0.000    0.054
##  ci.upper   Std.lv  Std.all
##
##    -0.097   -0.337   -0.337
##
##     0.051    0.041    0.224
##
##     0.338    0.290    0.370
##
##     1.557    0.464    0.127
##
##     0.144    0.099    0.135
##
## Variances:
```

```
##                     Estimate  Std.Err  z-value  P(>|z|) ci.lower ci.upper
##    .cr_talk            0.757    0.055   13.680    0.000    0.649    0.866
##    .cr_copingrslnc     9.018    0.624   14.451    0.000    7.795   10.241
##    .cr_voice          20.892    0.850   24.587    0.000   19.227   22.557
##    .dCRET20_pfrnd_     0.151    0.006   24.609    0.000    0.139    0.163
##    .dCRET20_pldr_y     0.225    0.004   53.012    0.000    0.217    0.233
##    .cr_media           1.543    0.076   20.366    0.000    1.395    1.692
##    .cr_socialmedia     0.373    0.035   10.672    0.000    0.305    0.442
##    .dCRET20_rolm_y     0.200    0.005   36.287    0.000    0.189    0.210
##    .dCRET20_clbm_y     0.202    0.005   37.537    0.000    0.191    0.212
##    .cr_gndrnrmsc_1     2.848    0.160   17.854    0.000    2.535    3.161
##    .cr_gndrnrmsc_2     3.068    0.144   21.309    0.000    2.786    3.351
##    .cr_gndrnrmsc_3     1.841    0.070   26.283    0.000    1.703    1.978
##    .cr_gndrnrmsc_4     2.435    0.126   19.307    0.000    2.188    2.683
##    .cr_gendrdctvts     2.690    0.175   15.403    0.000    2.348    3.032
##    .cr_ghq12          13.443    1.010   13.312    0.000   11.464   15.422
##    .dCRET20_female     0.229    0.004   64.598    0.000    0.222    0.236
##    .Competencies       0.290    0.042    6.942    0.000    0.208    0.372
##    .GenderNorms        0.336    0.034    9.945    0.000    0.270    0.402
##    .GenderAttituds     1.118    0.143    7.793    0.000    0.837    1.399
##    Std.lv  Std.all
##     0.757    0.560
##     9.018    0.595
##    20.892    0.844
##     0.151    0.898
##     0.225    0.914
##     1.543    0.627
##     0.373    0.866
##     0.200    0.801
##     0.202    0.815
##     2.848    0.576
##     3.068    0.699
##     1.841    0.871
##     2.435    0.663
##     2.690    0.627
##    13.443    0.962
##     0.229    0.922
##     0.488    0.488
##     0.366    0.366
##     0.534    0.534
##
## R-Square:
##                     Estimate
##     cr_talk            0.440
##     cr_copingrslnc     0.405
##     cr_voice           0.156
##     dCRET20_pfrnd_     0.102
##     dCRET20_pldr_y     0.086
##     cr_media           0.373
##     cr_socialmedia     0.134
##     dCRET20_rolm_y     0.199
##     dCRET20_clbm_y     0.185
##     cr_gndrnrmsc_1     0.424
##     cr_gndrnrmsc_2     0.301
##     cr_gndrnrmsc_3     0.129
##     cr_gndrnrmsc_4     0.337
```

```
##     cr_gendrdctvts    0.373
##     cr_ghq12          0.038
##     dCRET20_female    0.078
##     Competencies      0.512
##     GenderNorms       0.634
##     GenderAttituds    0.466
##
## Defined Parameters:
##                    Estimate  Std.Err  z-value  P(>|z|) ci.lower ci.upper
##     partGNGAMH       -0.636    0.722   -0.881    0.378   -2.050    0.779
##     partGNGRMH        0.048    0.101    0.478    0.633   -0.150    0.246
##     partGNGRCOM      -0.058    0.030   -1.916    0.055   -0.117    0.001
##     partSexGAMH      -0.250    0.288   -0.867    0.386   -0.816    0.315
##     tieGNMH          -0.587    0.763   -0.769    0.442   -2.084    0.909
##     tieGNCOM         -0.058    0.030   -1.916    0.055   -0.117    0.001
##     tieSexMH         -0.250    0.288   -0.867    0.386   -0.816    0.315
##     teSexMH          -0.567    0.220   -2.577    0.010   -0.998   -0.136
##     teGNMH           -0.087    0.240   -0.360    0.719   -0.557    0.384
##     teGNCOM           0.571    0.051   11.147    0.000    0.471    0.672
##     med_propSexMH     0.441    0.542    0.815    0.415   -0.620    1.503
##     med_propGNGAMH    7.344   22.744    0.323    0.747  -37.233   51.921
##     med_propGNGRMH   -0.558    1.620   -0.345    0.730   -3.734    2.618
##     med_prpGNGRCOM   -0.101    0.050   -2.026    0.043   -0.199   -0.003
##    Std.lv  Std.all
##    -0.609   -0.163
##     0.046    0.012
##    -0.072   -0.072
##    -0.250   -0.033
##    -0.563   -0.151
##    -0.072   -0.072
##    -0.250   -0.033
##    -0.567   -0.075
##    -0.083   -0.022
##     0.710    0.710
##     0.441    0.441
##     7.344    7.344
##    -0.558   -0.558
##    -0.101   -0.101
```

```
#fitMeasures(fit.testmPUBSub)

#parameterEstimates(fit.testmPUBSub, standardized = TRUE)
#modindices(fit.testmPUBSub, sort. = TRUE)
```

7.1.1 Sensitivity analysis exclusion of variables below

CFA<0.4

```
testmPUBCFA <- '
Competencies =~ cr_talk + cr_copingresilience #+  cr_voice + dCRET20_opfriend_y + dCRET
20_opelder_y
GenderNorms =~ cr_media  + dCRET20_rolem_y + dCRET20_clubm_y #+ peervio_ordinal + cr_so
cialmedia
GenderAttitudes =~ cr_gendernormscale_f1 + cr_gendernormscale_f2 +  cr_gendernormscale_
f4 #+ cr_gendernormscale_f5 + cr_gendernormscale_f6 + cr_gendernormscale_f7 cr_genderno
rmscale_f3 +

#mediations/regressions
GenderAttitudes ~ aGN_GA * GenderNorms + aSex_GA * dCRET20_female
cr_genderedactivities ~ aGN_GR * GenderNorms
cr_ghq12~ bGA_MH * GenderAttitudes + bGR_MH * cr_genderedactivities + cSexMH * dCRET20_
female + cGNMH * GenderNorms + Competencies
Competencies ~ bGR_COM * cr_genderedactivities + cGNCOM * GenderNorms#new

#covariances to improve model fit based on modindices
#cr_copingresilience~~ cr_genderedactivities
GenderNorms ~~  GenderAttitudes
dCRET20_opfriend_y ~~ dCRET20_opelder_y
cr_genderedactivities ~~ dCRET20_female
GenderAttitudes ~~ cr_ghq12
dCRET20_rolem_y ~~ cr_genderedactivities

#partial indirect effects
partGNGAMH := aGN_GA * bGA_MH
partGNGRMH := aGN_GR * bGR_MH
partGNGRCOM  := aGN_GR * bGR_COM
partSexGAMH := aSex_GA * bGA_MH

#total indirect effects
tieGNMH := partGNGAMH + partGNGRMH
tieGNCOM := partGNGRCOM
tieSexMH:= partSexGAMH

#total effect
teSexMH := tieSexMH + cSexMH
teGNMH := tieGNMH + cGNMH
teGNCOM := tieGNCOM + cGNCOM#new

med_propSexMH := partSexGAMH / teSexMH
med_propGNGAMH := partGNGAMH / teGNMH
med_propGNGRMH := partGNGRMH / teGNMH
med_propGNGRCOM := partGNGRCOM / teGNCOM

#covariates to the two exogeneous variables (or to the ones the covariates apply)
dCRET20_female~cr_cs_crage + dCRET20_urban + dCRET20_married_y + dCRET20_purpsamp_y + d
CRET20_disability_y + af_asset_10 + dCRET20_fatherinhh +  dCRET20_aspiratt_sec +
dCRET20_aspiratt_teach + dCRET20_aspiratt_uni +  dCRET20_languagehh_201 + dCRET20_langu
agehh_202 + dCRET20_languagehh_211  + dCRET20_workfambus_y

GenderNorms~cr_cs_crage + dCRET20_urban + dCRET20_married_y +dCRET20_purpsamp_y + dCRET
20_disability_y + af_asset_10 + dCRET20_fatherinhh +  dCRET20_aspiratt_sec +
```

```
dCRET20_aspiratt_teach + dCRET20_aspiratt_uni +  dCRET20_languagehh_201 + dCRET20_langu
agehh_202 + dCRET20_languagehh_211  + dCRET20_workfambus_y
'

fit.testmPUBCFA <- sem(testmPUBCFA, data=subsetSEMNeu, estimator="MLR", sampling.weight
s = "rand_weight")
summary(fit.testmPUBCFA, fit.measures=TRUE, standardized=TRUE, rsq=TRUE, ci=TRUE)
```

```
## lavaan 0.6-21 ended normally after 124 iterations
##
##   Estimator                                         ML
##   Optimization method                           NLMINB
##   Number of model parameters                        64
##
##                                                   Used       Total
##   Number of observations                          1650        4777
##   Sampling weights variable                    rand_weight
##
## Model Test User Model:
##                                               Standard      Scaled
##   Test Statistic                              1479.183    1250.023
##   Degrees of freedom                               209         209
##   P-value (Chi-square)                           0.000       0.000
##   Scaling correction factor                                  1.183
##     Yuan-Bentler correction (Mplus variant)
##
## Model Test Baseline Model:
##
##   Test statistic                              5469.308    4612.530
##   Degrees of freedom                               260         260
##   P-value                                        0.000       0.000
##   Scaling correction factor                                  1.186
##
## User Model versus Baseline Model:
##
##   Comparative Fit Index (CFI)                    0.756       0.761
##   Tucker-Lewis Index (TLI)                       0.697       0.702
##
##   Robust Comparative Fit Index (CFI)                         0.761
##   Robust Tucker-Lewis Index (TLI)                            0.703
##
## Loglikelihood and Information Criteria:
##
##   Loglikelihood user model (H0)             -32640.942  -32640.942
##   Scaling correction factor                                  1.165
##       for the MLR correction
##   Loglikelihood unrestricted model (H1)     -31901.350  -31901.350
##   Scaling correction factor                                  1.179
##       for the MLR correction
##
##   Akaike (AIC)                               65409.883   65409.883
##   Bayesian (BIC)                             65756.029   65756.029
##   Sample-size adjusted Bayesian (SABIC)      65552.711   65552.711
##
## Root Mean Square Error of Approximation:
##
##   RMSEA                                          0.061       0.055
##   90 Percent confidence interval - lower         0.058       0.052
##   90 Percent confidence interval - upper         0.064       0.058
##   P-value H_0: RMSEA <= 0.050                    0.000       0.001
##   P-value H_0: RMSEA >= 0.080                    0.000       0.000
##
##   Robust RMSEA                                               0.060
```

```
##   90 Percent confidence interval - lower                     0.057
##   90 Percent confidence interval - upper                     0.063
##   P-value H_0: Robust RMSEA <= 0.050                         0.000
##   P-value H_0: Robust RMSEA >= 0.080                         0.000
##
## Standardized Root Mean Square Residual:
##
##   SRMR                                          0.052       0.052
##
## Parameter Estimates:
##
##   Standard errors                             Sandwich
##   Information bread                           Observed
##   Observed information based on                Hessian
##
## Latent Variables:
##                      Estimate  Std.Err  z-value  P(>|z|) ci.lower ci.upper
##   Competencies =~
##     cr_talk             1.000                               1.000    1.000
##     cr_copingrslnc      3.505    0.259   13.511    0.000    2.997    4.014
##   GenderNorms =~
##     cr_media            1.000                               1.000    1.000
##     dCRET20_rolm_y      0.248    0.018   13.726    0.000    0.213    0.283
##     dCRET20_clbm_y      0.243    0.017   14.336    0.000    0.209    0.276
##   GenderAttitudes =~
##     cr_gndrnrmsc_1      1.000                               1.000    1.000
##     cr_gndrnrmsc_2      0.823    0.057   14.435    0.000    0.711    0.935
##     cr_gndrnrmsc_4      0.820    0.061   13.472    0.000    0.700    0.939
##    Std.lv  Std.all
##
##     0.707    0.603
##     2.478    0.635
##
##     0.909    0.581
##     0.225    0.452
##     0.221    0.444
##
##     1.380    0.622
##     1.136    0.543
##     1.131    0.591
##
## Regressions:
##                           Estimate  Std.Err  z-value  P(>|z|) ci.lower ci.upper
##   GenderAttitudes ~
##     GndrN (aGN_GA)           1.351    0.110   12.248    0.000    1.134    1.567
##     dCRET   (aS_G)           0.543    0.084    6.457    0.000    0.378    0.707
##   cr_genderedactivities ~
##     GndrN (aGN_GR)          -1.486    0.094  -15.774    0.000   -1.670   -1.301
##   cr_ghq12 ~
##     GndrA   (bGA_)          -0.423    0.481   -0.879    0.380   -1.365    0.520
##     cr_gn  (bGR_M)           0.008    0.093    0.090    0.928   -0.174    0.191
##     dCRET   (cSMH)          -0.330    0.337   -0.981    0.326   -0.990    0.329
##     GndrN   (cGNM)           1.127    0.941    1.198    0.231   -0.717    2.972
##     Cmptn                   -2.047    0.658   -3.113    0.002   -3.336   -0.758
##   Competencies ~
##     cr_gn  (bGR_C)           0.051    0.022    2.263    0.024    0.007    0.094
```

```
##     GndrN   (cGNC)           0.700    0.078    8.989    0.000    0.548    0.853
##   dCRET20_female ~
##     cr_c_                   -0.028    0.014   -2.030    0.042   -0.055   -0.001
##     dCRET                   -0.034    0.027   -1.267    0.205   -0.086    0.018
##     dCRET                   -0.269    0.056   -4.782    0.000   -0.379   -0.159
##     dCRET                    0.058    0.064    0.913    0.361   -0.067    0.183
##     dCRET                    0.075    0.055    1.364    0.173   -0.033    0.183
##     a__10                   -0.004    0.005   -0.752    0.452   -0.014    0.006
##     dCRET                   -0.040    0.029   -1.383    0.167   -0.097    0.017
##     dCRET                   -0.025    0.057   -0.436    0.663   -0.138    0.088
##     dCRET                   -0.189    0.078   -2.416    0.016   -0.342   -0.036
##     dCRET                   -0.269    0.056   -4.840    0.000   -0.377   -0.160
##     dCRET                   -0.110    0.029   -3.817    0.000   -0.167   -0.054
##     dCRET                   -0.080    0.040   -2.008    0.045   -0.159   -0.002
##     dCRET                    0.038    0.102    0.370    0.711   -0.162    0.237
##     dCRET                   -0.093    0.056   -1.664    0.096   -0.203    0.017
##   GenderNorms ~
##     cr_c_                    0.052    0.022    2.331    0.020    0.008    0.096
##     dCRET                   -0.238    0.049   -4.876    0.000   -0.334   -0.142
##     dCRET                    0.742    0.125    5.924    0.000    0.497    0.988
##     dCRET                    0.054    0.102    0.533    0.594   -0.145    0.253
##     dCRET                    0.233    0.077    3.024    0.002    0.082    0.385
##     a__10                    0.110    0.011    9.663    0.000    0.088    0.133
##     dCRET                   -0.032    0.044   -0.715    0.474   -0.119    0.055
##     dCRET                    0.202    0.104    1.950    0.051   -0.001    0.405
##     dCRET                    0.381    0.121    3.136    0.002    0.143    0.619
##     dCRET                    0.867    0.103    8.454    0.000    0.666    1.068
##     dCRET                   -0.566    0.047  -11.999    0.000   -0.659   -0.474
##     dCRET                   -0.517    0.060   -8.687    0.000   -0.634   -0.401
##     dCRET                   -0.218    0.148   -1.475    0.140   -0.508    0.072
##     dCRET                   -0.165    0.078   -2.118    0.034   -0.318   -0.012
##    Std.lv  Std.all
##
##     0.890    0.890
##     0.393    0.196
##
##    -1.351   -0.634
##
##    -0.583   -0.157
##     0.008    0.005
##    -0.330   -0.044
##     1.025    0.276
##    -1.447   -0.389
##
##     0.072    0.152
##     0.901    0.901
##
##    -0.028   -0.052
##    -0.034   -0.034
##    -0.269   -0.106
##     0.058    0.025
##     0.075    0.036
##    -0.004   -0.021
##    -0.040   -0.034
##    -0.025   -0.021
##    -0.189   -0.083
```

```
##    -0.269   -0.258
##    -0.110   -0.103
##    -0.080   -0.058
##     0.038    0.009
##    -0.093   -0.041
##
##     0.057    0.053
##    -0.262   -0.130
##     0.817    0.160
##     0.059    0.013
##     0.257    0.062
##     0.121    0.331
##    -0.035   -0.015
##     0.222    0.094
##     0.419    0.092
##     0.953    0.455
##    -0.623   -0.288
##    -0.569   -0.205
##    -0.240   -0.028
##    -0.182   -0.040
##
## Covariances:
##                              Estimate  Std.Err  z-value  P(>|z|) ci.lower
##   .GenderNorms ~~
##    .GenderAttituds             -0.233    0.053   -4.385    0.000   -0.337
##   dCRET20_opfriend_y ~~
##     dCRET20_pldr_y              0.062    0.005   12.875    0.000    0.052
##   .cr_genderedactivities ~~
##    .dCRET20_female              0.288    0.024   11.769    0.000    0.240
##   .GenderAttitudes ~~
##    .cr_ghq12                    0.502    0.387    1.297    0.195   -0.257
##   .dCRET20_rolem_y ~~
##    .cr_gendrdctvts              0.114    0.023    4.919    0.000    0.068
##  ci.upper   Std.lv  Std.all
##
##    -0.129   -0.415   -0.415
##
##     0.071    0.062    0.302
##
##     0.336    0.288    0.368
##
##     1.261    0.497    0.138
##
##     0.159    0.114    0.155
##
## Variances:
##                    Estimate  Std.Err  z-value  P(>|z|) ci.lower ci.upper
##    .cr_talk           0.876    0.048   18.276    0.000    0.782    0.970
##    .cr_copingrslnc    9.063    0.593   15.281    0.000    7.901   10.226
##    .cr_media          1.621    0.072   22.402    0.000    1.480    1.763
##    .dCRET20_rolm_y    0.198    0.006   35.888    0.000    0.187    0.209
##    .dCRET20_clbm_y    0.199    0.005   38.167    0.000    0.188    0.209
##    .cr_gndrnrmsc_1    3.025    0.156   19.448    0.000    2.720    3.330
##    .cr_gndrnrmsc_2    3.086    0.145   21.350    0.000    2.803    3.369
##    .cr_gndrnrmsc_4    2.378    0.126   18.918    0.000    2.132    2.625
##    .cr_gendrdctvts    2.709    0.178   15.243    0.000    2.360    3.057
```

```
##    .cr_ghq12         12.974    0.946   13.722    0.000   11.121   14.827
##    .dCRET20_female    0.226    0.004   64.626    0.000    0.220    0.233
##     dCRET20_pfrnd_    0.170    0.006   27.615    0.000    0.158    0.182
##     dCRET20_pldr_y    0.246    0.002  141.503    0.000    0.242    0.249
##    .Competencies      0.170    0.036    4.670    0.000    0.098    0.241
##    .GenderNorms       0.309    0.032    9.674    0.000    0.246    0.371
##    .GenderAttituds    1.020    0.129    7.913    0.000    0.767    1.273
##    Std.lv  Std.all
##     0.876    0.637
##     9.063    0.596
##     1.621    0.662
##     0.198    0.796
##     0.199    0.803
##     3.025    0.614
##     3.086    0.705
##     2.378    0.650
##     2.709    0.598
##    12.974    0.937
##     0.226    0.915
##     0.170    1.000
##     0.246    1.000
##     0.340    0.340
##     0.374    0.374
##     0.535    0.535
##
## R-Square:
##                   Estimate
##     cr_talk          0.363
##     cr_copingrslnc    0.404
##     cr_media         0.338
##     dCRET20_rolm_y    0.204
##     dCRET20_clbm_y    0.197
##     cr_gndrnrmsc_1    0.386
##     cr_gndrnrmsc_2    0.295
##     cr_gndrnrmsc_4    0.350
##     cr_gendrdctvts    0.402
##     cr_ghq12         0.063
##     dCRET20_female    0.085
##     Competencies      0.660
##     GenderNorms       0.626
##     GenderAttituds    0.465
##
## Defined Parameters:
##                   Estimate  Std.Err  z-value  P(>|z|) ci.lower ci.upper
##     partGNGAMH       -0.571    0.648   -0.881    0.378   -1.841    0.699
##     partGNGRMH       -0.012    0.138   -0.090    0.928   -0.283    0.258
##     partGNGRCOM      -0.075    0.034   -2.183    0.029   -0.143   -0.008
##     partSexGAMH      -0.229    0.263   -0.872    0.383   -0.745    0.286
##     tieGNMH          -0.583    0.716   -0.815    0.415   -1.986    0.819
##     tieGNCOM         -0.075    0.034   -2.183    0.029   -0.143   -0.008
##     tieSexMH         -0.229    0.263   -0.872    0.383   -0.745    0.286
##     teSexMH          -0.560    0.219   -2.554    0.011   -0.989   -0.130
##     teGNMH            0.544    0.423    1.287    0.198   -0.284    1.373
##     teGNCOM           0.625    0.056   11.105    0.000    0.515    0.735
##     med_propSexMH     0.410    0.491    0.834    0.404   -0.553    1.373
##     med_propGNGAMH   -1.049    1.277   -0.822    0.411   -3.551    1.453
```

```
##     med_propGNGRMH   -0.023    0.244   -0.094    0.925   -0.501    0.456
##     med_prpGNGRCOM   -0.120    0.051   -2.348    0.019   -0.220   -0.020
##    Std.lv  Std.all
##    -0.519   -0.140
##    -0.011   -0.003
##    -0.097   -0.097
##    -0.229   -0.031
##    -0.530   -0.143
##    -0.097   -0.097
##    -0.229   -0.031
##    -0.560   -0.075
##     0.495    0.133
##     0.804    0.804
##     0.410    0.410
##    -1.049   -1.049
##    -0.023   -0.023
##    -0.120   -0.120
```

```
#fitMeasures(fit.testmNEUPUB)

#parameterEstimates(fit.testmNEUPUB, standardized = TRUE)
#modindices(fit.testmNEUPUB, sort. = TRUE)
```

7.1.2 Sensitivity analysis SEM in submission for the Gender

norms construct

```
#excluding club member and role model

testmNEUPUB <- '
Competencies =~ cr_talk + cr_copingresilience +  cr_voice + dCRET20_opfriend_y + dCRET2
0_opelder_y
GenderNorms =~ cr_media  + cr_socialmedia #+ dCRET20_rolem_y + dCRET20_clubm_y
GenderAttitudes =~ cr_gendernormscale_f1 + cr_gendernormscale_f2 + cr_gendernormscale_f
3 + cr_gendernormscale_f4

#mediations/regressions
GenderAttitudes ~ aGN_GA * GenderNorms + aSex_GA * dCRET20_female
cr_genderedactivities ~ aGN_GR * GenderNorms
cr_ghq12~ bGA_MH * GenderAttitudes + bGR_MH * cr_genderedactivities + cSexMH * dCRET20_
female + cGNMH * GenderNorms + Competencies
Competencies ~ bGR_COM * cr_genderedactivities + cGNCOM * GenderNorms#new

#covariances to improve model fit based on modindices
#cr_copingresilience~~ cr_genderedactivities
GenderNorms ~~  GenderAttitudes
dCRET20_opfriend_y ~~ dCRET20_opelder_y
cr_genderedactivities ~~ dCRET20_female
GenderAttitudes ~~ cr_ghq12
dCRET20_rolem_y ~~ cr_genderedactivities

#partial indirect effects
partGNGAMH := aGN_GA * bGA_MH
partGNGRMH := aGN_GR * bGR_MH
partGNGRCOM  := aGN_GR * bGR_COM
partSexGAMH := aSex_GA * bGA_MH

#total indirect effects
tieGNMH := partGNGAMH + partGNGRMH
tieGNCOM := partGNGRCOM
tieSexMH:= partSexGAMH

#total effect
teSexMH := tieSexMH + cSexMH
teGNMH := tieGNMH + cGNMH
teGNCOM := tieGNCOM + cGNCOM#new

med_propSexMH := partSexGAMH / teSexMH
med_propGNGAMH := partGNGAMH / teGNMH
med_propGNGRMH := partGNGRMH / teGNMH
med_propGNGRCOM := partGNGRCOM / teGNCOM

#covariates to the two exogeneous variables (or to the ones the covariates apply)
dCRET20_female~cr_cs_crage + dCRET20_urban + dCRET20_married_y + dCRET20_purpsamp_y + d
CRET20_disability_y + af_asset_10 + dCRET20_fatherinhh +  dCRET20_aspiratt_sec +
dCRET20_aspiratt_teach + dCRET20_aspiratt_uni +  dCRET20_languagehh_201 + dCRET20_langu
agehh_202 + dCRET20_languagehh_211  + dCRET20_workfambus_y

GenderNorms~cr_cs_crage + dCRET20_urban + dCRET20_married_y +dCRET20_purpsamp_y + dCRET
20_disability_y + af_asset_10 + dCRET20_fatherinhh +  dCRET20_aspiratt_sec +
```

```
dCRET20_aspiratt_teach + dCRET20_aspiratt_uni +  dCRET20_languagehh_201 + dCRET20_langu
agehh_202 + dCRET20_languagehh_211  + dCRET20_workfambus_y
'
fit.testmNEUPUB <- sem(testmNEUPUB, data=subsetSEMNeu, estimator="MLR", sampling.weight
s = "rand_weight")
summary(fit.testmNEUPUB, fit.measures=TRUE, standardized=TRUE, rsq=TRUE, ci=TRUE)
```

```
## lavaan 0.6-21 ended normally after 124 iterations
##
##   Estimator                                         ML
##   Optimization method                           NLMINB
##   Number of model parameters                        69
##
##                                                   Used       Total
##   Number of observations                          1584        4777
##   Sampling weights variable                 rand_weight
##
## Model Test User Model:
##                                               Standard      Scaled
##   Test Statistic                              1751.559    1508.807
##   Degrees of freedom                               261         261
##   P-value (Chi-square)                           0.000       0.000
##   Scaling correction factor                                  1.161
##     Yuan-Bentler correction (Mplus variant)
##
## Model Test Baseline Model:
##
##   Test statistic                              5704.377    4901.911
##   Degrees of freedom                               315         315
##   P-value                                        0.000       0.000
##   Scaling correction factor                                  1.164
##
## User Model versus Baseline Model:
##
##   Comparative Fit Index (CFI)                    0.723       0.728
##   Tucker-Lewis Index (TLI)                       0.666       0.672
##
##   Robust Comparative Fit Index (CFI)                         0.729
##   Robust Tucker-Lewis Index (TLI)                            0.672
##
## Loglikelihood and Information Criteria:
##
##   Loglikelihood user model (H0)             -39292.713  -39292.713
##   Scaling correction factor                                  1.255
##       for the MLR correction
##   Loglikelihood unrestricted model (H1)     -38416.934  -38416.934
##   Scaling correction factor                                  1.181
##       for the MLR correction
##
##   Akaike (AIC)                               78723.426   78723.426
##   Bayesian (BIC)                             79093.798   79093.798
##   Sample-size adjusted Bayesian (SABIC)      78874.599   78874.599
##
## Root Mean Square Error of Approximation:
##
##   RMSEA                                          0.060       0.055
##   90 Percent confidence interval - lower         0.057       0.052
##   90 Percent confidence interval - upper         0.063       0.057
##   P-value H_0: RMSEA <= 0.050                    0.000       0.001
##   P-value H_0: RMSEA >= 0.080                    0.000       0.000
##
##   Robust RMSEA                                               0.059
```

```
##   90 Percent confidence interval - lower                     0.056
##   90 Percent confidence interval - upper                     0.062
##   P-value H_0: Robust RMSEA <= 0.050                         0.000
##   P-value H_0: Robust RMSEA >= 0.080                         0.000
##
## Standardized Root Mean Square Residual:
##
##   SRMR                                          0.057       0.057
##
## Parameter Estimates:
##
##   Standard errors                             Sandwich
##   Information bread                           Observed
##   Observed information based on                Hessian
##
## Latent Variables:
##                      Estimate  Std.Err  z-value  P(>|z|) ci.lower ci.upper
##   Competencies =~
##     cr_talk             1.000                               1.000    1.000
##     cr_copingrslnc      3.228    0.314   10.273    0.000    2.612    3.844
##     cr_voice            2.574    0.181   14.225    0.000    2.219    2.929
##     dCRET20_pfrnd_      0.172    0.021    8.049    0.000    0.130    0.214
##     dCRET20_pldr_y      0.190    0.021    8.938    0.000    0.149    0.232
##   GenderNorms =~
##     cr_media            1.000                               1.000    1.000
##     cr_socialmedia      0.263    0.022   11.729    0.000    0.219    0.307
##   GenderAttitudes =~
##     cr_gndrnrmsc_1      1.000                               1.000    1.000
##     cr_gndrnrmsc_2      0.831    0.057   14.645    0.000    0.720    0.942
##     cr_gndrnrmsc_3      0.371    0.032   11.417    0.000    0.307    0.434
##     cr_gndrnrmsc_4      0.806    0.063   12.868    0.000    0.683    0.928
##    Std.lv  Std.all
##
##     0.766    0.660
##     2.474    0.636
##     1.973    0.397
##     0.132    0.321
##     0.146    0.294
##
##     0.977    0.623
##     0.257    0.391
##
##     1.404    0.632
##     1.167    0.557
##     0.520    0.358
##     1.131    0.591
##
## Regressions:
##                           Estimate  Std.Err  z-value  P(>|z|) ci.lower ci.upper
##   GenderAttitudes ~
##     GndrN (aGN_GA)           1.142    0.089   12.876    0.000    0.968    1.316
##     dCRET   (aS_G)           0.457    0.085    5.392    0.000    0.291    0.624
##   cr_genderedactivities ~
##     GndrN (aGN_GR)          -1.293    0.079  -16.414    0.000   -1.448   -1.139
##   cr_ghq12 ~
##     GndrA   (bGA_)          -0.385    1.087   -0.355    0.723   -2.516    1.745
```

```
##      cr_gn  (bGR_M)          -0.079    0.078   -1.007    0.314   -0.233    0.075
##      dCRET   (cSMH)          -0.365    0.530   -0.689    0.491   -1.404    0.674
##      GndrN   (cGNM)           0.174    1.296    0.134    0.893   -2.366    2.713
##      Cmptn                   -0.976    0.323   -3.020    0.003   -1.609   -0.342
##    Competencies ~
##      cr_gn  (bGR_C)           0.003    0.022    0.121    0.903   -0.040    0.045
##      GndrN   (cGNC)           0.486    0.059    8.271    0.000    0.371    0.601
##    dCRET20_female ~
##      cr_c_                   -0.028    0.014   -1.985    0.047   -0.055   -0.000
##      dCRET                   -0.026    0.027   -0.934    0.350   -0.079    0.028
##      dCRET                   -0.282    0.057   -4.931    0.000   -0.394   -0.170
##      dCRET                    0.079    0.068    1.163    0.245   -0.054    0.212
##      dCRET                    0.081    0.056    1.451    0.147   -0.028    0.190
##      a__10                   -0.005    0.005   -1.042    0.297   -0.015    0.005
##      dCRET                   -0.032    0.030   -1.073    0.283   -0.090    0.026
##      dCRET                   -0.014    0.059   -0.234    0.815   -0.130    0.102
##      dCRET                   -0.202    0.081   -2.509    0.012   -0.360   -0.044
##      dCRET                   -0.251    0.057   -4.378    0.000   -0.364   -0.139
##      dCRET                   -0.106    0.029   -3.611    0.000   -0.163   -0.048
##      dCRET                   -0.100    0.041   -2.407    0.016   -0.181   -0.019
##      dCRET                   -0.011    0.099   -0.110    0.913   -0.205    0.183
##      dCRET                   -0.106    0.058   -1.833    0.067   -0.220    0.007
##    GenderNorms ~
##      cr_c_                    0.066    0.025    2.666    0.008    0.017    0.114
##      dCRET                   -0.345    0.057   -6.085    0.000   -0.456   -0.234
##      dCRET                    0.772    0.138    5.591    0.000    0.502    1.043
##      dCRET                    0.017    0.110    0.150    0.881   -0.200    0.233
##      dCRET                    0.197    0.094    2.086    0.037    0.012    0.382
##      a__10                    0.146    0.013   11.516    0.000    0.121    0.171
##      dCRET                   -0.117    0.053   -2.220    0.026   -0.220   -0.014
##      dCRET                    0.217    0.115    1.890    0.059   -0.008    0.442
##      dCRET                    0.554    0.131    4.227    0.000    0.297    0.811
##      dCRET                    0.918    0.111    8.263    0.000    0.700    1.136
##      dCRET                   -0.604    0.052  -11.584    0.000   -0.706   -0.502
##      dCRET                   -0.406    0.073   -5.590    0.000   -0.548   -0.263
##      dCRET                   -0.073    0.185   -0.395    0.692   -0.437    0.290
##      dCRET                   -0.172    0.091   -1.900    0.057   -0.350    0.005
##     Std.lv  Std.all
##
##      0.795    0.795
##      0.326    0.162
##
##     -1.264   -0.606
##
##     -0.541   -0.145
##     -0.079   -0.044
##     -0.365   -0.049
##      0.170    0.045
##     -0.748   -0.200
##
##      0.003    0.007
##      0.619    0.619
##
##     -0.028   -0.051
##     -0.026   -0.026
##     -0.282   -0.096
```

```
##     0.079    0.030
##     0.081    0.039
##    -0.005   -0.029
##    -0.032   -0.027
##    -0.014   -0.012
##    -0.202   -0.089
##    -0.251   -0.240
##    -0.106   -0.098
##    -0.100   -0.072
##    -0.011   -0.003
##    -0.106   -0.047
##
##     0.068    0.063
##    -0.353   -0.176
##     0.790    0.134
##     0.017    0.003
##     0.202    0.049
##     0.150    0.410
##    -0.119   -0.050
##     0.222    0.093
##     0.567    0.124
##     0.939    0.446
##    -0.618   -0.286
##    -0.415   -0.149
##    -0.075   -0.009
##    -0.176   -0.039
##
## Covariances:
##                              Estimate  Std.Err  z-value  P(>|z|) ci.lower
##   .GenderNorms ~~
##     .GenderAttituds            -0.117    0.058   -2.020    0.043   -0.231
##   .dCRET20_opfriend_y ~~
##     .dCRET20_pldr_y             0.041    0.005    8.328    0.000    0.031
##   .cr_genderedactivities ~~
##     .dCRET20_female             0.297    0.024   12.339    0.000    0.250
##   .GenderAttitudes ~~
##     .cr_ghq12                   0.401    0.963    0.416    0.677   -1.487
##   .cr_genderedactivities ~~
##     dCRET20_rolm_y              0.023    0.026    0.908    0.364   -0.027
##   ci.upper   Std.lv  Std.all
##
##    -0.003   -0.221   -0.221
##
##     0.051    0.041    0.223
##
##     0.344    0.297    0.374
##
##     2.289    0.403    0.110
##
##     0.073    0.023    0.028
##
## Variances:
##                    Estimate  Std.Err  z-value  P(>|z|) ci.lower ci.upper
##    .cr_talk           0.763    0.062   12.402    0.000    0.642    0.883
##    .cr_copingrslnc    9.007    0.669   13.469    0.000    7.696   10.318
##    .cr_voice         20.854    0.871   23.933    0.000   19.146   22.562
```

```
##    .dCRET20_pfrnd_    0.151    0.006   24.302    0.000    0.139    0.163
##    .dCRET20_pldr_y    0.225    0.004   52.113    0.000    0.216    0.233
##    .cr_media          1.507    0.077   19.565    0.000    1.356    1.658
##    .cr_socialmedia    0.366    0.034   10.703    0.000    0.299    0.433
##    .cr_gndrnrmsc_1    2.960    0.161   18.369    0.000    2.644    3.275
##    .cr_gndrnrmsc_2    3.020    0.144   20.969    0.000    2.738    3.303
##    .cr_gndrnrmsc_3    1.842    0.070   26.457    0.000    1.706    1.979
##    .cr_gndrnrmsc_4    2.387    0.128   18.583    0.000    2.135    2.638
##    .cr_gendrdctvts    2.754    0.181   15.193    0.000    2.399    3.109
##    .cr_ghq12         13.363    1.119   11.946    0.000   11.171   15.556
##    .dCRET20_female    0.228    0.004   64.823    0.000    0.222    0.235
##     dCRET20_rolm_y    0.249    0.001  284.981    0.000    0.247    0.251
##    .Competencies      0.365    0.049    7.418    0.000    0.269    0.462
##    .GenderNorms       0.284    0.039    7.216    0.000    0.207    0.361
##    .GenderAttituds    0.988    0.139    7.115    0.000    0.716    1.260
##    Std.lv  Std.all
##     0.763    0.565
##     9.007    0.595
##    20.854    0.843
##     0.151    0.897
##     0.225    0.914
##     1.507    0.612
##     0.366    0.847
##     2.960    0.600
##     3.020    0.689
##     1.842    0.872
##     2.387    0.651
##     2.754    0.633
##    13.363    0.957
##     0.228    0.923
##     0.249    1.000
##     0.622    0.622
##     0.298    0.298
##     0.501    0.501
##
## R-Square:
##                    Estimate
##     cr_talk           0.435
##     cr_copingrslnc    0.405
##     cr_voice          0.157
##     dCRET20_pfrnd_    0.103
##     dCRET20_pldr_y    0.086
##     cr_media          0.388
##     cr_socialmedia    0.153
##     cr_gndrnrmsc_1    0.400
##     cr_gndrnrmsc_2    0.311
##     cr_gndrnrmsc_3    0.128
##     cr_gndrnrmsc_4    0.349
##     cr_gendrdctvts    0.367
##     cr_ghq12          0.043
##     dCRET20_female    0.077
##     Competencies      0.378
##     GenderNorms       0.702
##     GenderAttituds    0.499
##
## Defined Parameters:
```

```
##                      Estimate  Std.Err  z-value  P(>|z|) ci.lower ci.upper
##     partGNGAMH        -0.440    1.239   -0.355    0.722   -2.869    1.989
##     partGNGRMH         0.102    0.102    1.004    0.315   -0.097    0.302
##     partGNGRCOM       -0.003    0.028   -0.121    0.904   -0.059    0.052
##     partSexGAMH       -0.176    0.498   -0.354    0.723   -1.153    0.800
##     tieGNMH           -0.338    1.289   -0.262    0.793   -2.865    2.189
##     tieGNCOM          -0.003    0.028   -0.121    0.904   -0.059    0.052
##     tieSexMH          -0.176    0.498   -0.354    0.723   -1.153    0.800
##     teSexMH           -0.542    0.222   -2.436    0.015   -0.977   -0.106
##     teGNMH            -0.164    0.182   -0.903    0.367   -0.520    0.192
##     teGNCOM            0.482    0.045   10.710    0.000    0.394    0.570
##     med_propSexMH      0.326    0.920    0.354    0.723   -1.477    2.128
##     med_propGNGAMH     2.681    7.994    0.335    0.737  -12.986   18.349
##     med_propGNGRMH    -0.623    0.893   -0.697    0.486   -2.373    1.127
##     med_prpGNGRCOM    -0.007    0.058   -0.122    0.903   -0.121    0.107
##    Std.lv  Std.all
##    -0.430   -0.115
##     0.100    0.027
##    -0.004   -0.004
##    -0.176   -0.023
##    -0.330   -0.088
##    -0.004   -0.004
##    -0.176   -0.023
##    -0.542   -0.072
##    -0.160   -0.043
##     0.615    0.615
##     0.326    0.326
##     2.681    2.681
##    -0.623   -0.623
##    -0.007   -0.007
```

```
#fitMeasures(fit.testmNEUPUB)

#parameterEstimates(fit.testmNEUPUB, standardized = TRUE)
#modindices(fit.testmNEUPUB, sort. = TRUE)
```

7.1.3 Sensitivity analysis SEM in submission gender norms

construct, only exclude social media

```
testmNEUPUB <- '
Competencies =~ cr_talk + cr_copingresilience +  cr_voice + dCRET20_opfriend_y + dCRET2
0_opelder_y
GenderNorms =~ cr_media  + dCRET20_rolem_y + dCRET20_clubm_y #+ cr_socialmedia
GenderAttitudes =~ cr_gendernormscale_f1 + cr_gendernormscale_f2 + cr_gendernormscale_f
3 + cr_gendernormscale_f4

#mediations/regressions
GenderAttitudes ~ aGN_GA * GenderNorms + aSex_GA * dCRET20_female
cr_genderedactivities ~ aGN_GR * GenderNorms
cr_ghq12~ bGA_MH * GenderAttitudes + bGR_MH * cr_genderedactivities + cSexMH * dCRET20_
female + cGNMH * GenderNorms + Competencies
Competencies ~ bGR_COM * cr_genderedactivities + cGNCOM * GenderNorms#new

#covariances to improve model fit based on modindices
#cr_copingresilience~~ cr_genderedactivities
GenderNorms ~~  GenderAttitudes
dCRET20_opfriend_y ~~ dCRET20_opelder_y
cr_genderedactivities ~~ dCRET20_female
GenderAttitudes ~~ cr_ghq12
dCRET20_rolem_y ~~ cr_genderedactivities

#partial indirect effects
partGNGAMH := aGN_GA * bGA_MH
partGNGRMH := aGN_GR * bGR_MH
partGNGRCOM  := aGN_GR * bGR_COM
partSexGAMH := aSex_GA * bGA_MH

#total indirect effects
tieGNMH := partGNGAMH + partGNGRMH
tieGNCOM := partGNGRCOM
tieSexMH:= partSexGAMH

#total effect
teSexMH := tieSexMH + cSexMH
teGNMH := tieGNMH + cGNMH
teGNCOM := tieGNCOM + cGNCOM#new

med_propSexMH := partSexGAMH / teSexMH
med_propGNGAMH := partGNGAMH / teGNMH
med_propGNGRMH := partGNGRMH / teGNMH
med_propGNGRCOM := partGNGRCOM / teGNCOM

#covariates to the two exogeneous variables (or to the ones the covariates apply)
dCRET20_female~cr_cs_crage + dCRET20_urban + dCRET20_married_y + dCRET20_purpsamp_y + d
CRET20_disability_y + af_asset_10 + dCRET20_fatherinhh +  dCRET20_aspiratt_sec +
dCRET20_aspiratt_teach + dCRET20_aspiratt_uni +  dCRET20_languagehh_201 + dCRET20_langu
agehh_202 + dCRET20_languagehh_211  + dCRET20_workfambus_y

GenderNorms~cr_cs_crage + dCRET20_urban + dCRET20_married_y +dCRET20_purpsamp_y + dCRET
20_disability_y + af_asset_10 + dCRET20_fatherinhh +  dCRET20_aspiratt_sec +
dCRET20_aspiratt_teach + dCRET20_aspiratt_uni +  dCRET20_languagehh_201 + dCRET20_langu
agehh_202 + dCRET20_languagehh_211  + dCRET20_workfambus_y
```

```
'

fit.testmNEUPUB <- sem(testmNEUPUB, data=subsetSEMNeu, estimator="MLR", sampling.weight
s = "rand_weight")
summary(fit.testmNEUPUB, fit.measures=TRUE, standardized=TRUE, rsq=TRUE, ci=TRUE)
```

```
## lavaan 0.6-21 ended normally after 121 iterations
##
##   Estimator                                         ML
##   Optimization method                           NLMINB
##   Number of model parameters                        70
##
##                                                   Used       Total
##   Number of observations                          1588        4777
##   Sampling weights variable                 rand_weight
##
## Model Test User Model:
##                                               Standard      Scaled
##   Test Statistic                              1528.250    1288.471
##   Degrees of freedom                               260         260
##   P-value (Chi-square)                           0.000       0.000
##   Scaling correction factor                                  1.186
##     Yuan-Bentler correction (Mplus variant)
##
## Model Test Baseline Model:
##
##   Test statistic                              5798.021    4873.839
##   Degrees of freedom                               315         315
##   P-value                                        0.000       0.000
##   Scaling correction factor                                  1.190
##
## User Model versus Baseline Model:
##
##   Comparative Fit Index (CFI)                    0.769       0.774
##   Tucker-Lewis Index (TLI)                       0.720       0.727
##
##   Robust Comparative Fit Index (CFI)                         0.775
##   Robust Tucker-Lewis Index (TLI)                            0.727
##
## Loglikelihood and Information Criteria:
##
##   Loglikelihood user model (H0)             -38788.998  -38788.998
##   Scaling correction factor                                  1.169
##       for the MLR correction
##   Loglikelihood unrestricted model (H1)     -38024.873  -38024.873
##   Scaling correction factor                                  1.182
##       for the MLR correction
##
##   Akaike (AIC)                               77717.996   77717.996
##   Bayesian (BIC)                             78093.912   78093.912
##   Sample-size adjusted Bayesian (SABIC)      77871.537   77871.537
##
## Root Mean Square Error of Approximation:
##
##   RMSEA                                          0.055       0.050
##   90 Percent confidence interval - lower         0.053       0.047
##   90 Percent confidence interval - upper         0.058       0.052
##   P-value H_0: RMSEA <= 0.050                    0.000       0.519
##   P-value H_0: RMSEA >= 0.080                    0.000       0.000
##
##   Robust RMSEA                                               0.054
```

```
##   90 Percent confidence interval - lower                      0.051
##   90 Percent confidence interval - upper                      0.057
##   P-value H_0: Robust RMSEA <= 0.050                          0.008
##   P-value H_0: Robust RMSEA >= 0.080                          0.000
##
## Standardized Root Mean Square Residual:
##
##   SRMR                                         0.042       0.042
##
## Parameter Estimates:
##
##   Standard errors                           Sandwich
##   Information bread                         Observed
##   Observed information based on              Hessian
##
## Latent Variables:
##                     Estimate  Std.Err  z-value  P(>|z|) ci.lower ci.upper
##   Competencies =~
##     cr_talk            1.000                               1.000    1.000
##     cr_copingrslnc     3.207    0.270   11.858    0.000    2.677    3.737
##     cr_voice           2.534    0.178   14.244    0.000    2.185    2.883
##     dCRET20_pfrnd_     0.169    0.020    8.491    0.000    0.130    0.208
##     dCRET20_pldr_y     0.186    0.020    9.383    0.000    0.147    0.225
##   GenderNorms =~
##     cr_media           1.000                               1.000    1.000
##     dCRET20_rolm_y     0.250    0.019   13.329    0.000    0.213    0.286
##     dCRET20_clbm_y     0.238    0.017   13.773    0.000    0.204    0.272
##   GenderAttitudes =~
##     cr_gndrnrmsc_1     1.000                               1.000    1.000
##     cr_gndrnrmsc_2     0.784    0.055   14.149    0.000    0.675    0.892
##     cr_gndrnrmsc_3     0.362    0.032   11.320    0.000    0.299    0.425
##     cr_gndrnrmsc_4     0.757    0.059   12.809    0.000    0.641    0.872
##    Std.lv  Std.all
##
##     0.773    0.665
##     2.478    0.637
##     1.958    0.394
##     0.130    0.318
##     0.144    0.290
##
##     0.921    0.587
##     0.230    0.461
##     0.219    0.440
##
##     1.454    0.655
##     1.140    0.544
##     0.526    0.362
##     1.100    0.575
##
## Regressions:
##                          Estimate  Std.Err  z-value  P(>|z|) ci.lower ci.upper
##   GenderAttitudes ~
##     GndrN (aGN_GA)          1.358    0.113   11.974    0.000    1.136    1.580
##     dCRET   (aS_G)          0.504    0.087    5.786    0.000    0.333    0.675
##   cr_genderedactivities ~
##     GndrN (aGN_GR)         -1.395    0.090  -15.478    0.000   -1.572   -1.219
```

```
##   cr_ghq12 ~
##     GndrA   (bGA_)          -0.483    0.492   -0.982    0.326   -1.447    0.481
##     cr_gn  (bGR_M)          -0.016    0.087   -0.184    0.854   -0.186    0.154
##     dCRET   (cSMH)          -0.321    0.331   -0.970    0.332   -0.969    0.327
##     GndrN   (cGNM)           0.589    0.848    0.695    0.487   -1.073    2.251
##     Cmptn                   -1.144    0.452   -2.534    0.011   -2.029   -0.259
##   Competencies ~
##     cr_gn  (bGR_C)           0.061    0.023    2.626    0.009    0.015    0.106
##     GndrN   (cGNC)           0.703    0.076    9.193    0.000    0.553    0.853
##   dCRET20_female ~
##     cr_c_                   -0.028    0.014   -2.042    0.041   -0.056   -0.001
##     dCRET                   -0.038    0.027   -1.391    0.164   -0.092    0.016
##     dCRET                   -0.281    0.058   -4.804    0.000   -0.395   -0.166
##     dCRET                    0.075    0.068    1.090    0.276   -0.060    0.209
##     dCRET                    0.071    0.056    1.273    0.203   -0.038    0.181
##     a__10                   -0.002    0.005   -0.443    0.658   -0.012    0.008
##     dCRET                   -0.042    0.030   -1.405    0.160   -0.100    0.016
##     dCRET                   -0.032    0.059   -0.541    0.589   -0.148    0.084
##     dCRET                   -0.203    0.080   -2.541    0.011   -0.360   -0.046
##     dCRET                   -0.278    0.057   -4.895    0.000   -0.389   -0.166
##     dCRET                   -0.109    0.030   -3.677    0.000   -0.167   -0.051
##     dCRET                   -0.082    0.041   -1.988    0.047   -0.162   -0.001
##     dCRET                   -0.005    0.101   -0.053    0.958   -0.202    0.192
##     dCRET                   -0.100    0.056   -1.762    0.078   -0.210    0.011
##   GenderNorms ~
##     cr_c_                    0.065    0.023    2.815    0.005    0.020    0.110
##     dCRET                   -0.230    0.052   -4.394    0.000   -0.332   -0.127
##     dCRET                    0.691    0.138    4.992    0.000    0.420    0.962
##     dCRET                    0.047    0.105    0.443    0.658   -0.160    0.253
##     dCRET                    0.238    0.082    2.895    0.004    0.077    0.400
##     a__10                    0.111    0.012    9.230    0.000    0.087    0.134
##     dCRET                   -0.056    0.046   -1.213    0.225   -0.146    0.034
##     dCRET                    0.231    0.109    2.118    0.034    0.017    0.445
##     dCRET                    0.430    0.127    3.396    0.001    0.182    0.678
##     dCRET                    0.922    0.107    8.594    0.000    0.712    1.132
##     dCRET                   -0.561    0.050  -11.287    0.000   -0.658   -0.463
##     dCRET                   -0.510    0.063   -8.148    0.000   -0.633   -0.387
##     dCRET                   -0.159    0.159   -1.003    0.316   -0.470    0.152
##     dCRET                   -0.185    0.078   -2.360    0.018   -0.338   -0.031
##    Std.lv  Std.all
##
##     0.860    0.860
##     0.347    0.173
##
##    -1.286   -0.621
##
##    -0.702   -0.188
##    -0.016   -0.009
##    -0.321   -0.043
##     0.543    0.145
##    -0.884   -0.237
##
##     0.079    0.163
##     0.838    0.838
##
##    -0.028   -0.053
```

```
##    -0.038   -0.038
##    -0.281   -0.096
##     0.075    0.029
##     0.071    0.034
##    -0.002   -0.012
##    -0.042   -0.035
##    -0.032   -0.027
##    -0.203   -0.090
##    -0.278   -0.264
##    -0.109   -0.101
##    -0.082   -0.059
##    -0.005   -0.001
##    -0.100   -0.044
##
##     0.071    0.065
##    -0.249   -0.124
##     0.750    0.127
##     0.051    0.010
##     0.259    0.062
##     0.120    0.329
##    -0.060   -0.026
##     0.251    0.105
##     0.466    0.103
##     1.000    0.475
##    -0.608   -0.281
##    -0.553   -0.200
##    -0.173   -0.020
##    -0.200   -0.044
##
## Covariances:
##                            Estimate  Std.Err  z-value  P(>|z|) ci.lower
##   .GenderNorms ~~
##     .GenderAttituds           -0.244    0.057   -4.281    0.000   -0.356
##   .dCRET20_opfriend_y ~~
##     .dCRET20_pldr_y            0.041    0.005    8.533    0.000    0.032
##   .cr_genderedactivities ~~
##     .dCRET20_female            0.291    0.024   11.907    0.000    0.243
##   .GenderAttitudes ~~
##     .cr_ghq12                  0.475    0.488    0.973    0.330   -0.481
##   .dCRET20_rolem_y ~~
##     .cr_gendrdctvts            0.113    0.023    4.858    0.000    0.067
##   ci.upper   Std.lv  Std.all
##
##    -0.132   -0.389   -0.389
##
##     0.051    0.041    0.225
##
##     0.339    0.291    0.375
##
##     1.431    0.433    0.118
##
##     0.158    0.113    0.157
##
## Variances:
##                    Estimate  Std.Err  z-value  P(>|z|) ci.lower ci.upper
##    .cr_talk           0.754    0.055   13.792    0.000    0.647    0.861
```

```
##    .cr_copingrslnc    8.997    0.619   14.538    0.000    7.784   10.210
##    .cr_voice         20.872    0.845   24.699    0.000   19.216   22.528
##    .dCRET20_pfrnd_    0.151    0.006   24.597    0.000    0.139    0.163
##    .dCRET20_pldr_y    0.225    0.004   53.699    0.000    0.217    0.234
##    .cr_media          1.616    0.076   21.304    0.000    1.467    1.764
##    .dCRET20_rolm_y    0.196    0.006   34.129    0.000    0.185    0.207
##    .dCRET20_clbm_y    0.199    0.005   37.294    0.000    0.189    0.210
##    .cr_gndrnrmsc_1    2.815    0.161   17.508    0.000    2.500    3.131
##    .cr_gndrnrmsc_2    3.094    0.145   21.295    0.000    2.809    3.378
##    .cr_gndrnrmsc_3    1.834    0.070   26.123    0.000    1.696    1.971
##    .cr_gndrnrmsc_4    2.454    0.126   19.539    0.000    2.208    2.700
##    .cr_gendrdctvts    2.636    0.178   14.791    0.000    2.287    2.985
##    .cr_ghq12         13.400    0.989   13.549    0.000   11.462   15.339
##    .dCRET20_female    0.229    0.004   64.046    0.000    0.222    0.236
##    .Competencies      0.263    0.041    6.405    0.000    0.182    0.343
##    .GenderNorms       0.327    0.033    9.855    0.000    0.262    0.392
##    .GenderAttituds    1.204    0.149    8.101    0.000    0.913    1.496
##    Std.lv  Std.all
##     0.754    0.558
##     8.997    0.594
##    20.872    0.845
##     0.151    0.899
##     0.225    0.916
##     1.616    0.656
##     0.196    0.788
##     0.199    0.806
##     2.815    0.571
##     3.094    0.704
##     1.834    0.869
##     2.454    0.670
##     2.636    0.615
##    13.400    0.960
##     0.229    0.919
##     0.440    0.440
##     0.385    0.385
##     0.569    0.569
##
## R-Square:
##                    Estimate
##     cr_talk           0.442
##     cr_copingrslnc    0.406
##     cr_voice          0.155
##     dCRET20_pfrnd_    0.101
##     dCRET20_pldr_y    0.084
##     cr_media          0.344
##     dCRET20_rolm_y    0.212
##     dCRET20_clbm_y    0.194
##     cr_gndrnrmsc_1    0.429
##     cr_gndrnrmsc_2    0.296
##     cr_gndrnrmsc_3    0.131
##     cr_gndrnrmsc_4    0.330
##     cr_gendrdctvts    0.385
##     cr_ghq12          0.040
##     dCRET20_female    0.081
##     Competencies      0.560
##     GenderNorms       0.615
```

```
##     GenderAttituds    0.431
##
## Defined Parameters:
##                      Estimate  Std.Err  z-value  P(>|z|) ci.lower ci.upper
##     partGNGAMH         -0.656    0.664   -0.987    0.324   -1.958    0.647
##     partGNGRMH          0.022    0.121    0.184    0.854   -0.215    0.260
##     partGNGRCOM        -0.085    0.034   -2.525    0.012   -0.150   -0.019
##     partSexGAMH        -0.243    0.251   -0.969    0.332   -0.735    0.249
##     tieGNMH            -0.633    0.727   -0.871    0.384   -2.058    0.792
##     tieGNCOM           -0.085    0.034   -2.525    0.012   -0.150   -0.019
##     tieSexMH           -0.243    0.251   -0.969    0.332   -0.735    0.249
##     teSexMH            -0.564    0.222   -2.546    0.011   -0.999   -0.130
##     teGNMH             -0.044    0.288   -0.154    0.878   -0.609    0.521
##     teGNCOM             0.618    0.056   11.028    0.000    0.508    0.728
##     med_propSexMH       0.431    0.472    0.913    0.361   -0.494    1.357
##     med_propGNGAMH     14.805  100.387    0.147    0.883 -181.950  211.560
##     med_propGNGRMH     -0.505    3.155   -0.160    0.873   -6.688    5.678
##     med_prpGNGRCOM     -0.137    0.050   -2.723    0.006   -0.236   -0.038
##    Std.lv  Std.all
##    -0.604   -0.162
##     0.021    0.006
##    -0.101   -0.101
##    -0.243   -0.032
##    -0.584   -0.156
##    -0.101   -0.101
##    -0.243   -0.032
##    -0.564   -0.075
##    -0.041   -0.011
##     0.737    0.737
##     0.431    0.431
##    14.805   14.805
##    -0.505   -0.505
##    -0.137   -0.137
```

```
#fitMeasures(fit.testmNEUPUB)

#parameterEstimates(fit.testmNEUPUB, standardized = TRUE)
#modindices(fit.testmNEUPUB, sort. = TRUE)
```

7.2 Final SEM after peer review

```
#changes: CFI > 0.4
#new modindices

testmPUBPeer <- '
Competencies =~ cr_talk + cr_copingresilience
GenderNorms =~ cr_media  + dCRET20_rolem_y + dCRET20_clubm_y
GenderAttitudes =~ cr_gendernormscale_f1 + cr_gendernormscale_f2 +  cr_gendernormscale_
f4

#mediations/regressions
GenderAttitudes ~ aGN_GA * GenderNorms + aSex_GA * dCRET20_female
cr_genderedactivities ~ aGN_GR * GenderNorms
cr_ghq12~ bGA_MH * GenderAttitudes + bGR_MH * cr_genderedactivities + cSexMH * dCRET20_
female + cGNMH * GenderNorms + Competencies
Competencies ~ bGR_COM * cr_genderedactivities + cGNCOM * GenderNorms#new

#Modindizes: covariances to improve model fit
cr_genderedactivities ~~ dCRET20_female
cr_gendernormscale_f4 ~~ cr_ghq12
cr_gendernormscale_f4 ~~ dCRET20_female
cr_media ~~ cr_genderedactivities
cr_copingresilience ~~ cr_gendernormscale_f2

#regressions MIs: latent variable + covariate is ok!
GenderAttitudes ~ dCRET20_languagehh_201
Competencies ~ dCRET20_urban
#Competencies ~ af_asset_10 makes gender norms ->mh negative
GenderAttitudes ~ dCRET20_languagehh_202

#partial indirect effects
partGNGAMH := aGN_GA * bGA_MH
partGNGRMH := aGN_GR * bGR_MH
partGNGRCOM  := aGN_GR * bGR_COM
partSexGAMH := aSex_GA * bGA_MH

#total indirect effects
tieGNMH := partGNGAMH + partGNGRMH
tieGNCOM := partGNGRCOM
tieSexMH:= partSexGAMH

#total effect
teSexMH := tieSexMH + cSexMH
teGNMH := tieGNMH + cGNMH
teGNCOM := tieGNCOM + cGNCOM#new

med_propSexMH := partSexGAMH / teSexMH
med_propGNGAMH := partGNGAMH / teGNMH
med_propGNGRMH := partGNGRMH / teGNMH
med_propGNGRCOM := partGNGRCOM / teGNCOM

#covariates to the two exogeneous variables (or to the ones the covariates apply)
dCRET20_female~cr_cs_crage + dCRET20_urban + dCRET20_married_y + dCRET20_purpsamp_y + d
CRET20_disability_y + af_asset_10 + dCRET20_fatherinhh +  dCRET20_aspiratt_sec +
```

```
dCRET20_aspiratt_teach + dCRET20_aspiratt_uni +  dCRET20_languagehh_201 + dCRET20_langu
agehh_202 + dCRET20_languagehh_211  + dCRET20_workfambus_y

GenderNorms~cr_cs_crage + dCRET20_urban + dCRET20_married_y +dCRET20_purpsamp_y + dCRET
20_disability_y + af_asset_10 + dCRET20_fatherinhh +  dCRET20_aspiratt_sec +
dCRET20_aspiratt_teach + dCRET20_aspiratt_uni +  dCRET20_languagehh_201 + dCRET20_langu
agehh_202 + dCRET20_languagehh_211  + dCRET20_workfambus_y
'

fit.testmPUBPeer <- sem(testmPUBPeer, data=subsetSEMNeu, estimator="MLR", sampling.weig
hts = "rand_weight")
summary(fit.testmPUBPeer, fit.measures=TRUE, standardized=TRUE, rsq=TRUE, ci=TRUE)
```

```
## lavaan 0.6-21 ended normally after 114 iterations
##
##   Estimator                                         ML
##   Optimization method                           NLMINB
##   Number of model parameters                        65
##
##                                                   Used       Total
##   Number of observations                          1651        4777
##   Sampling weights variable                rand_weight
##
## Model Test User Model:
##                                               Standard      Scaled
##   Test Statistic                              1041.886     876.651
##   Degrees of freedom                               155         155
##   P-value (Chi-square)                           0.000       0.000
##   Scaling correction factor                                  1.188
##     Yuan-Bentler correction (Mplus variant)
##
## Model Test Baseline Model:
##
##   Test statistic                              5004.766    4197.235
##   Degrees of freedom                               209         209
##   P-value                                        0.000       0.000
##   Scaling correction factor                                  1.192
##
## User Model versus Baseline Model:
##
##   Comparative Fit Index (CFI)                    0.815       0.819
##   Tucker-Lewis Index (TLI)                       0.751       0.756
##
##   Robust Comparative Fit Index (CFI)                         0.820
##   Robust Tucker-Lewis Index (TLI)                            0.757
##
## Loglikelihood and Information Criteria:
##
##   Loglikelihood user model (H0)             -30609.695  -30609.695
##   Scaling correction factor                                  1.192
##       for the MLR correction
##   Loglikelihood unrestricted model (H1)     -30088.752  -30088.752
##   Scaling correction factor                                  1.189
##       for the MLR correction
##
##   Akaike (AIC)                               61349.390   61349.390
##   Bayesian (BIC)                             61700.984   61700.984
##   Sample-size adjusted Bayesian (SABIC)      61494.489   61494.489
##
## Root Mean Square Error of Approximation:
##
##   RMSEA                                          0.059       0.053
##   90 Percent confidence interval - lower         0.056       0.050
##   90 Percent confidence interval - upper         0.062       0.056
##   P-value H_0: RMSEA <= 0.050                    0.000       0.051
##   P-value H_0: RMSEA >= 0.080                    0.000       0.000
##
##   Robust RMSEA                                               0.058
```

```
##   90 Percent confidence interval - lower                     0.054
##   90 Percent confidence interval - upper                     0.062
##   P-value H_0: Robust RMSEA <= 0.050                         0.000
##   P-value H_0: Robust RMSEA >= 0.080                         0.000
##
## Standardized Root Mean Square Residual:
##
##   SRMR                                         0.038       0.038
##
## Parameter Estimates:
##
##   Standard errors                            Sandwich
##   Information bread                          Observed
##   Observed information based on               Hessian
##
## Latent Variables:
##                      Estimate  Std.Err  z-value  P(>|z|) ci.lower ci.upper
##   Competencies =~
##     cr_talk             1.000                               1.000    1.000
##     cr_copingrslnc      3.599    0.282   12.762    0.000    3.046    4.152
##   GenderNorms =~
##     cr_media            1.000                               1.000    1.000
##     dCRET20_rolm_y      0.222    0.016   13.585    0.000    0.190    0.254
##     dCRET20_clbm_y      0.236    0.017   13.774    0.000    0.203    0.270
##   GenderAttitudes =~
##     cr_gndrnrmsc_1      1.000                               1.000    1.000
##     cr_gndrnrmsc_2      0.884    0.060   14.684    0.000    0.766    1.001
##     cr_gndrnrmsc_4      0.871    0.061   14.342    0.000    0.752    0.990
##    Std.lv  Std.all
##
##     0.687    0.586
##     2.474    0.637
##
##     0.917    0.586
##     0.203    0.408
##     0.217    0.436
##
##     1.337    0.602
##     1.181    0.565
##     1.165    0.609
##
## Regressions:
##                           Estimate  Std.Err  z-value  P(>|z|) ci.lower ci.upper
##   GenderAttitudes ~
##     GndrN (aGN_GA)           0.895    0.090    9.939    0.000    0.719    1.072
##     dCRET   (aS_G)           0.543    0.091    5.982    0.000    0.365    0.721
##   cr_genderedactivities ~
##     GndrN (aGN_GR)          -1.368    0.084  -16.331    0.000   -1.532   -1.204
##   cr_ghq12 ~
##     GndrA   (bGA_)          -0.110    0.177   -0.623    0.533   -0.456    0.236
##     cr_gn  (bGR_M)          -0.079    0.067   -1.187    0.235   -0.209    0.051
##     dCRET   (cSMH)          -0.492    0.240   -2.053    0.040   -0.962   -0.022
##     GndrN   (cGNM)           0.303    0.343    0.884    0.377   -0.370    0.976
##     Cmptn                   -1.706    0.418   -4.080    0.000   -2.525   -0.886
##   Competencies ~
##     cr_gn  (bGR_C)           0.016    0.018    0.865    0.387   -0.020    0.052
```

```
##     GndrN   (cGNC)          0.683    0.075    9.120    0.000    0.536    0.829
##   GenderAttitudes ~
##     dCRET                  -0.779    0.112   -6.953    0.000   -0.998   -0.559
##   Competencies ~
##     dCRET                   0.311    0.059    5.256    0.000    0.195    0.427
##   GenderAttitudes ~
##     dCRET                  -0.636    0.149   -4.275    0.000   -0.927   -0.344
##   dCRET20_female ~
##     cr_c_                  -0.028    0.014   -2.066    0.039   -0.055   -0.001
##     dCRET                  -0.026    0.027   -0.960    0.337   -0.080    0.027
##     dCRET                  -0.274    0.057   -4.787    0.000   -0.386   -0.162
##     dCRET                   0.046    0.065    0.705    0.481   -0.081    0.172
##     dCRET                   0.080    0.055    1.464    0.143   -0.027    0.188
##     a__10                  -0.003    0.005   -0.678    0.498   -0.013    0.006
##     dCRET                  -0.041    0.029   -1.431    0.153   -0.098    0.015
##     dCRET                  -0.034    0.057   -0.592    0.554   -0.146    0.078
##     dCRET                  -0.181    0.078   -2.318    0.020   -0.335   -0.028
##     dCRET                  -0.280    0.055   -5.087    0.000   -0.388   -0.172
##     dCRET                  -0.129    0.029   -4.407    0.000   -0.186   -0.072
##     dCRET                  -0.108    0.039   -2.745    0.006   -0.184   -0.031
##     dCRET                   0.004    0.103    0.042    0.967   -0.198    0.206
##     dCRET                  -0.072    0.055   -1.309    0.190   -0.180    0.036
##   GenderNorms ~
##     cr_c_                   0.058    0.024    2.407    0.016    0.011    0.106
##     dCRET                  -0.391    0.057   -6.867    0.000   -0.503   -0.279
##     dCRET                   0.806    0.132    6.117    0.000    0.548    1.064
##     dCRET                   0.060    0.107    0.561    0.575   -0.149    0.269
##     dCRET                   0.261    0.084    3.121    0.002    0.097    0.425
##     a__10                   0.122    0.013    9.339    0.000    0.097    0.148
##     dCRET                  -0.026    0.050   -0.524    0.600   -0.123    0.071
##     dCRET                   0.309    0.112    2.750    0.006    0.089    0.530
##     dCRET                   0.481    0.131    3.672    0.000    0.224    0.737
##     dCRET                   1.019    0.104    9.757    0.000    0.814    1.223
##     dCRET                  -0.412    0.054   -7.652    0.000   -0.518   -0.307
##     dCRET                  -0.405    0.069   -5.883    0.000   -0.540   -0.270
##     dCRET                  -0.152    0.163   -0.929    0.353   -0.472    0.168
##     dCRET                  -0.236    0.080   -2.958    0.003   -0.393   -0.080
##    Std.lv  Std.all
##
##     0.614    0.614
##     0.406    0.202
##
##    -1.254   -0.590
##
##    -0.147   -0.040
##    -0.079   -0.045
##    -0.492   -0.066
##     0.278    0.075
##    -1.173   -0.316
##
##     0.023    0.049
##     0.911    0.911
##
##    -0.582   -0.270
##
##     0.452    0.225
```

```
##
##    -0.476   -0.172
##
##    -0.028   -0.052
##    -0.026   -0.026
##    -0.274   -0.108
##     0.046    0.019
##     0.080    0.039
##    -0.003   -0.018
##    -0.041   -0.035
##    -0.034   -0.029
##    -0.181   -0.080
##    -0.280   -0.269
##    -0.129   -0.120
##    -0.108   -0.078
##     0.004    0.001
##    -0.072   -0.032
##
##     0.063    0.059
##    -0.426   -0.212
##     0.879    0.172
##     0.065    0.014
##     0.285    0.068
##     0.133    0.364
##    -0.028   -0.012
##     0.337    0.143
##     0.524    0.115
##     1.111    0.531
##    -0.449   -0.208
##    -0.442   -0.160
##    -0.165   -0.019
##    -0.258   -0.057
##
## Covariances:
##                              Estimate  Std.Err  z-value  P(>|z|) ci.lower
##   .cr_genderedactivities ~~
##     .dCRET20_female             0.288    0.024   12.045    0.000    0.242
##   .cr_gendernormscale_f4 ~~
##     .cr_ghq12                   0.862    0.200    4.305    0.000    0.470
##     .dCRET20_female            -0.070    0.025   -2.824    0.005   -0.119
##   .cr_media ~~
##     .cr_gendrdctvts            -0.148    0.068   -2.182    0.029   -0.281
##   .cr_copingresilience ~~
##     .cr_gndrnrmsc_2            -0.614    0.170   -3.609    0.000   -0.947
##   ci.upper   Std.lv  Std.all
##
##     0.335    0.288    0.353
##
##     1.255    0.862    0.157
##    -0.021   -0.070   -0.096
##
##    -0.015   -0.148   -0.068
##
##    -0.280   -0.614   -0.119
##
## Variances:
```

```
##                       Estimate  Std.Err  z-value  P(>|z|) ci.lower ci.upper
##    .cr_talk              0.902    0.049   18.508    0.000    0.806    0.997
##    .cr_copingrslnc       8.943    0.599   14.941    0.000    7.770   10.116
##    .cr_media             1.607    0.074   21.771    0.000    1.462    1.751
##    .dCRET20_rolm_y       0.208    0.005   43.270    0.000    0.198    0.217
##    .dCRET20_clbm_y       0.200    0.005   38.300    0.000    0.190    0.210
##    .cr_gndrnrmsc_1       3.146    0.154   20.382    0.000    2.844    3.449
##    .cr_gndrnrmsc_2       2.975    0.142   21.020    0.000    2.698    3.253
##    .cr_gndrnrmsc_4       2.365    0.124   19.046    0.000    2.121    2.608
##    .cr_gendrdctvts       2.951    0.174   16.965    0.000    2.610    3.291
##    .cr_ghq12            12.720    0.877   14.501    0.000   11.001   14.440
##    .dCRET20_female       0.226    0.004   64.205    0.000    0.219    0.233
##    .Competencies         0.164    0.034    4.784    0.000    0.097    0.232
##    .GenderNorms          0.244    0.028    8.590    0.000    0.188    0.300
##    .GenderAttituds       0.670    0.092    7.264    0.000    0.489    0.851
##    Std.lv  Std.all
##     0.902    0.656
##     8.943    0.594
##     1.607    0.656
##     0.208    0.834
##     0.200    0.810
##     3.146    0.638
##     2.975    0.681
##     2.365    0.647
##     2.951    0.652
##    12.720    0.923
##     0.226    0.913
##     0.348    0.348
##     0.290    0.290
##     0.375    0.375
##
## R-Square:
##                       Estimate
##     cr_talk              0.344
##     cr_copingrslnc       0.406
##     cr_media             0.344
##     dCRET20_rolm_y       0.166
##     dCRET20_clbm_y       0.190
##     cr_gndrnrmsc_1       0.362
##     cr_gndrnrmsc_2       0.319
##     cr_gndrnrmsc_4       0.353
##     cr_gendrdctvts       0.348
##     cr_ghq12             0.077
##     dCRET20_female       0.087
##     Competencies         0.652
##     GenderNorms          0.710
##     GenderAttituds       0.625
##
## Defined Parameters:
##                       Estimate  Std.Err  z-value  P(>|z|) ci.lower ci.upper
##     partGNGAMH          -0.098    0.159   -0.619    0.536   -0.410    0.213
##     partGNGRMH           0.108    0.091    1.182    0.237   -0.071    0.287
##     partGNGRCOM         -0.022    0.025   -0.859    0.390   -0.071    0.028
##     partSexGAMH         -0.060    0.097   -0.619    0.536   -0.249    0.130
##     tieGNMH              0.010    0.185    0.051    0.959   -0.354    0.373
##     tieGNCOM            -0.022    0.025   -0.859    0.390   -0.071    0.028
```

```
##     tieSexMH         -0.060    0.097   -0.619    0.536   -0.249    0.130
##     teSexMH          -0.552    0.217   -2.547    0.011   -0.976   -0.127
##     teGNMH            0.313    0.263    1.191    0.234   -0.202    0.828
##     teGNCOM           0.661    0.063   10.488    0.000    0.537    0.784
##     med_propSexMH     0.108    0.181    0.598    0.550   -0.247    0.464
##     med_propGNGAMH   -0.315    0.561   -0.561    0.575   -1.414    0.785
##     med_propGNGRMH    0.345    0.454    0.761    0.447   -0.544    1.234
##     med_prpGNGRCOM   -0.033    0.037   -0.879    0.379   -0.106    0.040
##    Std.lv  Std.all
##    -0.090   -0.024
##     0.099    0.027
##    -0.029   -0.029
##    -0.060   -0.008
##     0.009    0.002
##    -0.029   -0.029
##    -0.060   -0.008
##    -0.552   -0.074
##     0.287    0.077
##     0.882    0.882
##     0.108    0.108
##    -0.315   -0.315
##     0.345    0.345
##    -0.033   -0.033
```

```
#fitMeasures(fit.testmPUBPeer)

#parameterEstimates(fit.testmPUBPeer, standardized = TRUE)
#modindices(fit.testmPUBPeer, sort. = TRUE)
```

7.2.1 Sensitivity analysis final SEM after peer review gender norms construct

##both variables club member and role model removed

```
testmPUBPeerSen <- '
Competencies =~ cr_talk + cr_copingresilience
GenderNorms =~ cr_media
GenderAttitudes =~ cr_gendernormscale_f1 + cr_gendernormscale_f2 +  cr_gendernormscale_
f4 #+ cr_gendernormscale_f5 + cr_gendernormscale_f6 + cr_gendernormscale_f7 cr_genderno
rmscale_f3 +

#mediations/regressions
GenderAttitudes ~ aGN_GA * GenderNorms + aSex_GA * dCRET20_female
cr_genderedactivities ~ aGN_GR * GenderNorms
cr_ghq12~ bGA_MH * GenderAttitudes + bGR_MH * cr_genderedactivities + cSexMH * dCRET20_
female + cGNMH * GenderNorms + Competencies
Competencies ~ bGR_COM * cr_genderedactivities + cGNCOM * GenderNorms#new

#Modindizes: covariances to improve model fit
cr_genderedactivities ~~ dCRET20_female
cr_gendernormscale_f4 ~~ cr_ghq12
cr_gendernormscale_f4 ~~ dCRET20_female
cr_media ~~ cr_genderedactivities
cr_copingresilience ~~ cr_gendernormscale_f2

#regressions MIs: latent variable + covariate is ok!
GenderAttitudes ~ dCRET20_languagehh_201
Competencies ~ dCRET20_urban
#Competencies ~ af_asset_10 makes gender norms ->mh negative
GenderAttitudes ~ dCRET20_languagehh_202

#partial indirect effects
partGNGAMH := aGN_GA * bGA_MH
partGNGRMH := aGN_GR * bGR_MH
partGNGRCOM  := aGN_GR * bGR_COM
partSexGAMH := aSex_GA * bGA_MH

#total indirect effects
tieGNMH := partGNGAMH + partGNGRMH
tieGNCOM := partGNGRCOM
tieSexMH:= partSexGAMH

#total effect
teSexMH := tieSexMH + cSexMH
teGNMH := tieGNMH + cGNMH
teGNCOM := tieGNCOM + cGNCOM#new

med_propSexMH := partSexGAMH / teSexMH
med_propGNGAMH := partGNGAMH / teGNMH
med_propGNGRMH := partGNGRMH / teGNMH
med_propGNGRCOM := partGNGRCOM / teGNCOM

#covariates to the two exogeneous variables (or to the ones the covariates apply)
dCRET20_female~cr_cs_crage + dCRET20_urban + dCRET20_married_y + dCRET20_purpsamp_y + d
CRET20_disability_y + af_asset_10 + dCRET20_fatherinhh +  dCRET20_aspiratt_sec +
dCRET20_aspiratt_teach + dCRET20_aspiratt_uni +  dCRET20_languagehh_201 + dCRET20_langu
agehh_202 + dCRET20_languagehh_211  + dCRET20_workfambus_y

GenderNorms~cr_cs_crage + dCRET20_urban + dCRET20_married_y +dCRET20_purpsamp_y + dCRET
```

```
20_disability_y + af_asset_10 + dCRET20_fatherinhh +  dCRET20_aspiratt_sec +
dCRET20_aspiratt_teach + dCRET20_aspiratt_uni +  dCRET20_languagehh_201 + dCRET20_langu
agehh_202 + dCRET20_languagehh_211  + dCRET20_workfambus_y
'

fit.testmPUBPeerSen <- sem(testmPUBPeerSen, data=subsetSEMNeu, estimator="MLR", samplin
g.weights = "rand_weight")
```

```
## Warning: lavaan->lav_object_post_check():
##    the covariance matrix of the residuals of the observed variables (theta)
##    is not positive definite ; use lavInspect(fit, "theta") to investigate.
```

```
summary(fit.testmPUBPeerSen, fit.measures=TRUE, standardized=TRUE, rsq=TRUE, ci=TRUE)
```

```
## lavaan 0.6-21 ended normally after 112 iterations
##
##   Estimator                                         ML
##   Optimization method                           NLMINB
##   Number of model parameters                        60
##
##                                                   Used       Total
##   Number of observations                          1654        4777
##   Sampling weights variable                rand_weight
##
## Model Test User Model:
##                                               Standard      Scaled
##   Test Statistic                              1088.743     893.278
##   Degrees of freedom                               111         111
##   P-value (Chi-square)                           0.000       0.000
##   Scaling correction factor                                  1.219
##     Yuan-Bentler correction (Mplus variant)
##
## Model Test Baseline Model:
##
##   Test statistic                              4240.960    3524.832
##   Degrees of freedom                               162         162
##   P-value                                        0.000       0.000
##   Scaling correction factor                                  1.203
##
## User Model versus Baseline Model:
##
##   Comparative Fit Index (CFI)                    0.760       0.767
##   Tucker-Lewis Index (TLI)                       0.650       0.660
##
##   Robust Comparative Fit Index (CFI)                         0.764
##   Robust Tucker-Lewis Index (TLI)                            0.656
##
## Loglikelihood and Information Criteria:
##
##   Loglikelihood user model (H0)             -28685.883  -28685.883
##   Scaling correction factor                                  1.201
##       for the MLR correction
##   Loglikelihood unrestricted model (H1)     -28141.511  -28141.511
##   Scaling correction factor                                  1.213
##       for the MLR correction
##
##   Akaike (AIC)                               57491.765   57491.765
##   Bayesian (BIC)                             57816.422   57816.422
##   Sample-size adjusted Bayesian (SABIC)      57625.812   57625.812
##
## Root Mean Square Error of Approximation:
##
##   RMSEA                                          0.073       0.065
##   90 Percent confidence interval - lower         0.069       0.062
##   90 Percent confidence interval - upper         0.077       0.069
##   P-value H_0: RMSEA <= 0.050                    0.000       0.000
##   P-value H_0: RMSEA >= 0.080                    0.002       0.000
##
##   Robust RMSEA                                               0.072
```

```
##   90 Percent confidence interval - lower                     0.068
##   90 Percent confidence interval - upper                     0.076
##   P-value H_0: Robust RMSEA <= 0.050                         0.000
##   P-value H_0: Robust RMSEA >= 0.080                         0.002
##
## Standardized Root Mean Square Residual:
##
##   SRMR                                          0.056       0.056
##
## Parameter Estimates:
##
##   Standard errors                             Sandwich
##   Information bread                           Observed
##   Observed information based on                Hessian
##
## Latent Variables:
##                      Estimate  Std.Err  z-value  P(>|z|) ci.lower ci.upper
##   Competencies =~
##     cr_talk             1.000                               1.000    1.000
##     cr_copingrslnc      4.530    0.556    8.152    0.000    3.441    5.620
##   GenderNorms =~
##     cr_media            1.000                               1.000    1.000
##   GenderAttitudes =~
##     cr_gndrnrmsc_1      1.000                               1.000    1.000
##     cr_gndrnrmsc_2      1.014    0.069   14.695    0.000    0.879    1.149
##     cr_gndrnrmsc_4      0.917    0.065   14.045    0.000    0.789    1.045
##    Std.lv  Std.all
##
##     0.615    0.524
##     2.788    0.718
##
##     1.563    1.000
##
##     1.262    0.569
##     1.280    0.611
##     1.158    0.606
##
## Regressions:
##                           Estimate  Std.Err  z-value  P(>|z|) ci.lower ci.upper
##   GenderAttitudes ~
##     GndrN (aGN_GA)           0.235    0.027    8.829    0.000    0.183    0.287
##     dCRET   (aS_G)           0.319    0.084    3.778    0.000    0.153    0.484
##   cr_genderedactivities ~
##     GndrN (aGN_GR)          -1.101    0.069  -15.881    0.000   -1.237   -0.965
##   cr_ghq12 ~
##     GndrA   (bGA_)          -0.210    0.118   -1.782    0.075   -0.440    0.021
##     cr_gn  (bGR_M)          -0.147    0.073   -2.017    0.044   -0.290   -0.004
##     dCRET   (cSMH)          -0.429    0.227   -1.894    0.058   -0.873    0.015
##     GndrN   (cGNM)          -0.009    0.080   -0.116    0.907   -0.167    0.148
##     Cmptn                   -1.731    0.309   -5.601    0.000   -2.337   -1.125
##   Competencies ~
##     cr_gn  (bGR_C)          -0.083    0.019   -4.283    0.000   -0.121   -0.045
##     GndrN   (cGNC)           0.054    0.022    2.438    0.015    0.011    0.098
##   GenderAttitudes ~
##     dCRET                   -1.219    0.095  -12.861    0.000   -1.405   -1.034
##   Competencies ~
```

```
##     dCRET                   -0.026    0.048   -0.552    0.581   -0.121    0.068
##   GenderAttitudes ~
##     dCRET                   -1.319    0.135   -9.777    0.000   -1.583   -1.055
##   dCRET20_female ~
##     cr_c_                   -0.026    0.013   -1.923    0.054   -0.052    0.000
##     dCRET                   -0.021    0.028   -0.764    0.445   -0.076    0.033
##     dCRET                   -0.286    0.056   -5.141    0.000   -0.395   -0.177
##     dCRET                    0.040    0.064    0.629    0.530   -0.085    0.166
##     dCRET                    0.104    0.055    1.891    0.059   -0.004    0.211
##     a__10                   -0.008    0.005   -1.575    0.115   -0.017    0.002
##     dCRET                   -0.022    0.029   -0.755    0.450   -0.078    0.035
##     dCRET                   -0.011    0.057   -0.199    0.842   -0.122    0.100
##     dCRET                   -0.161    0.080   -2.008    0.045   -0.318   -0.004
##     dCRET                   -0.241    0.055   -4.363    0.000   -0.349   -0.133
##     dCRET                   -0.155    0.029   -5.305    0.000   -0.212   -0.098
##     dCRET                   -0.162    0.039   -4.164    0.000   -0.238   -0.086
##     dCRET                   -0.061    0.099   -0.612    0.541   -0.255    0.134
##     dCRET                   -0.078    0.058   -1.342    0.180   -0.193    0.036
##   GenderNorms ~
##     cr_c_                    0.046    0.032    1.442    0.149   -0.017    0.109
##     dCRET                   -0.567    0.067   -8.432    0.000   -0.699   -0.435
##     dCRET                    1.164    0.151    7.735    0.000    0.869    1.459
##     dCRET                    0.155    0.140    1.109    0.268   -0.119    0.430
##     dCRET                   -0.001    0.120   -0.008    0.994   -0.237    0.235
##     a__10                    0.188    0.014   13.141    0.000    0.160    0.216
##     dCRET                   -0.242    0.070   -3.464    0.001   -0.379   -0.105
##     dCRET                    0.232    0.134    1.732    0.083   -0.030    0.494
##     dCRET                    0.367    0.171    2.149    0.032    0.032    0.701
##     dCRET                    0.876    0.129    6.818    0.000    0.624    1.128
##     dCRET                   -0.230    0.076   -3.026    0.002   -0.380   -0.081
##     dCRET                    0.074    0.092    0.806    0.420   -0.106    0.255
##     dCRET                    0.798    0.220    3.627    0.000    0.367    1.229
##     dCRET                   -0.283    0.122   -2.326    0.020   -0.522   -0.045
##    Std.lv  Std.all
##
##     0.291    0.291
##     0.252    0.126
##
##    -1.722   -0.812
##
##    -0.265   -0.071
##    -0.147   -0.084
##    -0.429   -0.058
##    -0.015   -0.004
##    -1.065   -0.287
##
##    -0.135   -0.286
##     0.138    0.138
##
##    -0.966   -0.447
##
##    -0.043   -0.021
##
##    -1.045   -0.378
##
##    -0.026   -0.048
```

```
##    -0.021   -0.021
##    -0.286   -0.113
##     0.040    0.017
##     0.104    0.050
##    -0.008   -0.042
##    -0.022   -0.019
##    -0.011   -0.010
##    -0.161   -0.071
##    -0.241   -0.231
##    -0.155   -0.144
##    -0.162   -0.117
##    -0.061   -0.014
##    -0.078   -0.035
##
##     0.030    0.027
##    -0.363   -0.180
##     0.745    0.146
##     0.099    0.021
##    -0.001   -0.000
##     0.120    0.328
##    -0.155   -0.066
##     0.148    0.063
##     0.235    0.051
##     0.560    0.268
##    -0.147   -0.068
##     0.047    0.017
##     0.511    0.060
##    -0.181   -0.040
##
## Covariances:
##                              Estimate  Std.Err  z-value  P(>|z|) ci.lower
##   .cr_genderedactivities ~~
##     .dCRET20_female             0.317    0.024   13.146    0.000    0.270
##   .cr_gendernormscale_f4 ~~
##     .cr_ghq12                   0.863    0.200    4.310    0.000    0.471
##     .dCRET20_female            -0.059    0.025   -2.371    0.018   -0.107
##   .cr_media ~~
##     .cr_gendrdctvts             1.393    0.163    8.551    0.000    1.074
##   .cr_copingresilience ~~
##     .cr_gndrnrmsc_2            -0.376    0.169   -2.225    0.026   -0.708
##  ci.upper   Std.lv  Std.all
##
##     0.364    0.317    0.538
##
##     1.256    0.863    0.159
##    -0.010   -0.059   -0.080
##
##     1.713    1.393      Inf
##
##    -0.045   -0.376   -0.084
##
## Variances:
##                      Estimate  Std.Err  z-value  P(>|z|) ci.lower ci.upper
##     .cr_talk            1.000    0.057   17.459    0.000    0.887    1.112
##     .cr_copingrslnc     7.297    0.989    7.376    0.000    5.358    9.236
##     .cr_media           0.000                               0.000    0.000
```

```
##    .cr_gndrnrmsc_1    3.328    0.159   20.911    0.000    3.016    3.640
##    .cr_gndrnrmsc_2    2.756    0.144   19.170    0.000    2.474    3.038
##    .cr_gndrnrmsc_4    2.347    0.130   18.060    0.000    2.092    2.602
##    .cr_gendrdctvts    1.530    0.343    4.461    0.000    0.858    2.203
##    .cr_ghq12         12.612    0.858   14.694    0.000   10.930   14.294
##    .dCRET20_female    0.227    0.004   63.526    0.000    0.220    0.234
##    .Competencies      0.314    0.043    7.342    0.000    0.230    0.398
##    .GenderNorms       1.650    0.058   28.558    0.000    1.537    1.764
##    .GenderAttituds    0.922    0.105    8.791    0.000    0.717    1.128
##    Std.lv  Std.all
##     1.000    0.725
##     7.297    0.484
##     0.000    0.000
##     3.328    0.676
##     2.756    0.627
##     2.347    0.643
##     1.530    0.341
##    12.612    0.918
##     0.227    0.914
##     0.829    0.829
##     0.675    0.675
##     0.579    0.579
##
## R-Square:
##                    Estimate
##     cr_talk           0.275
##     cr_copingrslnc    0.516
##     cr_media          1.000
##     cr_gndrnrmsc_1    0.324
##     cr_gndrnrmsc_2    0.373
##     cr_gndrnrmsc_4    0.357
##     cr_gendrdctvts    0.659
##     cr_ghq12          0.082
##     dCRET20_female    0.086
##     Competencies      0.171
##     GenderNorms       0.325
##     GenderAttituds    0.421
##
## Defined Parameters:
##                    Estimate  Std.Err  z-value  P(>|z|) ci.lower ci.upper
##     partGNGAMH       -0.049    0.028   -1.772    0.076   -0.104    0.005
##     partGNGRMH        0.162    0.082    1.983    0.047    0.002    0.322
##     partGNGRCOM       0.092    0.025    3.698    0.000    0.043    0.140
##     partSexGAMH      -0.067    0.041   -1.635    0.102   -0.147    0.013
##     tieGNMH           0.113    0.082    1.380    0.168   -0.047    0.273
##     tieGNCOM          0.092    0.025    3.698    0.000    0.043    0.140
##     tieSexMH         -0.067    0.041   -1.635    0.102   -0.147    0.013
##     teSexMH          -0.496    0.218   -2.270    0.023   -0.924   -0.068
##     teGNMH            0.103    0.069    1.491    0.136   -0.033    0.239
##     teGNCOM           0.146    0.020    7.318    0.000    0.107    0.185
##     med_propSexMH     0.135    0.107    1.262    0.207   -0.074    0.344
##     med_propGNGAMH   -0.475    0.403   -1.178    0.239   -1.266    0.315
##     med_propGNGRMH    1.566    0.975    1.605    0.108   -0.346    3.478
##     med_prpGNGRCOM    0.627    0.145    4.329    0.000    0.343    0.912
##    Std.lv  Std.all
##    -0.077   -0.021
```

```
##     0.253    0.068
##     0.233    0.233
##    -0.067   -0.009
##     0.176    0.048
##     0.233    0.233
##    -0.067   -0.009
##    -0.496   -0.067
##     0.162    0.044
##     0.371    0.371
##     0.135    0.135
##    -0.475   -0.475
##     1.566    1.566
##     0.627    0.627
```

```
#fitMeasures(fit.testmPUBPeerSen)

#parameterEstimates(fit.testmPUBPeerSen, standardized = TRUE)
#modindices(fit.testmPUBPeerSen, sort. = TRUE)
```

##variable club member removed

```
testmPUBPeerSen <- '
Competencies =~ cr_talk + cr_copingresilience
GenderNorms =~ cr_media  +  dCRET20_rolem_y
GenderAttitudes =~ cr_gendernormscale_f1 + cr_gendernormscale_f2 +  cr_gendernormscale_
f4

#mediations/regressions
GenderAttitudes ~ aGN_GA * GenderNorms + aSex_GA * dCRET20_female
cr_genderedactivities ~ aGN_GR * GenderNorms
cr_ghq12~ bGA_MH * GenderAttitudes + bGR_MH * cr_genderedactivities + cSexMH * dCRET20_
female + cGNMH * GenderNorms + Competencies
Competencies ~ bGR_COM * cr_genderedactivities + cGNCOM * GenderNorms#new

#Modindizes: covariances to improve model fit
cr_genderedactivities ~~ dCRET20_female
cr_gendernormscale_f4 ~~ cr_ghq12
cr_gendernormscale_f4 ~~ dCRET20_female
cr_media ~~ cr_genderedactivities
cr_copingresilience ~~ cr_gendernormscale_f2

#regressions MIs: latent variable + covariate is ok!
GenderAttitudes ~ dCRET20_languagehh_201
Competencies ~ dCRET20_urban
#Competencies ~ af_asset_10 makes gender norms ->mh negative
GenderAttitudes ~ dCRET20_languagehh_202

#partial indirect effects
partGNGAMH := aGN_GA * bGA_MH
partGNGRMH := aGN_GR * bGR_MH
partGNGRCOM  := aGN_GR * bGR_COM
partSexGAMH := aSex_GA * bGA_MH

#total indirect effects
tieGNMH := partGNGAMH + partGNGRMH
tieGNCOM := partGNGRCOM
tieSexMH:= partSexGAMH

#total effect
teSexMH := tieSexMH + cSexMH
teGNMH := tieGNMH + cGNMH
teGNCOM := tieGNCOM + cGNCOM#new

med_propSexMH := partSexGAMH / teSexMH
med_propGNGAMH := partGNGAMH / teGNMH
med_propGNGRMH := partGNGRMH / teGNMH
med_propGNGRCOM := partGNGRCOM / teGNCOM

#covariates to the two exogeneous variables (or to the ones the covariates apply)
dCRET20_female~cr_cs_crage + dCRET20_urban + dCRET20_married_y + dCRET20_purpsamp_y + d
CRET20_disability_y + af_asset_10 + dCRET20_fatherinhh +  dCRET20_aspiratt_sec +
dCRET20_aspiratt_teach + dCRET20_aspiratt_uni +  dCRET20_languagehh_201 + dCRET20_langu
agehh_202 + dCRET20_languagehh_211  + dCRET20_workfambus_y

GenderNorms~cr_cs_crage + dCRET20_urban + dCRET20_married_y +dCRET20_purpsamp_y + dCRET
20_disability_y + af_asset_10 + dCRET20_fatherinhh +  dCRET20_aspiratt_sec +
```

```
dCRET20_aspiratt_teach + dCRET20_aspiratt_uni +  dCRET20_languagehh_201 + dCRET20_langu
agehh_202 + dCRET20_languagehh_211  + dCRET20_workfambus_y
'

fit.testmPUBPeerSen <- sem(testmPUBPeerSen, data=subsetSEMNeu, estimator="MLR", samplin
g.weights = "rand_weight")
summary(fit.testmPUBPeerSen, fit.measures=TRUE, standardized=TRUE, rsq=TRUE, ci=TRUE)
```

```
## lavaan 0.6-21 ended normally after 113 iterations
##
##   Estimator                                         ML
##   Optimization method                           NLMINB
##   Number of model parameters                        63
##
##                                                   Used       Total
##   Number of observations                          1651        4777
##   Sampling weights variable                 rand_weight
##
## Model Test User Model:
##                                               Standard      Scaled
##   Test Statistic                               927.441     772.051
##   Degrees of freedom                               132         132
##   P-value (Chi-square)                           0.000       0.000
##   Scaling correction factor                                  1.201
##     Yuan-Bentler correction (Mplus variant)
##
## Model Test Baseline Model:
##
##   Test statistic                              4615.327    3844.697
##   Degrees of freedom                               185         185
##   P-value                                        0.000       0.000
##   Scaling correction factor                                  1.200
##
## User Model versus Baseline Model:
##
##   Comparative Fit Index (CFI)                    0.820       0.825
##   Tucker-Lewis Index (TLI)                       0.748       0.755
##
##   Robust Comparative Fit Index (CFI)                         0.825
##   Robust Tucker-Lewis Index (TLI)                            0.755
##
## Loglikelihood and Information Criteria:
##
##   Loglikelihood user model (H0)             -29558.045  -29558.045
##   Scaling correction factor                                  1.205
##       for the MLR correction
##   Loglikelihood unrestricted model (H1)     -29094.324  -29094.324
##   Scaling correction factor                                  1.203
##       for the MLR correction
##
##   Akaike (AIC)                               59242.089   59242.089
##   Bayesian (BIC)                             59582.865   59582.865
##   Sample-size adjusted Bayesian (SABIC)      59382.724   59382.724
##
## Root Mean Square Error of Approximation:
##
##   RMSEA                                          0.060       0.054
##   90 Percent confidence interval - lower         0.057       0.051
##   90 Percent confidence interval - upper         0.064       0.058
##   P-value H_0: RMSEA <= 0.050                    0.000       0.020
##   P-value H_0: RMSEA >= 0.080                    0.000       0.000
##
##   Robust RMSEA                                               0.059
```

```
##   90 Percent confidence interval - lower                     0.055
##   90 Percent confidence interval - upper                     0.063
##   P-value H_0: Robust RMSEA <= 0.050                         0.000
##   P-value H_0: Robust RMSEA >= 0.080                         0.000
##
## Standardized Root Mean Square Residual:
##
##   SRMR                                         0.037       0.037
##
## Parameter Estimates:
##
##   Standard errors                           Sandwich
##   Information bread                         Observed
##   Observed information based on              Hessian
##
## Latent Variables:
##                      Estimate  Std.Err  z-value  P(>|z|) ci.lower ci.upper
##   Competencies =~
##     cr_talk             1.000                               1.000    1.000
##     cr_copingrslnc      3.725    0.305   12.217    0.000    3.127    4.322
##   GenderNorms =~
##     cr_media            1.000                               1.000    1.000
##     dCRET20_rolm_y      0.213    0.016   13.199    0.000    0.181    0.245
##   GenderAttitudes =~
##     cr_gndrnrmsc_1      1.000                               1.000    1.000
##     cr_gndrnrmsc_2      0.897    0.060   14.835    0.000    0.779    1.016
##     cr_gndrnrmsc_4      0.888    0.062   14.329    0.000    0.767    1.010
##    Std.lv  Std.all
##
##     0.676    0.576
##     2.517    0.648
##
##     0.920    0.588
##     0.196    0.393
##
##     1.321    0.595
##     1.185    0.567
##     1.174    0.614
##
## Regressions:
##                           Estimate  Std.Err  z-value  P(>|z|) ci.lower ci.upper
##   GenderAttitudes ~
##     GndrN (aGN_GA)           0.883    0.090    9.834    0.000    0.707    1.059
##     dCRET   (aS_G)           0.536    0.091    5.918    0.000    0.358    0.713
##   cr_genderedactivities ~
##     GndrN (aGN_GR)          -1.374    0.084  -16.370    0.000   -1.539   -1.210
##   cr_ghq12 ~
##     GndrA   (bGA_)          -0.096    0.186   -0.513    0.608   -0.461    0.269
##     cr_gn  (bGR_M)          -0.093    0.067   -1.384    0.166   -0.224    0.039
##     dCRET   (cSMH)          -0.501    0.238   -2.108    0.035   -0.968   -0.035
##     GndrN   (cGNM)           0.209    0.320    0.653    0.514   -0.418    0.835
##     Cmptn                   -1.697    0.381   -4.450    0.000   -2.444   -0.949
##   Competencies ~
##     cr_gn  (bGR_C)           0.004    0.018    0.228    0.820   -0.032    0.040
##     GndrN   (cGNC)           0.623    0.075    8.360    0.000    0.477    0.769
##   GenderAttitudes ~
```

```
##      dCRET                  -0.710    0.116   -6.107    0.000   -0.938   -0.482
##    Competencies ~
##      dCRET                   0.310    0.061    5.080    0.000    0.190    0.429
##    GenderAttitudes ~
##      dCRET                  -0.674    0.148   -4.543    0.000   -0.964   -0.383
##    dCRET20_female ~
##      cr_c_                  -0.027    0.014   -1.967    0.049   -0.054   -0.000
##      dCRET                  -0.022    0.027   -0.790    0.429   -0.076    0.032
##      dCRET                  -0.277    0.057   -4.866    0.000   -0.389   -0.166
##      dCRET                   0.046    0.065    0.711    0.477   -0.081    0.173
##      dCRET                   0.082    0.054    1.509    0.131   -0.024    0.188
##      a__10                  -0.004    0.005   -0.880    0.379   -0.014    0.005
##      dCRET                  -0.038    0.029   -1.300    0.194   -0.095    0.019
##      dCRET                  -0.033    0.058   -0.567    0.571   -0.146    0.080
##      dCRET                  -0.185    0.079   -2.348    0.019   -0.339   -0.031
##      dCRET                  -0.281    0.055   -5.066    0.000   -0.390   -0.172
##      dCRET                  -0.121    0.029   -4.148    0.000   -0.179   -0.064
##      dCRET                  -0.116    0.039   -2.960    0.003   -0.193   -0.039
##      dCRET                   0.004    0.102    0.040    0.968   -0.196    0.204
##      dCRET                  -0.078    0.055   -1.410    0.158   -0.186    0.030
##    GenderNorms ~
##      cr_c_                   0.049    0.025    1.985    0.047    0.001    0.097
##      dCRET                  -0.425    0.058   -7.292    0.000   -0.540   -0.311
##      dCRET                   0.830    0.133    6.240    0.000    0.569    1.091
##      dCRET                   0.055    0.110    0.506    0.613   -0.159    0.270
##      dCRET                   0.246    0.086    2.867    0.004    0.078    0.415
##      a__10                   0.129    0.013    9.573    0.000    0.103    0.155
##      dCRET                  -0.053    0.051   -1.038    0.299   -0.153    0.047
##      dCRET                   0.296    0.118    2.519    0.012    0.066    0.526
##      dCRET                   0.500    0.131    3.814    0.000    0.243    0.758
##      dCRET                   1.014    0.108    9.400    0.000    0.802    1.225
##      dCRET                  -0.466    0.054   -8.579    0.000   -0.572   -0.359
##      dCRET                  -0.335    0.075   -4.462    0.000   -0.481   -0.188
##      dCRET                  -0.146    0.170   -0.860    0.390   -0.479    0.187
##      dCRET                  -0.190    0.080   -2.364    0.018   -0.347   -0.032
##     Std.lv  Std.all
##
##      0.615    0.615
##      0.406    0.202
##
##     -1.264   -0.593
##
##     -0.126   -0.034
##     -0.093   -0.053
##     -0.501   -0.067
##      0.192    0.052
##     -1.146   -0.309
##
##      0.006    0.013
##      0.848    0.848
##
##     -0.538   -0.249
##
##      0.459    0.228
##
##     -0.510   -0.184
```

```
##
##    -0.027   -0.050
##    -0.022   -0.022
##    -0.277   -0.109
##     0.046    0.019
##     0.082    0.040
##    -0.004   -0.024
##    -0.038   -0.032
##    -0.033   -0.028
##    -0.185   -0.081
##    -0.281   -0.270
##    -0.121   -0.113
##    -0.116   -0.084
##     0.004    0.001
##    -0.078   -0.035
##
##     0.053    0.049
##    -0.462   -0.230
##     0.902    0.177
##     0.060    0.013
##     0.268    0.064
##     0.140    0.383
##    -0.058   -0.025
##     0.322    0.136
##     0.544    0.120
##     1.102    0.526
##    -0.506   -0.234
##    -0.364   -0.131
##    -0.159   -0.019
##    -0.206   -0.046
##
## Covariances:
##                              Estimate  Std.Err  z-value  P(>|z|) ci.lower
##  .cr_genderedactivities ~~
##    .dCRET20_female              0.291    0.024   12.208    0.000    0.244
##  .cr_gendernormscale_f4 ~~
##    .cr_ghq12                    0.857    0.201    4.263    0.000    0.463
##    .dCRET20_female             -0.072    0.025   -2.902    0.004   -0.121
##  .cr_media ~~
##    .cr_gendrdctvts             -0.140    0.069   -2.025    0.043   -0.275
##  .cr_copingresilience ~~
##    .cr_gndrnrmsc_2             -0.612    0.170   -3.594    0.000   -0.946
##  ci.upper   Std.lv  Std.all
##
##     0.337    0.291    0.356
##
##     1.251    0.857    0.157
##    -0.023   -0.072   -0.099
##
##    -0.004   -0.140   -0.064
##
##    -0.278   -0.612   -0.120
##
## Variances:
##                      Estimate  Std.Err  z-value  P(>|z|) ci.lower ci.upper
##    .cr_talk            0.917    0.049   18.546    0.000    0.820    1.014
```

```
##     .cr_copingrslnc    8.745    0.619   14.136    0.000    7.532    9.957
##     .cr_media          1.602    0.074   21.674    0.000    1.457    1.747
##     .dCRET20_rolm_y    0.211    0.005   44.742    0.000    0.201    0.220
##     .cr_gndrnrmsc_1    3.181    0.155   20.543    0.000    2.878    3.485
##     .cr_gndrnrmsc_2    2.960    0.141   21.034    0.000    2.684    3.236
##     .cr_gndrnrmsc_4    2.343    0.124   18.874    0.000    2.100    2.587
##     .cr_gendrdctvts    2.944    0.174   16.925    0.000    2.603    3.285
##     .cr_ghq12         12.685    0.876   14.483    0.000   10.969   14.402
##     .dCRET20_female    0.226    0.004   64.442    0.000    0.219    0.233
##     .Competencies      0.192    0.034    5.652    0.000    0.126    0.259
##     .GenderNorms       0.217    0.029    7.407    0.000    0.160    0.274
##     .GenderAttituds    0.660    0.092    7.200    0.000    0.481    0.840
##     Std.lv  Std.all
##      0.917    0.668
##      8.745    0.580
##      1.602    0.654
##      0.211    0.846
##      3.181    0.646
##      2.960    0.678
##      2.343    0.642
##      2.944    0.648
##     12.685    0.920
##      0.226    0.912
##      0.421    0.421
##      0.257    0.257
##      0.379    0.379
##
## R-Square:
##                    Estimate
##      cr_talk          0.332
##      cr_copingrslnc   0.420
##      cr_media         0.346
##      dCRET20_rolm_y   0.154
##      cr_gndrnrmsc_1   0.354
##      cr_gndrnrmsc_2   0.322
##      cr_gndrnrmsc_4   0.358
##      cr_gendrdctvts   0.352
##      cr_ghq12         0.080
##      dCRET20_female   0.088
##      Competencies     0.579
##      GenderNorms      0.743
##      GenderAttituds   0.621
##
## Defined Parameters:
##                    Estimate  Std.Err  z-value  P(>|z|) ci.lower ci.upper
##      partGNGAMH      -0.084    0.165   -0.511    0.609   -0.408    0.239
##      partGNGRMH       0.127    0.092    1.378    0.168   -0.054    0.308
##      partGNGRCOM     -0.006    0.025   -0.227    0.820   -0.056    0.044
##      partSexGAMH     -0.051    0.100   -0.510    0.610   -0.248    0.146
##      tieGNMH          0.043    0.195    0.219    0.826   -0.340    0.426
##      tieGNCOM        -0.006    0.025   -0.227    0.820   -0.056    0.044
##      tieSexMH        -0.051    0.100   -0.510    0.610   -0.248    0.146
##      teSexMH         -0.553    0.217   -2.550    0.011   -0.977   -0.128
##      teGNMH           0.252    0.227    1.108    0.268   -0.193    0.696
##      teGNCOM          0.617    0.063    9.851    0.000    0.494    0.740
##      med_propSexMH    0.093    0.185    0.502    0.616   -0.270    0.455
```

```
##     med_propGNGAMH   -0.336    0.701   -0.478    0.632   -1.710    1.039
##     med_propGNGRMH    0.506    0.630    0.803    0.422   -0.728    1.740
##     med_prpGNGRCOM   -0.009    0.041   -0.229    0.819   -0.090    0.071
##    Std.lv  Std.all
##    -0.078   -0.021
##     0.117    0.032
##    -0.008   -0.008
##    -0.051   -0.007
##     0.039    0.011
##    -0.008   -0.008
##    -0.051   -0.007
##    -0.553   -0.074
##     0.231    0.062
##     0.840    0.840
##     0.093    0.093
##    -0.336   -0.336
##     0.506    0.506
##    -0.009   -0.009
```

```
fitMeasures(fit.testmPUBPeerSen)
```

```
##                          npar                          fmin 
##                        63.000                         0.281 
##                         chisq                            df 
##                       927.441                       132.000 
##                        pvalue                  chisq.scaled 
##                         0.000                       772.051 
##                     df.scaled                 pvalue.scaled 
##                       132.000                         0.000 
##          chisq.scaling.factor                baseline.chisq 
##                         1.201                      4615.327 
##                   baseline.df               baseline.pvalue 
##                       185.000                         0.000 
##         baseline.chisq.scaled            baseline.df.scaled 
##                      3844.697                       185.000 
##        baseline.pvalue.scaled baseline.chisq.scaling.factor 
##                         0.000                         1.200 
##                           cfi                           tli 
##                         0.820                         0.748 
##                    cfi.scaled                    tli.scaled 
##                         0.825                         0.755 
##                    cfi.robust                    tli.robust 
##                         0.825                         0.755 
##                          nnfi                           rfi 
##                         0.748                         0.718 
##                           nfi                          pnfi 
##                         0.799                         0.570 
##                           ifi                           rni 
##                         0.823                         0.820 
##                   nnfi.scaled                    rfi.scaled 
##                         0.755                         0.719 
##                    nfi.scaled                   pnfi.scaled 
##                         0.799                         0.570 
##                    ifi.scaled                    rni.scaled 
##                         0.828                         0.825 
##                   nnfi.robust                    rni.robust 
##                         0.755                         0.825 
##                          logl             unrestricted.logl 
##                    -29558.045                    -29094.324 
##                           aic                           bic 
##                     59242.089                     59582.865 
##                        ntotal                          bic2 
##                      1651.000                     59382.724 
##             scaling.factor.h1             scaling.factor.h0 
##                         1.203                         1.205 
##                         rmsea                rmsea.ci.lower 
##                         0.060                         0.057 
##                rmsea.ci.upper                rmsea.ci.level 
##                         0.064                         0.900 
##                  rmsea.pvalue                rmsea.close.h0 
##                         0.000                         0.050 
##         rmsea.notclose.pvalue             rmsea.notclose.h0 
##                         0.000                         0.080 
##                  rmsea.scaled         rmsea.ci.lower.scaled 
##                         0.054                         0.051 
##         rmsea.ci.upper.scaled           rmsea.pvalue.scaled 
```

```
##                          0.058                          0.020
##  rmsea.notclose.pvalue.scaled                   rmsea.robust
##                          0.000                          0.059
##         rmsea.ci.lower.robust         rmsea.ci.upper.robust
##                          0.055                          0.063
##           rmsea.pvalue.robust  rmsea.notclose.pvalue.robust
##                          0.000                          0.000
##                            rmr                     rmr_nomean
##                          0.096                          0.096
##                           srmr                   srmr_bentler
##                          0.037                          0.037
##           srmr_bentler_nomean                           crmr
##                          0.037                          0.038
##                   crmr_nomean                     srmr_mplus
##                          0.038                          0.037
##             srmr_mplus_nomean                          cn_05
##                          0.037                        285.495
##                          cn_01                            gfi
##                        308.454                          0.909
##                           agfi                           pgfi
##                          0.793                          0.400
##                            mfi                           ecvi
##                          0.786                          0.638
## attr(,"scaled.test")
## [1] "yuan.bentler.mplus"
```

```
#parameterEstimates(fit.testmPUBPeerSen, standardized = TRUE)
#modindices(fit.testmPUBPeerSen, sort. = TRUE)
```

##variable role model removed

```
testmPUBPeerSen <- '
Competencies =~ cr_talk + cr_copingresilience
GenderNorms =~ cr_media  +  dCRET20_clubm_y
GenderAttitudes =~ cr_gendernormscale_f1 + cr_gendernormscale_f2 +  cr_gendernormscale_
f4 #+ cr_gendernormscale_f5 + cr_gendernormscale_f6 + cr_gendernormscale_f7 cr_genderno
rmscale_f3 +

#mediations/regressions
GenderAttitudes ~ aGN_GA * GenderNorms + aSex_GA * dCRET20_female
cr_genderedactivities ~ aGN_GR * GenderNorms
cr_ghq12~ bGA_MH * GenderAttitudes + bGR_MH * cr_genderedactivities + cSexMH * dCRET20_
female + cGNMH * GenderNorms + Competencies
Competencies ~ bGR_COM * cr_genderedactivities + cGNCOM * GenderNorms#new

#Modindizes: covariances to improve model fit
cr_genderedactivities ~~ dCRET20_female
cr_gendernormscale_f4 ~~ cr_ghq12
cr_gendernormscale_f4 ~~ dCRET20_female
cr_media ~~ cr_genderedactivities
cr_copingresilience ~~ cr_gendernormscale_f2

#regressions MIs: latent variable + covariate is ok!
GenderAttitudes ~ dCRET20_languagehh_201
Competencies ~ dCRET20_urban
#Competencies ~ af_asset_10 makes gender norms ->mh negative
GenderAttitudes ~ dCRET20_languagehh_202

#partial indirect effects
partGNGAMH := aGN_GA * bGA_MH
partGNGRMH := aGN_GR * bGR_MH
partGNGRCOM  := aGN_GR * bGR_COM
partSexGAMH := aSex_GA * bGA_MH

#total indirect effects
tieGNMH := partGNGAMH + partGNGRMH
tieGNCOM := partGNGRCOM
tieSexMH:= partSexGAMH

#total effect
teSexMH := tieSexMH + cSexMH
teGNMH := tieGNMH + cGNMH
teGNCOM := tieGNCOM + cGNCOM#new

med_propSexMH := partSexGAMH / teSexMH
med_propGNGAMH := partGNGAMH / teGNMH
med_propGNGRMH := partGNGRMH / teGNMH
med_propGNGRCOM := partGNGRCOM / teGNCOM

#covariates to the two exogeneous variables (or to the ones the covariates apply)
dCRET20_female~cr_cs_crage + dCRET20_urban + dCRET20_married_y + dCRET20_purpsamp_y + d
CRET20_disability_y + af_asset_10 + dCRET20_fatherinhh +  dCRET20_aspiratt_sec +
dCRET20_aspiratt_teach + dCRET20_aspiratt_uni +  dCRET20_languagehh_201 + dCRET20_langu
agehh_202 + dCRET20_languagehh_211  + dCRET20_workfambus_y

GenderNorms~cr_cs_crage + dCRET20_urban + dCRET20_married_y +dCRET20_purpsamp_y + dCRET
```

```
20_disability_y + af_asset_10 + dCRET20_fatherinhh +  dCRET20_aspiratt_sec +
dCRET20_aspiratt_teach + dCRET20_aspiratt_uni +  dCRET20_languagehh_201 + dCRET20_langu
agehh_202 + dCRET20_languagehh_211  + dCRET20_workfambus_y
'


fit.testmPUBPeerSen <- sem(testmPUBPeerSen, data=subsetSEMNeu, estimator="MLR", samplin
g.weights = "rand_weight")
summary(fit.testmPUBPeerSen, fit.measures=TRUE, standardized=TRUE, rsq=TRUE, ci=TRUE)
```

```
## lavaan 0.6-21 ended normally after 112 iterations
##
##   Estimator                                         ML
##   Optimization method                           NLMINB
##   Number of model parameters                        63
##
##                                                   Used       Total
##   Number of observations                          1654        4777
##   Sampling weights variable                    rand_weight
##
## Model Test User Model:
##                                               Standard      Scaled
##   Test Statistic                               899.048     754.965
##   Degrees of freedom                               132         132
##   P-value (Chi-square)                           0.000       0.000
##   Scaling correction factor                                  1.191
##     Yuan-Bentler correction (Mplus variant)
##
## Model Test Baseline Model:
##
##   Test statistic                              4623.206    3873.370
##   Degrees of freedom                               185         185
##   P-value                                        0.000       0.000
##   Scaling correction factor                                  1.194
##
## User Model versus Baseline Model:
##
##   Comparative Fit Index (CFI)                    0.827       0.831
##   Tucker-Lewis Index (TLI)                       0.758       0.763
##
##   Robust Comparative Fit Index (CFI)                         0.831
##   Robust Tucker-Lewis Index (TLI)                            0.764
##
## Loglikelihood and Information Criteria:
##
##   Loglikelihood user model (H0)             -29591.526  -29591.526
##   Scaling correction factor                                  1.208
##       for the MLR correction
##   Loglikelihood unrestricted model (H1)     -29142.002  -29142.002
##   Scaling correction factor                                  1.196
##       for the MLR correction
##
##   Akaike (AIC)                               59309.053   59309.053
##   Bayesian (BIC)                             59649.943   59649.943
##   Sample-size adjusted Bayesian (SABIC)      59449.801   59449.801
##
## Root Mean Square Error of Approximation:
##
##   RMSEA                                          0.059       0.053
##   90 Percent confidence interval - lower         0.056       0.050
##   90 Percent confidence interval - upper         0.063       0.057
##   P-value H_0: RMSEA <= 0.050                    0.000       0.047
##   P-value H_0: RMSEA >= 0.080                    0.000       0.000
##
##   Robust RMSEA                                               0.058
```

```
##   90 Percent confidence interval - lower                     0.054
##   90 Percent confidence interval - upper                     0.062
##   P-value H_0: Robust RMSEA <= 0.050                         0.000
##   P-value H_0: Robust RMSEA >= 0.080                         0.000
##
## Standardized Root Mean Square Residual:
##
##   SRMR                                           0.037       0.037
##
## Parameter Estimates:
##
##   Standard errors                             Sandwich
##   Information bread                           Observed
##   Observed information based on                Hessian
##
## Latent Variables:
##                      Estimate  Std.Err  z-value  P(>|z|) ci.lower ci.upper
##   Competencies =~
##     cr_talk             1.000                               1.000    1.000
##     cr_copingrslnc      3.650    0.300   12.167    0.000    3.062    4.238
##   GenderNorms =~
##     cr_media            1.000                               1.000    1.000
##     dCRET20_clbm_y      0.228    0.017   13.615    0.000    0.196    0.261
##   GenderAttitudes =~
##     cr_gndrnrmsc_1      1.000                               1.000    1.000
##     cr_gndrnrmsc_2      0.898    0.061   14.787    0.000    0.779    1.017
##     cr_gndrnrmsc_4      0.890    0.062   14.264    0.000    0.768    1.012
##    Std.lv  Std.all
##
##     0.681    0.580
##     2.485    0.641
##
##     0.918    0.587
##     0.210    0.422
##
##     1.320    0.595
##     1.185    0.567
##     1.175    0.615
##
## Regressions:
##                           Estimate  Std.Err  z-value  P(>|z|) ci.lower ci.upper
##   GenderAttitudes ~
##     GndrN (aGN_GA)           0.866    0.087    9.935    0.000    0.695    1.037
##     dCRET   (aS_G)           0.524    0.090    5.817    0.000    0.347    0.700
##   cr_genderedactivities ~
##     GndrN (aGN_GR)          -1.382    0.084  -16.546    0.000   -1.546   -1.218
##   cr_ghq12 ~
##     GndrA   (bGA_)          -0.093    0.173   -0.536    0.592   -0.431    0.246
##     cr_gn  (bGR_M)          -0.101    0.066   -1.538    0.124   -0.230    0.028
##     dCRET   (cSMH)          -0.491    0.237   -2.070    0.038   -0.957   -0.026
##     GndrN   (cGNM)           0.167    0.305    0.548    0.583   -0.430    0.765
##     Cmptn                   -1.657    0.388   -4.270    0.000   -2.418   -0.897
##   Competencies ~
##     cr_gn  (bGR_C)           0.005    0.018    0.264    0.792   -0.031    0.041
##     GndrN   (cGNC)           0.631    0.073    8.654    0.000    0.488    0.774
##   GenderAttitudes ~
```

```
##     dCRET                      -0.787    0.111   -7.073    0.000   -1.005   -0.569
##   Competencies ~
##     dCRET                       0.304    0.061    4.991    0.000    0.185    0.424
##   GenderAttitudes ~
##     dCRET                      -0.631    0.149   -4.226    0.000   -0.923   -0.338
##   dCRET20_female ~
##     cr_c_                      -0.028    0.014   -2.079    0.038   -0.055   -0.002
##     dCRET                      -0.024    0.027   -0.891    0.373   -0.077    0.029
##     dCRET                      -0.281    0.056   -5.015    0.000   -0.391   -0.171
##     dCRET                       0.047    0.064    0.734    0.463   -0.078    0.171
##     dCRET                       0.078    0.055    1.418    0.156   -0.030    0.186
##     a__10                      -0.004    0.005   -0.894    0.371   -0.014    0.005
##     dCRET                      -0.042    0.029   -1.455    0.146   -0.098    0.015
##     dCRET                      -0.040    0.057   -0.706    0.480   -0.152    0.072
##     dCRET                      -0.199    0.078   -2.553    0.011   -0.351   -0.046
##     dCRET                      -0.289    0.055   -5.268    0.000   -0.397   -0.182
##     dCRET                      -0.130    0.029   -4.478    0.000   -0.187   -0.073
##     dCRET                      -0.110    0.039   -2.827    0.005   -0.187   -0.034
##     dCRET                      -0.003    0.104   -0.029    0.977   -0.206    0.200
##     dCRET                      -0.069    0.055   -1.255    0.210   -0.177    0.039
##   GenderNorms ~
##     cr_c_                       0.054    0.024    2.199    0.028    0.006    0.102
##     dCRET                      -0.417    0.058   -7.187    0.000   -0.530   -0.303
##     dCRET                       0.854    0.133    6.417    0.000    0.593    1.115
##     dCRET                       0.035    0.109    0.324    0.746   -0.178    0.248
##     dCRET                       0.272    0.085    3.218    0.001    0.106    0.438
##     a__10                       0.129    0.013    9.759    0.000    0.103    0.155
##     dCRET                      -0.031    0.051   -0.610    0.542   -0.130    0.068
##     dCRET                       0.343    0.113    3.033    0.002    0.121    0.564
##     dCRET                       0.595    0.129    4.606    0.000    0.342    0.848
##     dCRET                       1.052    0.104   10.120    0.000    0.848    1.256
##     dCRET                      -0.395    0.055   -7.214    0.000   -0.502   -0.288
##     dCRET                      -0.380    0.070   -5.414    0.000   -0.518   -0.243
##     dCRET                      -0.087    0.166   -0.521    0.602   -0.412    0.239
##     dCRET                      -0.243    0.084   -2.893    0.004   -0.408   -0.078
##    Std.lv  Std.all
##
##     0.602    0.602
##     0.397    0.198
##
##    -1.269   -0.594
##
##    -0.122   -0.033
##    -0.101   -0.058
##    -0.491   -0.066
##     0.153    0.041
##    -1.128   -0.304
##
##     0.007    0.015
##     0.851    0.851
##
##    -0.596   -0.276
##
##     0.447    0.222
##
##    -0.478   -0.173
```

```
##
##    -0.028   -0.052
##    -0.024   -0.024
##    -0.281   -0.111
##     0.047    0.020
##     0.078    0.038
##    -0.004   -0.024
##    -0.042   -0.036
##    -0.040   -0.034
##    -0.199   -0.087
##    -0.289   -0.277
##    -0.130   -0.120
##    -0.110   -0.080
##    -0.003   -0.001
##    -0.069   -0.031
##
##     0.059    0.054
##    -0.454   -0.226
##     0.930    0.182
##     0.038    0.008
##     0.296    0.071
##     0.141    0.385
##    -0.034   -0.014
##     0.373    0.158
##     0.648    0.142
##     1.146    0.547
##    -0.430   -0.199
##    -0.414   -0.150
##    -0.094   -0.011
##    -0.265   -0.058
##
## Covariances:
##                               Estimate  Std.Err  z-value  P(>|z|) ci.lower
##  .cr_genderedactivities ~~
##    .dCRET20_female              0.297    0.024   12.477    0.000    0.250
##  .cr_gendernormscale_f4 ~~
##    .cr_ghq12                    0.867    0.200    4.327    0.000    0.474
##    .dCRET20_female             -0.073    0.025   -2.955    0.003   -0.122
##  .cr_media ~~
##    .cr_gendrdctvts             -0.138    0.069   -1.989    0.047   -0.273
##  .cr_copingresilience ~~
##    .cr_gndrnrmsc_2             -0.556    0.170   -3.272    0.001   -0.888
##  ci.upper   Std.lv  Std.all
##
##     0.343    0.297    0.363
##
##     1.259    0.867    0.159
##    -0.025   -0.073   -0.101
##
##    -0.002   -0.138   -0.063
##
##    -0.223   -0.556   -0.108
##
## Variances:
##                       Estimate  Std.Err  z-value  P(>|z|) ci.lower ci.upper
##    .cr_talk              0.916    0.050   18.187    0.000    0.817    1.015
```

```
##    .cr_copingrslnc    8.873    0.613   14.472    0.000    7.671   10.074
##    .cr_media          1.601    0.074   21.740    0.000    1.457    1.745
##    .dCRET20_clbm_y    0.203    0.005   39.833    0.000    0.193    0.213
##    .cr_gndrnrmsc_1    3.181    0.155   20.533    0.000    2.877    3.484
##    .cr_gndrnrmsc_2    2.960    0.141   20.979    0.000    2.684    3.237
##    .cr_gndrnrmsc_4    2.335    0.125   18.688    0.000    2.090    2.580
##    .cr_gendrdctvts    2.961    0.176   16.815    0.000    2.616    3.306
##    .cr_ghq12         12.664    0.874   14.492    0.000   10.951   14.377
##    .dCRET20_female    0.226    0.004   64.008    0.000    0.219    0.233
##    .Competencies      0.192    0.035    5.538    0.000    0.124    0.261
##    .GenderNorms       0.215    0.030    7.282    0.000    0.157    0.273
##    .GenderAttituds    0.679    0.092    7.376    0.000    0.499    0.860
##    Std.lv  Std.all
##     0.916    0.664
##     8.873    0.590
##     1.601    0.655
##     0.203    0.822
##     3.181    0.646
##     2.960    0.678
##     2.335    0.640
##     2.961    0.648
##    12.664    0.921
##     0.226    0.910
##     0.415    0.415
##     0.255    0.255
##     0.390    0.390
##
## R-Square:
##                    Estimate
##     cr_talk           0.336
##     cr_copingrslnc    0.410
##     cr_media          0.345
##     dCRET20_clbm_y    0.178
##     cr_gndrnrmsc_1    0.354
##     cr_gndrnrmsc_2    0.322
##     cr_gndrnrmsc_4    0.360
##     cr_gendrdctvts    0.352
##     cr_ghq12          0.079
##     dCRET20_female    0.090
##     Competencies      0.585
##     GenderNorms       0.745
##     GenderAttituds    0.610
##
## Defined Parameters:
##                    Estimate  Std.Err  z-value  P(>|z|) ci.lower ci.upper
##     partGNGAMH       -0.080    0.150   -0.534    0.593   -0.374    0.214
##     partGNGRMH        0.140    0.091    1.529    0.126   -0.039    0.319
##     partGNGRCOM      -0.007    0.025   -0.263    0.792   -0.056    0.043
##     partSexGAMH      -0.048    0.091   -0.534    0.594   -0.227    0.130
##     tieGNMH           0.059    0.177    0.336    0.737   -0.288    0.407
##     tieGNCOM         -0.007    0.025   -0.263    0.792   -0.056    0.043
##     tieSexMH         -0.048    0.091   -0.534    0.594   -0.227    0.130
##     teSexMH          -0.540    0.217   -2.492    0.013   -0.964   -0.115
##     teGNMH            0.227    0.231    0.983    0.326   -0.225    0.679
##     teGNCOM           0.624    0.061   10.169    0.000    0.504    0.744
##     med_propSexMH     0.090    0.173    0.519    0.604   -0.250    0.429
```

```
##     med_propGNGAMH   -0.354    0.741   -0.477    0.633   -1.807    1.100
##     med_propGNGRMH    0.616    0.790    0.780    0.435   -0.932    2.164
##     med_prpGNGRCOM   -0.011    0.040   -0.265    0.791   -0.089    0.068
##    Std.lv  Std.all
##    -0.074   -0.020
##     0.128    0.035
##    -0.009   -0.009
##    -0.048   -0.007
##     0.055    0.015
##    -0.009   -0.009
##    -0.048   -0.007
##    -0.540   -0.073
##     0.208    0.056
##     0.842    0.842
##     0.090    0.090
##    -0.354   -0.354
##     0.616    0.616
##    -0.011   -0.011
```

```
fitMeasures(fit.testmPUBPeerSen)
```

```
##                          npar                           fmin
##                        63.000                          0.272
##                         chisq                             df
##                       899.048                        132.000
##                        pvalue                   chisq.scaled
##                         0.000                        754.965
##                     df.scaled                  pvalue.scaled
##                       132.000                          0.000
##          chisq.scaling.factor                 baseline.chisq
##                         1.191                       4623.206
##                   baseline.df                baseline.pvalue
##                       185.000                          0.000
##         baseline.chisq.scaled             baseline.df.scaled
##                      3873.370                        185.000
##        baseline.pvalue.scaled baseline.chisq.scaling.factor
##                         0.000                          1.194
##                           cfi                            tli
##                         0.827                          0.758
##                    cfi.scaled                     tli.scaled
##                         0.831                          0.763
##                    cfi.robust                     tli.robust
##                         0.831                          0.764
##                          nnfi                            rfi
##                         0.758                          0.727
##                           nfi                           pnfi
##                         0.806                          0.575
##                           ifi                            rni
##                         0.829                          0.827
##                   nnfi.scaled                     rfi.scaled
##                         0.763                          0.727
##                    nfi.scaled                    pnfi.scaled
##                         0.805                          0.574
##                    ifi.scaled                     rni.scaled
##                         0.833                          0.831
##                   nnfi.robust                     rni.robust
##                         0.764                          0.831
##                          logl              unrestricted.logl
##                    -29591.526                     -29142.002
##                           aic                            bic
##                     59309.053                      59649.943
##                        ntotal                           bic2
##                      1654.000                      59449.801
##             scaling.factor.h1              scaling.factor.h0
##                         1.196                          1.208
##                         rmsea                 rmsea.ci.lower
##                         0.059                          0.056
##                rmsea.ci.upper                 rmsea.ci.level
##                         0.063                          0.900
##                  rmsea.pvalue                 rmsea.close.h0
##                         0.000                          0.050
##         rmsea.notclose.pvalue              rmsea.notclose.h0
##                         0.000                          0.080
##                  rmsea.scaled          rmsea.ci.lower.scaled
##                         0.053                          0.050
##         rmsea.ci.upper.scaled            rmsea.pvalue.scaled
```

```
##                           0.057                           0.047
##  rmsea.notclose.pvalue.scaled                   rmsea.robust
##                           0.000                           0.058
##         rmsea.ci.lower.robust         rmsea.ci.upper.robust
##                           0.054                           0.062
##           rmsea.pvalue.robust  rmsea.notclose.pvalue.robust
##                           0.000                           0.000
##                             rmr                     rmr_nomean
##                           0.096                           0.096
##                            srmr                   srmr_bentler
##                           0.037                           0.037
##           srmr_bentler_nomean                           crmr
##                           0.037                           0.039
##                   crmr_nomean                    srmr_mplus
##                           0.039                           0.037
##             srmr_mplus_nomean                          cn_05
##                           0.037                         295.013
##                         cn_01                            gfi
##                         318.740                           0.911
##                          agfi                           pgfi
##                           0.798                           0.401
##                           mfi                           ecvi
##                           0.793                           0.620
## attr(,"scaled.test")
## [1] "yuan.bentler.mplus"
```

```
#parameterEstimates(fit.testmPUBPeerSen, standardized = TRUE)
#modindices(fit.testmPUBPeerSen, sort. = TRUE)
```

7.2.2 Descriptive statistics of the sample after peer review

```
#frequencies for model after peer review
sem_ids <- lavInspect(fit.testmNEUPUB, "case.idx")
sempeer_sample1 <- data_ET20_final[sem_ids, ]

frq(sempeer_sample1$cr_ghq12)
```

```
## x <numeric>
## # total N=1588 valid N=1588 mean=7.04 sd=3.84
##
## Value |   N | Raw % | Valid % | Cum. %
## --------------------------------------
##     0 |  49 |  3.09 |    3.09 |   3.09
##     1 |  33 |  2.08 |    2.08 |   5.16
##     2 |  45 |  2.83 |    2.83 |   8.00
##     3 |  68 |  4.28 |    4.28 |  12.28
##     4 |  88 |  5.54 |    5.54 |  17.82
##     5 | 150 |  9.45 |    9.45 |  27.27
##     6 | 465 | 29.28 |   29.28 |  56.55
##     7 | 183 | 11.52 |   11.52 |  68.07
##     8 | 130 |  8.19 |    8.19 |  76.26
##     9 |  87 |  5.48 |    5.48 |  81.74
##    10 |  68 |  4.28 |    4.28 |  86.02
##    11 |  48 |  3.02 |    3.02 |  89.04
##    12 |  39 |  2.46 |    2.46 |  91.50
##    13 |  25 |  1.57 |    1.57 |  93.07
##    14 |  25 |  1.57 |    1.57 |  94.65
##    15 |  22 |  1.39 |    1.39 |  96.03
##    16 |  17 |  1.07 |    1.07 |  97.10
##    17 |  11 |  0.69 |    0.69 |  97.80
##    18 |  10 |  0.63 |    0.63 |  98.43
##    19 |  12 |  0.76 |    0.76 |  99.18
##    20 |   4 |  0.25 |    0.25 |  99.43
##    22 |   3 |  0.19 |    0.19 |  99.62
##    24 |   2 |  0.13 |    0.13 |  99.75
##    25 |   2 |  0.13 |    0.13 |  99.87
##    30 |   1 |  0.06 |    0.06 |  99.94
##    36 |   1 |  0.06 |    0.06 | 100.00
##  <NA> |   0 |  0.00 |    <NA> |   <NA>
```

```
frq(sempeer_sample1$cr_female)
```

```
## x <numeric>
## # total N=1588 valid N=1588 mean=0.53 sd=0.50
##
## Value |   N | Raw % | Valid % | Cum. %
## --------------------------------------
##     0 | 744 | 46.85 |   46.85 |  46.85
##     1 | 844 | 53.15 |   53.15 | 100.00
##  <NA> |   0 |  0.00 |    <NA> |   <NA>
```

```
frq(sempeer_sample1$cr_copingresilience)
```

```
## x <numeric>
## # total N=1588 valid N=1588 mean=20.07 sd=4.01
##
## Value |   N | Raw % | Valid % | Cum. %
## --------------------------------------
##     0 |   3 |  0.19 |    0.19 |   0.19
##     2 |   5 |  0.31 |    0.31 |   0.50
##     3 |   2 |  0.13 |    0.13 |   0.63
##     4 |   6 |  0.38 |    0.38 |   1.01
##     6 |   5 |  0.31 |    0.31 |   1.32
##     7 |   1 |  0.06 |    0.06 |   1.39
##     8 |  10 |  0.63 |    0.63 |   2.02
##     9 |   5 |  0.31 |    0.31 |   2.33
##    10 |  11 |  0.69 |    0.69 |   3.02
##    11 |   9 |  0.57 |    0.57 |   3.59
##    12 |  27 |  1.70 |    1.70 |   5.29
##    13 |  27 |  1.70 |    1.70 |   6.99
##    14 |  31 |  1.95 |    1.95 |   8.94
##    15 |  33 |  2.08 |    2.08 |  11.02
##    16 |  73 |  4.60 |    4.60 |  15.62
##    17 |  77 |  4.85 |    4.85 |  20.47
##    18 | 136 |  8.56 |    8.56 |  29.03
##    19 |  82 |  5.16 |    5.16 |  34.19
##    20 | 181 | 11.40 |   11.40 |  45.59
##    21 | 101 |  6.36 |    6.36 |  51.95
##    22 | 297 | 18.70 |   18.70 |  70.65
##    23 |  92 |  5.79 |    5.79 |  76.45
##    24 | 374 | 23.55 |   23.55 | 100.00
##  <NA> |   0 |  0.00 |    <NA> |   <NA>
```

```
frq(sempeer_sample1$cr_talk)
```

```
## x <numeric>
## # total N=1588 valid N=1588 mean=2.63 sd=1.19
##
## Value |   N | Raw % | Valid % | Cum. %
## --------------------------------------
##     0 | 125 |  7.87 |    7.87 |   7.87
##     1 | 144 |  9.07 |    9.07 |  16.94
##     2 | 331 | 20.84 |   20.84 |  37.78
##     3 | 577 | 36.34 |   36.34 |  74.12
##     4 | 411 | 25.88 |   25.88 | 100.00
##  <NA> |   0 |  0.00 |    <NA> |   <NA>
```

```
frq(sempeer_sample1$cr_voice)
```

```
## x <numeric>
## # total N=1588 valid N=1588 mean=18.66 sd=5.05
##
## Value |   N | Raw % | Valid % | Cum. %
## --------------------------------------
##     8 |  19 |  1.20 |    1.20 |   1.20
##     9 |  19 |  1.20 |    1.20 |   2.39
##    10 |  26 |  1.64 |    1.64 |   4.03
##    11 |  64 |  4.03 |    4.03 |   8.06
##    12 |  63 |  3.97 |    3.97 |  12.03
##    13 |  74 |  4.66 |    4.66 |  16.69
##    14 |  75 |  4.72 |    4.72 |  21.41
##    15 | 106 |  6.68 |    6.68 |  28.09
##    16 | 119 |  7.49 |    7.49 |  35.58
##    17 | 110 |  6.93 |    6.93 |  42.51
##    18 | 132 |  8.31 |    8.31 |  50.82
##    19 | 127 |  8.00 |    8.00 |  58.82
##    20 |  84 |  5.29 |    5.29 |  64.11
##    21 |  99 |  6.23 |    6.23 |  70.34
##    22 |  88 |  5.54 |    5.54 |  75.88
##    23 | 116 |  7.30 |    7.30 |  83.19
##    24 |  56 |  3.53 |    3.53 |  86.71
##    25 |  55 |  3.46 |    3.46 |  90.18
##    26 |  55 |  3.46 |    3.46 |  93.64
##    27 |  28 |  1.76 |    1.76 |  95.40
##    28 |  21 |  1.32 |    1.32 |  96.73
##    29 |  20 |  1.26 |    1.26 |  97.98
##    30 |  11 |  0.69 |    0.69 |  98.68
##    31 |  12 |  0.76 |    0.76 |  99.43
##    32 |   9 |  0.57 |    0.57 | 100.00
##  <NA> |   0 |  0.00 |    <NA> |   <NA>
```

```
frq(sempeer_sample1$cr_opelder)
```

```
## x <numeric>
## # total N=1588 valid N=1588 mean=0.45 sd=0.50
##
## Value |   N | Raw % | Valid % | Cum. %
## --------------------------------------
##     0 | 880 | 55.42 |   55.42 |  55.42
##     1 | 708 | 44.58 |   44.58 | 100.00
##  <NA> |   0 |  0.00 |    <NA> |   <NA>
```

```
frq(sempeer_sample1$cr_opfriend)
```

```
## x <numeric>
## # total N=1588 valid N=1588 mean=0.78 sd=0.41
##
## Value |    N | Raw % | Valid % | Cum. %
## --------------------------------------
##     0 |  348 | 21.91 |   21.91 |  21.91
##     1 | 1240 | 78.09 |   78.09 | 100.00
##  <NA> |    0 |  0.00 |    <NA> |   <NA>
```

```
frq(sempeer_sample1$cr_gendernormscale_f1)
```

```
## x <numeric>
## # total N=1588 valid N=1588 mean=10.17 sd=2.23
##
## Value |   N | Raw % | Valid % | Cum. %
## -------------------------------------
##     4 |  44 |  2.77 |    2.77 |   2.77
##     5 |  18 |  1.13 |    1.13 |   3.90
##     6 |  94 |  5.92 |    5.92 |   9.82
##     7 |  58 |  3.65 |    3.65 |  13.48
##     8 | 177 | 11.15 |   11.15 |  24.62
##     9 |  66 |  4.16 |    4.16 |  28.78
##    10 | 285 | 17.95 |   17.95 |  46.73
##    11 |  99 |  6.23 |    6.23 |  52.96
##    12 | 747 | 47.04 |   47.04 | 100.00
##  <NA> |   0 |  0.00 |    <NA> |   <NA>
```

```
frq(sempeer_sample1$cr_gendernormscale_f2)
```

```
## x <numeric>
## # total N=1588 valid N=1588 mean=6.28 sd=2.10
##
## Value |   N | Raw % | Valid % | Cum. %
## -------------------------------------
##     3 | 249 | 15.68 |   15.68 |  15.68
##     4 |  93 |  5.86 |    5.86 |  21.54
##     5 | 299 | 18.83 |   18.83 |  40.37
##     6 | 140 |  8.82 |    8.82 |  49.18
##     7 | 331 | 20.84 |   20.84 |  70.03
##     8 |  85 |  5.35 |    5.35 |  75.38
##     9 | 391 | 24.62 |   24.62 | 100.00
##  <NA> |   0 |  0.00 |    <NA> |   <NA>
```

```
frq(sempeer_sample1$cr_gendernormscale_f3)
```

```
## x <numeric>
## # total N=1588 valid N=1588 mean=4.92 sd=1.44
##
## Value |   N | Raw % | Valid % | Cum. %
## ------------------------------------
##     2 | 208 | 13.10 |   13.10 |  13.10
##     3 |  63 |  3.97 |    3.97 |  17.07
##     4 | 287 | 18.07 |   18.07 |  35.14
##     5 | 122 |  7.68 |    7.68 |  42.82
##     6 | 908 | 57.18 |   57.18 | 100.00
##  <NA> |   0 |  0.00 |    <NA> |   <NA>
```

```
frq(sempeer_sample1$cr_gendernormscale_f4)
```

```
## x <numeric>
## # total N=1588 valid N=1588 mean=5.37 sd=1.99
##
## Value |   N | Raw % | Valid % | Cum. %
## ------------------------------------
##     3 | 435 | 27.39 |   27.39 |  27.39
##     4 | 115 |  7.24 |    7.24 |  34.63
##     5 | 399 | 25.13 |   25.13 |  59.76
##     6 | 130 |  8.19 |    8.19 |  67.95
##     7 | 252 | 15.87 |   15.87 |  83.82
##     8 |  88 |  5.54 |    5.54 |  89.36
##     9 | 169 | 10.64 |   10.64 | 100.00
##  <NA> |   0 |  0.00 |    <NA> |   <NA>
```

```
frq(sempeer_sample1$cr_gendernormscale_f5)
```

```
## x <numeric>
## # total N=1588 valid N=1587 mean=5.90 sd=1.89
##
## Value |   N | Raw % | Valid % | Cum. %
## ------------------------------------
##     4 | 561 | 35.33 |   35.35 |  35.35
##     5 | 151 |  9.51 |    9.51 |  44.86
##     6 | 390 | 24.56 |   24.57 |  69.44
##     7 | 131 |  8.25 |    8.25 |  77.69
##     8 | 208 | 13.10 |   13.11 |  90.80
##     9 |  55 |  3.46 |    3.47 |  94.27
##    10 |  65 |  4.09 |    4.10 |  98.36
##    11 |  14 |  0.88 |    0.88 |  99.24
##    12 |  12 |  0.76 |    0.76 | 100.00
##  <NA> |   1 |  0.06 |    <NA> |   <NA>
```

```
frq(sempeer_sample1$cr_gendernormscale_f6)
```

```
## x <numeric>
## # total N=1588 valid N=1583 mean=3.20 sd=1.32
##
## Value |   N | Raw % | Valid % | Cum. %
## ------------------------------------
##     2 | 735 | 46.28 |   46.43 |  46.43
##     3 | 182 | 11.46 |   11.50 |  57.93
##     4 | 430 | 27.08 |   27.16 |  85.09
##     5 |  93 |  5.86 |    5.87 |  90.97
##     6 | 143 |  9.01 |    9.03 | 100.00
##  <NA> |   5 |  0.31 |    <NA> |   <NA>
```

```
frq(sempeer_sample1$cr_gendernormscale_f7)
```

```
## x <numeric>
## # total N=1588 valid N=1585 mean=5.45 sd=0.99
##
## Value |    N | Raw % | Valid % | Cum. %
## -------------------------------------
##     2 |   39 |  2.46 |    2.46 |   2.46
##     3 |   33 |  2.08 |    2.08 |   4.54
##     4 |  240 | 15.11 |   15.14 |  19.68
##     5 |  137 |  8.63 |    8.64 |  28.33
##     6 | 1136 | 71.54 |   71.67 | 100.00
##  <NA> |    3 |  0.19 |    <NA> |   <NA>
```

```
frq(sempeer_sample1$cr_gendernormscale_overall)
```

```
## x <numeric>
## # total N=1588 valid N=1579 mean=41.31 sd=6.74
##
## Value |   N | Raw % | Valid % | Cum. %
## -------------------------------------
##    20 |   1 |  0.06 |    0.06 |   0.06
##    22 |   1 |  0.06 |    0.06 |   0.13
##    24 |  11 |  0.69 |    0.70 |   0.82
##    25 |   5 |  0.31 |    0.32 |   1.14
##    26 |  12 |  0.76 |    0.76 |   1.90
##    27 |   7 |  0.44 |    0.44 |   2.34
##    28 |  17 |  1.07 |    1.08 |   3.42
##    29 |   7 |  0.44 |    0.44 |   3.86
##    30 |  37 |  2.33 |    2.34 |   6.21
##    31 |  25 |  1.57 |    1.58 |   7.79
##    32 |  44 |  2.77 |    2.79 |  10.58
##    33 |  27 |  1.70 |    1.71 |  12.29
##    34 |  64 |  4.03 |    4.05 |  16.34
##    35 |  43 |  2.71 |    2.72 |  19.06
##    36 | 109 |  6.86 |    6.90 |  25.97
##    37 |  37 |  2.33 |    2.34 |  28.31
##    38 | 106 |  6.68 |    6.71 |  35.02
##    39 |  58 |  3.65 |    3.67 |  38.70
##    40 | 122 |  7.68 |    7.73 |  46.42
##    41 |  52 |  3.27 |    3.29 |  49.72
##    42 |  92 |  5.79 |    5.83 |  55.54
##    43 |  64 |  4.03 |    4.05 |  59.59
##    44 | 126 |  7.93 |    7.98 |  67.57
##    45 |  55 |  3.46 |    3.48 |  71.06
##    46 |  77 |  4.85 |    4.88 |  75.93
##    47 |  63 |  3.97 |    3.99 |  79.92
##    48 |  80 |  5.04 |    5.07 |  84.99
##    49 |  35 |  2.20 |    2.22 |  87.21
##    50 |  59 |  3.72 |    3.74 |  90.94
##    51 |  41 |  2.58 |    2.60 |  93.54
##    52 |  46 |  2.90 |    2.91 |  96.45
##    53 |  19 |  1.20 |    1.20 |  97.66
##    54 |  22 |  1.39 |    1.39 |  99.05
##    55 |   7 |  0.44 |    0.44 |  99.49
##    56 |   4 |  0.25 |    0.25 |  99.75
##    57 |   1 |  0.06 |    0.06 |  99.81
##    58 |   3 |  0.19 |    0.19 | 100.00
##  <NA> |   9 |  0.57 |    <NA> |   <NA>
```

```
frq(sempeer_sample1$cr_genderedactivities)
```

```
## Care for others (such as other children, elderly, sick, or disabled household me (x) <numeric>
## # total N=1588 valid N=1588 mean=1.96 sd=2.16
##
## Value |   N | Raw % | Valid % | Cum. %
## -------------------------------------
##     0 | 637 | 40.11 |   40.11 |  40.11
##     1 | 135 |  8.50 |    8.50 |  48.61
##     2 | 268 | 16.88 |   16.88 |  65.49
##     3 | 192 | 12.09 |   12.09 |  77.58
##     4 | 149 |  9.38 |    9.38 |  86.96
##     5 |  94 |  5.92 |    5.92 |  92.88
##     6 |  49 |  3.09 |    3.09 |  95.97
##     7 |  28 |  1.76 |    1.76 |  97.73
##     8 |  22 |  1.39 |    1.39 |  99.12
##     9 |   9 |  0.57 |    0.57 |  99.69
##    10 |   4 |  0.25 |    0.25 |  99.94
##    13 |   1 |  0.06 |    0.06 | 100.00
##  <NA> |   0 |  0.00 |    <NA> |   <NA>
```

```
frq(sempeer_sample1$cr_rolemodel)
```

```
## x <numeric>
## # total N=1588 valid N=1588 mean=0.55 sd=0.50
##
## Value |   N | Raw % | Valid % | Cum. %
## -------------------------------------
##     0 | 722 | 45.47 |   45.47 |  45.47
##     1 | 866 | 54.53 |   54.53 | 100.00
##  <NA> |   0 |  0.00 |    <NA> |   <NA>
```

```
frq(sempeer_sample1$cr_clubmember)
```

```
## x <numeric>
## # total N=1588 valid N=1588 mean=0.46 sd=0.50
##
## Value |   N | Raw % | Valid % | Cum. %
## -------------------------------------
##     0 | 857 | 53.97 |   53.97 |  53.97
##     1 | 731 | 46.03 |   46.03 | 100.00
##  <NA> |   0 |  0.00 |    <NA> |   <NA>
```

```
frq(sempeer_sample1$cr_socialmedia)
```

```
## x <numeric>
## # total N=1588 valid N=1584 mean=1.26 sd=0.75
##
## Value |    N | Raw % | Valid % | Cum. %
## --------------------------------------
##     1 | 1371 | 86.34 |   86.55 |  86.55
##     2 |   81 |  5.10 |    5.11 |  91.67
##     3 |   92 |  5.79 |    5.81 |  97.47
##     4 |   21 |  1.32 |    1.33 |  98.80
##     5 |   12 |  0.76 |    0.76 |  99.56
##     6 |    7 |  0.44 |    0.44 | 100.00
##  <NA> |    4 |  0.25 |    <NA> |   <NA>
```

```
frq(sempeer_sample1$cr_media)
```

```
## x <numeric>
## # total N=1588 valid N=1588 mean=2.66 sd=1.65
##
## Value |   N | Raw % | Valid % | Cum. %
## -------------------------------------
##     1 | 624 | 39.29 |   39.29 |  39.29
##     2 | 206 | 12.97 |   12.97 |  52.27
##     3 | 210 | 13.22 |   13.22 |  65.49
##     4 | 274 | 17.25 |   17.25 |  82.75
##     5 | 184 | 11.59 |   11.59 |  94.33
##     6 |  90 |  5.67 |    5.67 | 100.00
##  <NA> |   0 |  0.00 |    <NA> |   <NA>
```

```
frq(sempeer_sample1$peervio_ordinal)
```

```
## x <numeric>
## # total N=1588 valid N=1588 mean=0.93 sd=1.78
##
## Value |    N | Raw % | Valid % | Cum. %
## --------------------------------------
##     0 | 1058 | 66.62 |   66.62 |  66.62
##     1 |  169 | 10.64 |   10.64 |  77.27
##     2 |  148 |  9.32 |    9.32 |  86.59
##     3 |   64 |  4.03 |    4.03 |  90.62
##     4 |   67 |  4.22 |    4.22 |  94.84
##     5 |   22 |  1.39 |    1.39 |  96.22
##     6 |   28 |  1.76 |    1.76 |  97.98
##     7 |   15 |  0.94 |    0.94 |  98.93
##     8 |    6 |  0.38 |    0.38 |  99.31
##     9 |    1 |  0.06 |    0.06 |  99.37
##    10 |    3 |  0.19 |    0.19 |  99.56
##    11 |    2 |  0.13 |    0.13 |  99.69
##    12 |    5 |  0.31 |    0.31 | 100.00
##  <NA> |    0 |  0.00 |    <NA> |   <NA>
```

```
frq(sempeer_sample1$cr_urban)
```

```
## x <numeric>
## # total N=1588 valid N=1588 mean=0.39 sd=0.49
##
## Value |   N | Raw % | Valid % | Cum. %
## --------------------------------------
##     0 | 975 | 61.40 |   61.40 |  61.40
##     1 | 613 | 38.60 |   38.60 | 100.00
##  <NA> |   0 |  0.00 |    <NA> |   <NA>
```

```
frq(sempeer_sample1$purpsamp)
```

```
## x <numeric>
## # total N=1588 valid N=1588 mean=0.07 sd=0.26
##
## Value |    N | Raw % | Valid % | Cum. %
## ---------------------------------------
##     0 | 1472 | 92.70 |   92.70 |  92.70
##     1 |  116 |  7.30 |    7.30 | 100.00
##  <NA> |    0 |  0.00 |    <NA> |   <NA>
```

```
frq(sempeer_sample1$sample_r2_disability2)
```

```
## x <numeric>
## # total N=1588 valid N=1588 mean=0.07 sd=0.26
##
## Value |    N | Raw % | Valid % | Cum. %
## ---------------------------------------
##     0 | 1473 | 92.76 |   92.76 |  92.76
##     1 |  115 |  7.24 |    7.24 | 100.00
##  <NA> |    0 |  0.00 |    <NA> |   <NA>
```

```
frq(sempeer_sample1$af_dw_lang_recode)
```

```
## What is the primary language spoken in this household? (x) <numeric>
## # total N=1588 valid N=1588 mean=203.02 sd=1.65
##
## Value |   N | Raw % | Valid % | Cum. %
## --------------------------------------
##   201 | 407 | 25.63 |   25.63 |  25.63
##   202 | 253 | 15.93 |   15.93 |  41.56
##   204 | 904 | 56.93 |   56.93 |  98.49
##   211 |  24 |  1.51 |    1.51 | 100.00
##  <NA> |   0 |  0.00 |    <NA> |   <NA>
```

```
frq(sempeer_sample1$af_asset_index)
```

```
## How many mattresses and/or bed(s) does the household own? (x) <numeric>
## # total N=1588 valid N=1588 mean=6.15 sd=5.11
##
## Value |   N | Raw % | Valid % | Cum. %
## ------------------------------------
##     0 |  35 |  2.20 |    2.20 |   2.20
##     1 | 136 |  8.56 |    8.56 |  10.77
##     2 | 200 | 12.59 |   12.59 |  23.36
##     3 | 202 | 12.72 |   12.72 |  36.08
##     4 | 191 | 12.03 |   12.03 |  48.11
##     5 | 137 |  8.63 |    8.63 |  56.74
##     6 | 117 |  7.37 |    7.37 |  64.11
##     7 |  90 |  5.67 |    5.67 |  69.77
##     8 |  99 |  6.23 |    6.23 |  76.01
##     9 |  78 |  4.91 |    4.91 |  80.92
##    10 |  69 |  4.35 |    4.35 |  85.26
##    11 |  37 |  2.33 |    2.33 |  87.59
##    12 |  38 |  2.39 |    2.39 |  89.99
##    13 |  31 |  1.95 |    1.95 |  91.94
##    14 |  25 |  1.57 |    1.57 |  93.51
##    15 |  23 |  1.45 |    1.45 |  94.96
##    16 |  13 |  0.82 |    0.82 |  95.78
##    17 |  12 |  0.76 |    0.76 |  96.54
##    18 |  10 |  0.63 |    0.63 |  97.17
##    19 |   5 |  0.31 |    0.31 |  97.48
##    20 |   6 |  0.38 |    0.38 |  97.86
##    21 |   8 |  0.50 |    0.50 |  98.36
##    22 |   7 |  0.44 |    0.44 |  98.80
##    23 |   3 |  0.19 |    0.19 |  98.99
##    24 |   2 |  0.13 |    0.13 |  99.12
##    25 |   2 |  0.13 |    0.13 |  99.24
##    26 |   1 |  0.06 |    0.06 |  99.31
##    28 |   1 |  0.06 |    0.06 |  99.37
##    29 |   4 |  0.25 |    0.25 |  99.62
##    31 |   1 |  0.06 |    0.06 |  99.69
##    34 |   1 |  0.06 |    0.06 |  99.75
##    35 |   1 |  0.06 |    0.06 |  99.81
##    39 |   1 |  0.06 |    0.06 |  99.87
##    40 |   1 |  0.06 |    0.06 |  99.94
##    48 |   1 |  0.06 |    0.06 | 100.00
##  <NA> |   0 |  0.00 |    <NA> |   <NA>
```

```
frq(sempeer_sample1$cr_aspiratt_higheredu)
```

```
## x <numeric>
## # total N=1588 valid N=1588 mean=2.30 sd=1.04
##
## Value |    N | Raw % | Valid % | Cum. %
## -------------------------------------
##     0 |  115 |  7.24 |    7.24 |   7.24
##     1 |  351 | 22.10 |   22.10 |  29.35
##     2 |   72 |  4.53 |    4.53 |  33.88
##     3 | 1050 | 66.12 |   66.12 | 100.00
##  <NA> |    0 |  0.00 |    <NA> |   <NA>
```

```
frq(sempeer_sample1$cr_workfambus)
```

```
## x <numeric>
## # total N=1588 valid N=1588 mean=0.04 sd=0.20
##
## Value |    N | Raw % | Valid % | Cum. %
## ---------------------------------------
##     0 | 1521 | 95.78 |   95.78 |  95.78
##     1 |   67 |  4.22 |    4.22 | 100.00
##  <NA> |    0 |  0.00 |    <NA> |   <NA>
```

```
frq(sempeer_sample1$af_fb_biofhh)
```

```
## x <numeric>
## # total N=1588 valid N=1588 mean=1.26 sd=0.44
##
## Value |    N | Raw % | Valid % | Cum. %
## ---------------------------------------
##     1 | 1169 | 73.61 |   73.61 |  73.61
##     2 |  419 | 26.39 |   26.39 | 100.00
##  <NA> |    0 |  0.00 |    <NA> |   <NA>
```
