# Supplementary material for: Gendered pathways to adolescent mental health: An empirical assessment of a new conceptual framework
Source: PLOS Glob Public Health. 2026 Nov 10;6(11):e0006728. doi: 10.1371/journal.pgph.0006728 (PMC13750542; doi:10.1371/journal.pgph.0006728)
Supplement: Supplementary file 2 [file pgph.0006728.s001.docx]

# S1 Appendix: Recoding of the measures used in the analysis

We operationalised the community level of the **gender norms construct** with a *peer violence index*, *being a club member*, and *having a role model,* and the digital level with *media* and *social media use*. The GAGE *peer violence index* includes six items that ask how many times in the past twelve months have any peers (in person or on the internet): *used words to hurt you; left you out of their games or activities, or ignored you; stole or damaged something of yours; physically hurt you; made you do things that you didn’t want to do; or threatened you or someone close to you with harm*. Each item is a three-level variable with (1) *never*, (2) *once*, and (3) *more than once*, which we recoded from (0) *never* to (2) *more than once* so that a higher sum score indicates more experienced peer violence. For the variable *being a club member*, we collapsed the following five items: ‘Are you currently a member of a *girl’s/boy’s club*, *a co-ed youth club*, *a sports club*, *a religious club*, or *another club*?’ into the binary variable with (0) *not being a member of any club* and (1) *being a member of a club*. For the variable *having a role model*, we used the item that asks whether there is a person that the adolescent respects, follows, looks up to, or wants to be like with (0) *not having a role model* and (1) *having a role model*. For the *media* variable, we used four items that ask how often the adolescent (in the last 30 days): *read a newspaper or magazine; listened to the radio; watched television; and looked for news online*. We used another five items asking how often the adolescent *used a computer, laptop or tablet; played online games; visited a social network; used instant messaging; and watched video clips* for the *social media* variable. Some items were scored on a seven-point, others on a six-point Likert scale. We collapsed the respective items into a six-level Likert scale, with (1) *never* to (6) *almost all the time* for both the media and the social media variable. For those items with seven levels, we combined levels (2) *hardly ever* and (3) *at least every month*, with the latter being the missing response option in the six-level variables. All variables operationalising the gender norms construct were recoded so that higher values indicate higher exposure to gender norms.

We operationalised **gender attitudes** with the GAGE gender norms scale (conceptually measuring gender attitudes). Each item is scored on a three-level scale with (1) indicating *agreement*, (2) indicating *partial agreement* and (3) indicating *disagreement* with the statement. We recoded all items with a reverse question to ensure that agreement suggests a gender-unequal response. Thereafter, we explored the factor structure of the gender attitudes scale using Exploratory and Confirmatory Factor Analysis, which suggested retaining 20 of the scale’s original 32 items as a latent variable with seven sum scores. A higher score indicates more gender egalitarian attitudes.

We operationalised the **gender roles construct** with a *gendered activities score*. The score includes two items, which are measured in hours per typical school day: *time spent on care for others*; and *time spent on domestic chores for the household*. We recoded the items as a sum score so that a higher score translates to more hours spent on the activities per day, indicating adherence to more traditional gender roles.

We operationalised the construct **competencies** with six different instruments: a *voice* index; a r*esilience* index; an *index for talking to caregivers* about work, religion, education, and bullying; *speaking up in class*, *expression of opinion to friends* and *expression of opinion to elders*. We generated the variable *voice* with the six items that capture how much say the adolescent thinks s/he has on the following issues in the family: *how much time the adolescent spends helping around the house*; *how much education the adolescent will get*; *when to marry*; *who to marry*; *who the adolescent wants to be friends with*; *what the adolescent does in the free time*; *whether the adolescent can leave the house*; and *whether the adolescent can be involved in income-generating activities*. Each item is coded on a four-point Likert scale ranging from (1) *not at all* to (4) *a great deal*. We recoded the items as a sum score so that a higher score indicates a higher perception of say in the issues. The resilience index is based on the items of the child and youth resilience measure – 12 (CYRM-12) (39) and was slightly adapted by GAGE. The twelve questions ask to what extent the following statements describe the adolescent: *I have people I want to be like*; *getting an education is important to me*; *I feel like my parents know a lot about me*; *I try to finish activities that I start*; *when things don’t go my way, I can fix it without hurting myself or other people*; *I know where to go to get help*; *I feel that I belong at my school*; *I think my family cares about me when times are hard*; *I think my friends care about me when times are hard*; *I am treated fairly*; *I have changes to learn things that will be useful when I am older; I like the way my community celebrates things*. Each item is a three-level variable with (0) indicating *disagreement*, (1) indicating *partial agreement*, and (2) indicating *agreement* with the statements. We recoded the items as a sum score (0-24) with higher scores indicating higher resilience. The index for talking to parents or caregivers about work, religion, education, and bullying is based on the Power to Girls SASA! Evaluation Baseline Survey items (40), which ask four questions separately for fathers and mothers: *have you ever talked about what you want to do for work in the future with your father/mother?*; *have you ever talked about religion with your father/ mother?*; *have you ever talked about your education with your father/mother?*; and *have you ever talked about bullying/harassment at school with your father/mother?*. Each item is coded binary with (0) indicating *disagreement* and (1) indicating *agreement* with the question. We collapsed these items into a four-level sum score (0-4) with a higher score indicating that the adolescent discusses more topics with their parents. Finally, we used the item asking whether the adolescent feels comfortable expressing an opinion to or disagreeing with people in their age group, such as siblings and friends for the variable *expression of opinion to friends*, and the item asking whether the adolescent feels comfortable expressing an opinion to or disagreeing with people who are much older than them, such as parents and the elderly for the variable *expression of opinion to elderly*. Both variables are binary with (0) *not feeling comfortable* and (1) *feeling comfortable*.

We operationalised the **mental health** construct with the validated instrument GHQ-12, which contains the following twelve items: *able to concentrate*; *loss of sleep over worry*; *playing a useful part*; *capable of making decisions*; *felt constantly under strain*; *could not overcome difficulties*; *able to enjoy day-to-day activities*; *able to face problems*; *feeling unhappy and depressed*; *losing confidence*; *thinking of self as worthless*; *feeling reasonably happy (22)*. Each item is scored on a four-point Likert scale ranging from (0) *not at all* to (3) *much more than usual*. We recoded the items as a sum score (0-36) so that a higher score translates to increased psychological distress and thus worse mental health.
