# Supplementary material for: Gendered pathways to adolescent mental health: An empirical assessment of a new conceptual framework
Source: PLOS Glob Public Health. 2026 Nov 10;6(11):e0006728. doi: 10.1371/journal.pgph.0006728 (PMC13750542; doi:10.1371/journal.pgph.0006728)
Supplement: Supplementary file 3 [file pgph.0006728.s002.docx]

# S2 Appendix: Validation of the GAGE gender norms scale for operationalising the gender attitudes construct using EFA and CFA

### Methods

To validate the GAGE gender norms scale (conceptually measuring gender attitudes) for Ethiopian adolescents, we used Exploratory Factor Analysis (EFA) and Confirmatory Factor Analysis (CFA). First, we used parallel analysis with a 95% significance to determine how many factors to extract in the EFA. In the EFA, we used Principal Axis Factoring and the oblique oblimin rotation method, which is suitable for the correlated gender attitudes items. We used Bartlett’s test of sphericity and the Kaiser-Meyer-Olkin (KMO) test of sampling adequacy to assess the suitability of the datasets for EFA. Second, we excluded variables that did not load onto any factor or exhibited cross-loadings. We repeated this procedure until all remaining variables loaded onto one factor. Thereafter, we applied cutoffs and removed variables with communalities below 0.2 and factor loadings below 0.3 iteratively until all remaining variables loaded onto a factor.

In the CFA, we included the reduced set of variables based on the results of the EFA. We then calculated a one-factor model using the Robust Maximum Likelihood estimation (MLR). We then estimated a seven-factor model as proposed in the EFA and allowed the factors to correlate (=oblimin rotation). We excluded factor loadings below 0.4. We used the following fit indices and thresholds to assess model fit: the Chi-squared test statistics, the Bentler Comparative Fit Index (CFI) ≥ 0.95, the Tucker-Lewis Index (TLI) ≥ 0.90; the Root Mean Square Error of Approximation (RMSEA) <0.06, and the Standard Root Mean Square Residual (SMSR) <0.08 (1, 2).

### Results

Bartlett’s test of sphericity yielded values of χ²(496) = 22272.42, p<0.001 and the KMO was 0.88 overall, with the lowest individual value of 0.78. This indicates sufficiently large inter-item correlations and an adequate sample for EFA, respectively. Parallel analysis suggested extracting eight factors for the initial set of variables. To refine this factor solution, we removed six variables that did not load onto any factor (cr_srh_waitedu, cr_srh_changereaction, cr_fi_goodwife, cr_edu_girlsprotect, cr_edu_boysweak, cr_srh_boyfinal). This only slightly reduced the KMO from 0.88 to 0.86. For this and the following sets of variables, parallel analysis suggested extracting seven factors. We iteratively removed another three variables (cr_srh_aware, cr_srh_controldaugt, cr_edu_culture) and one variable (cr_srh_genderroles), resulting in a KMO of 0.85 and all variables loading onto a factor. In the last step, we excluded two more variables (cr_edu_girlshumble, cr_ta_eesharetasks) due to low communalities and/or factor loadings. The final set of 20 out of 32 variables is shown in Table A.

The seven-factor solution shows that variables from the same domain (education, financial and economic empowerment, violence, time use, sexual and reproductive health) predominantly group together. Factors one, five and six exclusively contain *education* attitudes. Factor two contains *violence* attitudes, factor three *sexual and reproductive health* attitudes, and factor seven *financial and economic empowerment* attitudes. Only factor four contains two *time-use* variables and one *education* variable. The eleven education variables are thus distributed onto four factors. Most variables load strongly onto the factors (threshold 0.5). The cumulative proportion of variance explained with 37% is rather low compared to a desired minimum of 50%. Communalities do not yield very low values with the lowest value of 0.22 but also no high values with the highest value of 0.58.

Table A: Factor Loadings and Communality for the seven-factor solution from Exploratory Factor Analysis

| Question content | F1 | F2 | F3 | F4 | F5 | F6 | F7 | Com |
| --- | --- | --- | --- | --- | --- | --- | --- | --- |
| If a family can afford for one child to go to secondary school it should be the boy only. | 0.64 | 0.02 | -0.06 | 0.07 | 0.01 | 0.02 | 0.02 | 0.46 |
| Only boys should learn about science, technology, and math. | 0.68 | 0.02 | 0.00 | -0.08 | 0.01 | -0.01 | -0.01 | 0.43 |
| Girls should be sent to school only if they are not needed to help at home. | 0.60 | -0.02 | 0.04 | 0.09 | 0.00 | -0.03 | 0.04 | 0.44 |
| Girls should avoid raising their voice to be ladylike. | 0.09 | 0.07 | 0.08 | 0.44 | 0.04 | 0.09 | 0.05 | 0.38 |
| Boys should be able to show their feelings without fear of being teased. | 0.00 | -0.03 | -0.02 | -0.13 | 0.49 | 0.02 | -0.09 | 0.28 |
| Boys should be raised tough so they can overcome any difficulty in life. | 0.02 | -0.03 | -0.01 | 0.06 | 0.51 | -0.02 | 0.03 | 0.25 |
| Boys should always defend themselves even if it means fighting. | -0.01 | 0.10 | 0.05 | 0.00 | 0.41 | 0.07 | 0.02 | 0.23 |
| It's important for boys to show they are tough even if they are nervous inside. | -0.03 | 0.05 | 0.02 | 0.10 | 0.40 | 0.03 | 0.01 | 0.22 |
| When teachers punish students, the learning environment in school is better. | 0.00 | -0.02 | -0.01 | 0.10 | 0.02 | 0.55 | 0.02 | 0.34 |
| When teachers don't punish students, students behave worse in school. | 0.00 | 0.00 | 0.00 | -0.09 | 0.00 | 0.53 | -0.03 | 0.27 |
| It is appropriate for parents to take boys out of school for work. | 0.52 | -0.01 | 0.09 | -0.03 | -0.03 | 0.03 | -0.03 | 0.30 |
| A woman’s most important role is to take care of her home and cook for her family. | 0.06 | 0.04 | 0.02 | 0.60 | 0.01 | 0.02 | 0.00 | 0.45 |
| A man should have the final word on decisions in his home. | 0.04 | 0.19 | 0.01 | 0.41 | 0.03 | 0.07 | -0.01 | 0.37 |
| Women should have the same chance to work outside of the home as men. | -0.01 | 0.00 | -0.02 | 0.11 | 0.04 | -0.04 | 0.54 | 0.32 |
| It is important for women and adolescent girls to have their own savings. | 0.04 | 0.01 | 0.02 | -0.12 | -0.07 | 0.03 | 0.56 | 0.34 |
| It is okay to tease a girl who acts like a boy. | 0.01 | 0.00 | 0.76 | -0.01 | 0.00 | 0.00 | -0.01 | 0.58 |
| It is okay to tease a boy who acts like a girl. | -0.01 | 0.01 | 0.75 | 0.02 | 0.00 | 0.00 | 0.01 | 0.57 |
| It is acceptable for a man to hit or beat his wife in order to mold or control her behavior. | -0.02 | 0.62 | 0.04 | 0.04 | 0.0 | 0.01 | 0.01 | 0.42 |
| A man using violence against his wife is a private matter that should not be discussed outside the couple. | 0.04 | 0.62 | 0.01 | -0.09 | 0.01 | -0.02 | 0.01 | 0.36 |
| A woman should tolerate violence to keep her family together. | 0.02 | 0.51 | -0.03 | 0.15 | 0.00 | 0.02 | -0.04 | 0.37 |
| Cumulative variance explained | 8% | 14% | 20% | 26% | 30% | 34% | 37% |  |

Green highlighted factor loadings = above 0.5 (strong loading)
Yellow highlighted factor loadings = above 0.3 (weak loading)
Com = Communality
F1 to F7 = seven extracted factors

In summary, the seven-factor solution is solid in terms of strong factor loadings and few very low communalities. However, the solution is not ideal due to not very high communalities, a rather low proportion of cumulative variance, and factors which only consist of two items. Out of the original 32 items, a final set of 20 variables remains.

In the CFA, we tested the final set of 20 items identified in the EFA, as shown in the initial model in Table B. We analysed these items in two versions: i) as a single-factor model and ii) as a seven-factor model. We tested the former to create a sum score which could serve as an observed variable for the gender attitudes construct in the SEM model. The objective of the latter was to create seven separate sum scores (per factor) which might then serve as seven indicator variables for the latent construct of gender attitudes.

In the first version, we included the 20 items in a one-factor model (initial model in the left column), which we revised by removing factor loadings below 0.4. This resulted in 12 out of the 20 gender attitudes items (revised model in the centre). The revised model suggests an improved but not yet ideal model fit compared to the initial model. In the second version, we included the 20 items per factor and allowed the seven factors to correlate (=oblimin rotation). This resulted in good fit statistics and no variable yielded factor loadings below 0.4.

Table B: Factor Loadings and Model Fit Statistics from three Confirmatory Factor Analysis Models

| Factor Structure | 1^st^ version:  Initial model with 20 items based on EFA | Revised model based on factor loadings | 2^nd^ version:  Initial model with 20 items based on EFA |
| --- | --- | --- | --- |
| Structure | 20 items combined in one factor | Remaining 12 items combined in one factor | Items per factor (with correlations between factors) |
| Fit Statistics |  |  |  |
| Chi squared | 5313.513, p<0.001 | 2640.719, p<0.001 | 746.123, p<0.001 |
| df | 170 | 66 | 149 |
| CFI | 0.65 | 0.76 | 0.96 |
| TLI | 0.60 | 0.71 | 0.95 |
| RMSEA | 0.08 | 0.1 | 0.03 |
| SRMR | 0.07 | 0.07 | 0.03 |
| Factor loadings (standardised) |  |  |  |
| If a family can afford for one child to go to secondary school it should be the boy only | 0.591 | 0.603 | 0.670 |
| Only boys should learn about science, technology, and math | 0.525 | 0.543 | 0.629 |
| Girls should be sent to school only if they are not needed to help at home | 0.576 | 0.595 | 0.675 |
| Girls should avoid raising their voice to be lady like | 0.562 | 0.542 | 0.629 |
| Boys should be able to show their feelings without fear of being teased | -0.007 |  | 0.415 |
| Boys should be raised tough so they can overcome any difficulty in life | 0.166 |  | 0.478 |
| Boys should always defend themselves even if it means fighting | 0.241 |  | 0.498 |
| It's important for boys to show they are tough even if they are nervous inside | 0.222 |  | 0.481 |
| When teachers punish students, the learning environment in school is better | 0.276 |  | 0.667 |
| When teachers don't punish students, students behave worse in school | 0.149 |  | 0.431 |
| It is appropriate for parents to take boys out of school for work | 0.457 | 0.474 | 0.536 |
| A woman’s most important role is to take care of her home and cook for her family | 0.532 | 0.515 | 0.619 |
| A man should have the final word on decisions in his home | 0.532 | 0.512 | 0.618 |
| Women should have the same chance to work outside of the home as men | 0.204 |  | 0.529 |
| It is important for women and adolescent girls to have their own savings | 0.110 |  | 0.573 |
| It is okay to tease a girl who acts like a boy | 0.401 | 0.414 | 0.753 |
| It is okay to tease a boy who acts like a girl | 0.414 | 0.427 | 0.768 |
| It is acceptable for a man to hit or beat his wife in order to mold or control her behavior | 0.528 | 0.523 | 0.642 |
| A man using violence against his wife is a private matter that should not be discussed outside the couple | 0.471 | 0.471 | 0.563 |
| A woman should tolerate violence to keep her family together | 0.508 | 0.497 | 0.620 |

The second version yielded the best model fit and factor loadings in the CFA. This indicates that it may be more appropriate to include the gender attitudes construct as a latent variable with seven sum scores as indicator variables.

# References

1. Goretzko D, Siemund K, Sterner P. Evaluating Model Fit of Measurement Models in Confirmatory Factor Analysis. Educational and Psychological Measurement 2024; 84(1):123–44. Available from: URL: https://pmc.ncbi.nlm.nih.gov/articles/PMC10795573/.

2. Hu L, Bentler PM. Cutoff criteria for fit indexes in covariance structure analysis: Conventional criteria versus new alternatives. Structural Equation Modeling: A Multidisciplinary Journal 1999; 6(1):1–55.
