# Supplementary material for: Gendered pathways to adolescent mental health: An empirical assessment of a new conceptual framework
Source: PLOS Glob Public Health. 2026 Nov 10;6(11):e0006728. doi: 10.1371/journal.pgph.0006728 (PMC13750542; doi:10.1371/journal.pgph.0006728)
Supplement: Supplementary file 4 [file pgph.0006728.s003.docx]

## S3 Appendix: Sensitivity Analysis of the Gender Norms Construct

Table A: Results of the same SEM model as in the manuscript but without the variables club member and role model (N=1,654)

| **Model Fit Statistics** | | | |
| --- | --- | --- | --- |
| **Fit Statistics** | **Value** |  |  |
| Chi-Squared | 1088.743, p<0.001 |  |  |
| Df | 111 |  |  |
| CFI | 0.76 |  |  |
| TLI | 0.65 |  |  |
| GFI | 0.88 |  |  |
| AGFI | 0.71 |  |  |
| RMSEA | 0.073 |  |  |
| SRMR | 0.056 |  |  |
| **Confirmatory Factor Analysis** | | | |
| **Latent Construct** | **SFL** | **FL (SE)** |  |
| **Competencies** | | | |
| Index talking to parents | 0.52 | 1.0 (-) |  |
| Resilience Score | 0.72 | 4.53 (0.56) |  |
| **Gender Norms** | | | |
| Media use | 1.0 | 1.0 (-) |  |
| **Gender Attitudes** | | | |
| Gender Attitudes Subscale F1 | 0.57 | 1.0 (-) |  |
| Gender Attitudes Subscale F2 | 0.61 | 1.01 (0.07) |  |
| Gender Attitudes Subscale F4 | 0.61 | 0.92 (0.07) |  |
| **Regression Analysis** | | | |
| **Variable** | **ß (SE)** | **95 % CI** | **p-value** |
| **Outcome: Mental Health** | | | |
| **Gender Attitudes** | -0.21 (0.12) | [-0.44; 0.21] | p=0.075 |
| **Sex** |  |  |  |
| *Ref.: Male* |  |  |  |
| Female | -0.43 (0.23) | [-0.87; 0.02] | p=0.058 |
| **Gender Roles** | **-0.15** (0.07) | [-0.29; -0.004] | p=0.044 |
| **Gender Norms** | -0.01 (0.08) | [-0.17; 0.15] | p=0.907 |
| **Competencies** | **-1.73** (0.31) | [-2.34; -1.13] | p<0.001 |
| **Outcome: Competencies** |  |  |  |
| **Gender Roles** | **-0.08** (0.02) | [-0.12; -0.05] | p<0.001 |
| **Gender Norms** | **0.05** (0.02) | [0.01; 0.10] | P=0.015 |
| **Outcome: Gender Attitudes** |  |  |  |
| **Gender Norms** | **0.24** (0.03) | [0.18; 0.29] | p<0.001 |
| **Sex** |  |  |  |
| *Ref.: Male* |  |  |  |
| Female | **0.32** (0.08) | [0.15; 0.48] | p<0.001 |
| **Outcome: Gender Roles** | | | |
| **Gender Norms** | **-1.10** (0.07) | [-1.24; -0.97] | p<0.001 |

Ref. = Reference category; ß= beta coefficient, CI = Confidence interval, SFL = Standardised factor loading, FL= Factor loading, SE = Standard error, CFI = Bentler Comparative Fit Index, TLI = Tucker-Lewis Index, GFI = Goodness-of-Fit Index, AGFI = Adjusted Goodness-of-Fit Index, RMSEA = Root Mean Square Error of Approximation, SRMR = Standard Root Mean Square Residual, Df = Degrees of freedom
Bold text = *p* < 0.05
Competencies R^2^ = 17.1%; Gender norms R^2^ = 32.5%; Gender attitudes R^2^ = 42.1%; Psychological distress R^2^ = 8.2%; Gender roles R^2^ = 65.9%; Sex R^2^ = 8.6%
Note:
1) The regression analysis was performed simultaneously in one SEM model and was adjusted for: age, work (working in family business or not working in family business), aspiration for higher education (aspiration for secondary school, teacher training, university, or no such aspiration), disability (living with disability or without disability), language spoken in the household (vernacular Afan Oromo, Afar, Amharic or Somali), urbanisation (living in urban or rural area), father living in household (living or not living in household), asset index (sum score capturing material factors in the household), sampling mode (purposeful or non-purposeful), and marital status (married before age 18 or not), regressed on the two exogeneous variables
2) Residual covariances between: gender roles and sex, gender attitudes subscale F4 and psychological distress, gender attitudes subscale F4 and female sex, media and gender roles, resilience and gender attitudes subscale F2 based on the modification indices; regressions: language Afan Oromo predicting gender attitudes (ß:-1.22, SE: 0.10, CI[-1.41; -1.03], p<0.001), urbanisation predicting competencies (ß: -0.03, SE: 0.05, CI[-0.12; 0.07], p<0.581), language Afar predicting gender attitudes (ß: -1.32, SE:0.14, CI[--1.58; -1.06], p<0.001) based on the modification indices.

Table B: Results of the same SEM model as in the manuscript but without the variable club member (N=1,651)

| **Model Fit Statistics** | | | |
| --- | --- | --- | --- |
| **Fit Statistics** | **Value** |  |  |
| Chi-Squared | 927.441, p<0.001 |  |  |
| Df | 132 |  |  |
| CFI | 0.82 |  |  |
| TLI | 0.75 |  |  |
| GFI | 0.91 |  |  |
| AGFI | 0.79 |  |  |
| RMSEA | 0.060 |  |  |
| SRMR | 0.037 |  |  |
| **Confirmatory Factor Analysis** | | | |
| **Latent Construct** | **SFL** | **FL (SE)** |  |
| **Competencies** | | | |
| Index talking to parents | 0.58 | 1.0 (-) |  |
| Resilience Score | 0.65 | 3.73 (0.31) |  |
| **Gender Norms** | | | |
| Media use | 0.59 | 1.0 (-) |  |
| Having a role model | 0.39 | 0.21 (0.02) |  |
| **Gender Attitudes** | | | |
| Gender Attitudes Subscale F1 | 0.60 | 1.0 (-) |  |
| Gender Attitudes Subscale F2 | 0.57 | 0.90 (0.06) |  |
| Gender Attitudes Subscale F4 | 0.61 | 0.89 (0.06) |  |
| **Regression Analysis** | | | |
| **Variable** | **ß (SE)** | **95 % CI** | **p-value** |
| **Outcome: Mental Health** | | | |
| **Gender Attitudes** | -0.10 (0.19) | [-0.46; 0.27] | p=0.608 |
| **Sex** |  |  |  |
| *Ref.: Male* |  |  |  |
| Female | **-0.50** (0.24) | [-0.97; 0.04] | p=0.035 |
| **Gender Roles** | -0.09 (0.07) | [-0.22; 0.04] | p=0.166 |
| **Gender Norms** | 0.21 (0.32) | [-0.42; 0.84] | p=0.514 |
| **Competencies** | **-1.70** (0.38) | [-2.44; -0.95] | p<0.001 |
| **Outcome: Competencies** |  |  |  |
| **Gender Roles** | 0.004 (0.02) | [-0.03; 0.04] | p=0.820 |
| **Gender Norms** | **0.62** (0.08) | [0.48; 0.77] | p<0.001 |
| **Outcome: Gender Attitudes** |  |  |  |
| **Gender Norms** | **0.88** (0.09) | [0.71; 1.06] | p<0.001 |
| **Sex** |  |  |  |
| *Ref.: Male* |  |  |  |
| Female | **0.54** (0.09) | [0.36; 0.71] | p<0.001 |
| **Outcome: Gender Roles** | | | |
| **Gender Norms** | **-1.37** (0.08) | [-1.54; -1.21] | p<0.001 |

Ref. = Reference category; ß= beta coefficient, CI = Confidence interval, SFL = Standardised factor loading, FL= Factor loading, SE = Standard error, CFI = Bentler Comparative Fit Index, TLI = Tucker-Lewis Index, GFI = Goodness-of-Fit Index, AGFI = Adjusted Goodness-of-Fit Index, RMSEA = Root Mean Square Error of Approximation, SRMR = Standard Root Mean Square Residual, Df = Degrees of freedom
Bold text = *p* < 0.05
Competencies R^2^ = 57.9%; Gender norms R^2^ = 74.3%; Gender attitudes R^2^ = 62.1%; Psychological distress R^2^ = 8.0%; Gender roles R^2^ = 35.2%; Sex R^2^ = 8.8%
Note:
1) The regression analysis was performed simultaneously in one SEM model and was adjusted for: age, work (working in family business or not working in family business), aspiration for higher education (aspiration for secondary school, teacher training, university, or no such aspiration), disability (living with disability or without disability), language spoken in the household (vernacular Afan Oromo, Afar, Amharic or Somali), urbanisation (living in urban or rural area), father living in household (living or not living in household), asset index (sum score capturing material factors in the household), sampling mode (purposeful or non-purposeful), and marital status (married before age 18 or not), regressed on the two exogeneous variables
2) Residual covariances between: gender roles and sex, gender attitudes subscale F4 and psychological distress, gender attitudes subscale F4 and female sex, media and gender roles, resilience and gender attitudes subscale F2 based on the modification indices; regressions: language Afan Oromo predicting gender attitudes (ß:-0.71, SE: 0.12, CI[-0.94; -0.48], p<0.001), urbanisation predicting competencies (ß: 0.31, SE: 0.06, CI[0.19; 0.43], p<0.001), language Afar predicting gender attitudes (ß: -0.67, SE:0.15, CI[-0.96; -0.38], p<0.001) based on the modification indices.

Table C: Results of the same SEM model as in the manuscript but without the variable role model (N=1,654)

| **Model Fit Statistics** | | | |
| --- | --- | --- | --- |
| **Fit Statistics** | **Value** |  |  |
| Chi-Squared | 899.048, p<0.001 |  |  |
| Df | 132 |  |  |
| CFI | 0.83 |  |  |
| TLI | 0.76 |  |  |
| GFI | 0.91 |  |  |
| AGFI | 0.80 |  |  |
| RMSEA | 0.059 |  |  |
| SRMR | 0.037 |  |  |
| **Confirmatory Factor Analysis** | | | |
| **Latent Construct** | **SFL** | **FL (SE)** |  |
| **Competencies** | | | |
| Index talking to parents | 0.58 | 1.0 (-) |  |
| Resilience Score | 0.64 | 3.70 (0.30) |  |
| **Gender Norms** | | | |
| Media use | 0.59 | 1.0 (-) |  |
| Being a club member | 0.42 | 0.23 (0.02) |  |
| **Gender Attitudes** | | | |
| Gender Attitudes Subscale F1 | 0.60 | 1.0 (-) |  |
| Gender Attitudes Subscale F2 | 0.57 | 0.90 (0.06) |  |
| Gender Attitudes Subscale F4 | 0.62 | 0.89 (0.06) |  |
| **Regression Analysis** | | | |
| **Variable** | **ß (SE)** | **95 % CI** | **p-value** |
| **Outcome: Mental Health** | | | |
| **Gender Attitudes** | -0.09 (0.18) | [-0.43; 0.25] | p=0.592 |
| **Sex** |  |  |  |
| *Ref.: Male* |  |  |  |
| Female | **-0.49** (0.24) | [-0.96; -0.03] | p=0.038 |
| **Gender Roles** | -0.10 (0.07) | [-0.23; 0.03] | p=0.124 |
| **Gender Norms** | 0.17 (0.31) | [-0.43; 0.77] | p=0.583 |
| **Competencies** | **-1.66** (0.39) | [-2.42; -0.90] | p<0.001 |
| **Outcome: Competencies** |  |  |  |
| **Gender Roles** | 0.005 (0.02) | [-0.03; 0.04] | p=0.792 |
| **Gender Norms** | **0.63** (0.07) | [0.49; 0.77] | p<0.001 |
| **Outcome: Gender Attitudes** |  |  |  |
| **Gender Norms** | **0.87** (0.09) | [0.70; 1.04] | p<0.001 |
| **Sex** |  |  |  |
| *Ref.: Male* |  |  |  |
| Female | **0.52** (0.09) | [0.35; 0.70] | p<0.001 |
| **Outcome: Gender Roles** | | | |
| **Gender Norms** | **-1.38** (0.08) | [-1.55; -1.22] | p<0.001 |

Ref. = Reference category; ß= beta coefficient, CI = Confidence interval, SFL = Standardised factor loading, FL= Factor loading, SE = Standard error, CFI = Bentler Comparative Fit Index, TLI = Tucker-Lewis Index, GFI = Goodness-of-Fit Index, AGFI = Adjusted Goodness-of-Fit Index, RMSEA = Root Mean Square Error of Approximation, SRMR = Standard Root Mean Square Residual, Df = Degrees of freedom
Bold text = *p* < 0.05
Competencies R^2^ = 58.5%; Gender norms R^2^ = 74.5%; Gender attitudes R^2^ = 61.0%; Psychological distress R^2^ = 7.9%; Gender roles R^2^ = 35.2%; Sex R^2^ = 9.0%
Note:
1) The regression analysis was performed simultaneously in one SEM model and was adjusted for: age, work (working in family business or not working in family business), aspiration for higher education (aspiration for secondary school, teacher training, university, or no such aspiration), disability (living with disability or without disability), language spoken in the household (vernacular Afan Oromo, Afar, Amharic or Somali), urbanisation (living in urban or rural area), father living in household (living or not living in household), asset index (sum score capturing material factors in the household), sampling mode (purposeful or non-purposeful), and marital status (married before age 18 or not), regressed on the two exogeneous variables
2) Residual covariances between: gender roles and sex, gender attitudes subscale F4 and psychological distress, gender attitudes subscale F4 and female sex, media and gender roles, resilience and gender attitudes subscale F2 based on the modification indices; regressions: language Afan Oromo predicting gender attitudes (ß:-0.79, SE: 0.11, CI[-1.01; -0.57], p<0.001), urbanisation predicting competencies (ß: 0.30, SE: 0.06, CI[0.19; 0.42], p<0.001), language Afar predicting gender attitudes (ß: -0.63, SE: 0.15, CI[-0.92; -0.34], p<0.001) based on the modification indices.
