# Supplementary material for: Gendered pathways to adolescent mental health: An empirical assessment of a new conceptual framework
Source: PLOS Glob Public Health. 2026 Nov 10;6(11):e0006728. doi: 10.1371/journal.pgph.0006728 (PMC13750542; doi:10.1371/journal.pgph.0006728)
Supplement: Supplementary file 5 [file pgph.0006728.s006.docx]

**Response to reviewers**

Dear Editorial Office and Reviewers,

Thank you for the opportunity to revise this manuscript. You will find our point-by-point responses to your revision requests below.

Kind regards,

The team

______________________________________________________________________________________

Journal Requirements:

1. Please note that PLOS Global Public Health has specific guidelines on code sharing for submissions in which author-generated code underpins the findings in the manuscript. In these cases, all author-generated code must be made available without restrictions upon publication of the work. Please review our guidelines at https://journals.plos.org/globalpublichealth/s/materials-and-software-sharing#loc-sharing-code and ensure that your code is shared in a way that follows best practice and facilitates reproducibility and reuse.

Thank you. We have made our code available.

2. Please update your online Competing Interests statement. If you have no competing interests to declare, please state: "The authors have declared that no competing interests exist."

We have updated our online Competing Interests statement and have declared that no competing interests exist.

3. For studies involving third-party data, we encourage authors to share any data specific to their analyses that they can legally distribute. PLOS recognizes, however, that authors may be using third-party data they do not have the rights to share. When third-party data cannot be publicly shared, authors must provide all information necessary for interested researchers to apply to gain access to the data. (https://journals.plos.org/globalpublichealth/s/data-availability#loc-acceptable-data-access-restrictions)

For any third-party data that the authors cannot legally distribute, they should include the following information in their Data Availability Statement upon submission:

a) A description of the data set and the third-party source

b) If applicable, verification of permission to use the data set

c) Confirmation of whether the authors received any special privileges in accessing the data that other researchers would not have

d) All necessary contact information others would need to apply to gain access to the data

We have updated our Data Availability Statement, which reads as follows:

The data on which the results of our study are based are the publicly available Gender and Adolescence: Global Evidence (GAGE) data, archived by the data provider ‘UK data service’ (see https://ukdataservice.ac.uk/). The datasets include the second round of data collection for Ethiopia in 2020 (core respondent module & adult female module). These datasets are publicly available and can be accessed free of charge after registration with the UK Data Service and the set-up of a research project. We confirm that we did not receive any special privileges in accessing the data that are not available to other researchers.

4. Please ensure that you refer to Table 1 in your text as, if accepted, production will need this reference to link the reader to the table.

Thank you. We have mistakenly labelled Table 1 as Table 3.

5. If the reviewer comments include a recommendation to cite specific previously published works, please review and evaluate these publications to determine whether they are relevant and should be cited. There is no requirement to cite these works unless the editor has indicated otherwise.

________________________________________________________________________________________

Dear Reviewers,

To address some of your overarching comments, we have included a revised SEM model that takes model fit statistics into greater account. Accordingly, we have excluded all variables with factor loadings below 0.4 in the CFA and have incorporated modification indices more systematically, without, however, changing the SEM model in a way that would no longer correspond to the GAM framework. This led to an SEM model with slightly higher model fit statistics. We have compared the initial SEM model with the revised SEM model in a sensitivity analysis (AIC/BIC):

|  | **Initial model** | **Revised model** |
| --- | --- | --- |
| Chi^2^ | 1648.365 | 1041.886 |
| Df | 288 | 155 |
| CFI | 0.76 | 0.82 |
| TLI | 0.72 | 0.75 |
| RMSEA | 0.055 | 0.059 |
| SRMR | 0.042 | 0.038 |
| AIC | 80521.548 | 61349.390 |
| BIC | 80908.023 | 61700.984 |

**Reviewer #1:**

Model Fit Requires Stronger Justification

The discussion should better justify why the model remains acceptable despite these shortcomings and explain how poorly fit may influence the validity of the conclusions.

Thank you. We have included a paragraph in the limitations section to discuss the shortcomings of the SEM model more specifically, as well as the consequences for its interpretation:

“Limitations also relate to our analytical approach. While absolute fit indices such as RMSEA and SRMR indicate a small misfit per degree of freedom and a small average residual correlation, relative fit indices such as CFI and TLI fell below recommended thresholds, indicating that the specified model might not fully capture the underlying data structure. Several factors may contribute to this, including model complexity, large sample size, numerous covariates necessary to operationalise the intersectional lens, local model misspecifications, and the use of sample weights (38). We investigated modification indices to identify potential sources of misfit. We incorporated residual covariances and regressions between latent variables and covariates (so as not to change the structural model) into our SEM model. We did not include any further indicators in the latent variables, as this would alter the interpretation of the constructs and thus result in an SEM model that no longer corresponds to the GAM framework. Since model evaluation should be guided by both theory and statistical fit (38), we assessed model fit by combining theoretical considerations with fit statistics. Although the remaining modification indices do not show a clear pattern suitable for any interpretations regarding other sources of misfit, a substantial unexplained misfit remains. Therefore, the SEM model does not provide an optimal representation of the data but can be interpreted as an initial assessment of the plausibility of the proposed pathways. Future research should examine the proposed relationships using alternative model specifications that are not constrained by the GAM framework and adapt the framework accordingly.”

The paper would benefit from clearer definitions distinguishing gender norms, gender attitudes, and gender roles, as these concepts occasionally overlap conceptually.

We have added the following paragraph in the introduction section to distinguish gender norms, roles, and attitudes more explicitly: “As gender norms, roles, and attitudes are often conflated conceptually, we define gender norms as the “spoken and unspoken rules of societies about the acceptable behaviours of girls and boys […] - how they should act, look, and even think or feel” (10) while we define gender attitudes as the individual perceptions toward these gender norms (9). Gender roles concern the “sexual division of labor, where specific tasks are strictly assigned based on gender” (16).”

We have also added additional explanations in line with your comment on gender norms, which you can find further below.

Interpretation of Non-significant Findings

- The discussion provides extensive explanations for non-significant associations, which risks overinterpreting the findings. The interpretation should remain more cautious and emphasize the exploratory nature of these results.The wording should consistently reflect that only associations not causal pathways can be inferred.

This comment has also been raised by reviewer 3. We have removed remaining causal wording (e.g.,”resulting in better mental health”). We have also limited extensive explanations of non-significant findings by removing a couple of sentences.

Some proxies used to measure gender norms (club membership, role model, media use) appear to capture broader social participation rather than gender norms specifically. The authors should better justify these indicators and discuss the possibility of measurement bias.

We agree that the operationalisation of the gender norms construct does not seem straightforward. We have explained in greater detail why we have chosen these proxies for measuring the gender norms construct in the GAM framework:

“While we could not operationalise the household and political levels of gender norms due to a lack of appropriate items, we operationalised the community level with a peer violence index and being a club member, and the digital level with having a role model, media, and social media use. We chose these operationalisations for the gender norms construct because the GAM framework operationalises this construct based on actors who carry gender norms that influence adolescents’ gender attitudes in different social environment levels, with the community level consisting of the indicators peers and school, and the digital level of media, social media, and influencers (15). We recoded these variables so that higher values indicate higher exposure to gender norms. A detailed description of the recoded variables is shown in Supplementary Information A.”

We have also added the resulting limitation in the limitations section. “Fourth, we operationalised the gender norms construct in accordance with propositions from the GAM framework. However, the indicators measure a potential exposure to gender norms rather than gender norms specifically. This may introduce measurement bias and limit construct validity.”

Similarly, and in line with your comment earlier about conceptually distinguishing gender norms, roles, and attitudes, we have also made our operationalisation of gender roles more explicit: “Gender roles can be defined by the sexual division of labour, where specific tasks are assigned based on gender and are operationalised in the GAM framework by time spent on gendered activities. Accordingly, we operationalised the gender roles construct with a gendered activities score. A higher score translates to more hours spent on activities per day, indicating adherence to more traditional gender roles.”

We have also added a short explanation for the operationalisation of gender attitudes: “We operationalised gender attitudes using the GAGE gender norms scale (although it is labelled as a gender norms scale, it measures adolescents’ perceptions of gender norms, that is, gender attitudes)….“

The rationale for reducing the gender attitudes scale from 32 to 12 items should be summarized briefly in the main manuscript rather than relying mainly on supplementary material.

We have provided the rationale for reducing the gender attitudes scale in the main manuscript:

“We operationalised gender attitudes using the GAGE gender norms scale (although it is labelled as a gender norms scale, it measures adolescents’ perceptions of gender norms, that is, gender attitudes). In previous work (23), we explored the factor structure of this newly developed scale using data from an earlier GAGE round. The scale combines items from existing adolescent gender norms scales with items developed specifically for the GAGE project, but neither the original scales nor the GAGE scale have undergone prior empirical validation for the specific study population under investigation. Therefore, we explored its factor structure in the present sample. Following methodological recommendations and our previous approach, we used EFA, as the underlying factor structure could not be assumed a priori, and subsequently tested the hypothesised factor model of the EFA in a confirmatory setting using CFA (see Supplementary Information B) (20). Ultimately, the CFA suggested retaining 20 of the scale’s original 32 items as a latent variable with seven sum scores (hereafter referred to as gender attitudes subscale F1-F7). We recoded the scale so that a higher score indicates more gender egalitarian attitudes.”

Some discussion paragraphs are lengthy and repetitive, particularly when explaining unexpected findings. Condensing these sections would improve readability.

We agree. We have limited extensive explanations of non-significant findings.

**Reviewer #2:**

1. Abstract:While the topic is very interesting, the abstract lacks methodological clarity. The authors should clearly state **when** and **where** the study was conducted, **how** data collection tools were used, and provide a clear, specific description of the data analysis methods.follow

We used an existing dataset and therefore conducted a secondary analysis. We did not use any data collection tools ourselves. In the Abstract, we have added the missing information for the methods section: “We used an existing cross-sectional dataset from Ethiopia (2020) from the Gender and Adolescence: Global Evidence (GAGE) project to explore the associations between sex, gender norms, psychological competencies, gender attitudes, gender roles, with the latter two also serving as mediators, and psychological distress (GHQ-12), using Structural Equation Modelling (SEM).”

‎2. Methodology: The methodology section is currently difficult to follow and lacks transparency. It needs to be rewritten to clearly explain the study design, sampling procedures, data collection process, and statistical analysis plan so that it can be easily understood and reproduced.

Similarly to the changes in the Abstract, we have revised the “data” chapter to emphasise the cross-sectional, secondary nature of our study. We have also added information about the data collection procedures used in the GAGE project. The statistical analysis plan is detailed in chapter 2.4. The “data” chapter now reads as follows:

“Our secondary data analysis draws on quantitative data from the Gender & Adolescence: Global Evidence (GAGE) project, a ten-year (2015-2024) mixed-methods longitudinal research and evaluation programme. GAGE surveys 18,000 adolescents aged 10 to 19 years and other community members across six countries in the Global South, with a particular focus on vulnerable adolescents, such as those with disabilities, who married before the age of 18, refugees, adolescent mothers and out-of-school youth (20). We use cross-sectional data from the second round of data collection in Ethiopia in 2020, as it includes validated tools for mental health and gender-related variables. Data collection took place in six locations in Ethiopia. A household census was used to identify potential participants (21). In addition to the random sample, out-of-school adolescents and adolescents with disabilities were purposefully collected by the GAGE project (20). Sampling weights reflect the probability of selection into the study sample and ensure the representativeness of the target population in the study area. Further details on data collection procedures are described in Baird et al. (21). The data on which the results of our study are based are third-party data that are publicly archived by the data provider ‘UK data service’. They are available free of charge for any researcher following registration with the data provider. We have complied with the data provider’s terms of use.”

‎3. Conclusion and Recommendations: The conclusion is too general and does not directly reflect the study's key findings. Similarly, the recommendations are not specific enough; they should be actionable, context-specific, and directly derived from the study results.

Thank you for pointing this out. We have revised the conclusion section to better align it with the study findings and the Ethiopian context, and we have formulated more concrete recommendations. The conclusion section now reads as follows:

“We found significant associations between psychological competencies and psychological distress; sex and psychological distress; sex and gender attitudes; and between gender norms and psychological competencies, gender attitudes, and gender roles among Ethiopian adolescents. Therefore, the gender-related constructs were mostly associated with each other, thereby potentially attenuating their individual effects on psychological distress. This and a potential interplay of gender-related pathways should be further investigated in longitudinal study designs that also better investigate possible causality and dose-response relationships.”

‎**Reviewer #3:**

The article “Gendered pathways to adolescent mental health: An empirical assessment of a new conceptual framework” addresses an important topic, builds on a conceptual framework that is a valuable contribution to the literature, and uses an intersectionality-informed SEM approach that is well motivated by previous literature. However, there are some important issues that need to be resolved before the article is accepted for publication.

1.The measurement structure of "Gender Attitudes" is inconsistent across the manuscript. In the main article text, the authors note that they used the 12-item version of the scale, but in the supplementary material, they note that the seven-factor, 20-item CFA model provides the best fit to the data. This is an important discrepancy: the supplementary material says the 20-item version is better, but the article uses the 12-item version without sufficient justification. In addition, the text refers to the scale as the gender attitudes scale, but the supplementary material labels it as gender norms, which could be confusing to readers. Please address these inconsistencies and provide a clear rationale for the final choice of measurement structure. I would also recommend labeling the scale as gender attitudes consistently throughout the main text, tables, and supplementary material rather than going back and forth between gender attitudes and gender norms, especially since the article includes another, separate gender norms construct.

Thank you for pointing this out. We had included the latent seven-factor variable in the SEM model but have forgotten to remove the 12-item version from the measures section and the sample characteristics. We have harmonised this across the manuscript.

We now refer to the gender norms scale consistently as the gender attitudes scale, except the first mention, where we have added a brief explanation - both in the manuscript and in the supplementary material.

2. Lines 155–159 state that four indicators with loadings between 0.2–0.4 were retained for theoretical relevance to the GAM framework. Please name these four indicators explicitly in the main text and provide a specific theoretical justification for the retention of each, rather than a general reference to the GAM framework. It could also be worthwhile to report a sensitivity analysis excluding these indicators, to show how robust the results are to the decision to keep/exclude these indicators.

We agree. The initial SEM model had an AIC of 80521.548 and a BIC of 80908.023. The sensitivity analysis showed that the AIC and BIC of the same model but without loadings <0.4 were lower than the initial model (AIC: 65409.883, BIC: 65756.029). Therefore, we have excluded all indicators with loadings below 0.4 in the revised SEM model, which now has an AIC of 61349.390 and a BIC of 61700.984. The respective paragraph now reads as follows:

“We assessed localised areas of model misfit using standardised factor loadings with the commonly recommended threshold of 0.4. Due to factor loadings below 0.4, we removed the indicators social media, expression of opinion to friends, expression of opinion to elders, the peer violence index, and the gender attitudes subscales F3, F5, F6, and F7.”

2.I am not fully convinced by the operationalization of the gender norms construct. In particular, using things like club participation or having a role model as indicators of higher exposure to gender norms seems like a stretch, and it muddles the interpretation of the results. For example, “higher exposure to gender norms” was found to be associated with higher levels of competencies, but if the items creating the gender norms variable include club participation and having a role model, it may be that adolescents are developing competencies through those relationships for other reasons, not because of gender role exposure. The paper also states that “higher exposure to gender norms was significantly associated with "fewer hours spent on gendered activities (gender roles construct)”, but again, it may be that things like club participation or use of social media are taking up time, so there is less time to spend on gendered activities. Please provide more justification as to why you chose to include these variables as indicators of gender norm exposure, and consider running sensitivity analyses without some of these indicators because they don’t seem to actually match the construct they are intended to measure, in my opinion.

We agree that the operationalisation of the gender norms construct does not seem straightforward. However, we have operationalised the gender norms construct according to the proposed operationalisation of the GAM framework. We have explained this more explicitly when describing the gender norms measures in the measures section and have added an explanation in the limitations section. The description of the operationalisation of the gender norms construct in the measures section now reads as:

“While we could not operationalise the household and political levels of gender norms due to a lack of appropriate items, we operationalised the community level with a peer violence index and being a club member, and the digital level with having a role model, media, and social media use. We chose these operationalisations of the gender norms construct because the GAM framework operationalises this construct based on actors who carry gender norms that influence adolescents’ gender attitudes in different social environment levels, with the community level consisting of peers and school, and the digital level represented by media, social media, and influencers (15). We recoded these variables so that higher values indicate higher exposure to gender norms. A detailed description of the recoded variables is shown in Supplementary Information A.”

We have also added a sentence in the limitations section: “Fourth, we operationalised the gender norms construct in accordance with propositions from the GAM framework. However, the indicators measure a potential exposure to gender norms rather than gender norms specifically. This may introduce measurement bias and limit construct validity.”

Due to several reviewer comments, we have revised the SEM model in which we have removed the variable social media from the gender norms construct, as it showed values below 0.4 in the CFA. After specifying the final model, we have conducted further sensitivity analyses, which we present in the Table below, and in more detail in the supplementary material of the manuscript. The sensitivity analyses are only minimally better than our revised model in terms of AIC/BIC, and the model without both club member and role model shows reduced model fit statistics, or those without one variable show nearly the same model fit statistics. Therefore, we have decided to keep the operationalisation of the GAM framework.

|  | **Revised model** | **Revised model without both club member and role model** | **Revised model without club member** | **Revised model without role model** |
| --- | --- | --- | --- | --- |
| Chi^2^ | 1041.886 | 1088.743 | 927.441 | 899.048 |
| Df | 155 | 111 | 132 | 132 |
| CFI | 0.815 | 0.760 | 0.820 | 0.827 |
| TLI | 0.751 | 0.650 | 0.748 | 0.758 |
| RMSEA | 0.059 | 0.073 | 0.060 | 0.059 |
| SRMR | 0.038 | 0.056 | 0.037 | 0.037 |
| AIC | 61349.390 | 57491.765 | 59242.089 | 59309.053 |
| BIC | 61700.984 | 57816.422 | 59582.865 | 59649.943 |

We have added a couple of sentences both in the methods section and in the results section, respectively:

“We further conducted sensitivity analyses to test the robustness of the gender norms construct by removing both variables club member and role model (Supplementary material, Table 4), by removing only the variable club member (Supplementary material, Table 5), and by removing only the variable role model (Supplementary material, Table 6).”

“The sensitivity analyses testing the robustness of the gender norms construct showed that the differences in model fit statistics between the initial model and the three sensitivity analyses were fairly minor, and the model with both variables (club member and role model) removed showed reduced model fit statistics (Supplementary Material, Tables 4-6).”

4. The overall SEM model fit is below threshold on several indices, and this should be discussed more specifically. Please report diagnostics like modification indices to indicate the source(s) of misfit and discuss how this affects confidence in the reported paths.

Thank you. We have included modification indices more systematically in the revised SEM model and have discussed them more specifically in the limitations section:

“Limitations also relate to our analytical approach. While absolute fit indices such as RMSEA and SRMR suggest a small misfit per degree of freedom and small standardised discrepancies between the observed and model-implied correlations, relative fit indices such as CFI and TLI fell below recommended thresholds, indicating that the specified model might not fully capture the underlying data structure. Several factors may contribute to this, including model complexity, large sample size, numerous covariates necessary to operationalise the intersectional lens, local model misspecifications, and the use of sample weights (38). We investigated modification indices to identify potential sources of misfit. We incorporated residual covariances and regressions between latent variables and covariates (so as not to change the structural model) into our SEM model. We did not include any further indicators in the latent variables, as this would alter the interpretation of the constructs and thus result in an SEM model that no longer corresponds to the GAM framework. Since model evaluation should be guided by both theory and statistical fit (38), we assessed model fit by combining theoretical considerations with fit statistics. Although the remaining modification indices do not show a clear pattern suitable for any interpretations regarding other sources of misfit, a substantial unexplained misfit remains. Therefore, the SEM model does not provide an optimal representation of the data but can be interpreted as an initial assessment of the plausibility of the proposed pathways. Future research should examine the proposed relationships using alternative model specifications that are not constrained by the GAM framework.”

5. The conclusion overstates what the mediation analysis actually found. Lines 376–378 state: "The gender-related constructs are strongly interlinked, thereby attenuating their individual effects on psychological distress […] The interplay […] should be considered when developing interventions." However, all formal mediation tests were non-significant, and only 5 of 14 total regression paths reached significance, with only one directly predicting the outcome. This "attenuation via interrelation" claim is not confirmed statistically, and drawing these implications for interventions at this stage seems premature. I recommend rephrasing to reflect what was actually shown and framing the proposed ideas as hypotheses for future (potentially longitudinal) research rather than a substantiated conclusion.

Thank you. We have revised the conclusions, which was also a comment of another reviewer. It now reads as:

“We found significant associations between psychological competencies and psychological distress; sex and psychological distress; sex and gender attitudes; and between gender norms and psychological competencies, gender attitudes, and gender roles among Ethiopian adolescents. Therefore, the gender-related constructs were mostly associated with each other, thereby potentially attenuating their individual effects on psychological distress. This and a potential interplay of gender-related pathways should be further investigated in longitudinal study designs that also better investigate possible causality and dose-response relationships. ”

6. Peer violence index appears to be dropped without explanation. Line 112 describes the community-level gender norms operationalization as including a peer violence index as one of 5 indicators, and Table 1 lists the peer violence index under Gender Norms descriptives. However, Table 2's CFA for the Gender Norms construct includes only four indicators, and the peer violence index is absent from the table. Please clarify whether/why this variable was excluded from the final model.

We have removed the peer violence index as the variable showed factor loadings below 0.2 in the SEM model. As stated above, we have included a justification in the statistical analysis section, which now reads as follows:

“We assessed localised areas of model misfit using standardised factor loadings with the commonly recommended threshold of 0.4. Due to factor loadings below 0.4, we removed the indicators social media, expression of opinion to friends, expression of opinion to elders, the peer violence index, and the gender attitudes subscales F3, F5, F6, and F7.”

7. The R² for psychological distress is a very low value for the paper's primary outcome and central research question (3.8%), but it is only mentioned in a table footnote. Consider discussing this explicitly in the discussion, since it impacts how much weight readers should give to the modeled pathways.

The percentage slightly increased in the revised model to 7.7%. We have integrated your suggestion in the paragraph at the end of the discussion section, which now reads as:

“Consequently, the gender-related pathways analysed in the SEM model appear to be more interrelated with each other than directly associated with mental health. This is also reflected in the low R2 of the primary outcome of psychological distress, which indicates that our model only explains 7.7% of the variation in mental health. Besides the fact that we controlled for gender-related constructs, another possible explanation may be the overall relatively high self-perceived mental health status of the Ethiopian adolescents. This could have limited the variability in the outcome measure and reduced the ability to detect subtle differences. Moreover, in countries with higher gender inequity, sex differences in mental health tend to be smaller. Traditional gendered expectations may be perceived as normative rather than oppressive (33), thereby mitigating their direct effect on psychological distress. Psychological competencies might be a more proximal factor for psychological distress than the other gender-related constructs and might therefore impact mental health more immediately. In this SEM model, we focused on gender-related pathways to adolescent mental health and thus may have left out other influential predictors for psychological distress.”

8. In the discussion, when discussing non-significant findings, please use more precise language (e.”g., in the observed but non-significant direction”, "would be consistent with") rather than causal phrasing (e.”g., resulting in better mental health”). The current phrasing risks overstating the strength of evidence for these pathways.

We have ensured that causal phrasing was removed in several instances in the discussion part when presenting non-significant findings.

Minor comments:

1. Please ensure correct spelling across all documents related to the paper (e.g., “Communalitites” in the supplementary material).

We have ensured correct spelling across all documents.

2. "Herman's Single Factor" should be "Harman's Single Factor Test."

Thank you. We have revised this accordingly.

3. "SRMR" vs. "SMSR”: please standardize spelling/abbreviation throughout.

We have standardised the spelling.

4. Figure 2 shows unlabeled indicator boxes for Gender Norms and Competencies, and it has no indicators visible for Gender Attitudes, despite Table 2 listing four indicators for it. Please ensure the figure is complete and consistent with Table 2.

This mistake was introduced when changing the format from jpn to tif. We have updated the Figure and have ensured it is consistent with Table 2.

5. Please clarify how sample weights were constructed given that the GAGE Ethiopia sample is described in the paper as purposefully selected, as well as how this affects the representativeness claim in the strengths and limitations section.

The sample weights were constructed by the GAGE project. We have added a sentence in the “data” chapter that explains the construction of the sample weights: “Data collection took place in six locations in Ethiopia. A household census was used to identify potential participants (21). In addition to the random sample, out-of-school adolescents and adolescents with disabilities were purposefully collected by the GAGE project (20). Sampling weights reflect the probability of selection into the study sample and ensure the representativeness of the target population in the study area.”

We have also revised the sentence regarding representativeness in the limitations section: “The findings are representative of GAGE’s target population in the study area, but may also be relevant for a similar adolescent population in countries with comparable cultural contexts.”
