# Supplementary material for: Gendered pathways to adolescent mental health: An empirical assessment of a new conceptual framework
Source: PLOS Glob Public Health. 2026 Nov 10;6(11):e0006728. doi: 10.1371/journal.pgph.0006728 (PMC13750542; doi:10.1371/journal.pgph.0006728)
Supplement: Supplementary file 6 [file pgph.0006728.s007.docx]

**Response to reviewers**

Dear Editorial Office and Reviewers,

We thank you for your detailed and constructive feedback. We also thank you for the opportunity to revise this manuscript. You will find our point-by-point responses to your revision requests below.

Kind regards,

The team

**Reviewer #1:**

there seems to be a change in analytic sample size, the analytic sample has increased from 1,584 participants in the original manuscript to 1,651 in the revised analysis. Please briefly explain the reason for this change.

The reason for the change in sample size is that we have modified some variables in the revised Structural Equation model (SEM) compared to the previous model. The SEM models the complete cases based on the variables included in the model. Therefore, a change in the variables also changes the complete cases that can be analysed, and thus the analytical sample size.

Although the revised model has improved, the CFI and TLI remain below conventional thresholds. The authors now acknowledge this appropriately in the limitations section. I suggest maintaining this cautious framing throughout the Discussion and Conclusions and avoiding language that might imply that the proposed framework has been confirmed by these data.

Thank you. We have revised the Conclusion section based on your subsequent comments (see below). In addition, we have toned down interpretations in the Discussion section in several instances and have added sentences such as “However, the non-significant finding and cross-sectional study design do not support this hypothesis.” or “Nevertheless, the directionality identified in this study must be confirmed in future studies, which should also investigate hypotheses regarding potential underlying mechanisms using longitudinal study designs.”

Furthermore, since you are referring to the proposed framework, we have added the following last sentence to the first paragraph in the Discussion section, which now reads as follows: “In this study, we empirically tested the hypothesised pathways from the GAM framework (15). We employed SEM to simultaneously model the set of gender-related constructs and their relationships with mental health, including a wide range of covariates to operationalise the intersectional lens applied in the framework. Based on these data collected from Ethiopian adolescents, six of ten regression paths and none of four mediation paths were statistically significant. *Since the SEM model does not provide an optimal representation of the data, the significant paths should be interpreted as an initial assessment of the plausibility of the proposed pathways.* “

The revised manuscript describes the negative association between female sex and psychological distress as indicating a “protective factor.” Given the cross-sectional design, it would be more accurate to state that female sex was associated with lower psychological distress in this model, without implying a protective or causal effect.

We have removed the mention of a protective factor both in the Results and in the Discussion sections, and have only emphasised the negative association.

The sentence in the Results section now reads as: “Being a girl (vs. being a boy) was also, on average, associated with lower psychological distress.”

The sentence in the Discussion section now reads as: “The negative directionality of the significant association between female sex (vs. male sex) and psychological distress is noteworthy, indicating that being an adolescent girl is associated with lower psychological distress and thus better mental health.“

All formal mediation effects remain non-significant. The revised conclusion is more cautious than before, but the phrase that interrelations may be “attenuating” individual effects still reads somewhat mechanistically. I would suggest wording this more conservatively—for example, that the constructs were more strongly associated with one another than with psychological distress, and that the mechanisms underlying these relationships require longitudinal investigation.

We have integrated this suggestion both in the abstract: “The gender-related constructs were associated with each other rather than with psychological distress. The mechanisms underlying these relationships and a potential interplay of gender-related pathways require longitudinal investigation.“, and in the Conclusion: “Therefore, the gender-related constructs were associated with each other rather than with psychological distress. The mechanisms underlying these relationships and a potential interplay of gender-related pathways should be further investigated using longitudinal study designs that can establish causality and better investigate dose-response relationships.”

**Reviewer #3:**

I thank the authors for their careful revision and detailed response. Several of my comments have been fully addressed, but some comments are only partly addressed, and there are some errors that bear on the paper's main conclusion. I therefore recommend a further round of minor revision.

Responses to previous comments:

Comment 1 (gender attitudes measurement): The main text has been harmonised, but S1 still describes gender attitudes as a 12-item sum score, and Supplementary Tables 4–6 still label the indicators "Gender Norms Scale." In addition, the revised model retains only subscales F1, F2 and F4. Please state which content domains these cover and acknowledge that the construct is now narrower than the validated 20-item scale.

Thank you for pointing this out. We have adapted the Tables accordingly and have revised the description of the gender attitudes measurement in S1: “ We operationalised gender attitudes with the GAGE gender norms scale (conceptually measuring gender attitudes). Each item is scored on a three-level scale with (1) indicating agreement, (2) indicating partial agreement and (3) indicating disagreement with the statement. We recoded all items with a reverse question to ensure that agreement suggests a gender-unequal response. Thereafter, we explored the factor structure of the gender attitudes scale using Exploratory and Confirmatory Factor Analysis, which suggested retaining 20 of the scale’s original 32 items as a latent variable with seven sum scores. A higher score indicates more gender egalitarian attitudes.”

In addition, we have added the following sentences to the Methods section regarding the Structural Equation Model: “Removing these gender attitudes subscales results in a gender attitudes construct that is narrower than the validated 20-item scale. The remaining gender attitudes subscales F1, F2, and F4 in the SEM model cover 10 items from the content domains of education, violence, and time use.”

Comment 2 (low loadings): The voice score was part of the competencies construct in the original model but no longer appears in Table 2, and it is not among the removed indicators listed at lines 179–181. Please clarify.

Thank you. The voice indicator also had a loading of 0.395, which is below 0.4, but we had forgotten to include it among the other removed indicators. We have now added the voice indicator to the list of removed indicators.

Comment 3 (operationalization of gender norms): The sensitivity analyses are useful, and the estimates are reassuringly stable when club membership or role model is removed individually (Supplementary Tables 5–6). However, the main text (lines 290–293) summarizes these analyses only in terms of model fit. It should also report that the media-only model (Supplementary Table 4) changes two findings: sex is no longer significantly associated with distress (p=0.058), and gender roles becomes significantly associated with distress (p=0.044). Also, the "Outcome: Competencies" rows in Supplementary Table 4 are identical to the main model even though competencies R² falls from 65.2% to 17.1%, which suggests a copy-paste error.

We could indeed identify a copy-paste error in the outcome competencies, which we have corrected. We have also added a more substantive interpretation to the explanations regarding model fit. The media-only model shows an estimation error in R, which we have addressed in the manuscript.

The entire paragraph on sensitivity analyses now reads as follows: “The sensitivity analyses testing the robustness of the gender norms construct showed that the differences in model fit statistics between the initial model and the three sensitivity analyses were fairly minor, and the model with both variables (club member and role model) removed showed reduced model fit statistics (Supporting information C, Tables 6-8). The estimates remain stable when the variable club membership or role model is removed individually (Tables 7–8). When both variables are removed (Table 6), the media-only model no longer has a positive-definite covariance matrix for the residuals of the observed variables. This means that the matrix is singular and has at least one eigenvalue that is zero or negative, which could be attributed to the now single-indicator latent construct gender norms, with media as the only remaining variable in the latent construct. This model yields three different findings: sex is no longer significantly associated with psychological distress (p=0.058), and gender roles becomes significantly associated both with psychological distress (p=0.044) and competencies (p<0.001). However, due to the model estimation error, the estimates in the media-only model cannot be considered reliable.”

New comment:

Errors in Table 3: Please check the accuracy of the reported numbers for the total effect of gender norms and the total indirect effect - the numbers included seem inconsistent with the confidence intervals presented.

We have checked the results of the mediation analysis and have found two small errors, which we have corrected accordingly.
