# Supplementary material for: Gendered pathways to adolescent mental health: An empirical assessment of a new conceptual framework
Source: PLOS Glob Public Health. 2026 Nov 10;6(11):e0006728. doi: 10.1371/journal.pgph.0006728 (PMC13750542; doi:10.1371/journal.pgph.0006728)
Supplement: Peer review history

# Peer review: Decision Letter 0

**DOI:** 10.1371/journal.pgph.0006728.r001

**Authors:** Somayeh Hessam

4 Aug 2026

PGPH-D-26-02116

Gendered pathways to adolescent mental health: An empirical assessment of a new conceptual framework

PLOS Global Public Health

Dear Dr. Alaze,

Thank you for submitting your manuscript to PLOS Global Public Health. After careful consideration, we feel that it has merit but does not fully meet PLOS Global Public Health’s publication criteria as it currently stands. Therefore, we invite you to submit a revised version of the manuscript that addresses the points raised during the review process.

Please submit your revised manuscript by Oct 03 2026 11:59PM. If you will need more time than this to complete your revisions, please reply to this message or contact the journal office at globalpubhealth@plos.org. When you're ready to submit your revision, log on to https://www.editorialmanager.com/pgph/ and select the 'Submissions Needing Revision' folder to locate your manuscript file.

Please include the following items when submitting your revised manuscript:

- A letter that responds to each point raised by the editor and reviewer(s). You should upload this letter as a separate file labeled 'Response to Reviewers'.
- A marked-up copy of your manuscript that highlights changes made to the original version. You should upload this as a separate file labeled 'Revised Manuscript with Track Changes'.
- An unmarked version of your revised paper without tracked changes. You should upload this as a separate file labeled 'Manuscript'.

Guidelines for resubmitting your figure files are available below the reviewer comments at the end of this letter.

As the corresponding author, your ORCID iD is verified in the submission system and will appear in the published article. PLOS supports the use of ORCID, and we encourage all coauthors to register for an ORCID iD and use it as well. Please encourage your coauthors to verify their ORCID iD within the submission system before final acceptance, as unverified ORCID iDs will not appear in the published article. *Only*  the individual author can complete the verification step; PLOS staff *cannot*  verify ORCID iDs on behalf of authors.

We look forward to receiving your revised manuscript.

Kind regards,

Somayeh Hessam

Academic Editor

PLOS Global Public Health

Journal Requirements:

1. Please note that PLOS Global Public Health has specific guidelines on code sharing for submissions in which author-generated code underpins the findings in the manuscript. In these cases, all author-generated code must be made available without restrictions upon publication of the work. Please review our guidelines at https://journals.plos.org/globalpublichealth/s/materials-and-software-sharing#loc-sharing-code and ensure that your code is shared in a way that follows best practice and facilitates reproducibility and reuse.

2. Please update your online Competing Interests statement. If you have no competing interests to declare, please state: "The authors have declared that no competing interests exist."

3. For studies involving third-party data, we encourage authors to share any data specific to their analyses that they can legally distribute. PLOS recognizes, however, that authors may be using third-party data they do not have the rights to share. When third-party data cannot be publicly shared, authors must provide all information necessary for interested researchers to apply to gain access to the data. (https://journals.plos.org/globalpublichealth/s/data-availability#loc-acceptable-data-access-restrictions)

For any third-party data that the authors cannot legally distribute, they should include the following information in their Data Availability Statement upon submission:

a) A description of the data set and the third-party source

b) If applicable, verification of permission to use the data set

c) Confirmation of whether the authors received any special privileges in accessing the data that other researchers would not have

d) All necessary contact information others would need to apply to gain access to the data

4. Please ensure that you refer to Table 1 in your text as, if accepted, production will need this reference to link the reader to the table.

5. If the reviewer comments include a recommendation to cite specific previously published works, please review and evaluate these publications to determine whether they are relevant and should be cited. There is no requirement to cite these works unless the editor has indicated otherwise.

Additional Editor Comments (if provided):

[Note: HTML markup is below. Please do not edit.]

Reviewers' comments:

Reviewer's Responses to Questions

**Comments to the Author**

1. Does this manuscript meet PLOS Global Public Health’s publication criteria? Is the manuscript technically sound, and do the data support the conclusions? The manuscript must describe methodologically and ethically rigorous research with conclusions that are appropriately drawn based on the data presented.

Reviewer #1: Partly

Reviewer #2: Partly

Reviewer #3: Partly

**********

2. Has the statistical analysis been performed appropriately and rigorously?

Reviewer #1: Yes

Reviewer #2: Yes

Reviewer #3: Yes

**********

3. Have the authors made all data underlying the findings in their manuscript fully available (please refer to the Data Availability Statement at the start of the manuscript PDF file)?

The PLOS Data policy requires authors to make all data underlying the findings described in their manuscript fully available without restriction, with rare exception. The data should be provided as part of the manuscript or its supporting information, or deposited to a public repository. For example, in addition to summary statistics, the data points behind means, medians and variance measures should be available. If there are restrictions on publicly sharing data—e.g. participant privacy or use of data from a third party—those must be specified.

Reviewer #1: Yes

Reviewer #2: Yes

Reviewer #3: Yes

**********

4. Is the manuscript presented in an intelligible fashion and written in standard English?

PLOS Global Public Health does not copyedit accepted manuscripts, so the language in submitted articles must be clear, correct, and unambiguous. Any typographical or grammatical errors should be corrected at revision, so please note any specific errors here.

Reviewer #1: Yes

Reviewer #2: Yes

Reviewer #3: Yes

**********

5. Review Comments to the Author

Please use the space provided to explain your answers to the questions above. You may also include additional comments for the author, including concerns about dual publication, research ethics, or publication ethics. (Please upload your review as an attachment if it exceeds 20,000 characters)

Reviewer #1: Model Fit Requires Stronger Justification

The discussion should better justify why the model remains acceptable despite these shortcomings and explain how poorly fit may influence the validity of the conclusions.

The paper would benefit from clearer definitions distinguishing gender norms, gender attitudes, and gender roles, as these concepts occasionally overlap conceptually.

Interpretation of Non-significant Findings

The discussion provides extensive explanations for non-significant associations, which risks overinterpreting the findings. The interpretation should remain more cautious and emphasize the exploratory nature of these results.

The wording should consistently reflect that only associations not causal pathways can be inferred.

Some proxies used to measure gender norms (club membership, role model, media use) appear to capture broader social participation rather than gender norms specifically.

The authors should better justify these indicators and discuss the possibility of measurement bias.

The rationale for reducing the gender attitudes scale from 32 to 12 items should be summarized briefly in the main manuscript rather than relying mainly on supplementary material.

Some discussion paragraphs are lengthy and repetitive, particularly when explaining unexpected findings. Condensing these sections would improve readability.

Reviewer #2: Reviewer Comments:

1. Abstract:While the topic is very interesting, the abstract lacks methodological clarity. The authors should clearly state **when** and **where** the study was conducted, **how** data collection tools were used, and provide a clear, specific description of the data analysis methods.follow

2. Methodology: The methodology section is currently difficult to follow and lacks transparency. It needs to be rewritten to clearly explain the study design, sampling procedures, data collection process, and statistical analysis plan so that it can be easily understood and reproduced.

3. Conclusion and Recommendations:The conclusion is too general and does not directly reflect the study's key findings. Similarly, the recommendations are not specific enough; they should be actionable, context-specific, and directly derived from the study results.

Reviewer #3: The article “Gendered pathways to adolescent mental health: An empirical assessment of a new conceptual framework” addresses an important topic, builds on a conceptual framework that is a valuable contribution to the literature, and uses an intersectionality-informed SEM approach that is well motivated by previous literature. However, there are some important issues that need to be resolved before the article is accepted for publication.

1. The measurement structure of "Gender Attitudes" is inconsistent across the manuscript. In the main article text, the authors note that they used the 12-item version of the scale, but in the supplementary material, they note that the seven-factor, 20-item CFA model provides the best fit to the data. This is an important discrepancy: the supplementary material says the 20-item version is better, but the article uses the 12-item version without sufficient justification. In addition, the text refers to the scale as the gender attitudes scale, but the supplementary material labels it as gender norms, which could be confusing to readers. Please address these inconsistencies and provide a clear rationale for the final choice of measurement structure. I would also recommend labeling the scale as gender attitudes consistently throughout the main text, tables, and supplementary material rather than going back and forth between gender attitudes and gender norms, especially since the article includes another, separate gender norms construct.

2. Lines 155–159 state that four indicators with loadings between 0.2–0.4 were retained for theoretical relevance to the GAM framework. Please name these four indicators explicitly in the main text and provide a specific theoretical justification for the retention of each, rather than a general reference to the GAM framework. It could also be worthwhile to report a sensitivity analysis excluding these indicators, to show how robust the results are to the decision to keep/exclude these indicators.

3. I am not fully convinced by the operationalization of the gender norms construct. In particular, using things like club participation or having a role model as indicators of higher exposure to gender norms seems like a stretch, and it muddles the interpretation of the results. For example, “higher exposure to gender norms” was found to be associated with higher levels of competencies, but if the items creating the gender norms variable include club participation and having a role model, it may be that adolescents are developing competencies through those relationships for other reasons, not because of gender role exposure. The paper also states that “higher exposure to gender norms was significantly associated with" "fewer hours spent on gendered activities (gender roles construct)”, but again, it may be that things like club participation or use of social media are taking up time, so there is less time to spend on gendered activities. Please provide more justification as to why you chose to include these variables as indicators of gender norm exposure, and consider running sensitivity analyses without some of these indicators because they don’t seem to actually match the construct they are intended to measure, in my opinion.

4. The overall SEM model fit is below threshold on several indices, and this should be discussed more specifically. Please report diagnostics like modification indices to indicate the source(s) of misfit and discuss how this affects confidence in the reported paths.

5. The conclusion overstates what the mediation analysis actually found. Lines 376–378 state: "The gender-related constructs are strongly interlinked, thereby attenuating their individual effects on psychological distress […] The interplay […] should be considered when developing interventions." However, all formal mediation tests were non-significant, and only 5 of 14 total regression paths reached significance, with only one directly predicting the outcome. This "attenuation via interrelation" claim is not confirmed statistically, and drawing these implications for interventions at this stage seems premature. I recommend rephrasing to reflect what was actually shown and framing the proposed ideas as hypotheses for future (potentially longitudinal) research rather than a substantiated conclusion.

6. Peer violence index appears to be dropped without explanation. Line 112 describes the community-level gender norms operationalization as including a peer violence index as one of 5 indicators, and Table 1 lists the peer violence index under Gender Norms descriptives. However, Table 2's CFA for the Gender Norms construct includes only four indicators, and the peer violence index is absent from the table. Please clarify whether/why this variable was excluded from the final model.

7. The R² for psychological distress is a very low value for the paper's primary outcome and central research question (3.8%), but it is only mentioned in a table footnote. Consider discussing this explicitly in the discussion, since it impacts how much weight readers should give to the modeled pathways.

8. In the discussion, when discussing non-significant findings, please use more precise language (e.”g., in the observed but non-significant direction”, "would be consistent with") rather than causal phrasing (e.”g., resulting in better mental health”). The current phrasing risks overstating the strength of evidence for these pathways.

Minor comments:

1. Please ensure correct spelling across all documents related to the paper (e.g., “Communalitites” in the supplementary material).

2. "Herman's Single Factor" should be "Harman's Single Factor Test."

3. "SRMR" vs. "SMSR”: please standardize spelling/abbreviation throughout.

4. Figure 2 shows unlabeled indicator boxes for Gender Norms and Competencies, and it has no indicators visible for Gender Attitudes, despite Table 2 listing four indicators for it. Please ensure the figure is complete and consistent with Table 2.

5. Please clarify how sample weights were constructed given that the GAGE Ethiopia sample is described in the paper as purposefully selected, as well as how this affects the representativeness claim in the strengths and limitations section.

**********

6. PLOS authors have the option to publish the peer review history of their article (what does this mean?). If published, this will include your full peer review and any attached files.

**Do you want your identity to be public for this peer review?**  If you choose “no”, your identity will remain anonymous but your review may still be made public.

For information about this choice, including consent withdrawal, please see our Privacy Policy.

Reviewer #1: **Yes:**  Mohammed Mubarak Bello

Reviewer #2: No

Reviewer #3: No

**********

[NOTE: If reviewer comments were submitted as an attachment file, they will be attached to this email and accessible via the submission site. Please log into your account, locate the manuscript record, and check for the action link "View Attachments". If this link does not appear, there are no attachment files.]

-->Figure Resubmissions:

While revising your submission, we strongly recommend that you use PLOS’s NAAS tool (https://ngplosjournals.pagemajik.ai/artanalysis) to test your figure files. NAAS can convert your figure files to the TIFF file type and meet basic requirements (such as print size, resolution), or provide you with a report on issues that do not meet our requirements and that NAAS cannot fix.

After uploading your figures to PLOS’s NAAS tool - https://ngplosjournals.pagemajik.ai/artanalysis, NAAS will process the files provided and display the results in the "Uploaded Files" section of the page as the processing is complete. If the uploaded figures meet our requirements (or NAAS is able to fix the files to meet our requirements), the figure will be marked as "fixed" above. If NAAS is unable to fix the files, a red "failed" label will appear above. When NAAS has confirmed that the figure files meet our requirements, please download the file via the download option, and include these NAAS processed figure files when submitting your revised manuscript.

# Author comment: Author response to Decision Letter 1

**DOI:** 10.1371/journal.pgph.0006728.r002

4 Sep 2026

Attachment Submitted filename: Response To Reviewers.docx

# Peer review: Decision Letter 1

**DOI:** 10.1371/journal.pgph.0006728.r003

**Authors:** Somayeh Hessam

5 Oct 2026

PGPH-D-26-02116R1

Gendered pathways to adolescent mental health: An empirical assessment of a new conceptual framework

PLOS Global Public Health

Dear Dr. Alaze,

Thank you for submitting your manuscript to PLOS Global Public Health. After careful consideration, we feel that it has merit but does not fully meet PLOS Global Public Health’s publication criteria as it currently stands. Therefore, we invite you to submit a revised version of the manuscript that addresses the points raised during the review process.

Please submit your revised manuscript by Nov 04 2026 11:59PM. If you will need more time than this to complete your revisions, please reply to this message or contact the journal office at globalpubhealth@plos.org. When you're ready to submit your revision, log on to https://www.editorialmanager.com/pgph/ and select the 'Submissions Needing Revision' folder to locate your manuscript file.

Please include the following items when submitting your revised manuscript:

- A letter that responds to each point raised by the editor and reviewer(s). You should upload this letter as a separate file labeled 'Response to Reviewers'.
- A marked-up copy of your manuscript that highlights changes made to the original version. You should upload this as a separate file labeled 'Revised Manuscript with Track Changes'.
- An unmarked version of your revised paper without tracked changes. You should upload this as a separate file labeled 'Manuscript'.

Guidelines for resubmitting your figure files are available below the reviewer comments at the end of this letter.

As the corresponding author, your ORCID iD is verified in the submission system and will appear in the published article. PLOS supports the use of ORCID, and we encourage all coauthors to register for an ORCID iD and use it as well. Please encourage your coauthors to verify their ORCID iD within the submission system before final acceptance, as unverified ORCID iDs will not appear in the published article. *Only*  the individual author can complete the verification step; PLOS staff *cannot*  verify ORCID iDs on behalf of authors.

We look forward to receiving your revised manuscript.

Kind regards,

Somayeh Hessam

Academic Editor

PLOS Global Public Health

Journal Requirements:

If the reviewer comments include a recommendation to cite specific previously published works, please review and evaluate these publications to determine whether they are relevant and should be cited. There is no requirement to cite these works unless the editor has indicated otherwise.

Please review your reference list to ensure that it is complete and correct. If you have cited papers that have been retracted, please include the rationale for doing so in the manuscript text, or remove these references and replace them with relevant current references. Any changes to the reference list should be mentioned in the rebuttal letter that accompanies your revised manuscript. If you need to cite a retracted article, indicate the article’s retracted status in the References list and also include a citation and full reference for the retraction notice.

Additional Editor Comments (if provided):

[Note: HTML markup is below. Please do not edit.]

Reviewers' comments:

Reviewer's Responses to Questions

**Comments to the Author**

1. If the authors have adequately addressed your comments raised in a previous round of review and you feel that this manuscript is now acceptable for publication, you may indicate that here to bypass the “Comments to the Author” section, enter your conflict of interest statement in the “Confidential to Editor” section, and submit your "Accept" recommendation.

Reviewer #1: All comments have been addressed

Reviewer #3: (No Response)

**********

2. Does this manuscript meet PLOS Global Public Health’s publication criteria? Is the manuscript technically sound, and do the data support the conclusions? The manuscript must describe methodologically and ethically rigorous research with conclusions that are appropriately drawn based on the data presented.

Reviewer #1: Yes

Reviewer #3: Yes

**********

3. Has the statistical analysis been performed appropriately and rigorously?

Reviewer #1: Yes

Reviewer #3: Yes

**********

4. Have the authors made all data underlying the findings in their manuscript fully available (please refer to the Data Availability Statement at the start of the manuscript PDF file)?

The PLOS Data policy requires authors to make all data underlying the findings described in their manuscript fully available without restriction, with rare exception. The data should be provided as part of the manuscript or its supporting information, or deposited to a public repository. For example, in addition to summary statistics, the data points behind means, medians and variance measures should be available. If there are restrictions on publicly sharing data—e.g. participant privacy or use of data from a third party—those must be specified.

Reviewer #1: Yes

Reviewer #3: Yes

**********

5. Is the manuscript presented in an intelligible fashion and written in standard English?

PLOS Global Public Health does not copyedit accepted manuscripts, so the language in submitted articles must be clear, correct, and unambiguous. Any typographical or grammatical errors should be corrected at revision, so please note any specific errors here.

Reviewer #1: Yes

Reviewer #3: Yes

**********

6. Review Comments to the Author

Please use the space provided to explain your answers to the questions above. You may also include additional comments for the author, including concerns about dual publication, research ethics, or publication ethics. (Please upload your review as an attachment if it exceeds 20,000 characters)

Reviewer #1: there seems to be a change in analytic sample size, the analytic sample has increased from 1,584 participants in the original manuscript to 1,651 in the revised analysis. Please briefly explain the reason for this change.

Although the revised model has improved, the CFI and TLI remain below conventional thresholds. The authors now acknowledge this appropriately in the limitations section. I suggest maintaining this cautious framing throughout the Discussion and Conclusions and avoiding language that might imply that the proposed framework has been confirmed by these data.

The revised manuscript describes the negative association between female sex and psychological distress as indicating a “protective factor.” Given the cross-sectional design, it would be more accurate to state that female sex was associated with lower psychological distress in this model, without implying a protective or causal effect.

All formal mediation effects remain non-significant. The revised conclusion is more cautious than before, but the phrase that interrelations may be “attenuating” individual effects still reads somewhat mechanistically. I would suggest wording this more conservatively—for example, that the constructs were more strongly associated with one another than with psychological distress, and that the mechanisms underlying these relationships require longitudinal investigation.

Reviewer #3: I thank the authors for their careful revision and detailed response. Several of my comments have been fully addressed, but some comments are only partly addressed, and there are some errors that bear on the paper's main conclusion. I therefore recommend a further round of minor revision.

Responses to previous comments:

Comment 1 (gender attitudes measurement): The main text has been harmonised, but S1 still describes gender attitudes as a 12-item sum score, and Supplementary Tables 4–6 still label the indicators "Gender Norms Scale." In addition, the revised model retains only subscales F1, F2 and F4. Please state which content domains these cover and acknowledge that the construct is now narrower than the validated 20-item scale.

Comment 2 (low loadings): The voice score was part of the competencies construct in the original model but no longer appears in Table 2, and it is not among the removed indicators listed at lines 179–181. Please clarify.

Comment 3 (operationalization of gender norms): The sensitivity analyses are useful, and the estimates are reassuringly stable when club membership or role model is removed individually (Supplementary Tables 5–6). However, the main text (lines 290–293) summarizes these analyses only in terms of model fit. It should also report that the media-only model (Supplementary Table 4) changes two findings: sex is no longer significantly associated with distress (p=0.058), and gender roles becomes significantly associated with distress (p=0.044). Also, the "Outcome: Competencies" rows in Supplementary Table 4 are identical to the main model even though competencies R² falls from 65.2% to 17.1%, which suggests a copy-paste error.

New comment:

Errors in Table 3: Please check the accuracy of the reported numbers for the total effect of gender norms and the total indirect effect - they numbers included seem inconsistent with the confidence intervals presented.

**********

7. PLOS authors have the option to publish the peer review history of their article (what does this mean?). If published, this will include your full peer review and any attached files.

**Do you want your identity to be public for this peer review?**  If you choose “no”, your identity will remain anonymous but your review may still be made public.

For information about this choice, including consent withdrawal, please see our Privacy Policy.

Reviewer #1: **Yes:**  Mohammed Mubarak Bello

Reviewer #3: No

**********

[NOTE: If reviewer comments were submitted as an attachment file, they will be attached to this email and accessible via the submission site. Please log into your account, locate the manuscript record, and check for the action link "View Attachments". If this link does not appear, there are no attachment files.]

Figure Resubmissions:

While revising your submission, we strongly recommend that you use PLOS’s NAAS tool (https://ngplosjournals.pagemajik.ai/artanalysis) to test your figure files. NAAS can convert your figure files to the TIFF file type and meet basic requirements (such as print size, resolution), or provide you with a report on issues that do not meet our requirements and that NAAS cannot fix.

After uploading your figures to PLOS’s NAAS tool - https://ngplosjournals.pagemajik.ai/artanalysis, NAAS will process the files provided and display the results in the "Uploaded Files" section of the page as the processing is complete. If the uploaded figures meet our requirements (or NAAS is able to fix the files to meet our requirements), the figure will be marked as "fixed" above. If NAAS is unable to fix the files, a red "failed" label will appear above. When NAAS has confirmed that the figure files meet our requirements, please download the file via the download option, and include these NAAS processed figure files when submitting your revised manuscript.

# Author comment: Author response to Decision Letter 2

**DOI:** 10.1371/journal.pgph.0006728.r004

9 Oct 2026

Attachment Submitted filename: Response_To_Reviewers_auresp_2.docx

# Peer review: Decision Letter 2

**DOI:** 10.1371/journal.pgph.0006728.r005

**Authors:** Somayeh Hessam

18 Oct 2026

Gendered pathways to adolescent mental health: An empirical assessment of a new conceptual framework

PGPH-D-26-02116R2

Dear None Alaze,

We are pleased to inform you that your manuscript 'Gendered pathways to adolescent mental health: An empirical assessment of a new conceptual framework' has been provisionally accepted for publication in PLOS Global Public Health.

Before your manuscript can be formally accepted you will need to complete some formatting changes, which you will receive in a follow up email. A member of our team will be in touch with a set of requests.

Please note that your manuscript will not be scheduled for publication until you have made the required changes, so a swift response is appreciated.

IMPORTANT: The editorial review process is now complete. PLOS will only permit corrections to spelling, formatting or significant scientific errors from this point onwards. Requests for major changes, or any which affect the scientific understanding of your work, will cause delays to the publication date of your manuscript.

If your institution or institutions have a press office, please notify them about your upcoming paper to help maximize its impact. If they'll be preparing press materials, please inform our press team as soon as possible -- no later than 48 hours after receiving the formal acceptance. Your manuscript will remain under strict press embargo until 2 pm Eastern Time on the date of publication. For more information, please contact globalpubhealth@plos.org.

Thank you again for supporting Open Access publishing; we are looking forward to publishing your work in PLOS Global Public Health.

Best regards,

Somayeh Hessam

Academic Editor

PLOS Global Public Health

***********************************************************

Reviewer Comments (if any, and for reference):

Reviewer's Responses to Questions

**Comments to the Author**

1. If the authors have adequately addressed your comments raised in a previous round of review and you feel that this manuscript is now acceptable for publication, you may indicate that here to bypass the “Comments to the Author” section, enter your conflict of interest statement in the “Confidential to Editor” section, and submit your "Accept" recommendation.

Reviewer #1: All comments have been addressed

Reviewer #3: All comments have been addressed

**********

2. Does this manuscript meet PLOS Global Public Health’s publication criteria? Is the manuscript technically sound, and do the data support the conclusions? The manuscript must describe methodologically and ethically rigorous research with conclusions that are appropriately drawn based on the data presented.

Reviewer #1: Yes

Reviewer #3: Yes

**********

3. Has the statistical analysis been performed appropriately and rigorously?

Reviewer #1: Yes

Reviewer #3: Yes

**********

4. Have the authors made all data underlying the findings in their manuscript fully available (please refer to the Data Availability Statement at the start of the manuscript PDF file)?

The PLOS Data policy requires authors to make all data underlying the findings described in their manuscript fully available without restriction, with rare exception. The data should be provided as part of the manuscript or its supporting information, or deposited to a public repository. For example, in addition to summary statistics, the data points behind means, medians and variance measures should be available. If there are restrictions on publicly sharing data—e.g. participant privacy or use of data from a third party—those must be specified.

Reviewer #1: Yes

Reviewer #3: Yes

**********

5. Is the manuscript presented in an intelligible fashion and written in standard English?

PLOS Global Public Health does not copyedit accepted manuscripts, so the language in submitted articles must be clear, correct, and unambiguous. Any typographical or grammatical errors should be corrected at revision, so please note any specific errors here.

Reviewer #1: Yes

Reviewer #3: Yes

**********

6. Review Comments to the Author

Please use the space provided to explain your answers to the questions above. You may also include additional comments for the author, including concerns about dual publication, research ethics, or publication ethics. (Please upload your review as an attachment if it exceeds 20,000 characters)

Reviewer #1: The previous concerns and comments raised from previous review has been substantially addressed, and the submission is now well polished. Categorical statements have been rephrased appropriately. They have also clarified their limitations.

Reviewer #3: The authors have sufficiently addressed my previous comments.

**********

7. PLOS authors have the option to publish the peer review history of their article (what does this mean?). If published, this will include your full peer review and any attached files.

**Do you want your identity to be public for this peer review?**  If you choose “no”, your identity will remain anonymous but your review may still be made public.

For information about this choice, including consent withdrawal, please see our Privacy Policy.

Reviewer #1: No

Reviewer #3: No

**********
